# A Synthetic Method to Hydrogen Radicals

**DOI:** 10.1021/jacs.6c07870

**Published:** 2026-08-25

**Authors:** Nils J. Flodén, Alberto Collauto, Peter H. Seeberger, Roopender Kumar

**Affiliations:** † Department of Biomolecular Systems, Max-Planck-Institute of Colloids and Interfaces, 14476, Potsdam, Germany; ‡ Department of Chemistry and Centre for Pulse EPR Spectroscopy, Imperial College London, Molecular Sciences Research Hub, 82 Wood Lane, W12 0BZ, London, U.K.; § Institute of Chemistry and Biochemistry, Freie Universität Berlin, 14195, Berlin, Germany; ∥ Department of Chemistry, 4919 University College London, 20 Gordon St., WC1H 0AJ, London, U.K.

## Abstract

Atomic hydrogen (H•), the union of an electron and a proton, is the smallest radical of the universe. Over the past century, advanced techniques such as electric discharge and mercury UV sensitization enabled a fundamental understanding of the reactivity of H• but fell short of providing synthetic methods to exploit its intriguing properties. Here we report a light-mediated method to access hydrogen radicals under synthetically useful conditions. This strategy enabled the mild, metal-free reduction of highly functionalized alkenes and halides. Mechanistic, EPR, and DFT studies suggest hydrogen radical generation via a Rydberg radical intermediate, with potential applications across the chemical and biological sciences.

## Graphical abstract

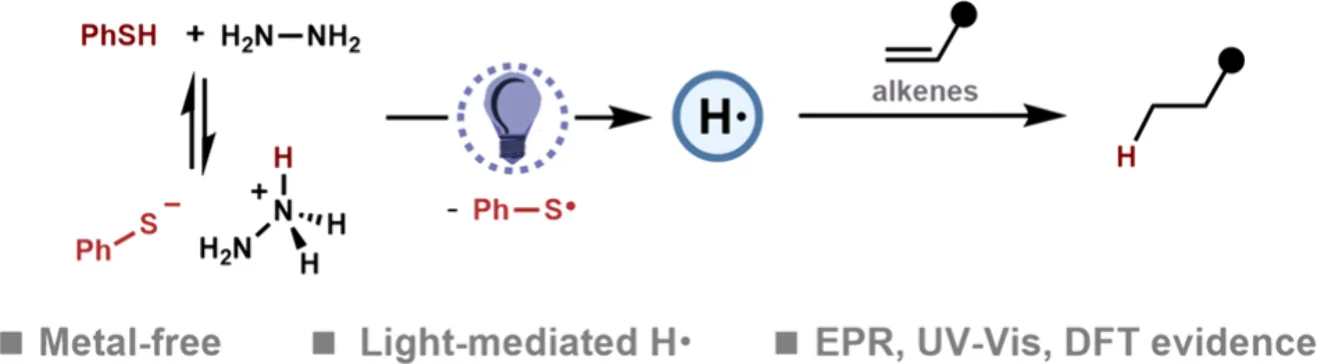

Atomic hydrogen (H•) has captured the imagination of scientists and philosophers alike.^1^ The smallest member of the periodic table with an atomic radius of 53 pm, it was once proposed by William Prout as the fundamental object of which all other elements were the sum total of.^2^ While Prout’s hypothesis was ultimately refuted, modern science has firmly established the importance of atomic hydrogen in a gamut of disciplines spanning from quantum physics to the origin of life.^3–9^ Key to these advances was a set of experiments that under extreme conditions led to the discovery of H•. In 1912, Langmuir observed that upon heating H_2_ to >2000K over a tungsten filament, it homolyzed to form H• that rapidly adsorbed onto a glass surface (Scheme 1A).^10–12^ New methods to generate atomic hydrogen such as electron- or γ-radiation, microwave discharge, or UV light followed;^13–18^ all ingenious, yet as concluded by Crabtree in 1991,^19^ none synthetically tenable.

**Scheme 1:**
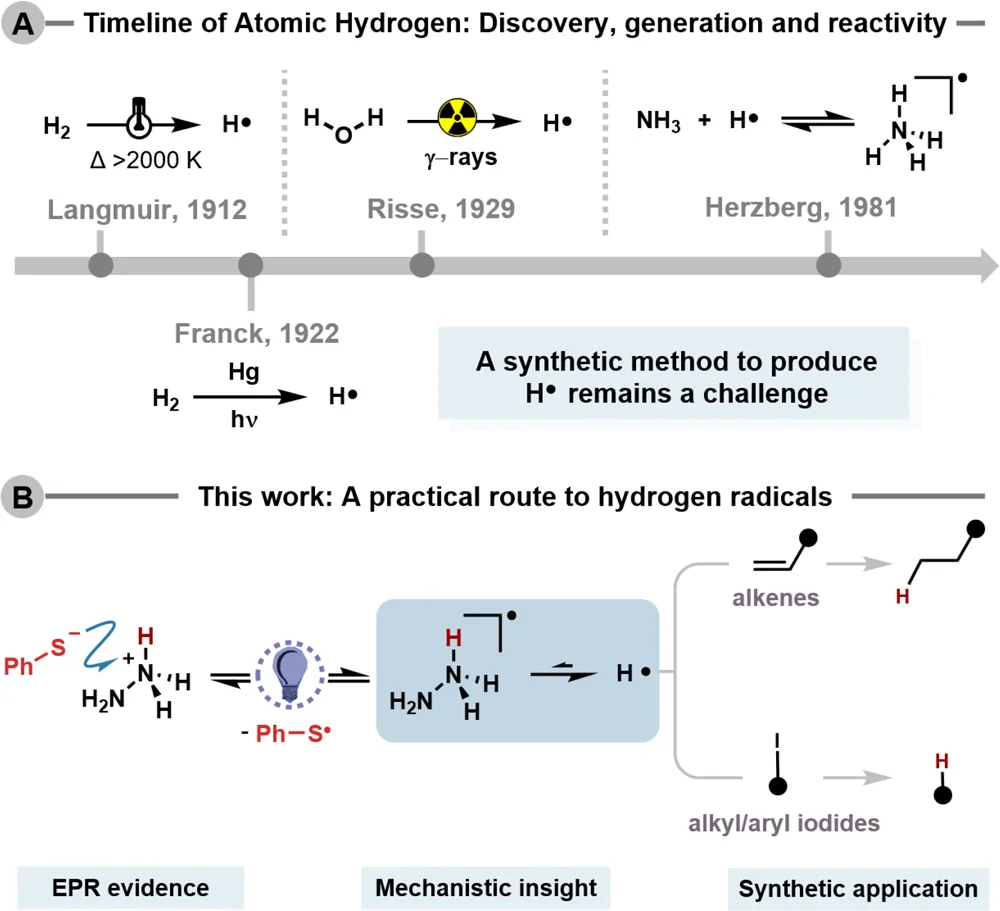
Chemistry of Atomic Hydrogen

Seeking a solution to the challenge of generating hydrogen radicals under mild conditions (Scheme 1B), our initial approach was inspired by the intriguing properties of NH_4_•. Known as a Rydberg radical,^20^ it is a short-lived intermediate with a picosecond lifetime before undergoing α-fragmentation to form NH_3_ and H•.^21,22^ We speculated that since NH_3_ and thiophenol exhibit similar p*K*_a_ (10.5 (NH_4_^+^) & 10.3 (PhSH) in DMSO),^23,24^ they would form an ion-pair which could absorb light and promote electron transfer from the thiophenolate to the ammonium cation, affording the H•, whose most pertinent feature is its reaction with alkenes.^25,26^ However, combining thiophenol and ammonia with alkene **1a** only afforded **2a** in 3% yield (Scheme 2A).

**Scheme 2:**
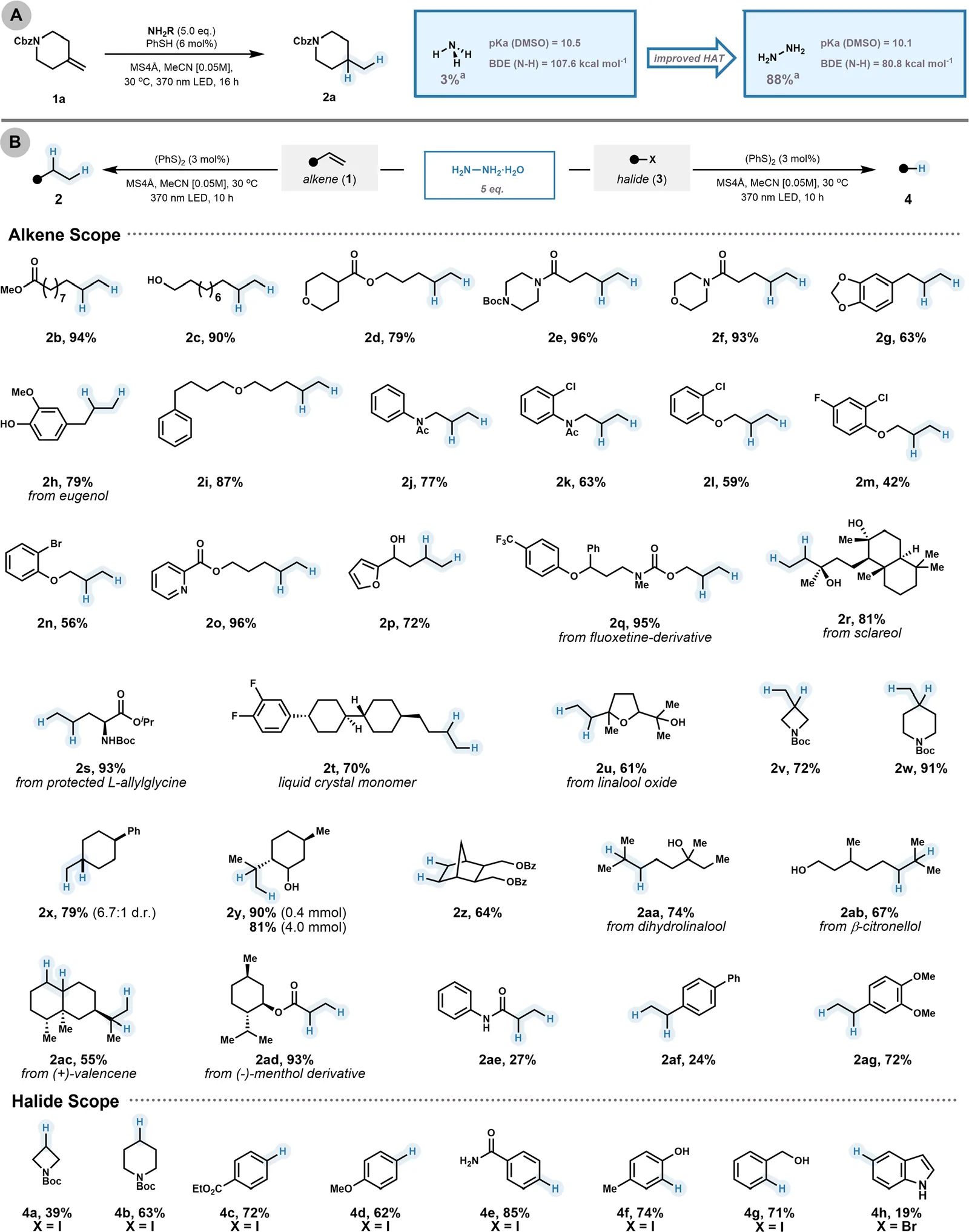
Scope

A) Initial Lead and Final Conditions of Radical Hydrogenation. B) Scope of Hydrogenation and Hydrodehalogenation.
a Yield by ^1^H NMR using 1,1,2,2-tetrachloroethane as an internal standard.

Anticipating that regeneration of PhSH from PhS• was hampered by the high bond dissociation energy of ammonia (BDE (N–H) = 107.6 kcal mol^–1^), we reasoned that HAT from hydrazine (p*K*_a_ = 10.1 in DMSO, BDE (N–H) = 80.8 kcal mol^–1^) by PhS· would be more feasible.^23,24,27^ Fortunately, after extensive optimization of the reaction conditions (Tables S1–S9), hydrazine hydrate proved a potent replacement for ammonia, affording the desired product in 88% yield (**2a**, Scheme 2A).

The scope of the hydrogenation reaction was then probed employing alkenes carrying alcohols, heterocycles, ethers, esters and protected amines, affording the desired hydrogenation products in excellent yields (**2b**–**2j**). To avoid the stench of PhSH, the scope was carried out with (PhS)_2_, which upon homolysis and HAT from hydrazine forms PhSH *in situ*. The reaction exhibits good mass balance, with the remainder being unreacted starting material. Reduced products **2k**–**2n** highlight the mechanistic divergence from previous work on “formal hydrogen atom”-like species,^28^ where the halogen instead of the alkene was reduced, suggesting that neither H• nor a “formal” variant thereof was the origin of the previously reported reactivity. The present conditions afforded the corresponding hydrogenation products with only minor hydrodehalogenation, in-line with the slower reaction rate of H• with aryl chlorides.^29^ Aromatic (**2o**–**2p**) and aliphatic (**2u**–**2w**) heterocycles were well-tolerated, and no racemization was observed in compounds presenting multiple stereochemical centers (**2r, 2x–2z**). Di- and trisubstituted alkenes all yielded to the reaction conditions to afford reduced products in useful yields (**2v–2ac**) even at 10-fold scale (**2y**, 4.0 mmol scale). The lower yields for internal versus external alkenes can be understood by a 2.1-fold higher rate of addition to terminal alkenes.^30^ **2x** was accessed in 6.7:1 d.r., highlighting the preference for the thermodynamic product under radical conditions while concerted hydrogenation with diazene afforded 1.1:1 d.r. (see SI). The hydrogenation is selective for the alkene in **1a** without affecting the Cbz functionality, underscoring complementarity versus Pd-catalyzed methods. Activated alkenes (**2ad–2ag**) provided lower yields of the reduced product, likely due to slower trapping of the stabilized radical resulting from hydrogen atom addition across the C=C bond leading to competing dimerization (e.g., 30% of dimer for **2af**) or polymerization. Finally, alkyl and aryl iodides and bromides (**3a**–**3h**) could be reduced under the same reaction conditions in good yields to afford hydrodehalogenated compounds (**4a**–**4h**). The hydrodehalogenation reaction herein mechanistically complements previous studies, which proceeds via an electron-donor–acceptor mechanism.^31,32^

We then set out to identify the key reactive intermediates and how they were generated. A set of control experiments established that the reaction does not proceed in the absence of hydrazine, diphenyl disulfide, or light (Scheme 3A, entries 1–3 and 5). Removing 4Å molecular sieves lowered the yield to 58% (entry 4). The reaction was also sensitive to the emission wavelength of the LED, with a 390 nm lamp affording 35% yield (entry 6). Only MeCN is a productive solvent, with other solvents affording significantly less product (entry 7; see also Table S4). This “uniqueness” of acetonitrile may rest in part on its known inertness to highly reactive electrophilic radicals (e.g., HO·) and a high polarity to sufficiently solvate ion-pair species. Investigation of substituted thiophenols revealed that the parent thiophenol and 4-methoxythiophenol strike a balance between ion-pair formation and reducing power (entries 1 and 8), with electronically extreme variants affording reduced yields (entry 9). Thiophenols with too strongly electron-donating substituents (e.g., 4-NMe_2_) increase reducing power but disfavor ion-pair formation due to a higher p*K*_a_. On the other hand, thiophenols carrying electron-withdrawing groups (e.g., 4-CN and 4-NO_2_) form strongly colored electron donor-acceptor complexes but are less reducing. Deuterium-incorporation experiments and a kinetic isotope effect of k_H_/k_D_ = 2.45 from initial rate measurements of parallel reactions using alkene **1j** suggested that hydrazine is the key reducing species providing hydrogen (deuterium) atoms to the substrate and that an N–H/N–D cleavage is a part of the rate-determining step (Scheme 3B–C).

**Scheme 3:**
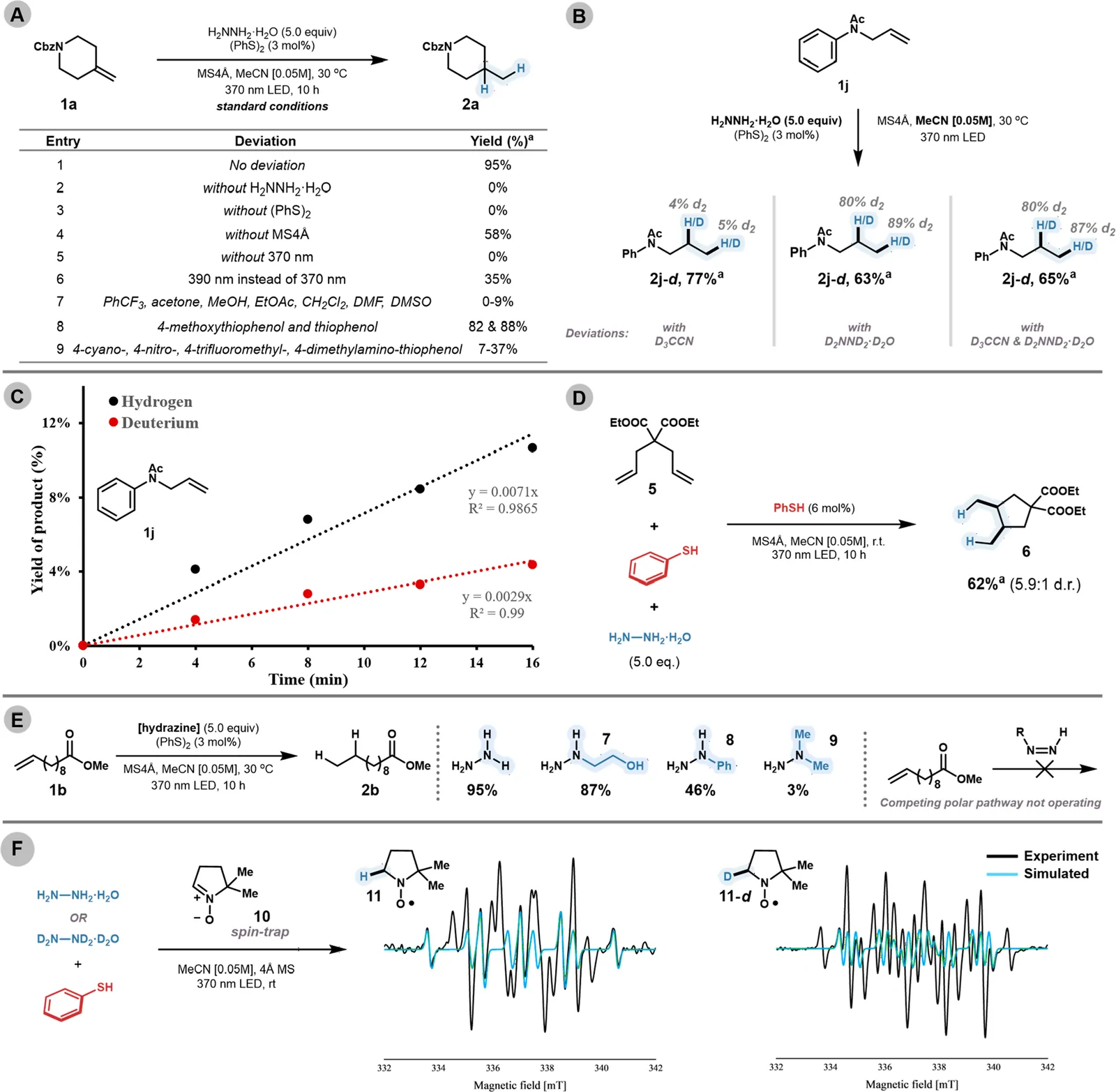
Control Reactions and Mechanistic Investigations

A) Control Reactions. B) Deuterium Labelling. C) Parallel KIE. D) Radical Cyclization. E) Reactions under Standard Conditions Varying the Hydrazine. F) EPR Trapping of H• and D• under the Reaction Conditions.
a Yield by ^1^H NMR using 1,1,2,2-tetrachloroethane as an internal standard.

In considering the role of hydrazine, we were mindful that diazene (HN=NH), a known hydrogenation reagent, can be produced *in situ* from H_2_NNH_2_.^33^ To investigate this possibility, radical cyclization probe **5** was tested, affording the cyclized product **6** in 62% yield (Scheme 3D). Furthermore, monosubstituted hydrazines (**7** & **8**) provided **2b** in excellent to good yields (Scheme 3E). The formation of diazene from **8** would require the elimination of a phenyl radical (112.9 kcal mol^–1^), a more reactive intermediate than H• (BDE = 104.2 kcal mol^–1^).^27^ The poor yield with disubstituted hydrazine **9** likely reflects the higher N–H BDE (85.0 vs 80.8 kcal mol^–1^ for hydrazine), surpassing that of PhSH (83.5 kcal mol^–1^) and thereby suppressing PhSH turnover.^27^ Together, this argues against a diazene intermediate and supports a radical pathway.

Hydrogen radicals are rarely invoked in synthetic chemistry despite much more reactive radicals such as phenyl, amidyl (BDE ≤ 111 kcal mol^–1^), and ·CF_3_ (BDE = 106.4 kcal mol^–1^) being commonplace.^27^ Nevertheless, H• is known to be trapped by spin-trapping agents such as 5,5-dimethyl-1-pyrroline-*N*-oxide (DMPO, **10**),^34,35^ affording persistent radicals observable by electron paramagnetic resonance (EPR) spectroscopy. To investigate whether H• is present under our reaction conditions, we irradiated hydrazine hydrate, thiophenol, 4Å molecular sieves, and DMPO in anhydrous MeCN under argon with a 370 nm LED for 5 min at room temperature and recorded the CW-EPR spectrum of the full reaction mixture (Scheme 3F & SI). It revealed the presence of the DMPO–H• adduct **11**, with coupling constants (aN = 1.49 mT, aH = 1.95 mT) consistent with those reported in literature (aN = 1.44 mT, aH = 1.89 mT) and a characteristic, unique pattern originating from the hyperfine coupling with two equivalent protons.^36–38^ Similar results were obtained when H_2_NNH_2_ was replaced with D_2_NND_2_, affording both DMPO–H• (**11**) and DMPO–D• (**11-** ***d***) with the obtained values of aD in line with what has been reported in the literature.^37–39^ Control experiments eliminating any of the reaction components led to the disappearance of the DMPO-H• signal (see SI).

We then sought to pinpoint the origin of hydrogen radicals. In addition to the hypothesized H_2_NNH_3_• (***Int-I***), it is also conceivable that H_2_NNH• (***Int-II***) could display similar reactivity (Scheme 4A). Probing the energetics of these two species by DFT calculations, the fragmentation of H_2_NNH_3_• was found highly exergonic (ΔG = −14.2 kcal mol^–1^) while that of H_2_NNH• is endergonic (ΔG = 45.4 to 48.4 kcal mol^–1^). Intriguingly, the use of hydrazine as corrosion protection in the primary cooling feed of nuclear reactors supports H_2_NNH_3_• as a reactive intermediate. In that context, H_2_NNH_3_^+^ reacts with solvated electrons originating from γ-irradiated water to afford H_2_NNH_3_•. This in turn fragments to H• which was trapped with a rate constant of *k* = 1.6 × 10^8^ M^–1^ s^–1^.^40^ To rule out the involvement of H_2_NNH• (***Int-II***) or HN=N• (***Int-III***), we noted that H_2_NNH• has previously been generated and observed through EPR-spectroscopy, using peroxides to abstract hydrogen atoms from hydrazine.^41^

**Scheme 4:**
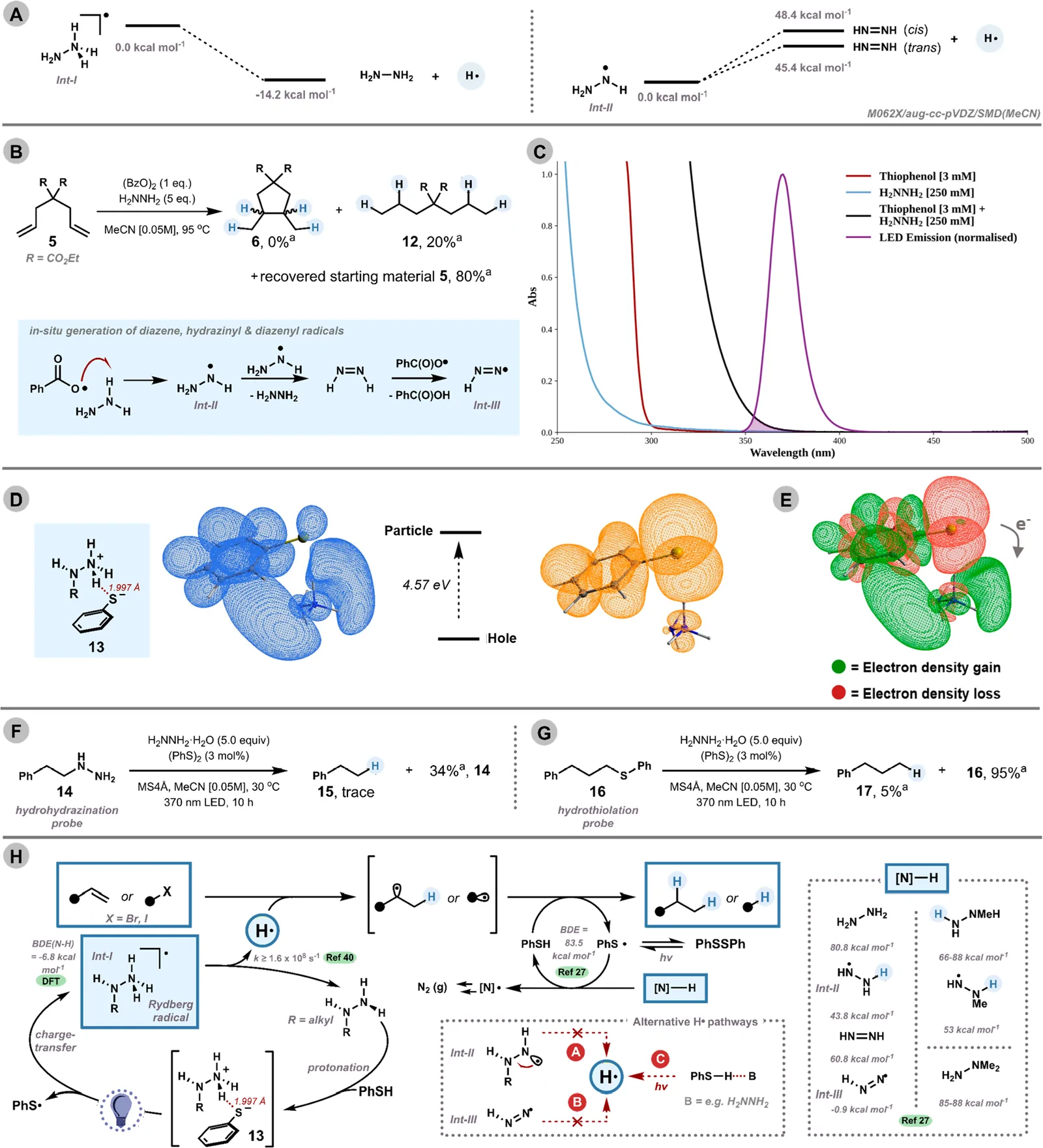
Mechanistic Investigations and Proposed Mechanism

A) DFT Studies of ***Int-I*** & ***Int-II*** as Sources of H•. B) Experimental Investigation of ***Int-II*** & ***Int-III***. C) UV-Vis of Proposed Charge-Transfer Interaction in MeCN. D) Particle and Hole Natural Transition Orbitals of Ion-Pair **13**. E) Charge-Density Difference Plot of Electron Transfer in Ion-Pair **13**. F-G) Probes of Radical Hydrogenation Via Hydrohydrazination and Hydrothiolation. H) Mechanistic Proposal.
a Yield by ^1^H NMR using 1,1,2,2-tetrachloroethane as an internal standard.
b Overlap between LED emission and ion-pair absorption is highlighted in purple.
c Arrow indicates direction of charge transfer.

However, when benzoyl peroxide was combined with hydrazine and **5**, 20% of the linear reduced compound **12** was obtained but no cyclization was observed (Scheme 4B). This outcome is consistent with a polar reduction by HN=NH, produced through disproportionation of H_2_NNH•.^42^ Crucially, the absence of cyclization product **6** rules out the possibility that H_2_NNH• or HN=N•, whether through unimolecular fragmentation to form H• or as a "H• mimic" through direct reaction with an alkene, is a competent intermediate for hydrogen atom delivery.^43^

With the above data and literature precedent suggesting that Rydberg radical H_2_NNH_3_• might be an intermediate, we probed the aforementioned hypothesis that a charge-transfer event between a thiophenolate and a hydrazinium ion could be the source of ***Int-I*** *.* A significant red-shift in the UV–visible absorption spectrum, suggestive of a charge-transfer event, was observed when thiophenol and hydrazine were mixed compared to the individual components (Scheme 4C). The spectroscopically observed excited states were then studied computationally at the CAM-B3LYP/aug-cc-pVDZ level of theory (Scheme 4D). Using diffuse functions to enhance accuracy for Rydberg radicals, an analysis of the three lowest excited states of ion-pair **13** revealed two transitions (4.57 and 4.67 eV) displaying clear charge-transfer properties, with a charge transfer fraction of up to 32%. Transforming the molecular orbitals of these excited state transitions using Martin’s natural transition orbital (NTO) representation^44^ revealed the NTO-hole to reside on the thiophenolate donor while the NTO-particle representation had substantial localization on the hydrazinium fragment.

This was further corroborated by a charge-density difference plot, depicting the depletion of electron density from the thiophenolate and to the hydrazinium fragment (Scheme 4E).

Finally, we considered a scenario where initial hydrohydrazination or hydrothiolation reactions could be followed by C–N/C–S bond cleavage. However, alkyl hydrazine **14** (the product of a hypothetical hydrohydrazination) decomposed under the reaction conditions to afford a complex mixture with only trace amounts of products formed (Scheme 4F), and sulfide **16** (the product of a hypothetical hydrothiolation) afforded only 5% of alkane (Scheme 4G), arguing against such pathways.

Taken together, the EPR, UV–vis, DFT, deuterium-labeling, mechanistic probes, and literature precedent allow us to propose a light-mediated mechanism for the generation of hydrogen radicals (Scheme 4H). H_2_NNH_3_• (***Int-I***) formed from **13** upon absorption of light fragments to H•, which rapidly reacts with alkenes. A lifetime of 13 ps for the related NH_4_• argues against direct reaction with alkenes at the relevant concentrations [≤0.05M].^45^ Hydrazine is catalytic in its interaction with one molecule of thiophenol to afford H• but is consumed to hydrazine radical ***Int-II*** in order to convert PhS• back to PhSH. A high pressure is built up during the reaction, as two molecules of ***Int-II*** undergo disproportionation (or subsequent HAT by PhS• from ***Int-II*** and other hydrazine-based intermediates) to provide N_2_ as the ultimate byproduct. We cannot yet rule out electron transfer between thiophenol/thiophenolate-derived complexes (c.f. path C, Scheme 4H), but the weak nature of such interactions makes them less plausible.

In summary, we have reported a method to access hydrogen radicals under synthetically useful conditions. Mechanistic studies suggest a H•/Rydberg duality which could find diverse application in the chemical and biological sciences. Ultimately, this work resolves a long-standing challenge to make the smallest radical of the universe accessible for molecular assembly.

## Supplementary Material



## Notes

Additional experimental, computational and spectroscopical details, materials, methods and full characterization data (^1^H, ^13^C, ^19^F NMR and HRMS) for all new compounds (PDF)

## Abbreviations

Ac: acetyl
Boc: *tert*-butyloxycarbonyl
Cbz: carboxybenzyl
CT: charge-transfer
EPR: electron paramagnetic resonance
NTO: natural transition orbital.
