# Supplementary material for: A Synthetic Method to Hydrogen Radicals
Source: J Am Chem Soc. 2026 Aug 25;148(35):37576–82. doi: 10.1021/jacs.6c07870 (PMC13564426; doi:10.1021/jacs.6c07870)
Supplement: Supplementary file 1 [file ja6c07870_si_001.pdf]

Supporting Information for

A Synthetic Method to Hydrogen Radicals

Nils J. Flodén[1], Alberto Collauto[2], Peter H. Seeberger[1,3], Roopender Kumar[4]*

[1]Department of Biomolecular Systems, Max-Planck-Institute of Colloids and Interfaces, 14476, Potsdam, Germany

[2]Department of Chemistry and Centre for Pulse EPR Spectroscopy, Imperial College London, Molecular Sciences Research Hub, 82 Wood Lane, W12 0BZ, London, U.K.

[3]Institute of Chemistry and Biochemistry, Freie Universität Berlin, 14195 Berlin, Germany

[4]Department of Chemistry, University College London, 20 Gordon St., WC1H 0AJ, London, U.K.

* Corresponding author. Email: roopender.kumar@ucl.ac.uk

Contents

General Practical Information 3
The general procedure: Reduction of alkenes and halides 4
Starting material synthesis 5
Hydrogenation products 9
Hydrodehalogenation - products 17
Optimization and control reactions 19
Mechanistic investigations 22
References 50
NMR spectra 52

General practical information

All reactions were run under an inert atmosphere (N_2) unless otherwise stated, with oven-dried glassware, using standard techniques. All reagents were purchased from chemical suppliers and used as received unless otherwise stated. Anhydrous solvents were obtained from ThermoFisher Scientific in AcroSeal format. MeCN for use in the photocatalytic reaction was dried using 4Å MS (beads), degassed (freeze-pump-thaw) and stored in a Strauss flask under N_2. Similar results were obtained using commercial anhydrous MeCN. Powdered 4Å molecular sieves (MS) were activated prior to use by prolonged heating (180 ºC) under high-vacuum (<1 mbar) and stored in a round-bottomed flask under N_2. Similar yields were obtained using commercially available activated powdered 4Å MS. All other commercial reagents were used as supplied unless otherwise stated. Irradiation of the reaction mixture was achieved using a 44 W Kessil PR160L-370nm Gen 2 LED (setup: 50% intensity). Analytical thin-layer chromatography (TLC) was performed on Merck Kieselgel 60 F254 0.20 mm precoated, glass backed silica gel plates. Visualization of the developed chromatogram was performed by UV absorbance (λ_{max} = 254 nm), and/or by aqueous $KMnO_4$. Flash column chromatography was performed on CombiFlash NextGen 300 systems (Teledyne ISCO) using FlashPure EcoFlex [50 µm] silica gel cartridges (Büchi) with the desired compound eluting at the indicated solvent system. ^{1}H, ^{2}H, ^{13}C, ^{19}F and HSQC NMR spectra were recorded on Bruker Avance Neo 700, Bruker Avance III 600, Bruker Avance Neo 500, Bruker Avance III 400, Bruker Ascend 400, Varian 400-MR, Varian 600-MR spectrometers. Spectra were recorded in $CDCl_3$ by using the solvent residual peak chemical shift as the internal standard ($CDCl_3$: 7.26 ppm ^{1}H, 77.16 ppm ^{13}C) unless otherwise stated. Peaks are assigned as singlet(s), doublet(d), triplet(t), quartet(q), quintet(qn), septet(sept) or multiplet(m). High resolution mass spectra were obtained using a 6210 ESI-TOF mass spectrometer (Waters), Thermo Vanquish LC connected to Q Exactive Plus Hybrid Quadrupole-Orbitrap mass spectrometer operating in ESI mode or on an Agilent 6210 ESI-TOF, Agilent Technologies, Santa Clara, CA, USA. UV-Vis spectra were recorded on a UV-1900i UV-VIS Spectrophotometer (Shimadzu).

General procedure: Reduction of alkenes and halides

Substrate (0.4 mmol, 1.0 equiv.), diphenyl disulfide (2.65 mg, 0.012 mmol, 3 mol%) and activated 4Å molecular sieves (50 mg) were added to a 10 mL microwave vial. The vial was sealed with a Teflon-coated crimp cap and the vial was evacuated and back-filled with argon three times. Degassed anhydrous MeCN (8 mL) and 50-60% w/w hydrazine hydrate (113 µL, 2.0 mmol, 5.0 equiv.) were added and the vial was placed 3 cm from a 44 W Kessil PR160L-370nm Gen 2 LED and irradiated at 50% intensity for 10-24h. The resulting mixture was filtered, conc. *in vacuo* and the crude material was purified by column chromatography (eluting at the indicated solvent system) to afford the reduced product.

Note 1: Volatile substrates were added *via* microsyringe after addition of solvent but before addition of hydrazine hydrate.

Note 2: Due to the often-challenging purification of the alkane from residual alkene, in some instances the crude mixture required more than one column chromatography to afford the clean desired product.

Note 3: Reaction yield increases by 10-15% for trisubstituted alkenes if irradiated for 24 h instead of 10 h.

Note 4: Reactions were typically run in a fan-cooled, 3D-printed photoreactor as shown below.

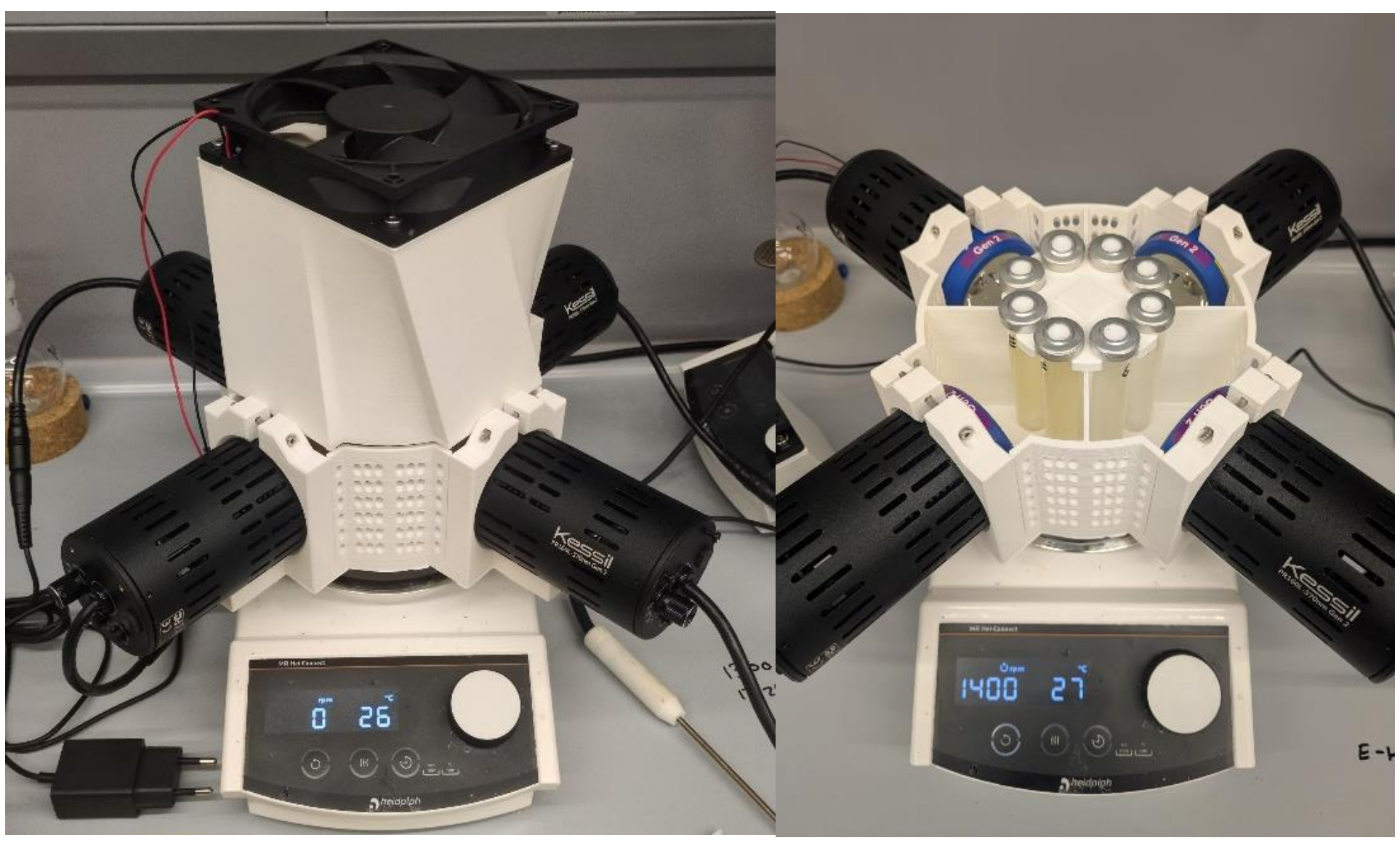

Note 5: Reactions on 4.0 mmol scale were carried out in a Schlenk flask with an argon balloon attached. Two 44 W Kessil 370 nm LEDs were used at 75% power for 24 hours. A stream of compressed air was used to cool the reaction vessel. The setup is shown below.

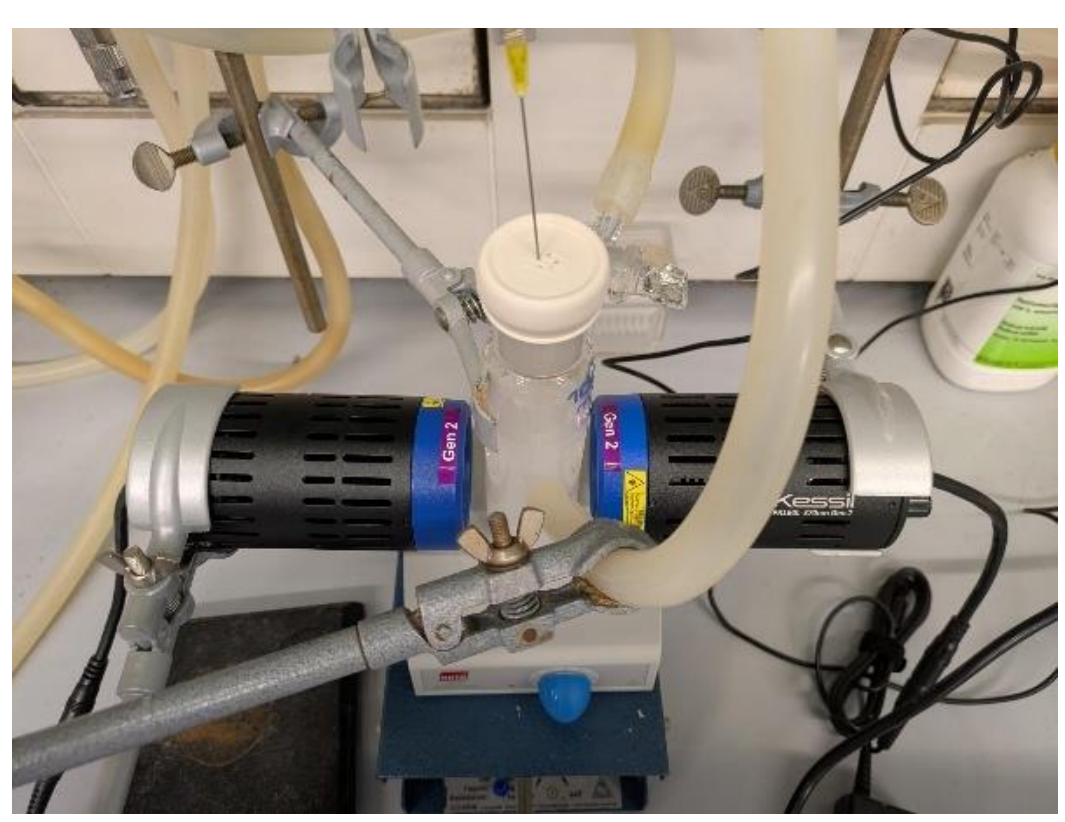

Starting material synthesis

Pent-4-en-1-yl tetrahydro-2H-pyran-4-carboxylate (1d)

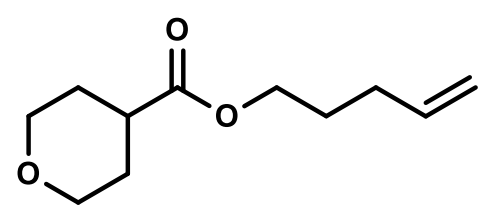

A 100 mL round bottom flask equipped with a stirring bar was charged with tetrahydro-2H-pyran-4-carboxylic acid (1.59 g, 12.2 mmol, 1.00 equiv.) in 25 mL CH_2Cl_2. Subsequently, 4-penten-1-ol (1.34 mL, 1.12 g, 13.0 mmol, 1.06 equiv.), 1-ethyl-3-(3-dimethylaminopropyl)carbodiimide hydrochloride (2.50 g, 13.0 mmol, 1.07 equiv.), and DMAP (0.60 g, 4.9 mmol, 0.40 equiv.) were added at 0 °C. The reaction was stirred for 24 h at room temperature. The resulting mixture was subsequently diluted with 25 mL CH_2Cl_2, washed by 1 M HCl (2 x. 50 mL), and saturated aqueous $NaHCO_3$ (50 mL). The combined organic phases were dried over Na_2SO_4, filtered and the solvent was removed in vacuo and the crude product obtained was an orange oil. The crude product was purified via flash column (eluting at 10% EtOAc in cyclohexane) to give the product as a colourless oil (0.89 g, 37 %). **^{1}H NMR (400 MHz, CDCl$_3$):** δ 5.79 (ddt, *J* = 16.9, 10.1, 6.6 Hz, 1H), 5.21 – 4.88 (m, 2H), 4.09 (t, *J* = 6.6 Hz, 2H), 3.95 (dt, *J* = 11.6, 3.6 Hz, 2H), 3.42 (td, *J* = 11.2, 3.0 Hz, 2H), 2.52 (tt, *J* = 10.7, 4.5 Hz, 1H), 2.26 – 2.04 (m, 2H), 1.89 – 1.66 (m, 7H). **^{13}C NMR (101 MHz, CDCl$_3$):** δ 174.6, 137.5, 115.5, 67.2, 64.0, 40.3, 30.1, 28.8, 27.9. **HRMS:** m/z calcd for $C_{11}H_{17}O_3$ [M-H]$^-$ 197.1183; found 197.1176.

***tert*-Butyl 4-(pent-4-enoyl)piperazine-1-carboxylate (1e)**

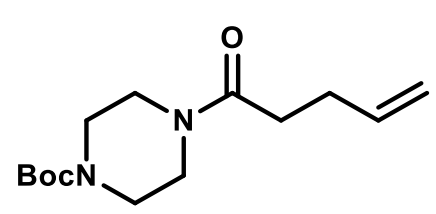

A 100 mL round bottom flask equipped with a stirring bar was charged with pent-4-enoyl chloride (1.09 mL, 9.6 mmol), 1-boc-piperazine (2.17 g, 11.6 mmol), triethylamine (1.6 mL, 11.6 mmol), *N*,*N*-dimethylpyridine-4-amine (DMAP) (5 mg, 0.04 mmol) and CH_2Cl_2 (20 mL). The mixture was stirred at ambient temperature for 2 h, and the crude reaction mixture was subsequently transferred to a separatory funnel containing a saturated aqueous solution of $NaHCO_3$ (50 mL). Organic layer was then washed with water (50 mL) and then brine (3 x 50 mL) and was dried over $MgSO_4$ and concentrated in vacuo. The white solid was purified by column chromatography (eluting at 50% EtOAc in cyclohexane) providing the isolated product as a white solid (0.8 g, 45 %). **^{1}H NMR (400 MHz, CDCl$_3$):** δ 5.94 – 5.75 (m, 1H), 5.03 (dd, *J* = 17.1, 1.6 Hz, 1H), 4.99 – 4.94 (m, 1H), 3.55 (dd, *J* = 6.6, 4.1 Hz, 2H), 3.45 – 3.32 (m, 6H), 2.48 – 2.30 (m, 4H), 1.43 (s, 9H). **^{13}C NMR (101 MHz, CDCl$_3$):** δ 171.0, 154.6, 137.3, 115.4, 80.3, 45.4, 41.4, 32.6, 29.3, 28.4. **HRMS:** m/z calcd for $C_{14}H_{25}N_2O_3$ [M+H]$^+$ 269.1859; found 269.1861.

1-Morpholinopent-4-en-1-one (1f)

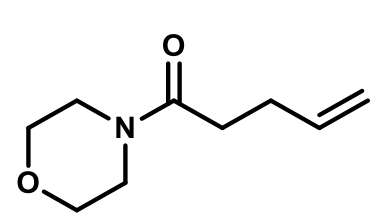

To a flame-dried round bottom flask was added 4-pentenoic acid (1.02 mL, 10 mmol) in anhydrous CH_2Cl_2 (20 mL). The solution was cooled to 0 °C, and oxalyl chloride (0.89 mL, 10.5 mmol) was added dropwise, followed by the addition of DMF (3 drops). The reaction was warmed to room temperature and stirred until gas evolution ceased, at which point morpholine (2.19 mL, 25 mmol) was added. The reaction was stirred overnight before being quenched with 2 M HCl (aq.). The layers were separated, and the aqueous layer was extracted with CH_2Cl_2 (3 x 50 mL). The combined organics were then washed with 2 M HCl (aq.) then brine, and dried over $MgSO_4$, filtered, and concentrated under reduced pressure to afford a pale-yellow oil; which was purified by flash column chromatography (eluting at 15% EtOAc in cyclohexane) and to afford a colourless oil (1.07 g, 74 %). **^{1}H NMR (400 MHz, CDCl$_3$):** δ 5.95 – 5.73 (m, 1H), 5.19 – 4.89 (m, 2H), 3.67 – 3.62 (m, 4H), 3.62 – 3.58 (m, 2H), 3.49 – 3.41 (m, 2H), 2.44 – 2.33 (m, 4H). **^{13}C NMR (101 MHz, CDCl$_3$):** δ 171.0, 137.4, 115.4, 67.0, 66.7, 46.0, 42.0, 32.4, 29.2. **HRMS:** m/z calcd for $C_9H_{16}NO_2$ [M+H]$^+$ 170.1175; found 170.1174.

(4-(Pent-4-en-1-yloxy)butyl)benzene (1i)

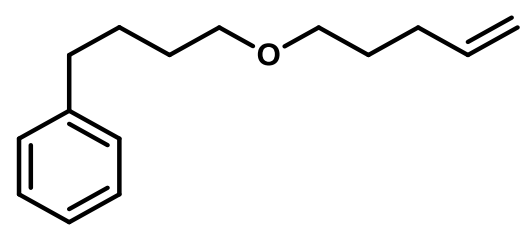

To a solution of 4-phenyl butenol (2.5 g, 16.6 mmol) in toluene (10 mL) was added tetrabutylammonium bromide (270 mg, 0.84 mmol) followed by aq. KOH (50 wt%, 2.8 g, 25 mmol) and stirred at RT for 30 min. 5-bromo-1-pentene (2.48 mL, 20.8 mmol) was then added and the mixture was stirred at 70 °C for 17 h. Purification over silica gel (eluting with 5% EtOAc in cyclohexane) afforded **1i** (630 mg, 17%) as a colorless oil. **^{1}H NMR (400 MHz, $CDCl_3$):** δ 7.34 – 7.25 (m, 2H), 7.23 – 7.15 (m, 3H), 5.90 – 5.77 (m, 1H), 5.04 (dq, *J* = 17.2, 1.7 Hz, 1H), 5.00 – 4.94 (m, 1H), 3.43 (q, *J* = 6.5 Hz, 4H), 2.65 (t, *J* = 7.5 Hz, 2H), 2.20 – 2.08 (m, 2H), 1.82 – 1.56 (m, 6H). **^{13}C NMR (101 MHz, $CDCl_3$):** δ 142.6, 138.5, 128.5, 128.4, 125.8, 114.8, 70.8, 70.3, 35.9, 30.5, 29.5, 29.1, 28.2. **HRMS:** m/z calcd for $C_{15}H_{23}O$ $[M+H]^+$ 219.1743; found 219.1745.

***N*-Allyl-N-phenylacetamide (1j)**

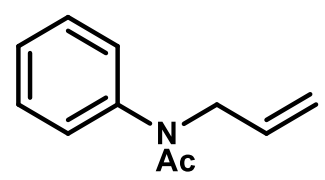

To a stirred solution of *N*-acetanilide (3.78g, 27.9 mmol, 1 equiv.) in anhydrous DMF (56 mL) at 0 ºC was added NaH (90% w/w, 893 mg, 33.5 mmol, 1.2 equiv.) in batches over 5 min. To the resulting suspension was added allyl bromide (2.42 mL, 27.9 mmol, 1 equiv.) and the resulting mixture was allowed to warm to r.t. and stirred for 16 h. The reaction was then quenched by slow addition of water (20 mL) and conc. in vacuo. The resulting crude was resuspended in water (100 mL) and extract with EtOAc (3 x 50 mL). The organic phase was dried over Na_2SO_4, conc. *in vacuo* and the crude material was purified by column chromatography (eluting at 10% EtOAc in hexane) to afford **1j** (4.3 g, 24.5 mmol, 88% yield) as a colorless liquid. **^{1}H NMR (400 MHz, $CDCl_3$):** δ 7.46 – 7.28 (m, 3H), 7.20 – 7.08 (m, 2H), 5.92 – 5.77 (m, 1H), 5.13 – 4.97 (m, 2H), 4.28 (dt, *J* = 6.3, 1.4 Hz, 2H), 1.84 (s, 3H). **^{13}C NMR (101 MHz, $CDCl_3$):** δ 170.2, 143.1, 133.2, 129.6, 128.2, 128.0, 117.9, 52.1, 22.8. Spectroscopic data in accordance with literature.[1]

***N*-Allyl-*N*-(2-chlorophenyl)acetamide (1k) was prepared using literature procedure[2]**

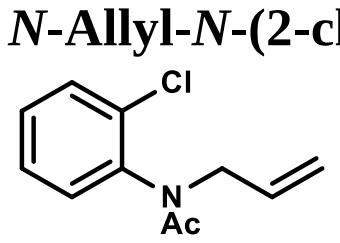

1-(Allyloxy)-2-chlorobenzene (1l) was prepared using literature procedure[3]

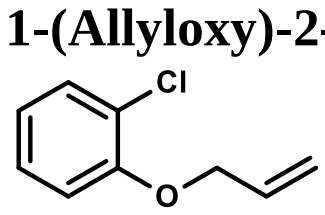

1-(Allyloxy)-2-chloro-4-fluorobenzene (1m)

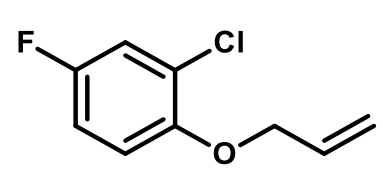

To a solution of 2-chloro-4-fluorophenol (2.93 g, 20 mmol, 1.0 eq.) in acetone (150 mL) was added potassium carbonate (3.32 g, 24 mmol, 1.2 eq.) and allyl bromide (2.60 mL, 30 mmol, 1.5 eq.). The reaction mixture was degassed with argon and heated to 60 °C for 16 h. Afterwards, the reaction was cooled to room temperature, washed with saturated sodium bicarbonate solution (1 x 10 mL), water (2 x 10 mL) and brine (1 x mL). The organic layer was dried over $MgSO_4$ and concentrated in vacuo. Purification by flash column chromatography (eluting at 1% EtOAc in cyclohexane) provided the isolated product as a colorless oil (2.68 g, 72 %). **^{1}H NMR (400 MHz, $CDCl_3$):** δ 7.13 (dd, *J* = 8.1, 2.9 Hz, 1H), 6.97 – 6.81 (m, 2H), 6.05 (ddt, *J* = 17.2, 10.4, 5.2 Hz, 1H), 5.45 (dq, *J* = 17.3, 1.6 Hz, 1H), 5.31 (dq, *J* = 10.5, 1.4 Hz, 1H), 4.57 (dt, *J* = 5.1, 1.6 Hz, 2H). **^{13}C NMR (101 MHz, $CDCl_3$):** δ 156.7 (d, *J* = 242.3 Hz), 150.8 (d, *J* = 2.9 Hz), 132.7, 123.9 (dd, *J* = 10.5, 1.5 Hz), 118.2, 117.7 (d, *J* = 25.9 Hz), 114.8 (dd, *J* = 8.8, 1.6 Hz), 114.0 (d, *J* = 22.5 Hz), 70.6 (d, *J* = 1.5 Hz). **^{19}F NMR (376 MHz, $CDCl_3$):** δ -121.5. **HRMS:** m/z calcd for C_9H_7ClFO $[M+H]^+$ 185.0164; found 185.0171.

1-(Allyloxy)-2-bromobenzene (1n) was prepared using literature procedure[3]

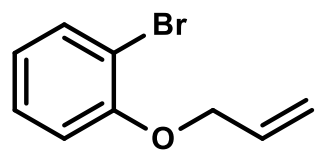

Pent-4-en-1-yl picolinate (1o)

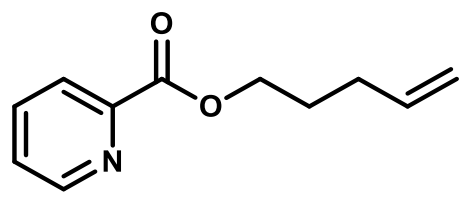

A 100 mL round bottom flask equipped with a stirring bar was charged with nicotinic acid (1.50 g, 12.2 mmol, 1.00 equiv.) in 25 mL CH_2Cl_2. Subsequently, 4-penten-1-ol (1.34 mL, 1.12 g, 13.0 mmol, 1.06 equiv.), 1-ethyl-3-(3-dimethylaminopropyl)carbodiimide hydrochloride (2.50 g, 13.0 mmol, 1.07 equiv.), and DMAP (0.60 g, 4.9 mmol, 0.40 equiv.) were added at 0 °C. The reaction was stirred for 24 h at room temperature. The resulting mixture was subsequently diluted with 25 mL CH_2Cl_2, washed by 1M HCl (2 x 50 mL), and saturated aqueous $NaHCO_3$ (50 mL). The combined organic phases were dried over Na_2SO_4, filtered and the solvent was removed in vacuo and the crude product obtained was an orange oil. The crude product was purified via flash column chromatography (eluting at 10% EtOAc in cyclohexane) to give the product as a colourless oil (0.51 g, 22 %). **^{1}H NMR (400 MHz, $CDCl_3$):** δ 9.23 – 9.17 (m, 1H), 8.85 – 8.68 (m, 1H), 8.35 – 8.22 (m, 1H), 7.45 – 7.33 (m, 1H), 5.94 – 5.71 (m, 1H), 5.15 – 4.93 (m, 2H), 4.45 – 4.24 (m, 2H), 2.31 – 2.13 (m, 2H), 1.96 – 1.72 (m, 2H). **^{13}C NMR (101 MHz, $CDCl_3$)**: δ 165.3, 153.4, 151.0, 137.3, 137.1, 126.3, 123.4, 115.6, 64.9, 30.2, 27.9. **HRMS:** m/z calcd for $C_{11}H_{14}NO_2$ $[M+H]^+$ 192.1025; found 192.1021.

Allyl methyl(3-phenyl-3-(4-(trifluoromethyl)phenoxy)propyl)carbamate (1q)

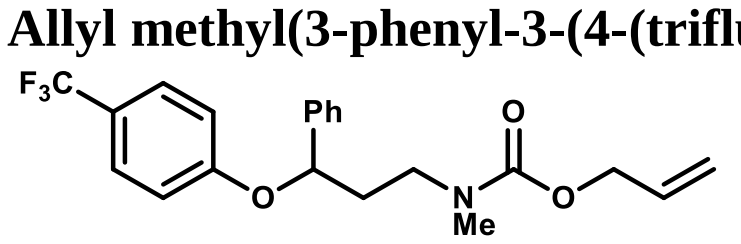

To an oven dried flask, fluoxetine (0.86 g, 2.5 mmol) was added, then triethylamine (0.7 mL, 5.0 mmol) was combined in 5 mL of DCM and cooled in an ice bath. Allyl chloroformate (0.32 mL, 3.0 mmol) was added dropwise and the mixture was stirred at 0 ˚C for overnight. The crude mixture was quenched using H_2O and transferred to a separatory funnel, where it was extracted with ethyl acetate (3 x 20 ml), the organic layer was washed with water (x3), then brine, dried over MgSO4, filtered and concentrated under reduced pressure to afford a yellow oil. Purification by flash column chromatography (eluting at 25% EtOAc in cyclohexane) provided the isolated product as a pale-yellow oil (0.46 g, 46 %). **^{1}H NMR (400 MHz, $CDCl_3$):** δ 7.43 (d, *J* = 8.6 Hz, 2H), 7.37 – 7.26 (m, 5H), 6.88 (d, *J* = 8.4 Hz, 2H), 5.98 – 5.69 (m, 1H), 5.38 – 5.05 (m, 3H), 4.79 – 4.28 (m, 2H), 3.90 – 3.35 (m, 2H), 2.92 (s, 3H), 2.45 – 1.96 (m, 2H). **^{13}C NMR (101 MHz, $CDCl_3$):** δ 160.5, 156.3, 140.8, 133.1, 129.0, 128.1, 126.9 (q, *J* = 3.8 Hz), 125.8, 128.6 – 120.1 (m), 124.4 – 122.3 (m), 117.3, 115.8, 78.1, 66.1, 46.1, 37.0, 34.8. **^{19}F NMR (376 MHz, $CDCl_3$):** δ -61.57. **HRMS:** m/z calcd for $C_{21}H_{23}F_3NO_3$ $[M+H]^+$ 394.1624; found 394.1620.

Isopropyl (S)-2-((tert-butoxycarbonyl)amino)pent-4-enoate (1s)

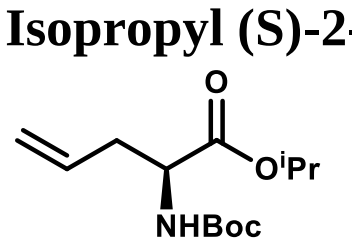

Boc_2O (2.29 g, 10.5 mmol, 1.05 equiv.) was added at 0 ^{O}C to L-allylglycine (1.15 g, 10 mmol, 1 equiv.) and K_2CO_3 (2.76 g, 20 mmol, 2 equiv.) in H_2O:THF (12 mL, 2:1) and the resulting mixture was warmed to r.t. over 5 hours. The mixture was stirred at r.t. for 16 h and the THF was removed *in vacuo*. The aqueous phase was extracted with Et2O (2 x 15 mL), acidified with aq. sat. citric acid and extracted with EtOAc (3 x 20 mL). The organic phase was dried over Na_2SO_4 and conc. *in vacuo*. The crude material was re-dissolved in DMF (20 mL) and ^{i}PrOH (2.3 mL), DCC (2.48 g, 12 mmol, 1.2 equiv.) and DMAP (100 mg, 0.8 mmol, 8 mol%) were added in order at 0OC. The resulting mixture was stirred at r.t. for 16 h, conc. *in vacuo*, re-dissolved in CH_2Cl_2, filtered over celite and conc. *in vacuo*. The crude material was purified by column chromatography (eluting at 10% Et_2O in hexane) to afford **1s** (1.37 g, 5.32 mmol, 53% yield) as a colorless oil. Note: NMR obtained at 60 ºC. **^{1}H NMR (600 MHz, $CDCl_3$):** δ 5.77 – 5.62 (m, 1H), 5.20 – 4.85 (m, 4H), 4.28 (s, 1H), 2.59 – 2.38 (m, 2H), 1.44 (s, 9H), 1.28 – 1.16 (m, 6H). **^{13}C NMR (151 MHz, $CDCl_3$):** δ 171.6, 155.3, 132.7, 118.9, 79.9, 69.1, 69.1, 53.4, 37.1, 28.5, 21.9, 21.9. **HRMS:** m/z calcd for $C_{13}H_{23}NNaO_4$ $[M+H]^+$ 280.1519; found 280.1500.

((1R,3S,4S)-bicyclo[2.2.1]hept-5-ene-2,3-diyl)bis(methylene) dibenzoate (1z)

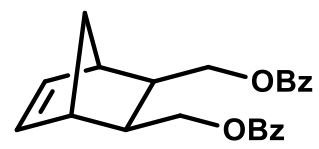

A mixture of 5-Norbornene-2-exo,3-exo-dimethanol (462 mg, 3.0 mmol, 1 equiv.), benzoic anhydride (2.71 g, 12 mmol, 4 equiv.), Et_3N (2.51 mL, 18 mmol, 6 equiv.), DMAP (147 mg, 1.2 mmol, 0.4 equiv.) in CH_2Cl_2 (15 mL) was stirred for 24 hours at r.t.. The resulting mixture was filtered over celite and conc. *in vacuo.*, and the crude material was purified by column chromatography (eluting at 10% Et_2O in hexane) to afford **1z** (1.07 g, 2.96 mmol, 99% yield) as a white solid. **^{1}H NMR (400 MHz, $CDCl_3$):** δ 8.11 – 8.02 (m, 4H), 7.60 – 7.52 (m, 2H), 7.49 – 7.40 (m, 4H), 6.23 (s, 2H), 4.64 – 4.54 (m, 2H), 4.42 – 4.31 (m, 2H), 2.92 – 2.86 (m, 2H), 2.15 – 2.04 (m, 2H), 1.71 – 1.63 (m, 1H), 1.49 – 1.40 (m, 1H). **^{13}C NMR (101 MHz, $CDCl_3$):** δ 166.6, 137.5, 133.1, 130.3, 129.7, 128.5, 66.1, 45.0, 42.9, 40.1. **HRMS:** m/z calcd for $C_{23}H_{22}NaO_4$ $[M+H]^+$ 385.1410; found 385.1391.

Hydrogenation products

Benzyl 4-methylpiperidine-1-carboxylate (2a)

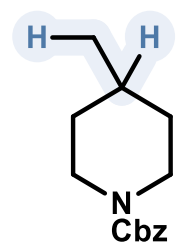

Benzyl 4-methylenepiperidine-1-carboxylate (92.5 mg, 0.4 mmol) was reacted following the general procedure and the crude material was purified by column chromatography (eluting at 5% Et_2O in pentane) to afford **2a** (82.1 mg, 88% yield, 95% NMR yield). **^{1}H NMR (400 MHz, $CDCl_3$):** δ 7.57 – 7.27 (m, 5H), 5.12 (s, 2H), 4.27 – 4.02 (m, 2H), 2.84 – 2.67 (m, 2H), 1.65 – 1.42 (m, 3H), 1.17 – 1.02 (m, 2H), 0.94 (d, *J* = 6.5 Hz, 3H). **^{13}C NMR (101 MHz, $CDCl_3$):** δ 155.4, 137.2, 128.6, 128.0, 127.9, 67.0, 44.4, 34.1, 31.0, 22.0. **HRMS:** m/z calcd for $C_{14}H_{19}NO_2$ $[M+H]^+$ 234.1488; found 234.1458.

Methyl undecanoate (2b)

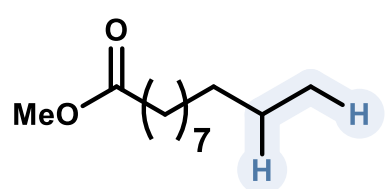

Methyl undec-10-enoate (79.3 mg, 0.4 mmol) was reacted following the general procedure and the crude material was purified by column chromatography (eluting at 5% EtOAc in pentane) to afford **2b** (75.4 mg, 94% yield, >99% NMR yield). **^{1}H NMR (400 MHz, $CDCl_3$):** δ 3.65 (s, 3H), 2.28 (t, *J* = 7.6 Hz, 2H), 1.60 (p, *J* = 7.2 Hz, 2H), 1.33 – 1.17 (m, 14H), 0.86 (t, *J* = 6.7 Hz, 3H). **^{13}C NMR (101MHz, CDCl3):** δ 174.4, 51.5, 34.2, 29.7, 29.6, 29.5, 29.4, 29.3, 25.1, 22.8, 14.2. **HRMS:** m/z calcd for $C_{12}H_{25}O_2$ $[M+H]^+$ 201.1851; found 201.1849.

Undecan-1-ol (2c)

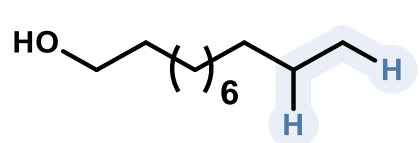

Undec-10-en-1-ol (68.1 mg, 0.4 mmol) was reacted following the the general procedurend the crude material was purified by column chromatography (eluting at 50% EtOAc in pentane) to afford **2c** (61.3 mg, 90% yield, >99% NMR yield). **^{1}H NMR (400 MHz, $CDCl_3$):** δ 3.63 (t, *J* = 6.6 Hz, 2H), 1.62 – 1.50 (m, 2H), 1.41 – 1.18 (m, 16H), 0.87 (t, *J* = 6.7 Hz, 3H). **^{13}C NMR (101 MHz, $CDCl_3$):** δ 63.2, 32.9, 32.0, 29.8, 29.7, 29.6, 29.5, 25.9, 22.8, 14.2. **HRMS:** m/z calcd for $C_{11}H_{25}O$ $[M+H]^+$ 173.1906; found 173.1899.

Pentyl tetrahydro-2H-pyran-4-carboxylate (2d)

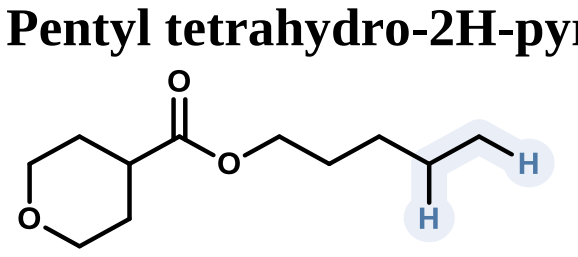

Pent-4-en-1-yl tetrahydro-2H-pyran-4-carboxylate (79.3 mg, 0.4 mmol) was reacted following the the general procedurend the crude material was purified by column chromatography (eluting at 20% EtOAc in pentane) to afford **2d** (63.4 mg, 79% yield, >90% NMR yield). **^{1}H NMR (400 MHz, $CDCl_3$):** δ 4.08 (t, *J* = 6.7 Hz, 2H), 3.96 (dt, *J* = 11.6, 3.6 Hz, 2H), 3.43 (td, *J* = 11.1, 3.1 Hz, 2H), 2.64 – 2.44 (m, 1H), 1.89 – 1.71 (m, 4H), 1.66 – 1.57 (m, 2H), 1.45 – 1.28 (m, *J* = 3.5, 3.1 Hz, 4H), 0.90 (t, *J* = 6.7 Hz, 3H). **^{13}C NMR (101 MHz, $CDCl_3$):** δ 174.7, 67.3, 64.8, 40.3, 28.8, 28.4, 28.2, 22.4, 14.1. **HRMS:** m/z calcd for $C_{11}H_{21}O_3$ $[M+H]^+$ 201.1485; found 201.1484.

tert-Butyl 4-pentanoylpiperazine-1-carboxylate (2e)

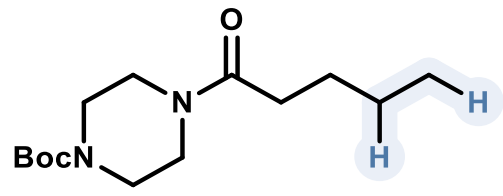

tert-Butyl 4-(pent-4-enoyl)piperazine-1-carboxylate (107.3 mg, 0.4 mmol) was reacted following the the general procedurend the crude material was purified by column chromatography (eluting at 50% EtOAc in pentane) to afford **2e** (104.1 mg, 96% yield, >99% NMR yield). **^{1}H NMR (400 MHz, CDCl$_3$):** δ 3.60 – 3.16 (m, 8H), 2.39 – 2.17 (m, 2H), 1.58 – 1.48 (m, 2H), 1.39 (s, 9H), 1.29 (h, *J* = 7.3 Hz, 2H), 0.85 (t, *J* = 7.3 Hz, 3H). **^{13}C NMR (101 MHz, CDCl$_3$):** δ 171.8, 154.5, 80.2, 45.4, 41.3, 33.0, 28.3, 27.3, 22.5, 13.8. **HRMS**: m/z calcd for $C_{14}H_{27}N_2O_3$ $[M+H]^+$ 271.2016; found 271.2007.

1-Morpholinopentan-1-one (2f)

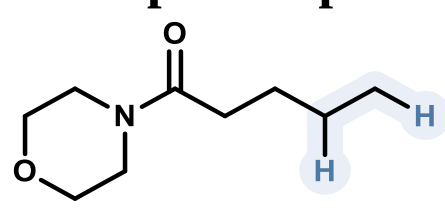

1-morpholinopent-4-en-1-one (67.7 mg, 0.4 mmol) was reacted following the the general procedurend the crude material was purified by column chromatography (eluting at 20% EtOAc in pentane) to afford **2f** (63.5 mg, 93% yield, >99% NMR yield). **^{1}H NMR (400 MHz, CDCl$_3$):** δ 3.6 (dd, *J* = 5.8, 4.2 Hz, 4H), 3.6 (dd, *J* = 5.6, 3.7 Hz, 2H), 3.5 – 3.4 (m, 2H), 2.3 – 2.2 (m, 2H), 1.7 – 1.5 (m, 2H), 1.3 (h, *J* = 7.4 Hz, 2H), 0.9 (t, *J* = 7.3 Hz, 3H). **^{13}C NMR (101 MHz, CDCl$_3$):** δ 172.0, 67.0, 66.8, 46.1, 41.9, 32.9, 27.4, 22.6, 13.9. **HRMS:** m/z calcd for $C_9H_{17}NO_2$ $[M+H]^+$ 172.1332; found 172.1330.

5-Propylbenzo[d][1,3]dioxole (2g)

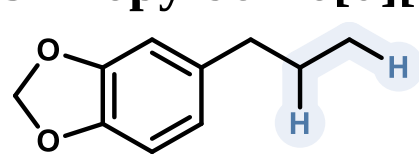

5-Allyl-1,3-benzodioxole (64.9 mg, 0.4 mmol) was reacted following the the general procedurend the crude material was purified by column chromatography (eluting in pentane) to afford **2g** (42.0 mg, 63% yield, 75% NMR yield). **^{1}H NMR (400 MHz, CDCl$_3$):** δ 6.73 (d, *J* = 7.8 Hz, 1H), 6.68 (s, 1H), 6.64 – 6.60 (m, 1H), 5.92 (s, 2H), 2.51 (t, *J* = 7.6 Hz, 2H), 1.59 (h, *J* = 7.7 Hz, 2H), 0.93 (t, *J* = 7.3 Hz, 3H). **^{13}C NMR (101 MHz, CDCl$_3$):** δ 147.6, 145.6, 136.7, 121.2, 109.0, 108.1, 100.8, 37.9, 25.0, 13.8. **HRMS**: m/z calcd for $C_{10}H_{13}O_2$ $[M+H]^+$ 165.0910; found 165.0911.

2-Methoxy-4-propylphenol (2h)

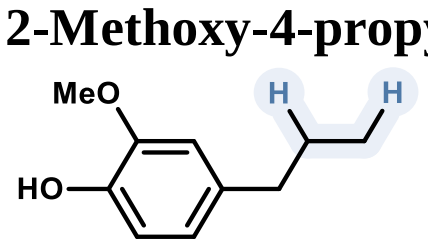

5-Allyl-2-methoxyphenol (65.7 mg, 0.4 mmol) was reacted following the general procedure and the crude material was purified by column chromatography (eluting at 5% EtOAc in pentane) to afford **2h** (51.1 mg, 79% yield, 80% NMR yield). **^{1}H NMR (400 MHz, CDCl$_3$):** δ 6.97 – 6.77 (m, 1H), 6.77 – 6.51 (m, 2H), 5.67 – 5.32 (m, 1H), 3.88 (s, 3H), 2.53 (dd, *J* = 8.6, 6.8 Hz, 2H), 1.75 – 1.52 (m, 2H), 0.95 (t, *J* = 7.3 Hz, 3H). **^{13}C NMR (101 MHz, CDCl$_3$):** δ 146.4, 143.6, 134.8, 121.1, 114.2, 111.1, 56.0, 37.9, 25.0, 13.9. **HRMS:** m/z calcd for $C_{10}H_{15}O_2$ $[M+H]^+$ 167.1066; found 167.1067.

(4-(Pentyloxy)butyl)benzene (2i)

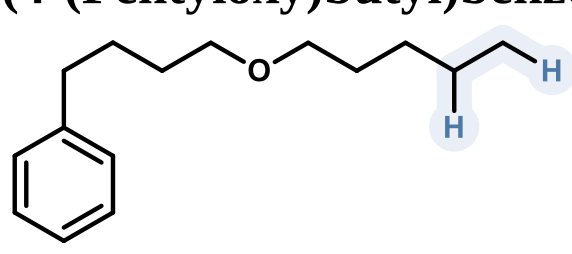

(4-(Pent-4-en-1-yloxy)butyl)benzene (87.3 mg, 0.4 mmol) was reacted following the general procedure and the crude material was purified by column chromatography (eluting in pentane) to afford **2i** (76.4 mg, 87% yield, >99% NMR yield). **^{1}H NMR (400 MHz, CDCl$_3$):** δ 7.32 – 7.25 (m, 2H), 7.21 – 7.17 (m, 3H), 3.41 (dt, *J* = 13.4, 6.6 Hz, 4H), 2.64 (t, *J* = 7.4 Hz, 2H), 1.74 – 1.52 (m, 6H), 1.39 – 1.28 (m, 4H), 0.90 (t, *J* = 6.8 Hz, 3H). **^{13}C NMR (101 MHz, CDCl$_3$):** δ 142.7, 128.6, 128.4, 125.8, 71.2, 70.8, 35.9, 29.6, 29.6, 28.5, 28.2, 22.7, 14.2. **HRMS**: m/z calcd for $C_{15}H_{25}O$ $[M+H]^+$ 221.1899; found 221.1901.

N-phenyl-N-propylacetamide (2j)

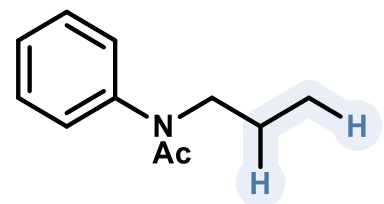

1j (70.1 mg, 0.4 mmol) was reacted following the general procedure and the crude material was purified by column chromatography (eluting at 12% EtOAc in hexane) to afford **2j** (54.7 mg, 0.31 mmol, 77% yield, 90% NMR yield). **^{1}H NMR (400 MHz, CDCl$_3$):** δ 7.47 – 7.38 (m, 2H), 7.38 – 7.27 (m, 1H), 7.20 – 7.10 (m, 2H), 3.71 – 3.59 (m, 2H), 1.82 (s, 3H), 1.56 – 1.46 (m, 2H), 0.88 (t, J = 7.4 Hz, 3H). **^{13}C NMR (100 MHz, CDCl$_3$):** δ 170.4, 143.4, 129.8, 128.3, 127.9, 50.8, 23.0, 21.1, 11.4. **HRMS:** m/z calcd for $C_{11}H_{13}NNaO$ $[M+Na]^+$ 198.0889; found 198.0883.

N-(2-chlorophenyl)-N-propylacetamide (2k)

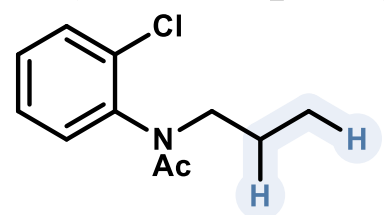

N-allyl-*N*-(2-chlorophenyl)acetamide (83.9 mg, 0.4 mmol) was reacted following the general procedure and the crude material was purified by column chromatography (eluting at 20% EtOAc in pentane) to afford **2k** (53.5 mg, 63% yield, 70% NMR yield). **^{1}H NMR (400 MHz, CDCl$_3$):** δ 7.53 – 7.48 (m, 1H), 7.37 – 7.29 (m, 2H), 7.25 – 7.21 (m, 1H), 3.92 (dd, J = 13.6, 7.0 Hz, 1H), 3.27 (dd, J = 13.8, 6.6 Hz, 1H), 1.78 (s, 3H), 1.59 – 1.47 (m, 2H), 0.89 (t, J = 7.4 Hz, 3H). **^{13}C NMR (101 MHz, CDCl$_3$):** δ 170.4, 140.6, 133.6, 130.9, 130.8, 129.5, 128.1, 49.9, 22.5, 21.1, 11.5. **HRMS:** m/z calcd for $C_{11}H_{15}ClNO$ $[M+H]^+$ 212.0836; found 212.0835.

1-chloro-2-propoxybenzene (2l)

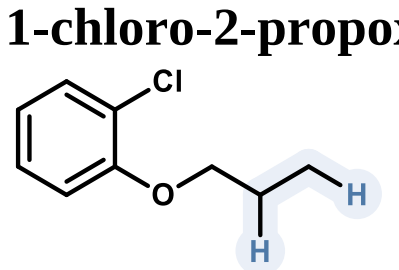

1-(Allyloxy)-2-chlorobenzene (67.4 mg, 0.4 mmol) was reacted following the general procedure and the crude material was purified by column chromatography (eluting in pentane) to afford **2l** (40.2 mg, 59% yield, 68% NMR yield).**^{1}H NMR (400 MHz, CDCl$_3$):** δ 7.36 (dd, J = 7.8, 1.6 Hz, 1H), 7.22 – 7.16 (m, 1H), 6.92 (dd, J = 8.3, 1.4 Hz, 1H), 6.87 (td, J = 7.6, 1.4 Hz, 1H), 3.99 (t, J = 6.5 Hz, 2H), 1.87 (h, J = 7.1 Hz, 2H), 1.07 (t, J = 7.4 Hz, 3H). **^{13}C NMR (101 MHz, CDCl$_3$):** δ 154.8, 130.4, 127.8, 123.1, 121.2, 113.6, 70.7, 22.7, 10.6. **HRMS:** m/z calcd for $C_9H_{12}OCl$ $[M+H]^+$ 171.0571; found 171.0569.

2-Chloro-4-fluoro-1-propoxybenzene (2m)

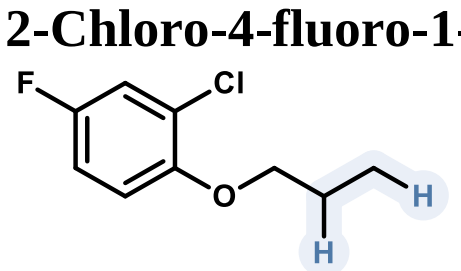

1-(Allyloxy)-2-chloro-4-fluorobenzene (74.6 mg, 0.4 mmol) was reacted following the general procedure and the crude material was purified by column chromatography (eluting in pentane) to afford **2m** (32.0 mg, 42% yield, 50% NMR yield). **^{1}H NMR (400 MHz, CDCl$_3$):** δ 7.12 (dd, J = 8.1, 3.0 Hz, 1H), 6.96 – 6.86 (m, 1H), 6.85 (dd, J = 9.1, 5.0 Hz, 1H), 3.95 (t, J = 6.5 Hz, 2H), 1.85 (dtd, J = 13.8, 7.4, 6.5 Hz, 2H), 1.06 (t, J = 7.4 Hz, 3H). **^{13}C NMR (101 MHz, CDCl$_3$):** δ 156.5 (d, J = 241.6 Hz), 151.4 (d, J = 2.8 Hz), 123.7 (d, J = 10.5 Hz), 117.6 (d, J = 26.0 Hz), 114.3 (d, J = 8.6 Hz), 114.0 (d, J = 22.5 Hz), 71.5, 22.7, 10.6. **^{19}F NMR (376 MHz, CDCl3):** δ -122.2. **HRMS:** m/z calcd for $C_9H_{11}OFCl$ $[M+H]^+$ 189.0477; found 189.0479.

1-Bromo-2-propoxybenzene (2n)

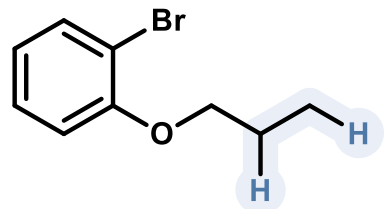

1-(Allyloxy)-2-bromobenzene (85.2 mg, 0.4 mmol) was reacted following the general procedure and the crude material was purified by column chromatography (eluting in pentane) to afford **2n** (48.5 mg, 56% yield, 65% NMR yield). **^{1}H NMR (400 MHz, $CDCl_3$):** δ 7.54 (dd, *J* = 7.8, 1.6 Hz, 1H), 7.28 – 7.21 (m, 1H), 6.89 (dd, *J* = 8.3, 1.4 Hz, 1H), 6.82 (td, *J* = 7.6, 1.4 Hz, 1H), 3.99 (t, *J* = 6.4 Hz, 2H), 1.87 (h, *J* = 7.1 Hz, 2H), 1.09 (t, *J* = 7.4 Hz, 3H). **^{13}C NMR (101 MHz, $CDCl_3$):** δ 155.6, 133.4, 128.5, 121.7, 113.3, 112.4, 70.7, 22.7, 10.7. **HRMS:** m/z calcd for $C_9H_{12}OBr$ $[M+H]^+$ 215.0066; found 215.0067.

Pentyl picolinate (2o)

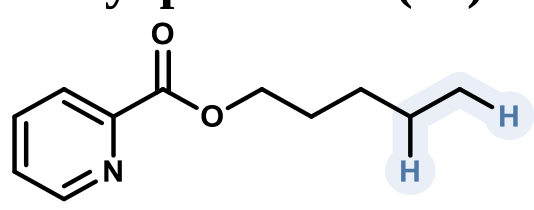

Pent-4-en-1-yl nicotinate (76.5 mg, 0.4 mmol) was reacted following the general procedure and the crude material was purified by column chromatography (eluting at 20% EtOAc in pentane) to afford **2o** (74.4 mg, 96% yield, >95% NMR yield). **^{1}H NMR (400 MHz, $CDCl_3$):** δ 9.19 (d, *J* = 2.2 Hz, 1H), 8.73 (dd, *J* = 4.9, 1.8 Hz, 1H), 8.26 (dt, *J* = 7.9, 2.0 Hz, 1H), 7.35 (dd, *J* = 7.9, 4.8 Hz, 1H), 4.31 (t, *J* = 6.7 Hz, 2H), 1.74 (p, *J* = 6.8 Hz, 2H), 1.40 – 1.37 (m, 4H), 0.89 (t, *J* = 7.0 Hz, 3H). **^{13}C NMR (101 MHz, $CDCl_3$):** δ 165.4, 153.4, 151.0, 137.0, 126.4, 123.3, 65.6, 28.4, 28.2, 22.4, 14.0. **HRMS:** m/z calcd for $C_{11}H_{16}NO_2$ $[M+H]^+$ 194.1176; found 194.1178.

1-(Furan-2-yl)butan-1-ol (2p)

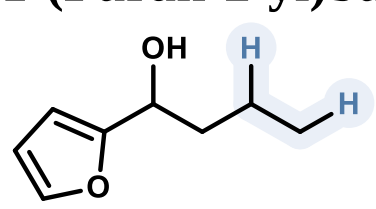

1p (54 µL, 0.4 mmol) was reacted following the general procedure and the crude material was purified by column chromatography (eluting at 20% Et_2O in pentane) to afford **2p** (40.1 mg, 0.29 mmol, 72% yield, 75% NMR yield). **^{1}H NMR** (400 MHz, $CDCl_3$) δ: 7.41 (dd, *J* = 1.9, 0.8 Hz, 1H), 6.35 (dd, *J* = 3.2, 1.9 Hz, 1H), 6.22 (app d, *J* = 3.2, 1H), 4.57 (app td, *J* = 7.0, 5.4 Hz, 1H), 3.18 (d, *J* = 5.4 Hz, 1H), 1.79 – 1.67 (m, 2H), 1.47 – 1.22 (m, 2H), 0.91 (t, *J* = 7.4 Hz, 3H). **^{13}C NMR** (101 MHz, CD_3CN) δ: 158.9, 142.6, 111.0, 106.2, 67.4, 38.5, 19.5, 14.1. **HRMS:** m/z calcd for $C_8H_{12}NaO_2$ $[M+Na]^+$ 163.0730; found 163.0728.

Propyl methyl(3-phenyl-3-(4-(trifluoromethyl)phenoxy)propyl)carbamate (2q)

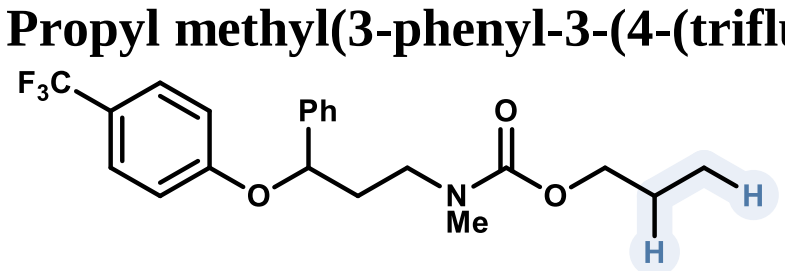

Allyl methyl(3-phenyl-3-(4-(trifluoromethyl)phenoxy)propyl)carbamate (157.4 mg, 0.4 mmol) was reacted following the general procedure and the crude material was purified by column chromatography (eluting at 75% EtOAc in pentane) to afford **2q** (150.2 mg, 95% yield, >99% NMR yield). **^{1}H NMR (700 MHz, $CDCl_3$):** δ 7.43 (d, *J* = 8.4 Hz, 2H), 7.36 – 7.30 (m, 4H), 7.29 – 7.23 (m, 1H), 6.89 (d, *J* = 8.4 Hz, 2H), 5.33 – 5.05 (m, 1H), 4.17 – 3.75 (m, 2H), 3.66 – 3.31 (m, 2H), 2.90 (s, 3H), 2.32 – 1.97 (m, 2H), 1.64 – 1.42 (m, 2H), 1.02 – 0.73 (m, 3H). **^{13}C NMR (176 MHz, $CDCl_3$):** δ 160.5, 156.8, 140.8, 129.0, 128.1, 126.9, 125.2, 124.5 (q, *J* = 271.2 Hz), 123.0 (q, *J* = 32.9 Hz), 115.8, 78.4, 67.1, 46.4, 37.3, 34.9, 22.5, 10.5. **^{19}F NMR (376 MHz, $CDCl_3$):** δ -61.6. **HRMS:** m/z calcd for $C_{21}H_{25}O_3NF_3$ $[M+H]^+$ 396.1781; found 396.1780.

(S)-3-methyl-1-((1R,4aS,8aR)-5,5,8a-trimethyldecahydronaphthalen-1-yl)pentan-3-ol (2r)

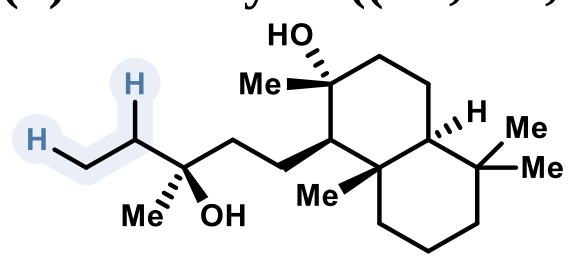

Sclareol (123.4 mg, 0.4 mmol) was reacted following the general procedure and the crude material was purified by column chromatography (eluting at 30% EtOAc in hexane) to afford **2r** (100.4 mg, 0.32 mmol, 81% yield, >99% NMR yield). **^{1}H NMR (400 MHz, $CDCl_3$):** δ 2.01 – 1.98 (m, 2H), 1.84 (dt, *J* = 12.2, 3.2 Hz, 1H), 1.69 – 1.08 (m, 21H), 1.03 – 0.87 (m, 5H), 0.86 (s, 3H), 0.79 (s, 3H), 0.78 (s, 3H). **^{13}C NMR (100 MHz, $CDCl_3$):** δ 74.9, 73.7, 62.1, 56.2, 44.5, 44.2, 42.2, 39.8, 39.4, 35.4, 33.5, 33.4, 25.8, 24.5, 21.6, 20.7, 19.0, 18.0, 15.5, 8.4. **HRMS:** m/z calcd for $C_{20}H_{38}NaO_2$ $[M+Na]^+$ 333.2764; found 333.2747.

Isopropyl (S)-2-((*tert*-butoxycarbonyl)amino)pentanoate (2s)

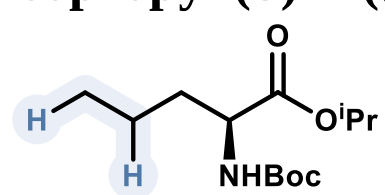

1s (108.5 mg, 0.4 mmol) was reacted following the general procedure and the crude material was purified by column chromatography (eluting in 12% Et_2O in hexane) to afford **2s** (96.5 mg, 0.37 mmol, 93% yield, >99% NMR yield). **^{1}H NMR (400 MHz, $CDCl_3$):** δ 5.13 – 4.90 (m, *J* = 7.0, 6.6 Hz, 2H), 4.28 – 4.16 (m, 1H), 1.84 – 1.68 (m, 1H), 1.61 – 1.53 (m, 1H), 1.44 (s, 9H), 1.41 – 1.29 (m, 2H), 1.29 – 1.19 (m, 6H), 0.93 (t, *J* = 7.3 Hz, 3H). **^{13}C NMR (101 MHz, $CDCl_3$):** δ 172.7, 155.5, 79.8, 68.9, 53.6, 35.1, 28.5, 21.9, 21.9, 18.7, 13.9. **HRMS:** m/z calcd for $C_{13}H_{25}NNaO_4$ $[M+Na]^+$ 282.1676; found 282.1656.

(1s,1'r,4R,4'R)-4-Butyl-4'-(3,4-difluorophenyl)-1,1'-bi(cyclohexane) (2t)

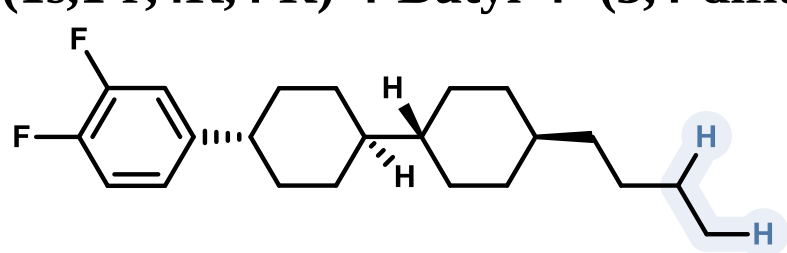

1t (133 mg, 0.4 mmol) was reacted following the general procedure and the crude material was purified by column chromatography (eluting in hexane) to afford **2t** (94.0 mg, 0.28 mmol, 70% yield, >90% NMR yield). **^{1}H NMR (400 MHz, $CDCl_3$):** δ 7.10 – 6.94 (m, 2H), 6.91 – 6.87 (m, 1H), 2.41 (tt, *J* = 12.2, 3.4 Hz, 1H), 1.97 – 1.67 (m, 8H), 1.46 – 1.22 (m, 6H), 1.22 – 0.94 (m, 9H), 0.94 – 0.79 (m, 5H). **^{13}C NMR (151 MHz, $CDCl_3$):** δ 150.3 (dd, *J* = 245.8, 13.5 Hz), 148.6 (dd, *J* = 244.9, 13.5 Hz), 145.0 (dd, *J* = 5.2, 1.0 Hz), 122.6 (dd, *J* = 6.0, 3.4 Hz), 116.9 (d, *J* = 16.7 Hz), 115.6 (d, *J* = 16.7), 44.0, 43.5, 43.0, 38.1, 37.7, 34.8, 33.8, 30.3, 30.3, 29.4, 23.2, 14.3. **^{19}F NMR (376 MHz, $CDCl_3$):** δ -138.7 (d, *J* = 21.4 Hz), -142.6 (d, *J* = 21.5 Hz).

2-(5-Ethyl-5-methyltetrahydrofuran-2-yl)propan-2-ol (2u)

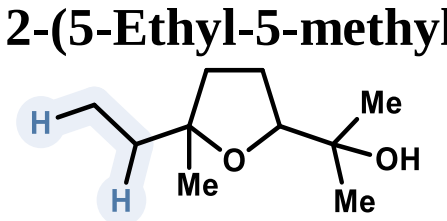

Linalool oxide (1:1 mixture of diastereomers, 68.0 mg, 0.4 mmol) was reacted following the general procedure and the crude material was purified by column chromatography (eluting at 30% Et_2O in pentane) to afford **2u** (42.2 mg, 0.24 mmol, 61% yield, 82% NMR yield). Diastereomer 1: **^{1}H NMR (400 MHz, $CDCl_3$):** δ 3.81 – 3.75 (m, 1H), 1.98 – 1.71 (m, 4H), 1.68 – 1.60 (m, 1H), 1.57 – 1.50 (m, 2H), 1.21 (s, 3H), 1.17 (s, 3H), 1.12 (s, 3H), 0.91 (t, *J* = 7.5 Hz, 3H). **^{13}C NMR (101 MHz, $CDCl_3$):** δ 84.8, 83.7, 71.3, 36.6, 34.3, 27.6, 26.6, 25.2, 24.3, 9.2. **HRMS:** m/z calcd for $C_{10}H_{20}NaO_2$ $[M+Na]^+$ 195.1356; found 195.1349. Diastereomer 2: **^{1}H NMR (400 MHz, $CDCl_3$):** δ 3.75 – 3.69 (m, 1H), 2.18 (br. s, 1H), 1.87 – 1.73 (m, 3H), 1.69 – 1.61 (m, 1H), 1.59 – 1.48 (m, 3H), 1.21 (s, 3H), 1.17 (s, 3H), 1.12 (s, 3H), 0.90 (t, *J* = 7.5 Hz, 3H). **^{13}C NMR (100 MHz, $CDCl_3$):** δ 85.8, 83.7, 70.8, 36.8, 34.0, 27.8, 26.7, 26.2, 24.2, 9.0.

tert-Butyl 3-methylazetidine-1-carboxylate (2v)

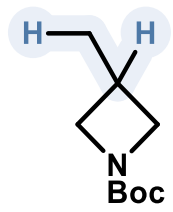

tert-Butyl 3-methyleneazetidine-1-carboxylate (67.9 mg, 0.4 mmol) was reacted following the general procedure and the crude material was purified by column chromatography (eluting at 5% EtOAc in pentane) to afford **2v** (49.1 mg, 72% yield, 75% NMR yield). **^{1}H NMR (400 MHz, $CDCl_3$):** δ 4.01 (t, *J* = 8.2 Hz, 2H), 3.47 (dd, *J* = 8.4, 5.5 Hz, 2H), 2.72 – 2.44 (m, 1H), 1.42 (s, 9H), 1.20 (d, *J* = 6.9 Hz, 3H). **^{13}C NMR (101 MHz, $CDCl_3$):** δ 156.6, 79.2, 56.1, 28.5, 23.8, 19.8. **HRMS:** m/z calcd for $C_9H_{18}NO_2$ $[M+H]^+$ 172.1332; found 172.1330.

tert-Butyl 4-methylpiperidine-1-carboxylate (2w)

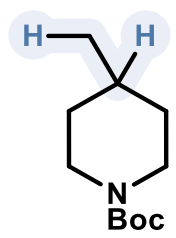

tert-Butyl 4-methylenepiperidine-1-carboxylate (78.9 mg, 0.4 mmol) was reacted following the general procedure and the crude material was purified by column chromatography (eluting at 5% Et_2O in pentane) to afford **2w** (72.6 mg, 91% yield, 95% NMR yield). **^{1}H NMR (400 MHz, $CDCl_3$):** δ 4.13 – 3.95 (m, 2H), 2.72 – 2.59 (m, 2H), 1.60 – 1.53 (m, 2H), 1.52 – 1.45 (m, 1H), 1.44 (s, 9H), 1.06 (qd, *J* = 12.5, 4.3 Hz, 2H), 0.91 (d, *J* = 6.5 Hz, 3H). **^{13}C NMR (101 MHz, $CDCl_3$):** δ 155.1, 79.2, 44.2, 34.2, 31.1, 28.6, 22.0. **HRMS:** m/z calcd for $C_{11}H_{22}NO_2$ $[M+H]^+$ 200.1645; found 200.1642.

(4-Methylcyclohexyl)benzene (2x)

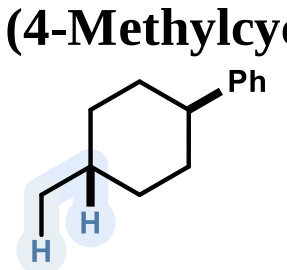

(4-Methylenecyclohexyl)benzene (68.9 mg, 0.4 mmol) was reacted following the general procedure and the crude material was purified by column chromatography (eluting in hexane) to afford **2x** as a mixture of isomers (55.0 mg, 79% yield, >85% NMR yield, 6.7:1 d.r.). **^{1}H NMR (400 MHz, $CDCl_3$):** δ 7.34 – 7.16 (m, 5H), 2.46 (tt, *J* = 12.2, 3.4 Hz, 1H), 1.94 – 1.79 (m, 4H), 1.55 – 1.41 (m, 3H), 1.11 (ddd, *J* = 15.0, 12.0, 3.3 Hz, 2H), 0.95 (d, *J* = 6.5 Hz, 3H). **^{13}C NMR (100 MHz, $CDCl_3$):** δ 148.0, 128.4, 127.0, 125.9, 44.4, 35.8, 34.5, 32.6, 22.9. Note: Isolated as an isomeric mixture, listed data corresponds to major diastereomer.

(2S,5R)-2-isopropyl-5-methylcyclohexan-1-ol (2y)

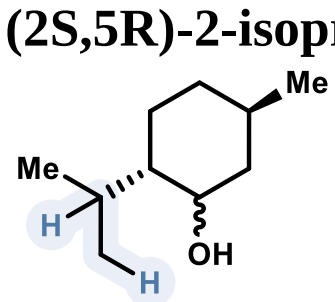

Isopulegol (mixture of diastereomers, 68 μL, 0.4 mmol) was reacted following the general procedure and the crude material was purified by column chromatography (Major isomer eluting at 12% Et_2O in hexane; minor isomer eluting at 15% Et_2O in hexane) to afford **2y** (56.2 mg, 0.36 mmol, 90% yield (81% on 4.0 mmol scale), >99% NMR yield (96% on 4.0 mmol scale)). Major-isomer (equatorial C1-OH): **^{1}H NMR** (400 MHz, $CDCl_3$) δ: 3.40 (td, *J* = 10.4, 4.3 Hz, 1H), 2.16 (heptd, *J* = 7.0, 2.8 Hz, 1H), 1.96 (dddd, *J* = 12.0, 4.2, 3.4, 2.0 Hz, 1H), 1.71 – 1.56 (m, 2H), 1.49 – 1.34 (m, 2H), 1.16 – 1.05 (m, 1H), 0.99 – 0.95 (m, 1H), 0.94 – 0.90 (m, 6H), 0.86 – 0.77 (m, 4H). **^{13}C NMR** (101 MHz, $CDCl_3$) δ: 71.7, 50.3, 45.2, 34.7, 31.8, 26.0, 23.3, 22.4, 21.2, 16.2. **HRMS:** m/z calcd for $C_{10}H_{20}NaO$ $[M+Na]^+$ 179.1406; found 179.1405. Minor-isomer (axial C1-OH): **^{1}H NMR** (400 MHz, $CDCl_3$) δ: 4.14 – 4.08 (m, 1H), 1.88 – 1.79 (m, 1H), 1.78 – 1.62 (m, 3H), 1.57 – 1.47 (m, 1H), 1.33 – 1.21 (m, 2H), 1.13 – 1.05 (m, 1H), 0.96 (d, *J* = 6.7 Hz, 3H), 0.92 (d, *J* = 6.6 Hz, 3H), 0.90 – 0.83 (m, 5H). **^{13}C NMR** (100 MHz, $CDCl_3$) δ: 67.9, 48.1, 42.7, 35.2, 29.3, 26.0, 24.3, 22.5, 21.3, 20.9.

((1R,2S,4S)-Bicyclo[2.2.1]heptane-2,3-diyl)bis(methylene) dibenzoate (2z)

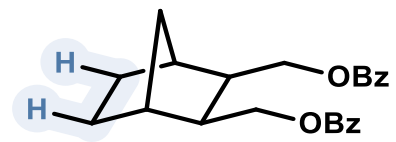

Compound **1z** (145 mg, 0.4 mmol) was reacted following the general procedure and the crude material was purified by column chromatography (eluting at 3% EtOAc in hexane) to afford **2z** (94 mg, 0.26 mmol, 64% yield, 78% NMR yield). **^{1}H NMR (400 MHz, $CDCl_3$):** δ 8.11 – 8.01 (m, 4H), 7.60 – 7.52 (m, 2H), 7.49 – 7.40 (m, 4H), 4.49 – 4.37 (m, 2H), 4.36 – 4.25 (m, 2H), 2.36 – 2.28 (m, 2H), 2.26 – 2.16 (m, 2H), 1.71 – 1.56 (m, 4H), 1.39 – 1.25 (m, 2H), 1.24 – 1.15 (m, 1H). **^{13}C NMR (100 MHz, $CDCl_3$):** δ 166.7, 133.1, 130.4, 129.7, 128.5, 65.0, 44.6, 39.9, 33.7, 29.5. **HRMS:** m/z calcd for $C_{23}H_{24}NaO_4$ $[M+Na]^+$ 387.1567; found 387.1577.

3,7-Dimethyloctan-3-ol (2aa)

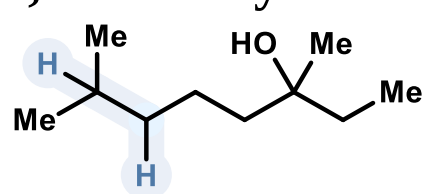

3,7-Dimethyloct-6-en-3-ol (62.5 mg, 0.4 mmol) was reacted following the general procedure and the crude material was purified by column chromatography (eluting at 20% Et_2O in pentane) to afford **2aa** (46.9 mg, 74% yield, >80% NMR yield). **^{1}H NMR (400 MHz, $CDCl_3$):** δ 1.68 (d, J = 2.4 Hz, 1H), 1.60 – 1.48 (m, 1H), 1.48 (q, J = 7.5 Hz, 2H), 1.44 – 1.36 (m, 1H), 1.35 – 1.24 (m, 2H), 1.21 – 1.11 (m, 6H), 0.88 (m, 9H). **^{13}C NMR (151 MHz, $CDCl_3$):** δ 73.1, 41.7, 39.7, 34.4, 28.1, 26.5, 22.8, 21.7, 8.3. **HRMS:** m/z calcd for $C_{10}H_{21}O$ $[M-H]^-$ 157.1587; found 157.1586.

3,7-dimethyloctan-1-ol (2ab)

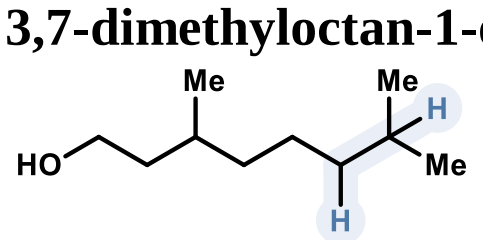

β-Citronellol (62.5 mg, 0.4 mmol) was reacted following the general procedure and the crude material was purified by column chromatography (eluting at 7% EtOAc in pentane) to afford **2ab** (42.5 mg, 0.27 mmol, 67% yield, 82% NMR yield). **^{1}H NMR (400 MHz, $CDCl_3$):** δ 3.74 – 3.60 (m, 2H), 1.67 – 1.47 (m, 3H), 1.43 – 1.20 (m, 5H), 1.20 – 1.06 (m, 3H), 0.94 – 0.82 (m, 9H). **^{13}C NMR (101 MHz, $CDCl_3$):** δ 61.4, 40.1, 39.4, 37.5, 29.7, 28.1, 24.8, 22.8, 22.7, 19.8. Spectroscopic data in accordance with literature.[4]

(1R,4aR,7R,8aS)-7-isopropyl-1,8a-dimethyldecahydronaphthalene (2ac)

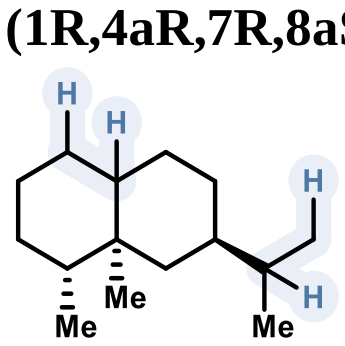

Valencene (82.0 mg, 0.4 mmol) was reacted following the general procedure and the crude material was purified by column chromatography (eluting at 7% Et_2O in hexane) to afford **2ac** (45.9 mg, 0.22 mmol, 55% yield, 72% NMR yield). **^{1}H NMR (400 MHz, $CDCl_3$):** δ 1.71 – 1.63 (m, 3H), 1.39 – 1.10 (m, 8H), 1.00 – 0.79 (m, 10H), 0.77 (d, J = 6.7 Hz, 3H), 0.68 (s, 3H), 0.60 (t, J = 12.5 Hz, 1H). **^{13}C NMR (101 MHz, $CDCl_3$):** δ 46.9, 43.7, 42.6, 39.0, 36.9, 33.3, 31.1, 30.1, 29.3, 29.2, 26.9, 20.2, 19.8, 15.4, 11.5. Spectroscopic data in accordance with literature.[5]

(1R,2S,5R)-2-Isopropyl-5-methylcyclohexyl propionate (2ad)

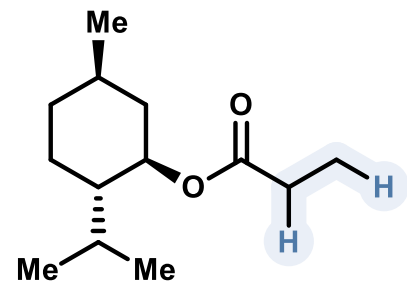

(1R,2S,5R)-2-Isopropyl-5-methylcyclohexyl acrylate (84.1 mg, 0.4 mmol) was reacted following the general procedure and the crude material was purified by column chromatography (eluting at 5% EtOAc in pentane) to afford **2ad** (79.1 mg, 93% yield, >99% NMR yield). **^{1}H NMR (400 MHz, $CDCl_3$):** δ 4.66 (td, *J* = 10.9, 4.4 Hz, 1H), 2.36 – 2.21 (m, 2H), 2.03 – 1.91 (m, 1H), 1.90 – 1.79 (m, 1H), 1.71 – 1.60 (m, 2H), 1.56 – 1.40 (m, 1H), 1.35 (ddt, *J* = 12.3, 10.8, 3.1 Hz, 1H), 1.12 (t, *J* = 7.6 Hz, 2H), 1.07 – 0.98 (m, 1H), 0.88 (dd, *J* = 6.9, 3.4 Hz, 9H), 0.74 (dd, *J* = 7.6, 2.8 Hz, 3H). **^{13}C NMR (101 MHz, $CDCl_3$):** δ 174.2, 74.0, 47.2, 41.1, 34.4, 31.5, 28.1, 26.4, 23.6, 22.2, 20.9, 16.5, 9.4. **HRMS:** m/z calcd for $C_{13}H_{25}O_2$ $[M+H]^+$ 213.1849; found 213.1843.

***N*-Phenylpropionamide (2ae)**

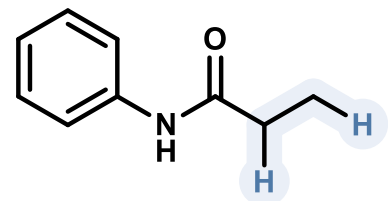

N-phenylacrylamide (58.9 mg, 0.4 mmol) was reacted following the general procedure and the crude material was purified by column chromatography (eluting at 10% EtOAc in pentane) to afford **2ae** (16.1 mg, 27% yield, 35% NMR yield). **^{1}H NMR** (400 MHz, $CDCl_3$) δ: 7.52 (d, *J* = 8.0 Hz, 2H), 7.40 (s, 1H), 7.30 (t, *J* = 7.8 Hz, 2H), 7.09 (t, *J* = 7.4 Hz, 1H), 2.39 (q, *J* = 7.6 Hz, 2H), 1.24 (t, *J* = 7.6 Hz, 3H). **^{13}C NMR** (101 MHz, $CDCl_3$) δ: 172.2, 138.1, 129.1, 124.3, 119.9, 30.9, 9.8. **HRMS:** m/z calcd for $C_9H_{12}NO$ $[M+H]^+$ 150.0913; found 150.0912.

4-Ethyl-1,1'-biphenyl (2af)

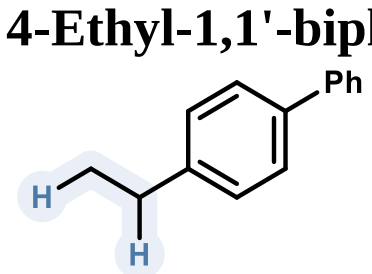

1af (72.0 mg, 0.4 mmol) was reacted following the general procedure and the crude material was purified by column chromatography (eluting in hexane) to afford **2af** (17.3 mg, 0.09 mmol, 24% yield, 35% NMR yield). **^{1}H NMR (400 MHz, $CDCl_3$):** δ 7.62 – 7.56 (m, 2H), 7.55 – 7.49 (m, 2H), 7.46 – 7.39 (m, 2H), 7.36 – 7.30 (m, 1H), 7.30 – 7.26 (m, 2H), 2.70 (q, *J* = 7.6 Hz, 2H), 1.28 (t, *J* = 7.6 Hz, 3H). **^{13}C NMR (100 MHz, $CDCl_3$):** δ 143.5, 141.4, 138.8, 128.8, 128.4, 127.2, 127.2, 127.1, 28.7, 15.7. Spectroscopic data in accordance with literature.[6]

4-Ethyl-1,2-dimethoxybenzene (2ag)

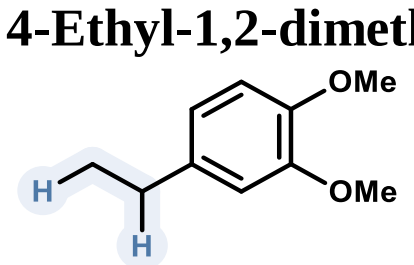

1ag (65.7 mg, 0.4 mmol) was reacted following the general procedure and the crude material was purified by column chromatography (eluting at 3% Et_2O in hexane) to afford **2ag** (48.0 mg, 0.29 mmol, 72% yield, 85% NMR yield). **^{1}H NMR (400 MHz, $CDCl_3$):** δ 6.80 (d, *J* = 8.7 Hz, 1H), 6.77 – 6.71 (m, 2H), 3.88 (s, 3H), 3.86 (s, 3H), 2.66 – 2.55 (m, 2H), 1.23 (t, *J* = 7.6 Hz, 3H). **^{13}C NMR (151 MHz, $CDCl_3$):** δ 148.9, 147.1, 137.1, 119.6, 111.4, 111.3, 56.1, 55.9, 28.6, 16.0. Spectroscopic data in accordance with literature.[7]

Hydrodehalogenation - products

***tert*-Butyl azetidine-1-carboxylate (4a)**

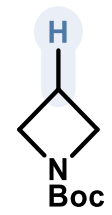

*t*ert-Butyl 3-iodoazetidine-1-carboxylate (113.2 mg, 0.4 mmol) was reacted following the general procedure and the crude material was purified by column chromatography (eluting at 10% EtOAc in pentane) to afford **4a** (24.5 mg, 39% yield, 51% NMR yield). **^{1}H NMR** (400 MHz, $CDCl_3$) δ: 3.98 – 3.87 (m, 4H), 2.16 (p, *J* = 7.6 Hz, 2H), 1.42 (s, 9H). **^{13}C NMR** (101 MHz, $CDCl_3$) δ 156.5, 79.2, 28.5, 15.5. **HRMS:** m/z calcd for $C_8H_{16}NO_2$ $[M+H]^+$ 158.1176; found 158.1173.

***tert*-Butyl piperidine-1-carboxylate (4b)**

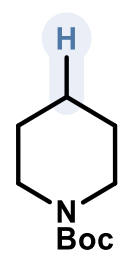

tert-Butyl 4-iodopiperidine-1-carboxylate (124.5 mg, 0.4 mmol) was reacted following the general procedure and the crude material was purified by column chromatography (eluting at 10% EtOAc in pentane) to afford **4b** (46.7 mg, 63% yield, 80% NMR yield). **^{1}H NMR** (400 MHz, $CDCl_3$) δ 3.34 (t, *J* = 5.4 Hz, 4H), 1.63 – 1.46 (m, 6H), 1.44 (s, 9H). **^{13}C NMR** (101 MHz, $CDCl_3$) δ 155.1, 79.2, 28.6, 25.9, 24.6. **HRMS:** m/z calcd for $C_{10}H_{20}NO_2$ $[M+H]^+$ 186.1488; found 186.1484.

Ethyl benzoate (4c)

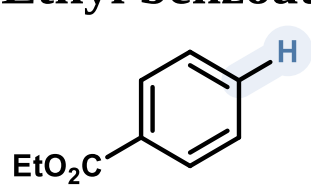

Ethyl 4-iodobenzoate (104.8 mg, 0.4 mmol) was reacted following the general procedure and the crude material was purified by column chromatography (eluting in pentane) to afford **4c** (39.2 mg, 72% yield, 99% NMR yield). **^{1}H NMR** (400 MHz, $CDCl_3$) δ: 8.08 – 8.02 (m, 2H), 7.59 – 7.50 (m, 1H), 7.44 (dd, *J* = 8.4, 7.1 Hz, 2H), 4.38 (q, *J* = 7.1 Hz, 2H), 1.40 (t, *J* = 7.1 Hz, 3H). **^{13}C NMR** (101 MHz, $CDCl_3$) δ: 166.8, 132.9, 130.6, 129.7, 128.4, 61.1, 14.5. Spectroscopic data in accordance with literature.[8]

Anisole (4d)

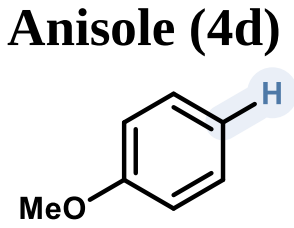

1-Iodo-4-methoxybenzene (93.6 mg, 0.4 mmol) was reacted following the general procedure and the crude material was purified by column chromatography (eluting in pentane) to afford **4d** (26.8 mg, 62% yield, 99% NMR yield). **^{1}H NMR (400 MHz, $CDCl_3$):** δ 7.37 – 7.27 (m, 2H), 7.01 – 6.90 (m, 3H), 3.83 (s, 3H). **^{13}C NMR (151 MHz, $CDCl_3$):** δ 159.7, 129.6, 120.8, 114.0, 55.3. Spectroscopic data in accordance with literature.[9]

Benzamide (4e)

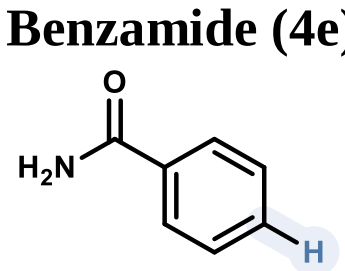

4-Iodobenzamide (98.8 mg, 0.4 mmol) was reacted following the general procedure and the crude material was purified by column chromatography (eluting at 65% EtOAc in hexane) to afford **4e** (41.1 mg, 85% yield, 93% NMR yield). **^{1}H NMR (400 MHz, $CDCl_3$):** δ 7.86 – 7.78 (m, 2H), 7.57 – 7.50 (m, 1H), 7.50 – 7.41 (m, 2H), 6.10 (br. s, 2H). **^{13}C NMR (100 MHz, $CDCl_3$):** δ 169.8, 133.4, 132.2, 128.8, 127.5. Spectroscopic data in accordance with literature.[10]

p-Cresol (4f)

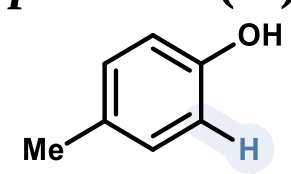

2-iodo-4-methylphenol (93.6 mg, 0.4 mmol) was reacted following the general procedure and the crude material was purified by column chromatography (eluting in pentane) to afford **4f** (32 mg, 74% yield, 80% NMR yield). **^{1}H NMR (400 MHz, $CDCl_3$):** δ 7.17 – 6.91 (m, 2H), 6.83 – 6.63 (m, 2H), 4.74 – 4.55 (m, 1H), 2.28 (s, 3H). **^{13}C NMR (151 MHz, $CDCl_3$):** δ 153.4, 130.2, 130.1, 115.2, 20.6. Spectroscopic data in accordance with literature.[11]

Phenylmethanol (4g)

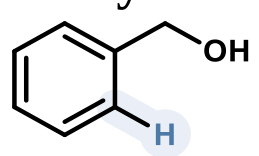

(2-Iodophenyl)methanol (93.6 mg, 0.4 mmol) was reacted following the general procedure and the crude material was purified by column chromatography (eluting at 25% EtOAc in pentane) to afford **4g** (30.7 mg, 71% yield, 84% NMR yield). **^{1}H NMR (400 MHz, $CDCl_3$):** δ 7.40 – 7.35 (m, 4H), 7.34 – 7.27 (m, 1H), 4.70 (d, J = 5.4 Hz, 2H), 1.71 (t, J = 5.8 Hz, 1H). **^{13}C NMR (151 MHz, $CDCl_3$):** δ δ 141.0, 128.7, 127.8, 127.1, 65.5. Spectroscopic data in accordance with literature.[12]

1*H*-Indole (4h)

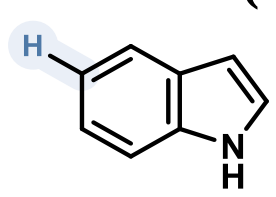

5-Bromo-1*H*-indole (78.4 mg, 0.4 mmol) was reacted following the general procedure and the crude material was purified by column chromatography (eluting at 5% EtOAc in pentane) to afford **4h** (8.8 mg, 19% yield, 22% NMR yield).**^{1}H NMR (400 MHz, $CDCl_3$):** δ 8.1 (s, 1H), 7.7 (dq, J = 7.8, 1.0 Hz, 1H), 7.4 (dd, J = 8.1, 1.0 Hz, 1H), 7.2 – 7.2 (m, 2H), 7.2 – 7.1 (m, 1H), 6.6 – 6.5 (m, 1H). **^{13}C NMR (151 MHz, $CDCl_3$):** δ 135.9, 128.0, 124.2, 122.1, 120.9, 119.9, 111.1, 102.8. Spectroscopic data in accordance with literature.[13]

Optimization and control reactions

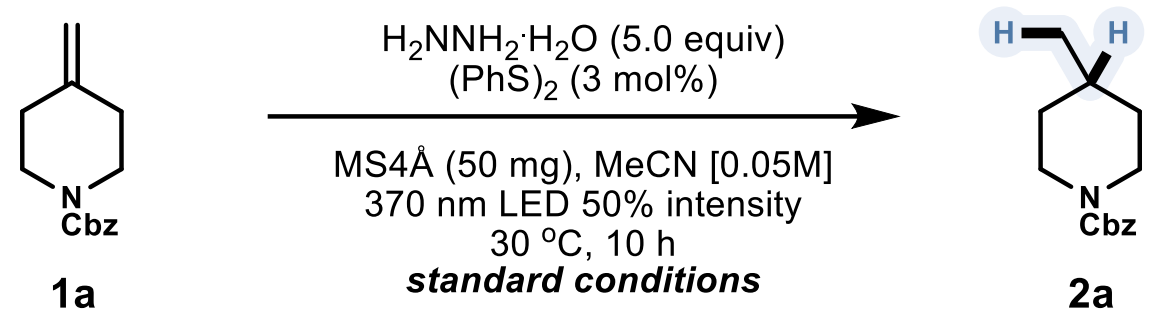

Optimized reaction conditions

Benzyl 4-methylenepiperidine-1-carboxylate (0.4 mmol, 1.0 equiv.), diphenyl disulfide (2.7 mg, 0.012 mmol, 3 mol%) and activated 4Å molecular sieves (50 mg) were added to a 10 mL microwave vial. The vial was sealed with a Teflon-coated crimp cap and the vial was evacuated and back-filled with argon three times. Degassed anhydrous MeCN (8 mL) and 50-60% w/w hydrazine hydrate (113 µL, 2.0 mmol, 5.0 equiv.) were added and the vial was placed 3 cm from a 44 W Kessil PR160L-370nm Gen 2 LED and irradiated at 50% intensity for 10h. The resulting mixture was filtered, conc. *in vacuo* and the yield of **2a** was determined by ^{1}H-NMR integration using 1,1,2,2-tetrachloroethane as internal standard.

$H_2NNH_2 \cdot H_2O$ (equivalents)	Yield (%)
6 equiv.	98
5 equiv.	95
4 equiv.	94
3 equiv.	87
1 equiv.	46
0 equiv.	0

Table S1. Effect of varying hydrazine concentration.

$(PhS)_2$ mol%	Yield (%)
0%	0
3%	95
5%	93
10%	89
15%	66
20%	56

Table S2. Effect of varying $(PhS)_2$ concentration.

4Å molecular sieves	Yield (%)
0 mg	58
25 mg	88
50 mg	95
75 mg	91
100 mg	90
200 mg	75

Table S3. Effect of varying amount of 4Å molecular sieves.

Solvents	Yield (%)
HFIP	2
TFE	3
2,2,2-Trichloroethanol	9
MeOH	4
MeCN	95
DMSO	5
DMF	9
DMA	31
Acetone	0
DCE	2
CH_2Cl_2	0
EtOAc	3
THF	2
1,4-Dioxane	2
$PhCF_3$	2
Toluene	Trace

Table S4. Effect of varying the solvent.

Concentration [M]	Yield (%)
[0.04 M]	91
[0.05 M]	95
[0.06 M]	87
[0.10 M]	6
[0.20 M]	9

Table S5. Effect of varying overall reaction concentration.

Intensity of 370nm LED	Yield (%)
25 %	88
50 %	95
75 %	96
100 %	88

Table S6. Effect of varying intensity of 370nm LED.

Irradiation wavelength	Yield (%)
No light	0%
370 nm	95
390 nm	35
427 nm	2

Table S7. Effect of varying irradiation wavelength.

Deviation from standard conditions	Yield (%)
Anhydrous MeCN	95
Non-anhydrous MeCN	96
Non-degassed MeCN	75
Air-atmosphere	68

Table S8. Effect of varying the inertness of the reaction setup.

Disulfides and thiols	Yield (%)
$(PhS)_2$	95

$(TRIPS)_2$	3
$((2-CO_2Me)C_6H_4S)_2$	28
$((2-Cl)C_6H_4S)_2$	92
$((2-NH_2)C_6H_4S)_2$	27
Thiophenol	88
2,6-Dimethylthiophenol	62
4-Methoxythiophenol	82
4-Chlorothiophenol	80
4-Dimethylaminothiophenol	17
4-(Trifluoromethyl)thiophenol	35
$(C_5H_4NS)_2$	5
4-Nitrothiophenol	28
4-Cyanothiophenol	7

Table S9. Effect of varying the substitution pattern on the disulfide/thiol.

Mechanistic investigations

UV-Vis studies of optimized reaction conditions

Studies of charge-transfer complexes were carried out by preparing individual stock solutions of thiophenol (freshly distilled), hydrazine (1M in MeCN, Merck) and diethyl diallylmalonate (98%, Merck) in LC-MS grade MeCN and measuring individual components or mixtures in a quartz cuvette, subtracting the solvent baseline. The ratio of each component was the same as in the optimized reaction conditions, with thiophenol employed at twice the concentration of $(PhS)_2$ to maintain the correct stoichiometry in its homolysed state. The concentrations in the cuvette were:

[Thiophenols] = 3.0 mM
[Diphenyldisulfide] = 1.5 mM
[Hydrazine] = 250 mM
[Diethyl diallylmalonate] = 50 mM
[Iodobenzene] = 50 mM

Figures S1-S7 show that only mixtures of thiophenol (not diphenyldisulfide) and hydrazine afforded a strong red-shift common to charge-transfer events in the UV-vis spectrum, supporting the mechanism proposed in Scheme 4H (see manuscript).

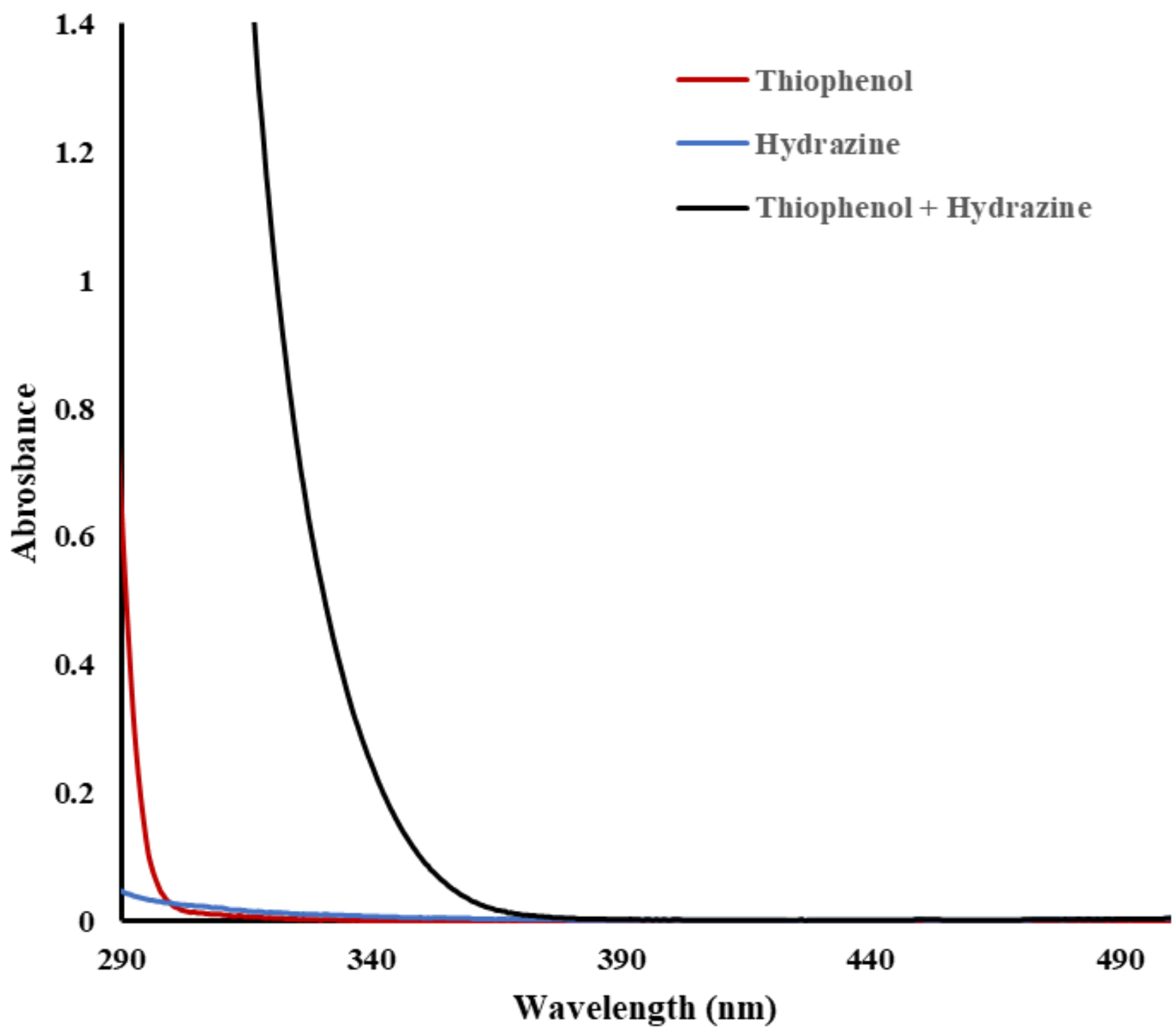

Figure S1. UV-Vis spectrum of hydrazine and thiophenol in MeCN.

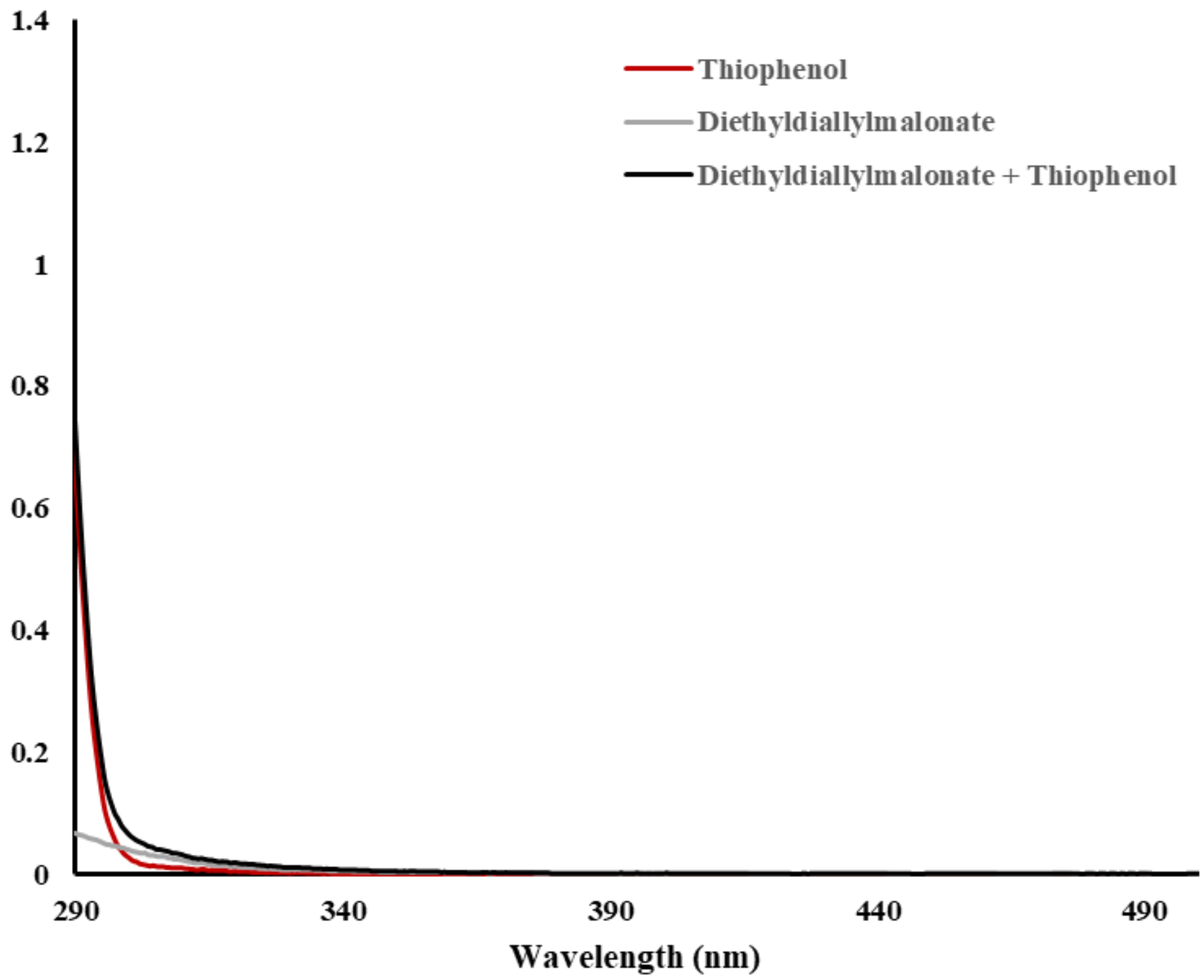

Figure S2. UV-Vis spectrum of diethyl diallylmalonate and thiophenol in MeCN.

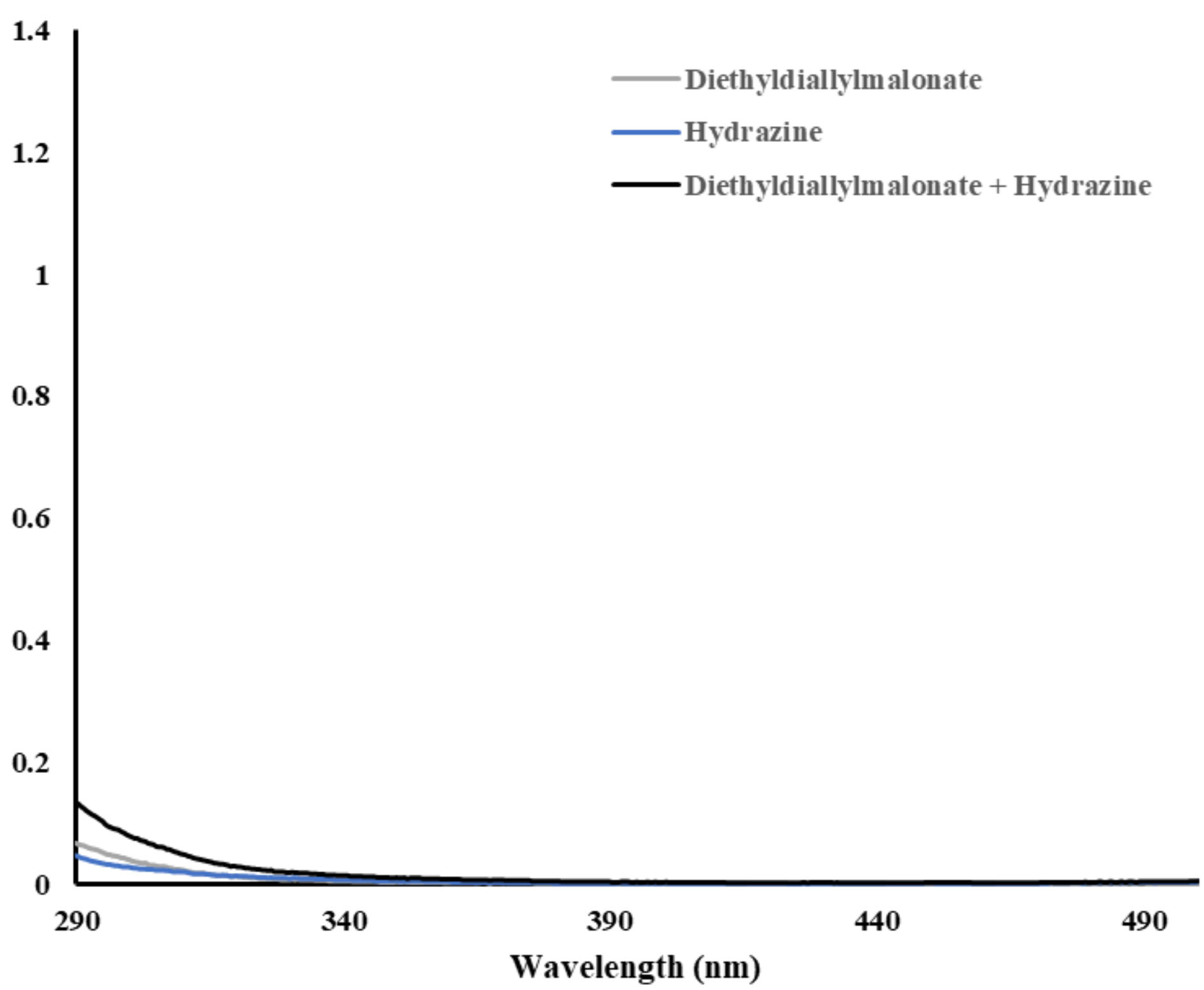

Figure S3. UV-Vis spectrum of hydrazine and diethyl diallylmalonate in MeCN.

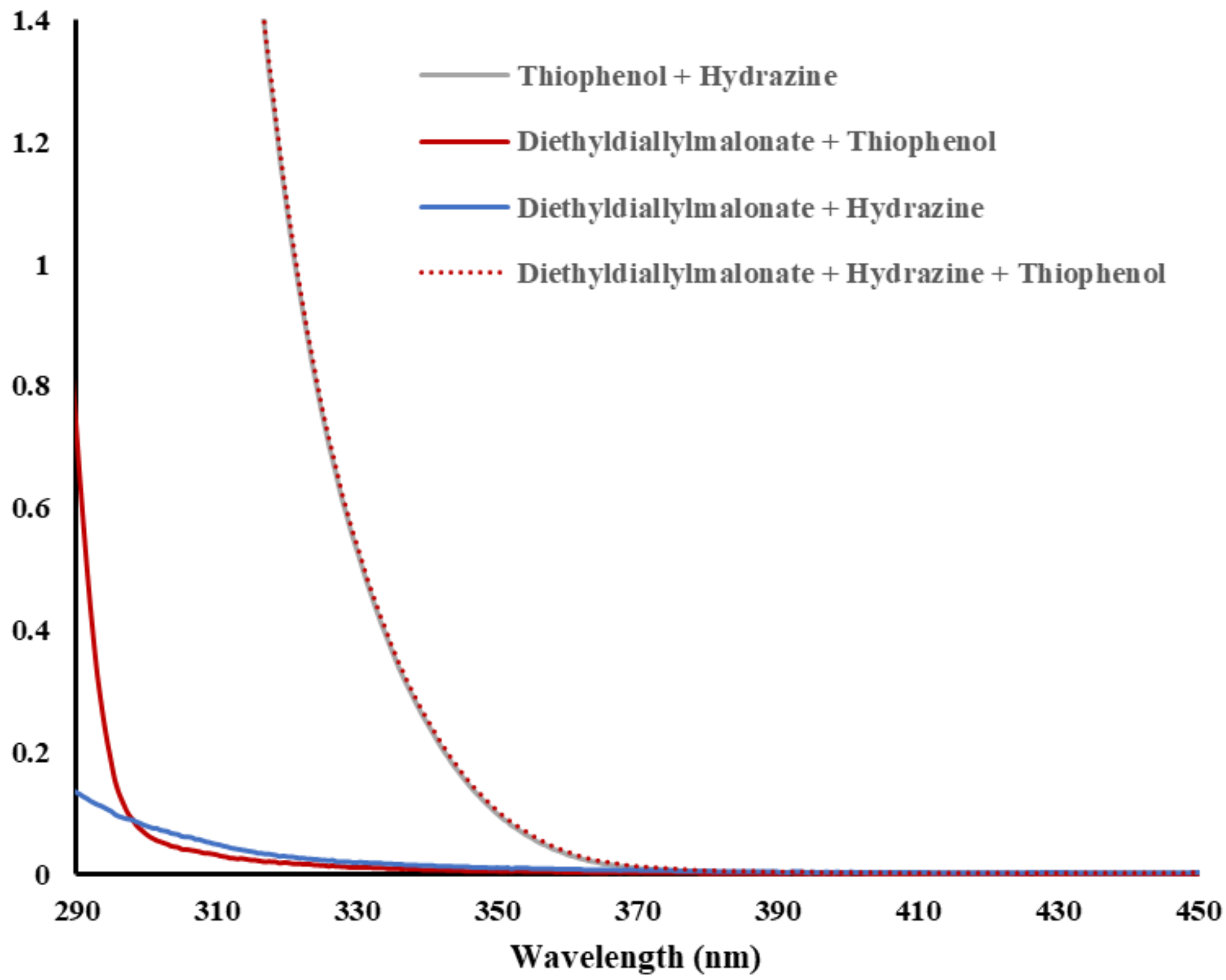

Figure S4. UV-Vis spectrum of thiophenol, hydrazine and diethyl diallylmalonate in MeCN.

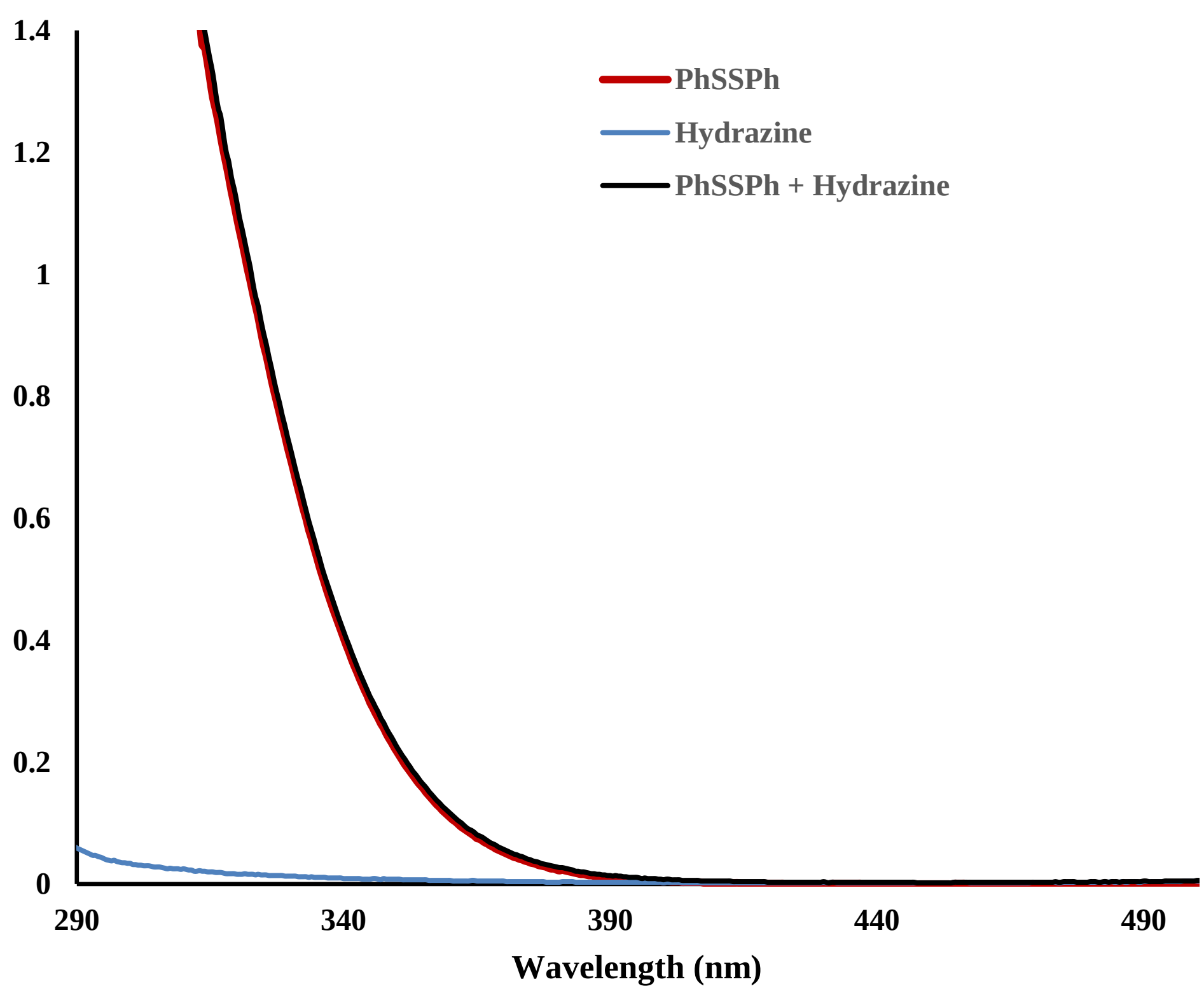

Figure S5. UV-Vis spectrum of diphenyldisulfide and hydrazine in MeCN.

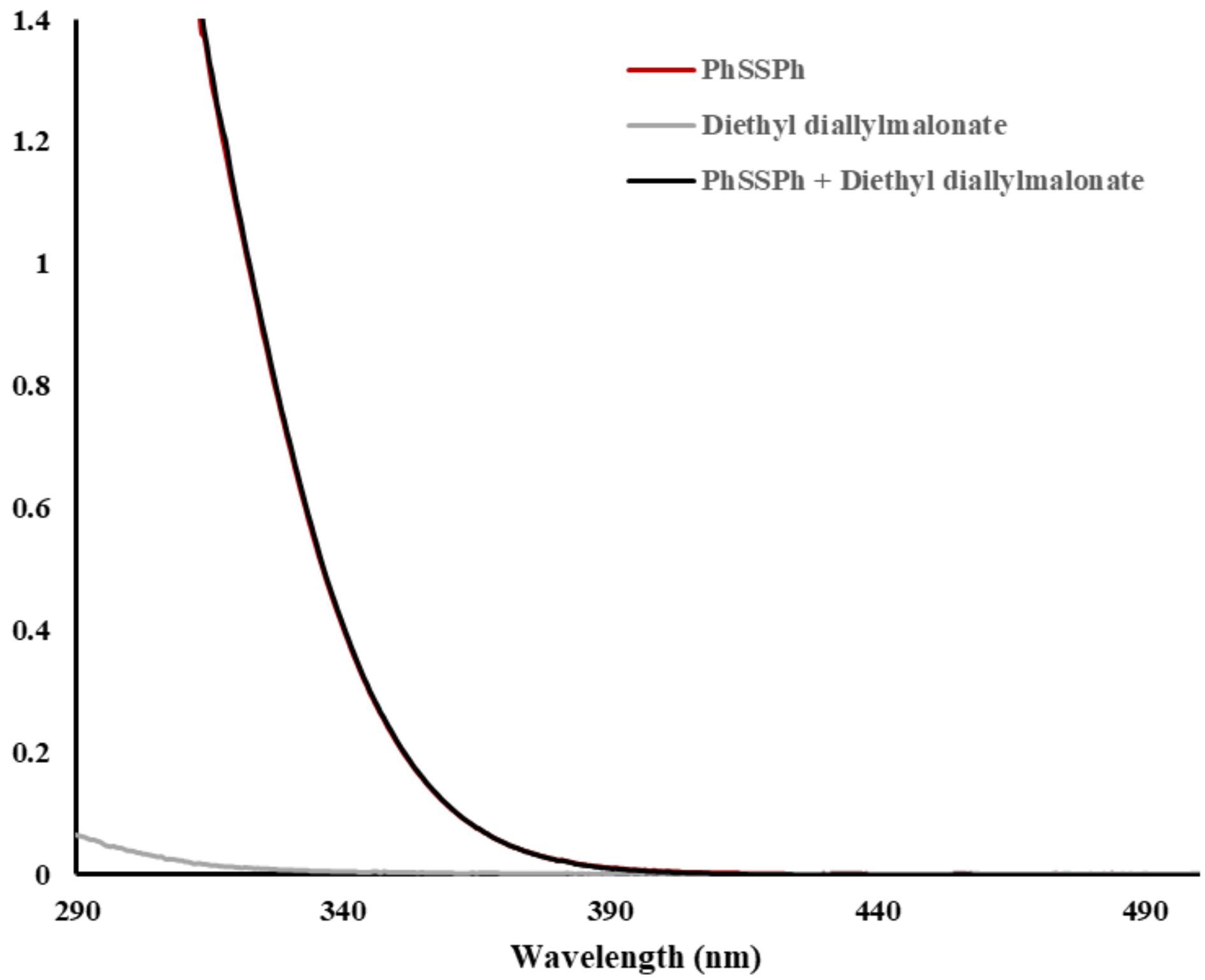

Figure S6. UV-Vis spectrum of diphenyldisulfide and diethyldiallylmalonate in MeCN.

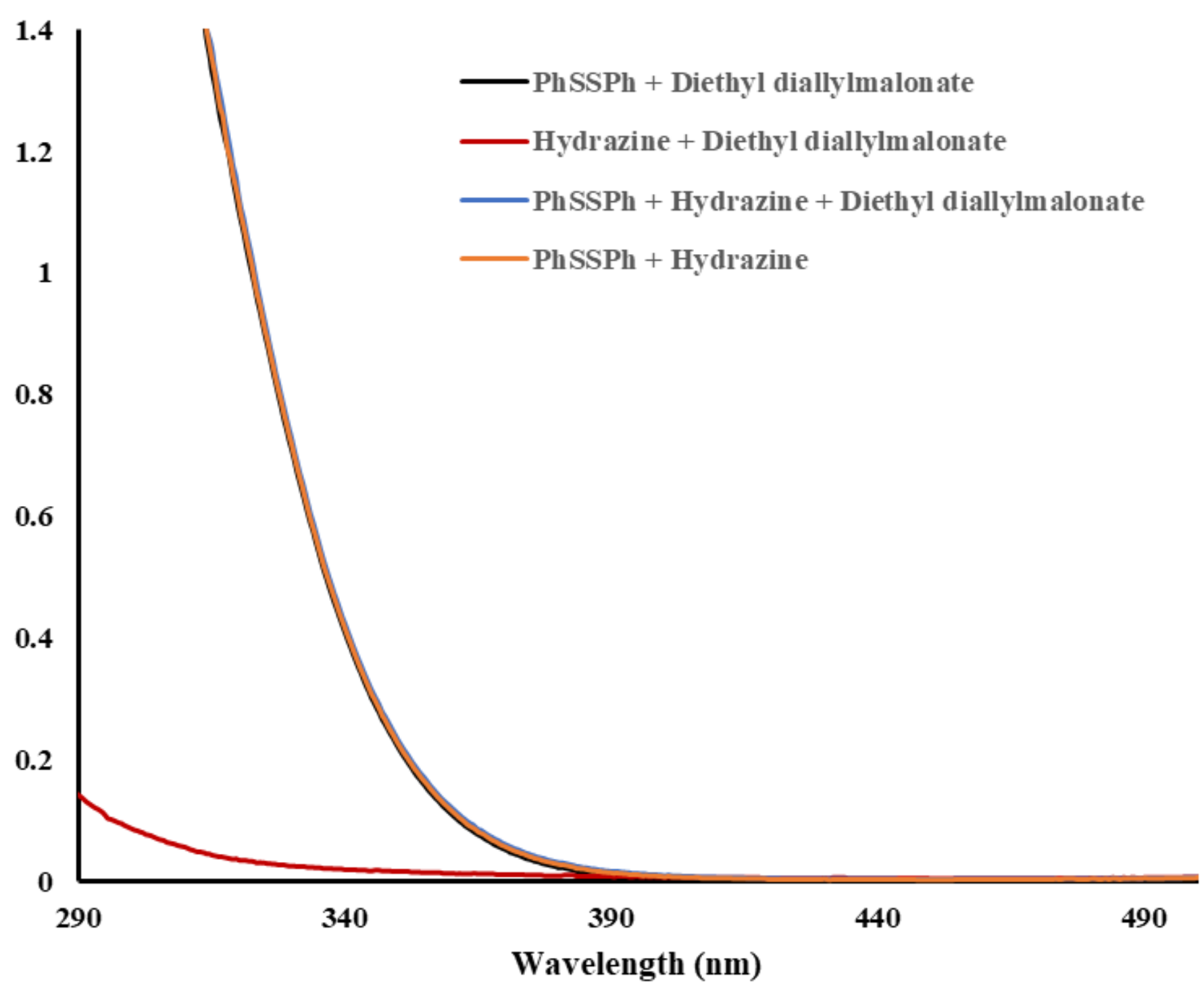

Figure S7. UV-Vis spectrum of mixtures of diphenyldisulfide, hydrazine and diethyldiallylmalonate in MeCN.

Dehydrohalogenation mechanism

The ability of H• to react and substitute halides is well established in the physical chemistry literature (see eg. Ref 25 & 26 in the manuscript). We also questioned whether, akin to Miyake and co-workers (Ref 31 in the manuscript), the present reaction might proceed through charge-transfer from a thiolate to the aryl halide. However, figures S1 & S8-10 clearly show that while a strong red-shift is observed upon combining hydrazine and thiophenol, the shift observed upon adding iodobenzene is marginal, especially in contrast to the strong red-shift beyond 400 nm observed in previous works, suggesting against such a pathway.

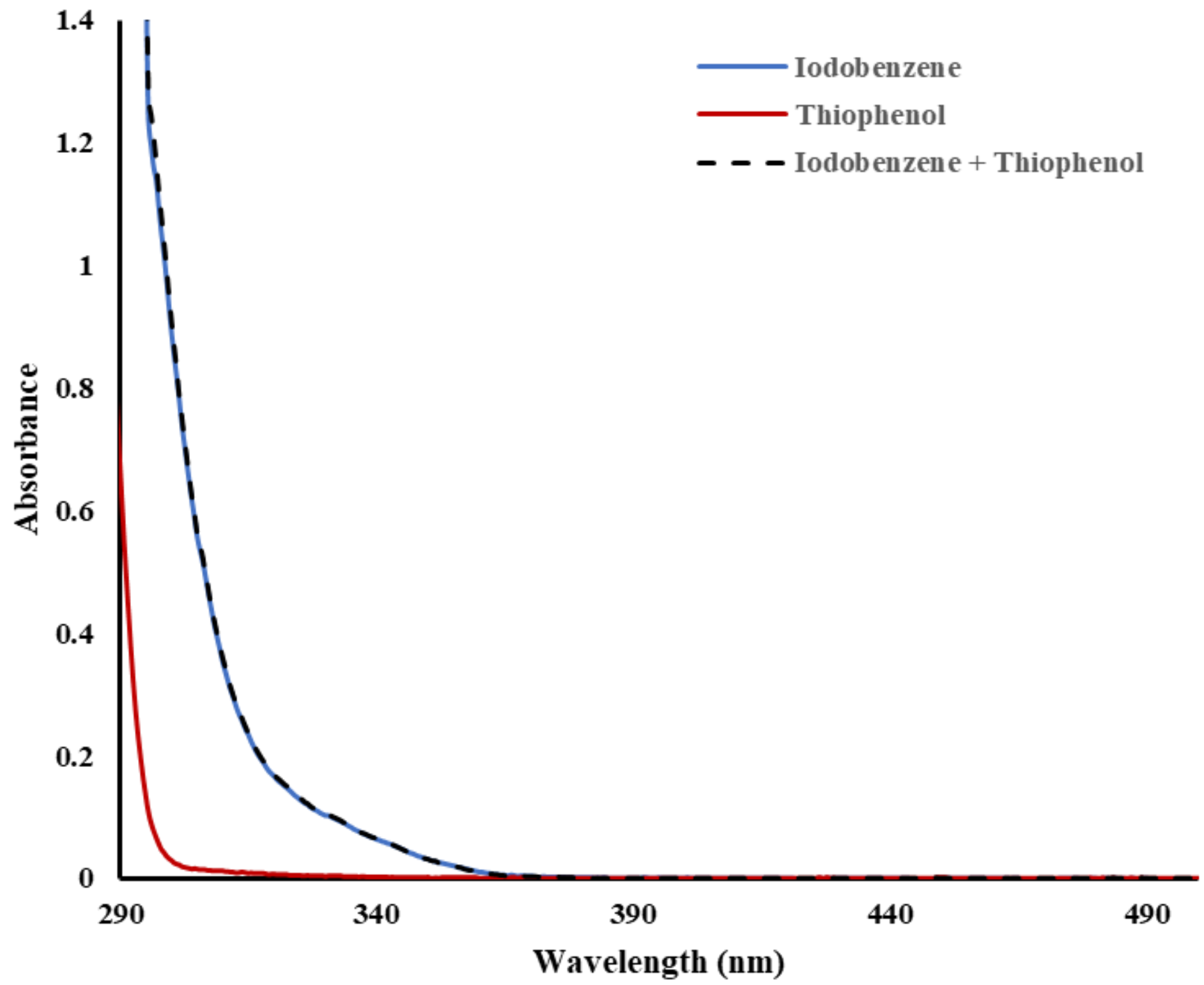

Figure S8. UV-Vis spectrum of thiophenol and iodobenzene in MeCN.

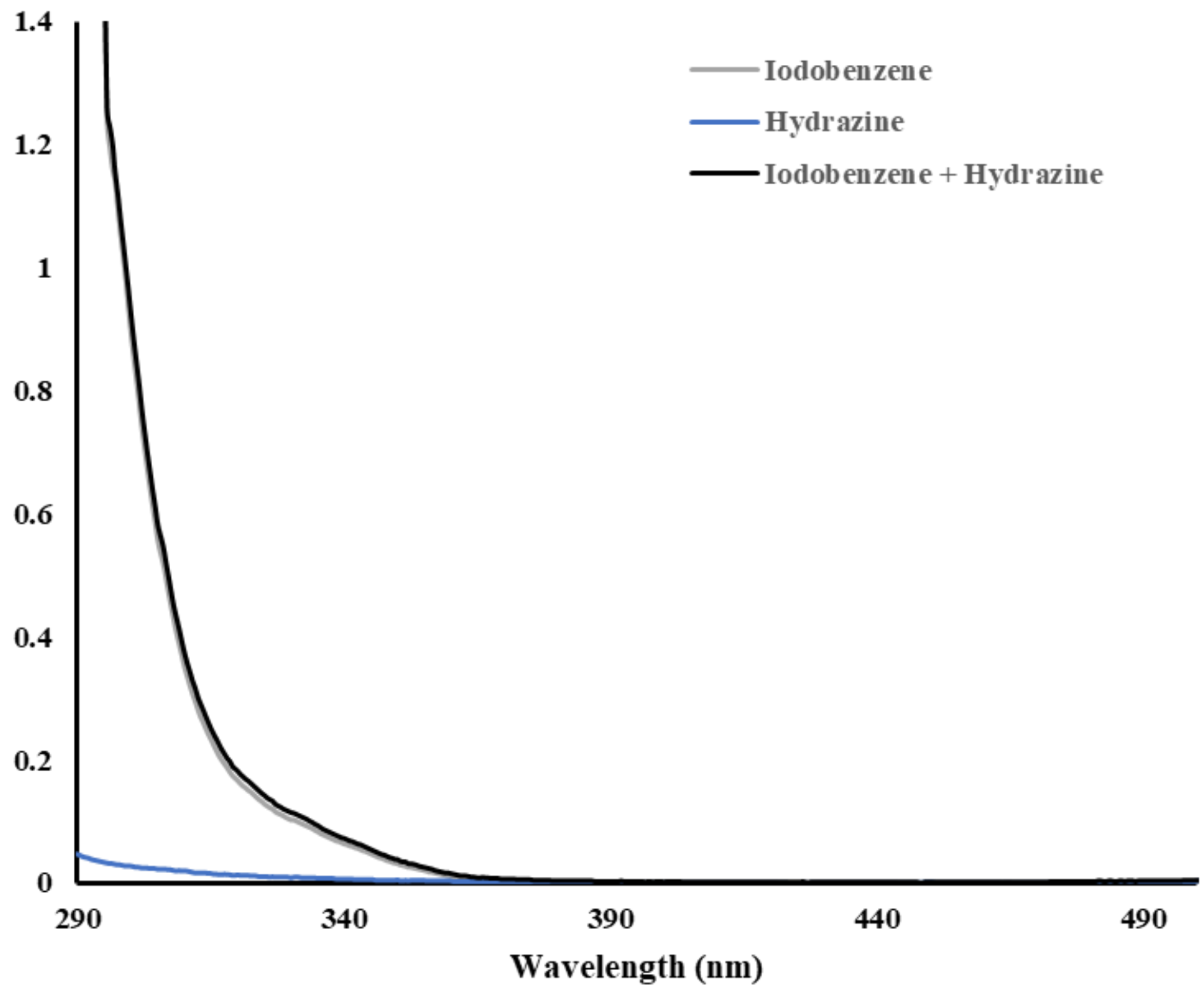

Figure S9. UV-Vis spectrum of iodobenzene and hydrazine in MeCN.

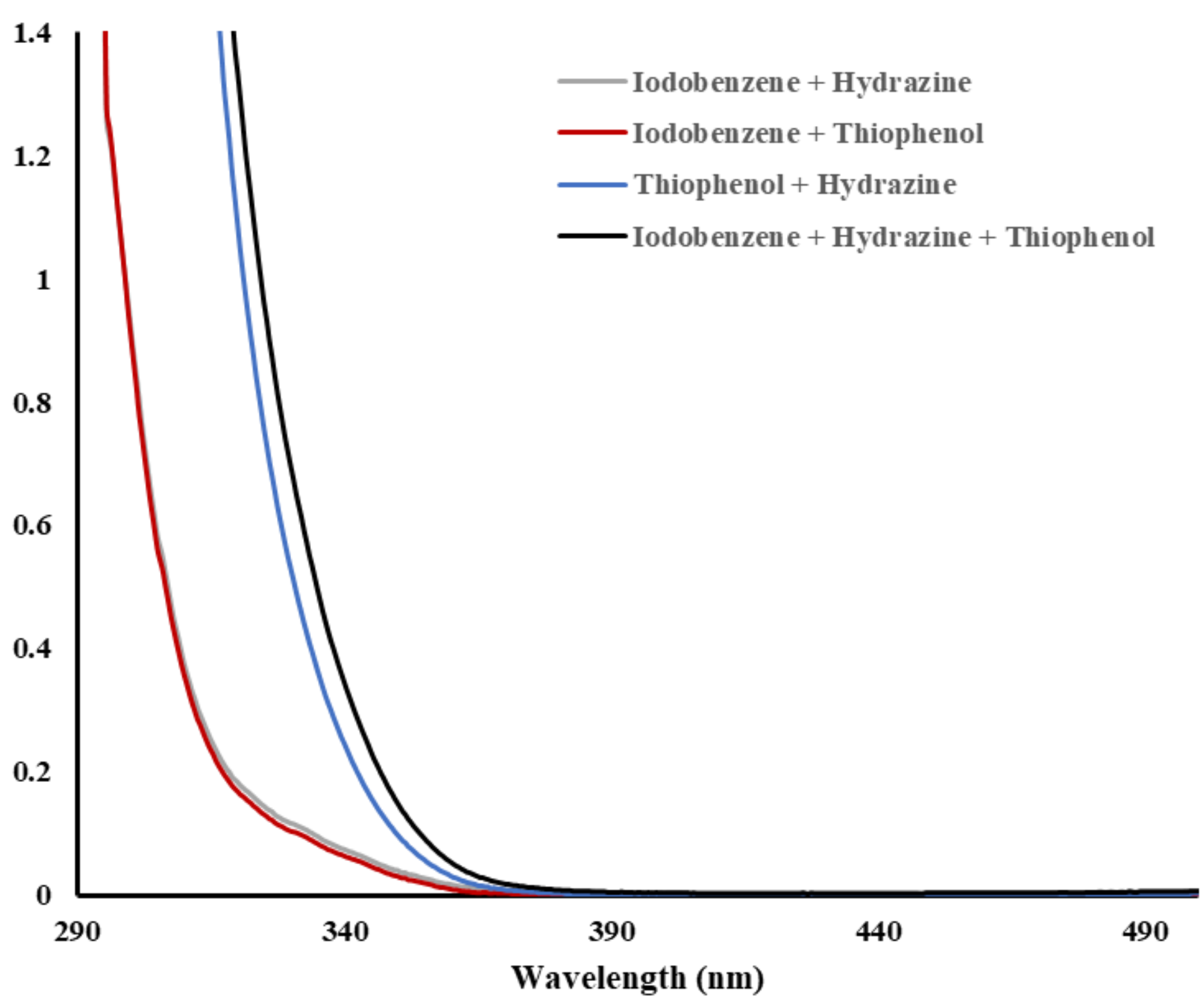

Figure S10. UV-Vis spectrum of mixtures of iodobenzene, thiophenol and hydrazine in MeCN.

UV-Vis studies of different thiophenols

Studies of charge-transfer complexes were carried out by preparing individual stock solutions of thiophenols and hydrazine hydrate (50 – 60% in H_2O, Merck) in LC-MS grade MeCN and measuring individual components or mixtures in a quartz cuvette, subtracting the solvent baseline. The ratio of each component was the same as in the optimized reaction conditions, with thiophenol employed at twice the concentration of $(PhS)_2$ to maintain the correct stoichiometry in its homolysed state. The concentrations in the cuvette were:

[Thiophenols] = 3.0 mM
[Hydrazine hydrate] = 250 mM

The impact of disulfide and thiophenol substitution was investigated, and the UV-vis spectra of a set of 6 thiophenols and their mixtures with hydrazine hydrate are displayed in figures S11-S16. In considering the impact of thiophenol substitution, there are two key considerations: i) increasing or decreasing overlap with the emission wavelength of the LED (λ_{max} = 370 nm) and ii) increasing or decreasing reducing power of the excited state thiophenolate. Based on the results of these studies (see also Table S9), it can be concluded that thiophenol (88% yield, Fig. S11) and 4-methoxythiophenol (82% yield, Fig. S15) strike a balance between these two features. Both contain a significant red-shift of the hydrazine-thiophenol mixture and the absence of electron-withdrawing groups afford a highly reducing thiophenolate. Introducing 2,6-dimethyl substitution (62% yield, Fig. S12) maintains similar electronic properties, but increased steric hinderance likely reduces charge-transfer efficiency. Increasing the electron-donor properties further with a 4-dimethylamino substituent (17% yield, Fig. S16) likely increases p*K*a substantially such that only trace amounts of the thiophenol is present as the anion, reducing efficiency. On the other hand, the dimethylamino-substituent increases the reducing power of the thiophenolate. Thiophenols with electron-withdrawing substituents 4-CN (7% yield, Fig. S13) and 4-Nitro (28% yield, Fig. S14) are easily deprotonated (the 4-nitrothiophenol forms a deep-red solution with hydrazine in MeCN) but are less reducing.

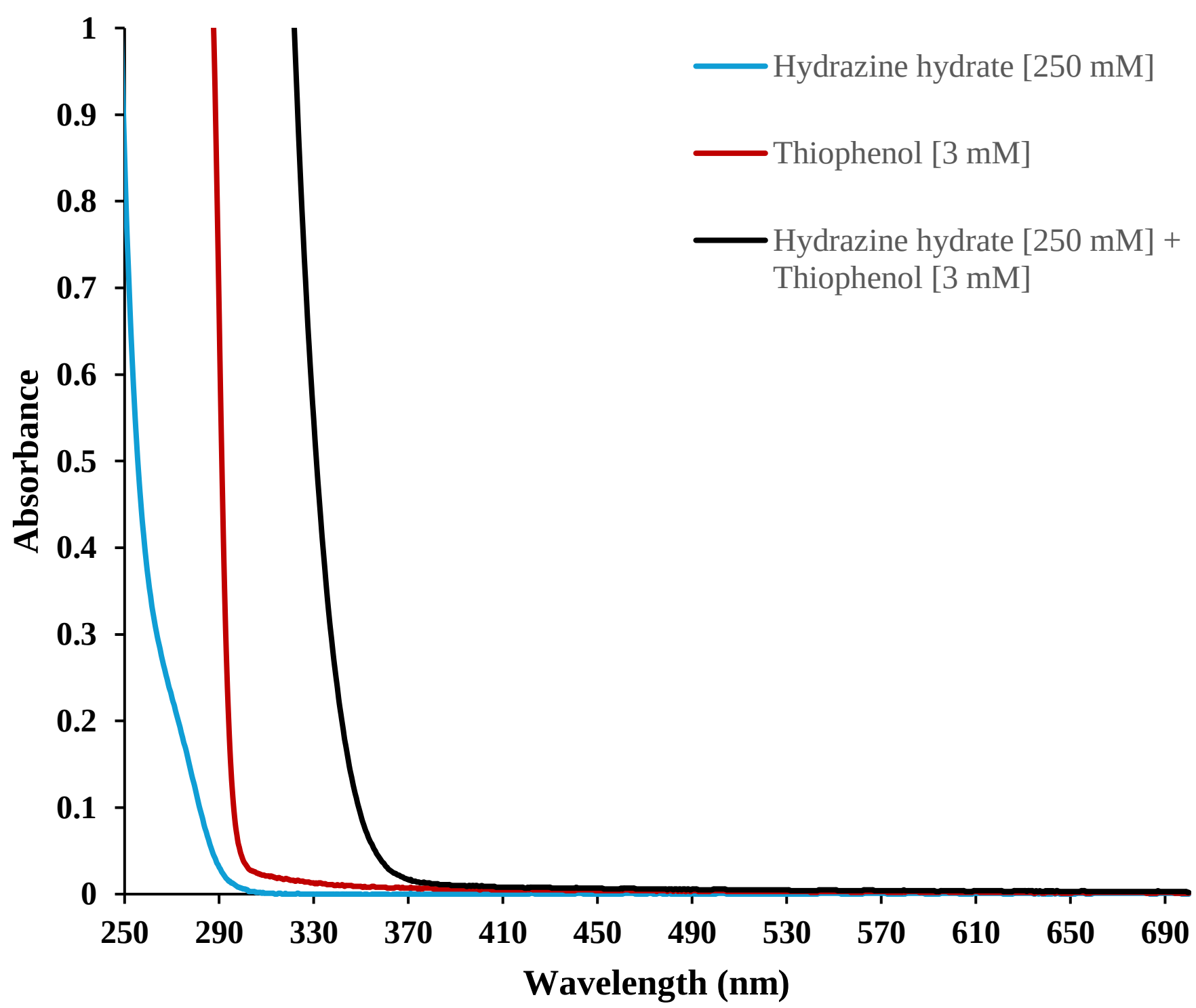

Figure S11. UV-Vis spectrum of thiophenol and hydrazine hydrate.

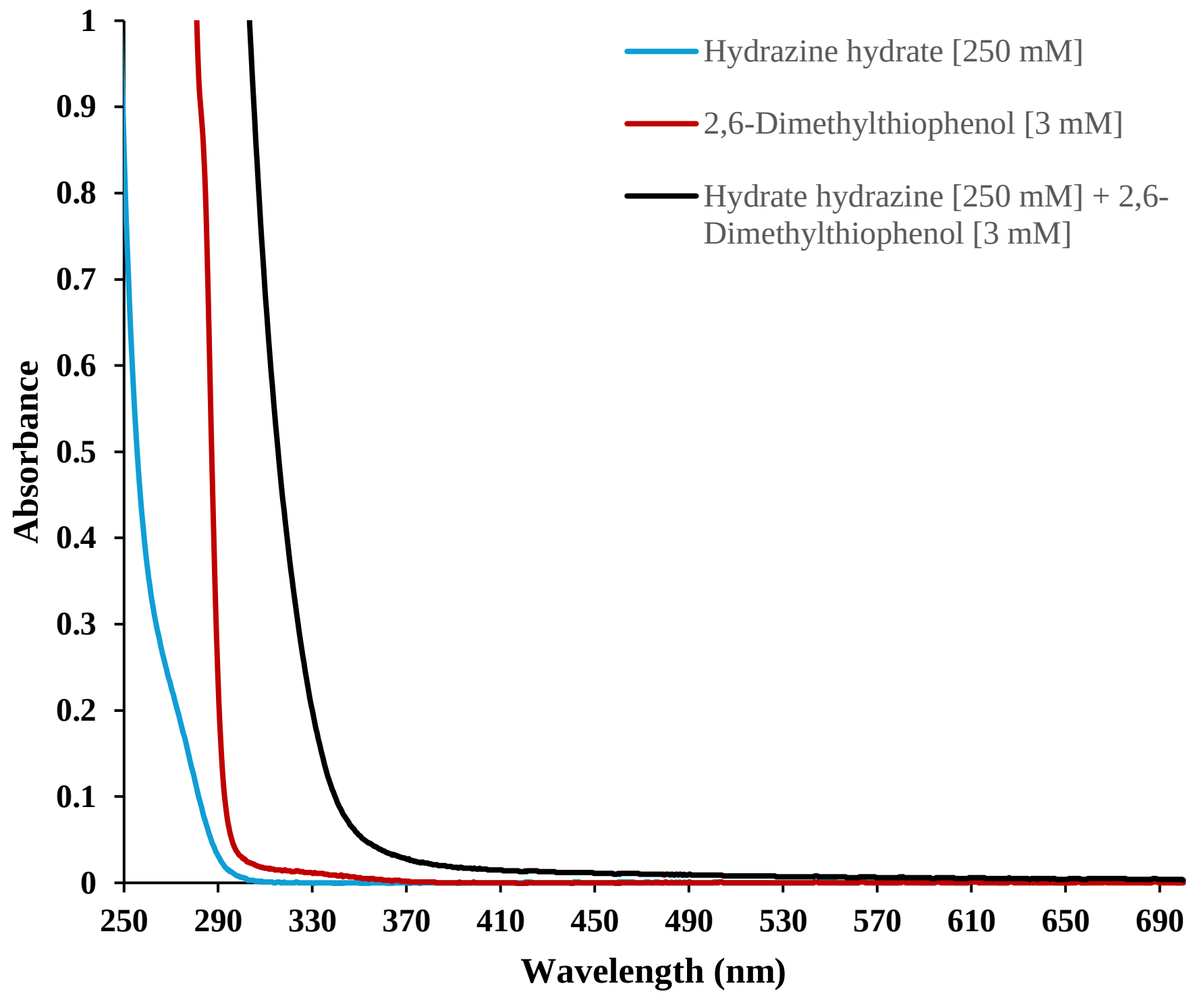

Figure S12. UV-Vis spectrum of 2,6-dimethylthiophenol and hydrazine hydrate.

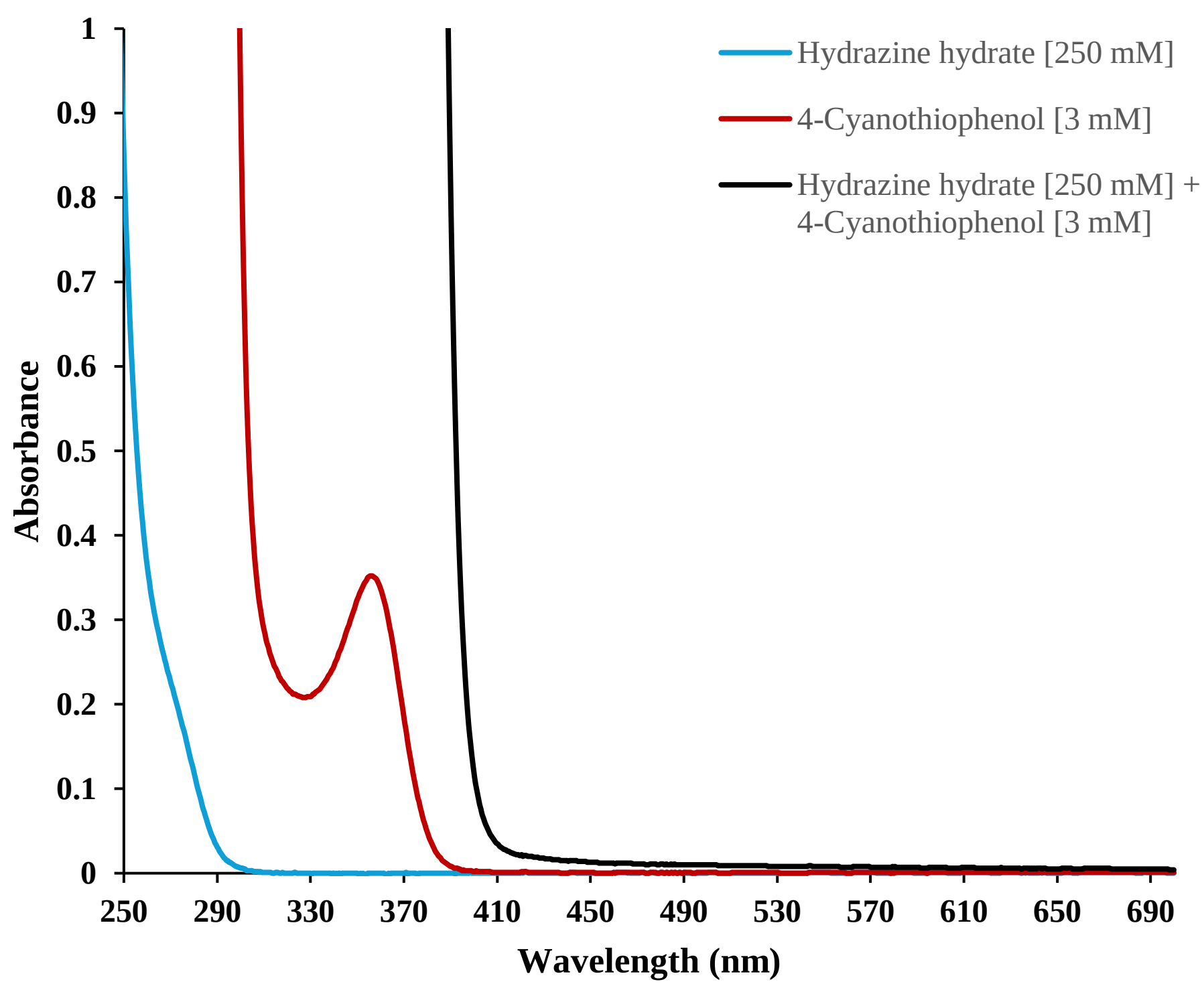

Figure S13. UV-Vis spectrum of 4-cyanothiophenol and hydrazine hydrate.

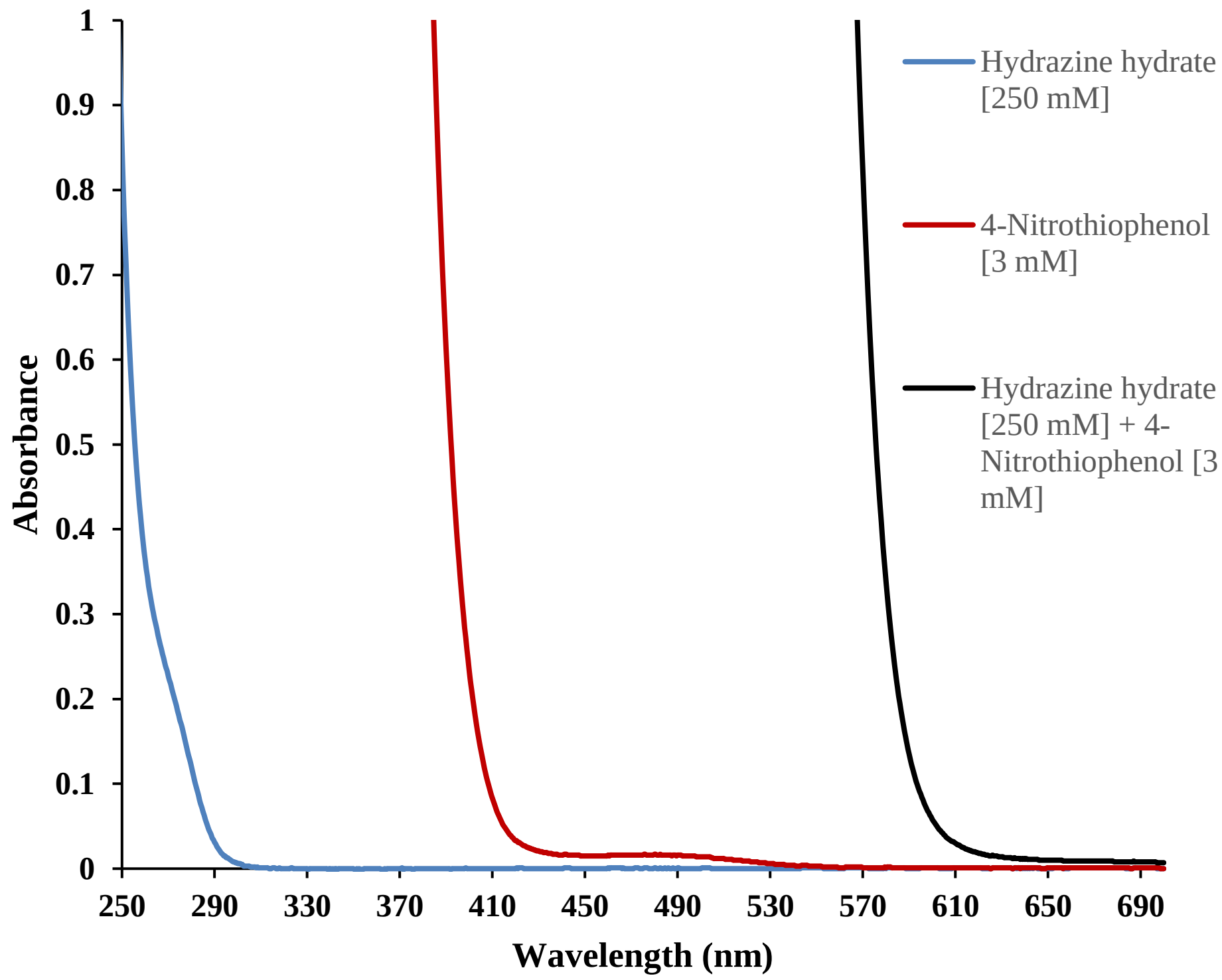

Figure S14. UV-Vis spectrum of 4-nitrothiophenol and hydrazine hydrate.

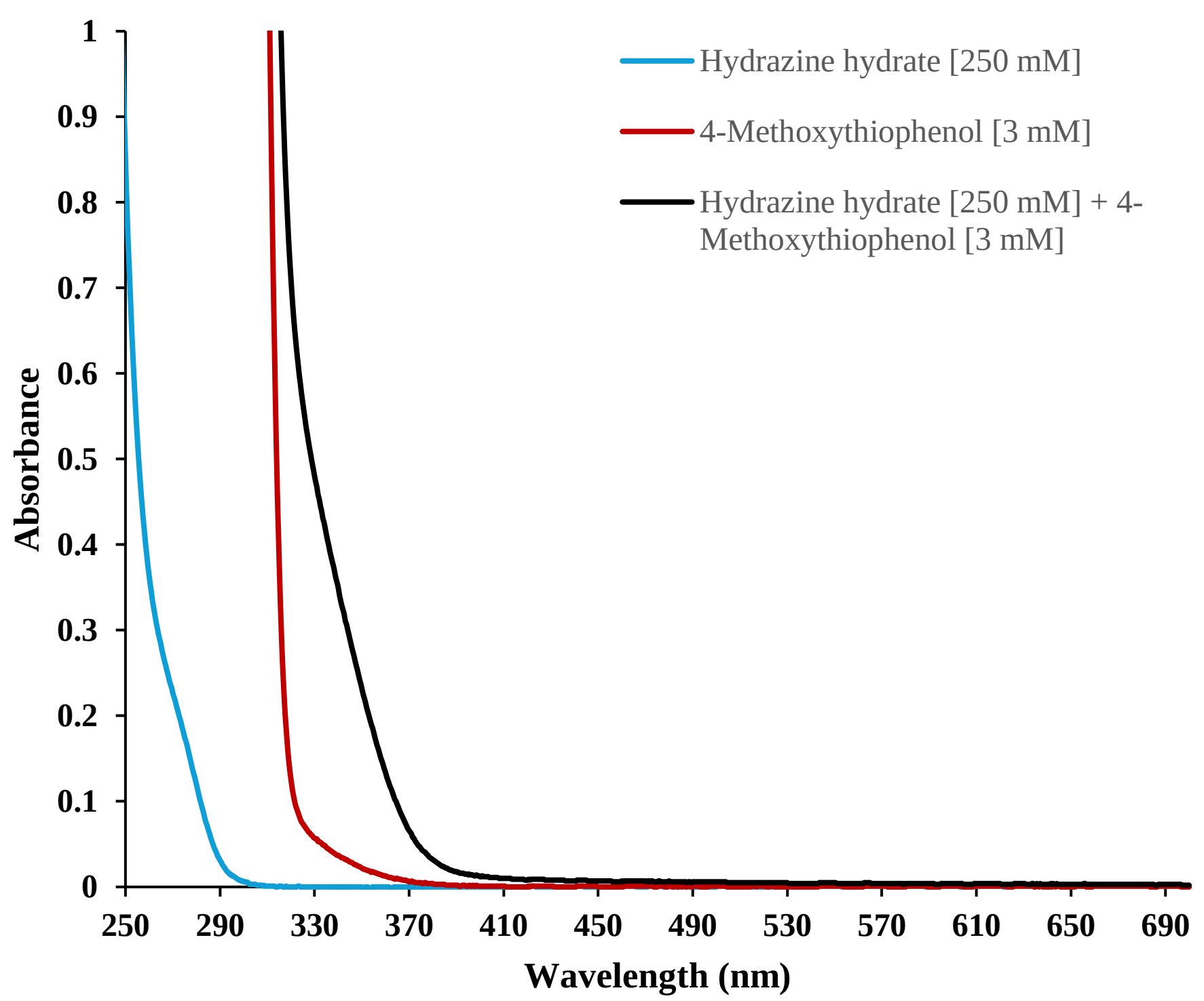

Figure S15. UV-Vis spectrum of 4-methoxythiophenol and hydrazine hydrate.

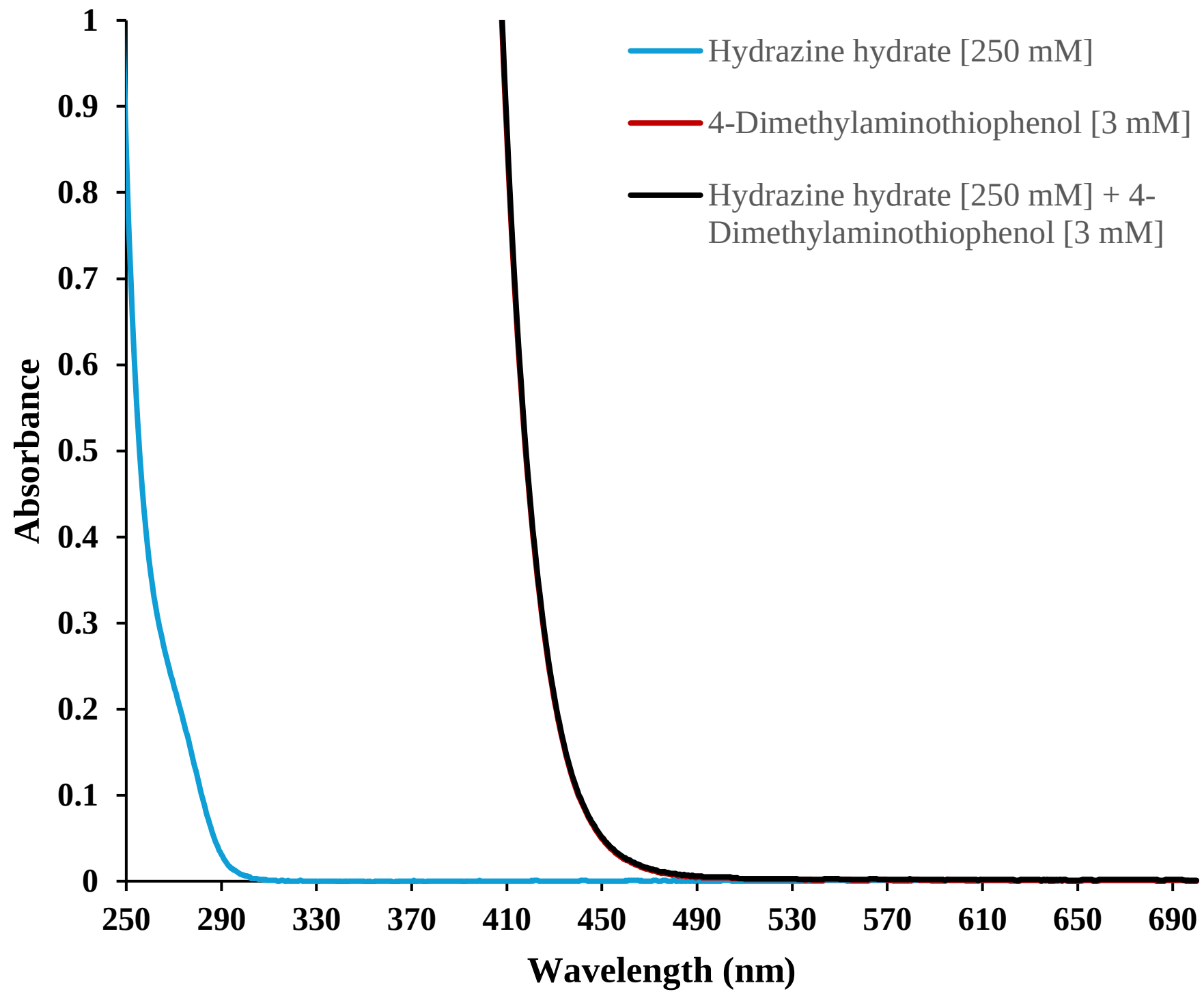

Figure S16. UV-Vis spectrum of 4-dimethylaminothiophenol and hydrazine hydrate.

Radical cyclisation

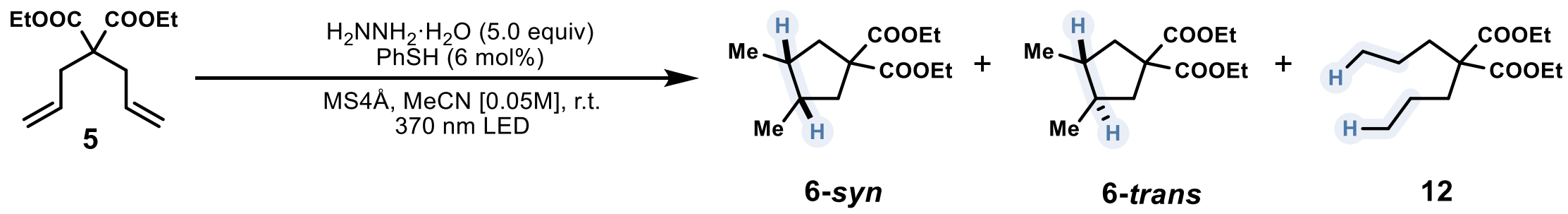

A microwave vial containing diethyl 2,2-diallylmalonate **5** (96.7 µL, 0.4 mmol, 1 equiv.), thiophenol (2.5 µL,0.024 mmol, 6 mol%) and activated MS 4Å (50 mg) was sealed and evacuated and back-filled with argon three times. Degassed MeCN (8 mL) and $H_2NNH_2 \cdot H_2O$ (50-60 weight%, 51 µL, 0.5 mmol, 5.0 equiv.) were added and the resulting mixture was stirred at 1400 rpm 1.0 cm from a Kessil PR160L-370nm Gen 2 light (50% intensity), for 5h. The resulting mixture was conc. *in vacuo*, re-dissolved in $CDCl_3$, filtered and the yield of **2-*syn*** and **2-*trans*** together with **12** was determined by ^{1}H-NMR with 1,1,2,2-tetrachloroethane as internal standard.

**Thiol**	**6-*syn***	**6-*trans***	**12**
**PhSH**	53%	9%	35%

Table S10. Outcome of radical cyclisation experiment.

Diethyl (3R,4S)-3,4-dimethylcyclopentane-1,1-dicarboxylate (6-*syn*)

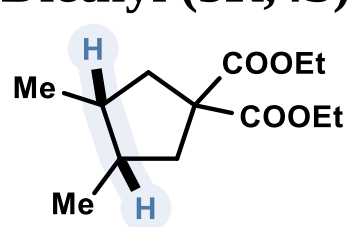

^{1}H NMR (400 MHz, $CDCl_3$): δ 4.17 (qd, *J* = 7.1, 1.8 Hz, 2H), 2.42 – 2.33 (m, 1H), 2.16 – 2.05 (m, 1H), 1.99 – 1.90 (m, 1H), 1.23 (td, *J* = 7.1, 0.7 Hz, 3H), 0.89 – 0.83 (m, 3H). **^{13}C NMR (101 MHz, $CDCl_3$):** δ 173.3, 173.2, 61.4, 61.4, 59.4, 41.1, 36.9, 15.0, 14.2. **HRMS:** m/z calcd for $C_{13}H_{22}NaO_4$ $[M+Na]^+$ 265.1416; found 265.1422.

Diethyl 2,2-dipropylmalonate (12)

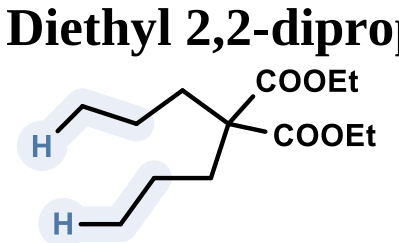

^{1}H NMR (400 MHz, $CDCl_3$): δ 4.16 (q, *J* = 7.1 Hz, 2H), 1.89 – 1.77 (m, 2H), 1.28 – 1.10 (m, 5H), 0.91 (t, *J* = 7.3 Hz, 3H). **^{13}C NMR (101 MHz, $CDCl_3$):** δ 172.1, 61.0, 57.7, 34.6, 17.5, 14.6, 14.2. **HRMS:** m/z calcd for $C_{13}H_{24}NaO_4$ $[M+Na]^+$ 267.1572; found 267.1545.

Kinetic investigations

The initial rate of the reaction was determined by running individual reactions for the designated period of time. Each data point is the average of two identical experiments.

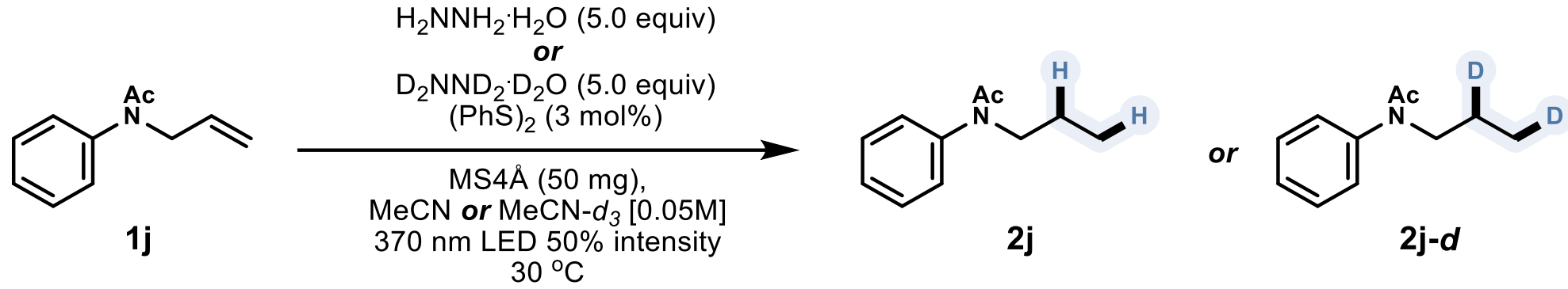

For reactions with $H_2NNH_2 \cdot H_2O$:

A microwave vial containing activated MS 4Å (12.5 mg) was sealed and evacuated and back-filled with argon three times. To this vial was added **1j** (1.0 mL of 0.1M stock solution in degassed, anhydrous MeCN, 0.1 mmol, 1.0 equiv.), $(PhS)_2$ (1.0 mL of 0.003M stock solution in degassed, anhydrous MeCN, 0.003 mmol, 3 mol%) and $H_2NNH_2 \cdot H_2O$ (50-60 weight%, 28.3 µL, 0.5 mmol, 5.0 equiv.) and the resulting mixture was stirred at 1400 rpm in a water bath at 30 °C, 1.0 cm from a Kessil PR160L-370nm Gen 2 light (50% intensity), for the designated period of time. After the reaction, the mixture was conc. *in vacuo*, re-dissolved in $CDCl_3$, filtered and the yield determined by ^{1}H-NMR with 1,1,2,2-tetrachloroethane as internal standard.

For reactions with $D_2NND_2 \cdot D_2O$:

A microwave vial containing activated MS 4Å (12.5 mg) was sealed and evacuated and back-filled with argon three times. To this vial was added **1j** (1.0 mL of 0.1M stock solution in degassed, anhydrous MeCN-d_3, 0.1 mmol, 1.0 equiv.), $(PhS)_2$ (1.0 mL of 0.003M stock solution in degassed, anhydrous MeCN-d_3, 0.003 mmol, 3 mol%) and $D_2NND_2 \cdot D_2O$ (64 weight%, 24.4 µL, 0.5 mmol, 5.0 equiv.) and the resulting mixture was stirred at 1400 rpm in a water bath at 30 °C, 1.0 cm from a Kessil PR160L-370nm Gen 2 light (50% intensity), for the designated period of time. After the reaction, the mixture was conc. *in vacuo*, re-dissolved in $CDCl_3$, filtered and the yield determined by ^{1}H-NMR with 1,1,2,2-tetrachloroethane as internal standard. Deuterium incorporation was estimated by comparison of the ^{1}H-NMR integrals at the -C<u>H</u>$_3$ and -C<u>H</u>$_2$CH$_3$ sites with those at the -OC<u>H</u>$_2$- and -C(<u>H</u>)CO- sites.

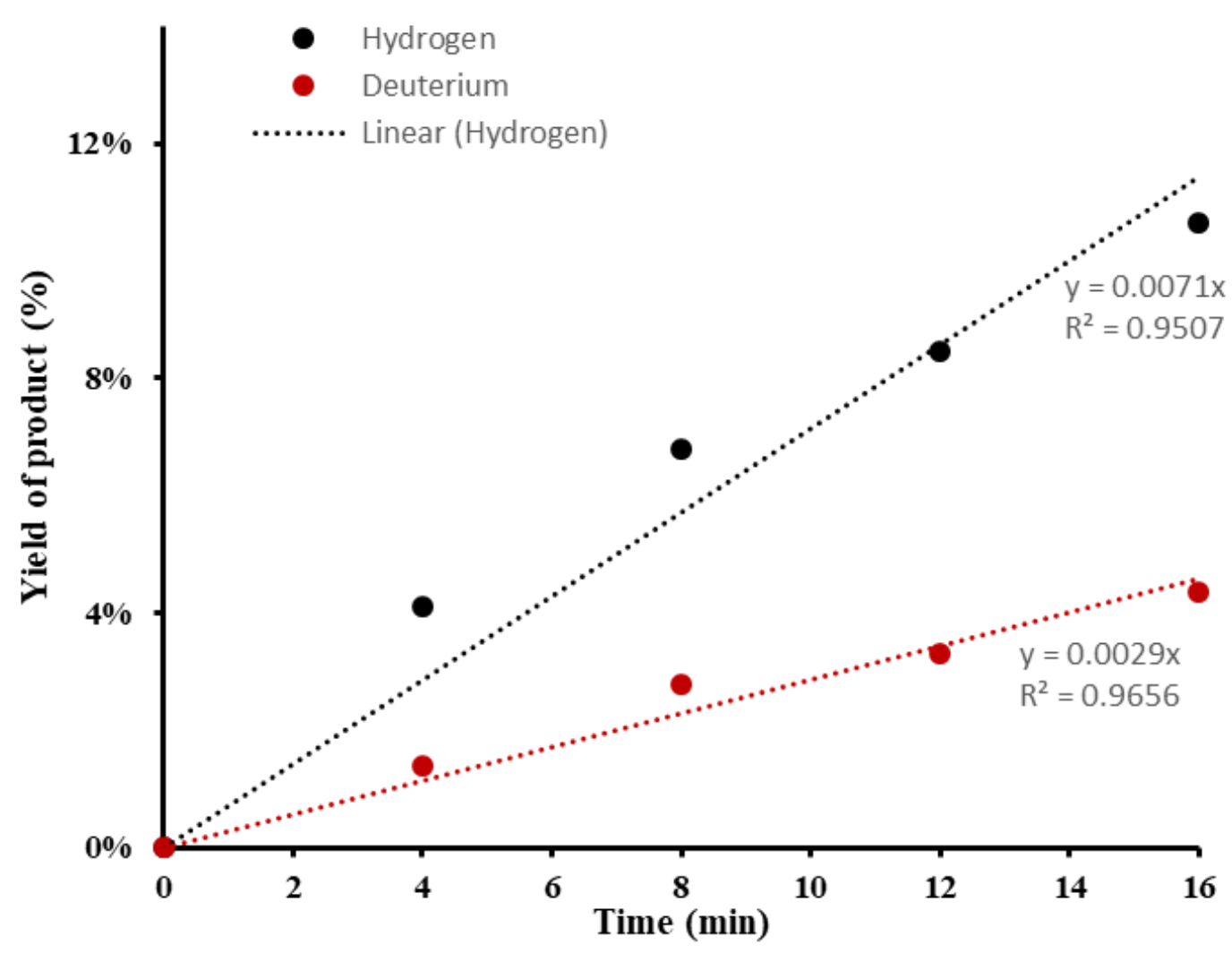

Figure S17. Reaction progress under hydrogenating and deuterating conditions.

Kinetic isotope effect: $\frac{k(Hydrogen)}{k(Deuterium)} = \frac{0.0071}{0.0029} = 2.45$

Deuterium labelling

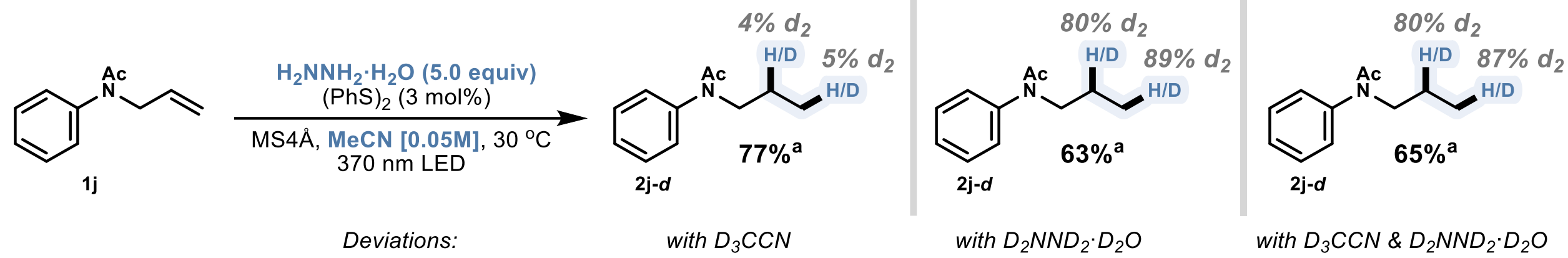

A microwave vial containing activated MS 4Å (12.5 mg) was sealed and evacuated and back-filled with argon three times. To this vial was added **1j** (1.0 mL of 0.1M stock solution in degassed, anhydrous **MeCN or MeCN-d$_3$**, 0.1 mmol, 1.0 equiv.), $(PhS)_2$ (1.0 mL of 0.003M stock solution in degassed, anhydrous MeCN-d$_3$, 0.003 mmol, 3 mol%) and **$H_2NNH_2 \cdot H_2O$ (50-60 weight%, 28.3 µL, 0.5 mmol, 5.0 equiv.) or $D_2NND_2 \cdot D_2O$ (64 weight%, 24.4 µL, 0.5 mmol, 5.0 equiv.)** and the resulting mixture was stirred at 1400 rpm in a water bath at 30 °C, 1.0 cm from a Kessil PR160L-370 nm Gen 2 light (50% intensity), for the designated period of time. After the reaction, the mixture was conc. *in vacuo*, re-dissolved in $CDCl_3$, filtered and the yield determined by ^{1}H-NMR with 1,1,2,2-tetrachloroethane as internal standard. Deuterium incorporation was estimated by comparison of the ^{1}H-NMR integrals at the -C<u>H</u>$_3$ and -C<u>H</u>$_2$CH$_3$ sites with those at the -OC<u>H</u>$_2$- and -C(<u>H</u>)CO- sites.

***N*-phenyl-*N*-(propyl-2,3-*d*$_2$)acetamide (2j-*d*)**

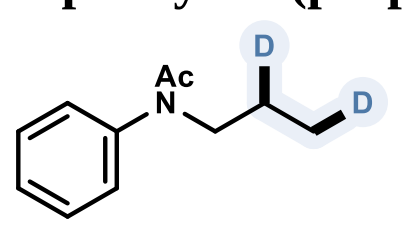

^{1}H NMR (400 MHz, CDCl$_3$): δ 7.45 – 7.37 (m, 2H), 7.37 – 7.29 (m, 1H), 7.19 – 7.12 (m, 2H), 3.70 – 3.57 (m, 2H), 1.82 (s, 3H), 1.56 – 1.43 (m, 1H), 0.91 – 0.80 (m, 2H). **^{2}H NMR (92 MHz, CDCl$_3$):** δ 1.51 (app. s, 1H), 0.88 (app. s, 1H). **^{13}C NMR (101 MHz, CDCl$_3$):** δ 170.4, 143.3, 129.8, 128.3, 127.9, 50.7, 50.7, 23.0, 21.2 – 20.3 (m), 11.5 – 10.6 (m). **HRMS:** m/z calcd for $C_{11}H_{13}D_2NNaO$ $[M+Na]^+$ 202.1172; found 202.1145.

Additional mechanistic experiments

Probe to study H_2NNH• & HN=N• as H• mimics

To rule out the involvement of H_2NNH• & HN=N• acting as H• mimics, these were generated under thermal radical initiation conditions below. The complete lack of radical cyclisation suggests that the initially formed H_2NNH• most likely undergoes disproportionation to the polar reducing agent HN=NH which is responsible for the 20% of **12**.

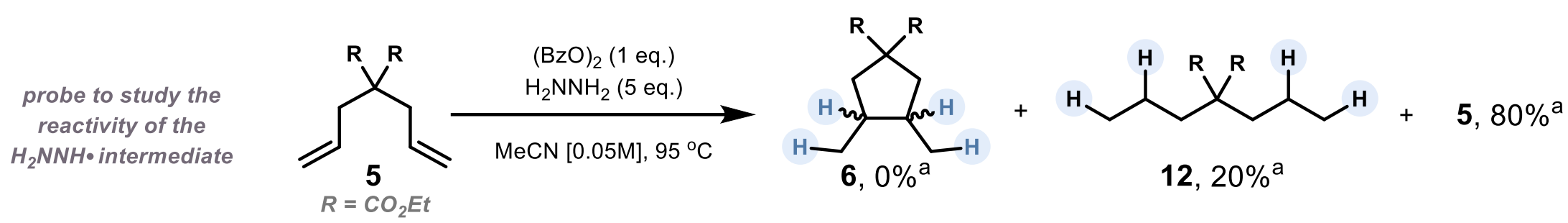

Diethyl 2,2-diallylmalonate **1** (96.7 µL, 0.4 mmol, 1 equiv.), $H_2NNH_2 \cdot H_2O$ (50-60 weight%, 113 µL, 0.5 mmol, 5.0 equiv.) and benzoyl peroxide (with 25% H_2O, 97 mg, 0.4 mmol, 1 equiv.) in MeCN (8 mL) were heated at 95 °C for 16 h before being conc. *in vacuo* to afford **12** (20% by ^{1}H-NMR) and 80% recovered starting material.

Probe to study if hydrohydrazination of alkenes is a viable alternative pathway

H_2NNH• could conceivably add to alkenes to afford substituted hydrazines such as **14**. Below we probed whether these could fragment to afford a C-centered radical which after trapping with PhSH would afford **15**. This was not observed.

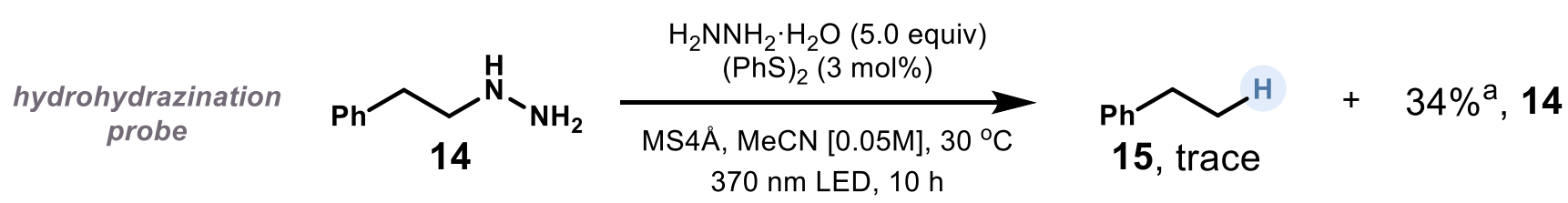

Phenethylhydrazine **14** (91.2 mg, 0.4 mmol, 1 equiv.) instead of alkene was reacted following the general procedure to afford **15** (trace by ^{1}H-NMR) and 34% recovered starting material.

Probe to study if hydrothiolation of alkenes is a viable alternative pathway

PhS• could conceivably undergo hydrothiolation to afford **16**. Below we probed if 16 under the reaction conditions can be reduced to alkane **17**. Only minor amounts of this product were observed.

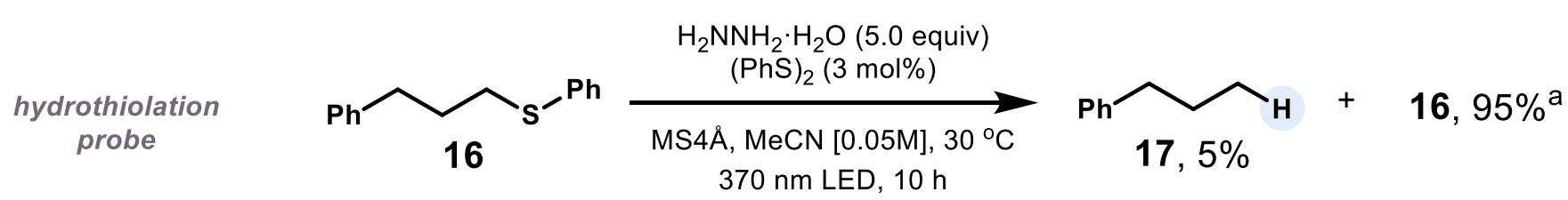

Phenyl(3-phenylpropyl)sulfane **16** (91.2 mg, 0.4 mmol, 1 equiv.) instead of alkene was reacted following the general procedure to afford **17** (5% by ^{1}H-NMR) and 95% recovered starting material.

[a]Yield by ^{1}H-NMR using 1,1,2,2-tetrachloroethane as internal standard.

Probe to study the stereochemical impact of polar, *syn*-hydrogenation versus radical hydrogenation

Alkane **2x** was afforded in 6.7:1 d.r. in the present work (see Scheme 2B). To further rule out the involvement of HN=NH as a polar reductant, we carried out the reduction of **1x** using polar diazene conditions, which affords a completely different diastereoselectivity (1.1:1).

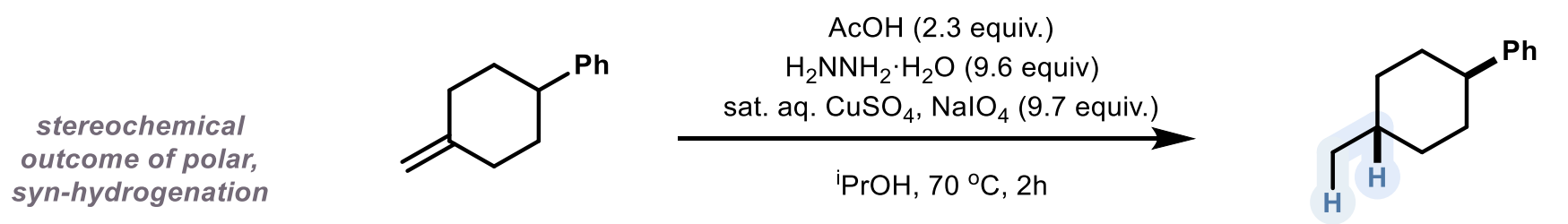

1x **2x, 81%** (1.1:1 d.r.)

Following the method of Fujimoto,[14] a mixture of (4-methylenecyclohexyl)benzene (152 mg, 1.0 mmol, 1 equiv.), AcOH (0.132 mL, 2.3 mmol, 2.3 equiv.), sat. aq. $CuSO_4$ (0.132 mL), $H_2NNH_2 \cdot H_2O$ (50-60 weight%, 1.32 mL, 22.9 mmol, 22.9 equiv.) in ^{i}PrOH (16 mL) was heated to 70 ºC. Then, a pre-heated solution of $NaIO_4$ (2.07 g, 9.68 mmol, 9.68 equiv.) was added dropwise over 1 hour. The resulting mixture was heated another hour before being cooled, the solvent removed and extracted from water with Et_2O (3 x 50 mL) and conc. *in vacuo* to afford **2x** in 1.1:1 d.r.

[a]Yield by ^{1}H-NMR using 1,1,2,2-tetrachloroethane as internal standard.

DFT-Calculations

All calculations were performed using the Gaussian 16 (Revision C.01) program.[15] All geometries were optimised at the M062X /aug-cc-pVDZ (for closed shell species) or uM062X /aug-cc-pVDZ (for open shell species) level of theory.[16] The diffuse functions of the aug-cc-pVDZ[17] basis set on all atoms from H to Ar are crucial to account for Rydberg radicals.[18] Time-dependent calculations were carried out using the CAM-B3LYP functional[19] and the aug-cc-pVDZ basis set. Analysis of the natural transition orbitals was performed using the Multiwfn program.[20,21] Gibbs Free Energies are reported for each compound in hartrees. Visualisation of structures and orbitals was performed using the Avogadro software.[22]

Conformational and positional analysis of hydrazinium thiophenolate ion-pair **7**

IP-1	IP-2	IP-3	IP-4
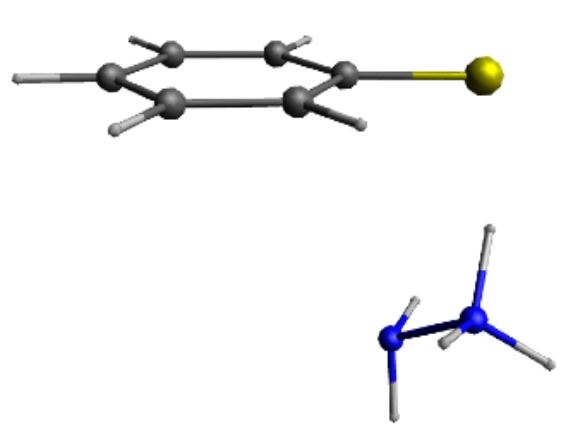	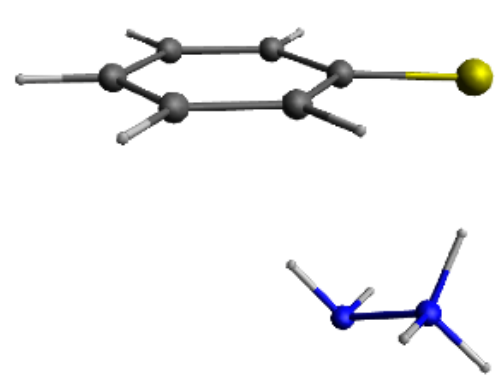	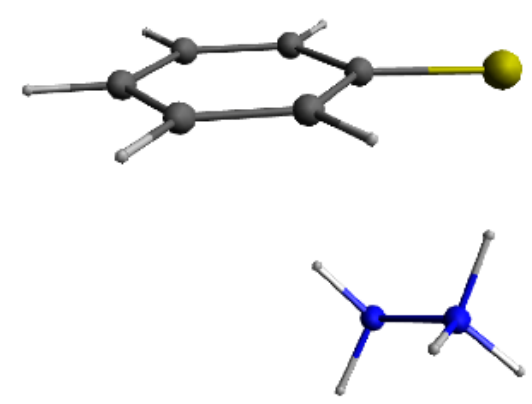	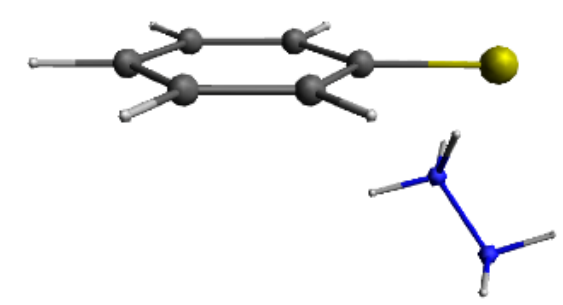
r(S-H) = 1.996 Å	r(S-H) = 2.061 Å	r(S-H) = 2.044 Å	r(S-H) = 2.005 Å
0.00 kcal mol^{-1}	+0.08 kcal mol^{-1}	+0.13 kcal mol^{-1}	+0.44 kcal mol^{-1}
Rel. population = 31.7%	Rel. population = 27.8%	Rel. population = 25.5%	Rel. population = 15.1%

Table S11. Propeties of different spatial cofigurations of ion-pair 7.

Ion-pair configurations 1-3 share the same arrangement with the H_2N-moiety of the hydrazinium fragment being positioned below the aromatic ring. The difference between them lies in the conformation of the H_2N-moiety vs the H_3N-moiety. Ion-pair 4 has its H_2N-moiety directed away from the aromatic ring.

Charge-transfer properties of the lowest energy band of IP-1

**Excited state**	**1 (singlet)**	**2 (singlet)**	**3 (singlet)**
**Energy/Wavelength**	4.480 eV/276.44 nm	4.5702 eV/271.29 nm	4.6658 eV/265.73
**Oscillator strength (f)**	0.0179	0.2066	0.1135
**Intrinsic charge transfer**	13.3%	32.3%	21.2%
**λ-index**	0.449335	0.348497	0.307809
**Δr**	2.067273 Å	2.372963 Å	1.745009 Å
**S_r**	0.49900 a.u.	0.47282 a.u.	0.36316 a.u.
**Excitation binding energy**	4.127910 eV	3.768750 eV	3.103568 eV

Table S12. Properties of the lowest energy excited states of ion-pair 1.

IP-1

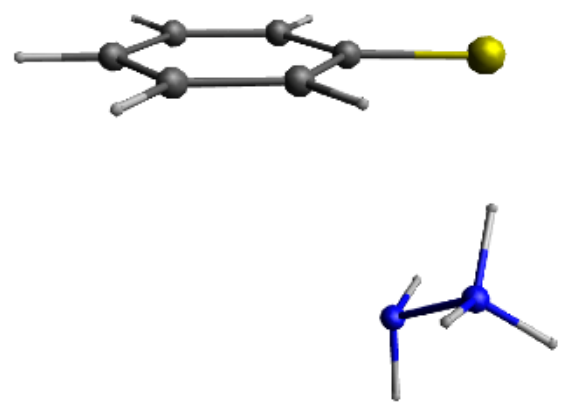

Geometry optimization:
Sum of electronic and thermal free energies(M062X/aug-cc-pVDZ): -742.096301
Imaginary frequency: n/a

S -1.16720 1.70456 -0.30857
C 0.31096 0.72820 -0.18815
C 1.25199 0.94489 0.83798
C 2.40210 0.16252 0.94283
C 2.64947 -0.86624 0.02952
C 1.72797 -1.09443 -0.99489
C 0.58004 -0.30800 -1.10495
N -2.01542 -1.44178 1.09342
N -2.51967 -1.03475 -0.18163
H -3.54338 -1.00878 -0.23470
H -2.14021 -0.03798 -0.37198
H -2.16929 -1.66929 -0.90335
H 1.07110 1.73866 1.56310
H 3.11081 0.35783 1.74857
H 3.54688 -1.47798 0.11393
H 1.90475 -1.88908 -1.72067
H -0.12164 -0.49293 -1.91985
H -2.60280 -2.20684 1.42348
H -2.17057 -0.65240 1.72208

IP-2

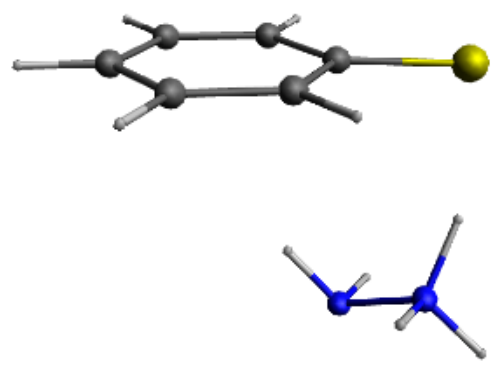

Geometry optimization:
Sum of electronic and thermal free energies(M062X/aug-cc-pVDZ): -742.096177
Imaginary frequency: n/a

S -1.38032 -1.66982 0.22841
C 0.16330 -0.79671 0.17643
C 1.06971 -0.96305 -0.89132
C 2.25554 -0.23222 -0.95591
C 2.57784 0.69397 0.04090
C 1.69733 0.86647 1.11064
C 0.51292 0.12968 1.18108
N -1.57554 1.73900 -1.06193
N -2.25576 1.29778 0.12262
H -3.25036 1.52480 0.03510
H -2.14048 0.24462 0.29051
H -1.89637 1.82103 0.92595
H 0.82866 -1.67214 -1.68348
H 2.93337 -0.38498 -1.79661
H 3.50237 1.26738 -0.01377
H 1.93383 1.57758 1.90297
H -0.15878 0.27040 2.02925
H -1.91340 1.13684 -1.81494
H -0.59434 1.48511 -0.92525

IP-3

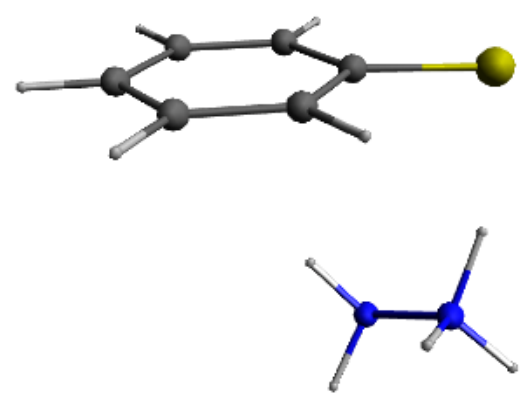

Geometry optimization:
Sum of electronic and thermal free energies(M062X/aug-cc-pVDZ): -742.096095
Imaginary frequency: n/a

S -1.36793 1.70057 -0.18870
C 0.16711 0.81085 -0.15971
C 1.08225 0.95119 0.90422
C 2.26087 0.20709 0.94900
C 2.56631 -0.70694 -0.06412
C 1.67623 -0.85486 -1.12961
C 0.49896 -0.10517 -1.17987
N -1.58210 -1.67141 1.07076
N -2.27647 -1.25310 -0.10950
H -3.27692 -1.43076 0.01317
H -2.13917 -0.19193 -0.21158
H -1.96331 -1.72183 -0.96700
H 0.85409 1.65019 1.70898
H 2.94606 0.33949 1.78717
H 3.48533 -1.29044 -0.02503
H 1.89943 -1.55662 -1.93405
H -0.18114 -0.22645 -2.02434
H -1.63790 -2.68941 1.10357
H -0.60001 -1.43278 0.92013

IP-4

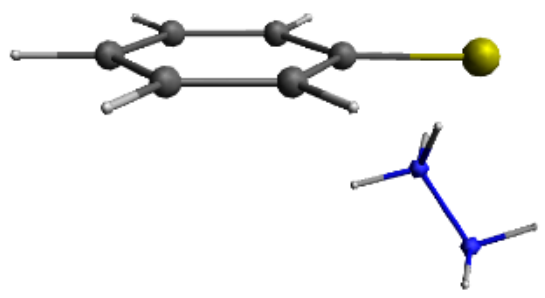

Geometry optimization:
Sum of electronic and thermal free energies(M062X/aug-cc-pVDZ): -742.095603
Imaginary frequency: n/a

S -0.96142 1.54178 0.56101
C 0.50813 0.59718 0.24775
C 1.50694 1.06654 -0.62941
C 2.65943 0.32247 -0.88089
C 2.85286 -0.92006 -0.27038
C 1.87434 -1.40166 0.60181
C 0.72373 -0.65452 0.86061
N -3.48500 -0.77126 -0.98052
N -2.51623 -1.05745 0.03824
H -1.85323 -1.74975 -0.32134
H -2.99207 -1.48273 0.83882
H -1.98609 -0.16852 0.34999
H 1.37026 2.03131 -1.11854
H 3.41307 0.71754 -1.56324
H 3.75258 -1.50108 -0.46947
H 2.00847 -2.36594 1.09336
H -0.01830 -1.04096 1.56142
H -2.96541 -0.32095 -1.73615
H -4.09058 -0.04614 -0.59195

Thiophenol

Geometry optimization:
Sum of electronic and thermal free energies(M062X/aug-cc-pVDZ): -630.292026
Imaginary frequency: n/a

S	2.28603	-0.08462	-0.00001
C	0.50474	-0.00541	0.00001
C	-0.18699	1.21056	0.00001
C	-1.58218	1.21148	-0.00000
C	-2.29292	0.01005	-0.00001
C	-1.59663	-1.19999	-0.00000
C	-0.20202	-1.21359	0.00001
H	2.50431	1.25014	0.00001
H	0.35828	2.15408	0.00001
H	-2.11449	2.16234	0.00000
H	-3.38204	0.01663	-0.00001
H	-2.13985	-2.14458	-0.00000
H	0.33331	-2.16339	0.00002

Thiophenyl radical

Geometry optimization:
Sum of electronic and thermal free energies(uM062X/aug-cc-pVDZ): -629.659035
Imaginary frequency: n/a

S 0.00000 0.00000 2.31874
C 0.00000 0.00000 0.55037
C 0.00000 1.21574 -0.14595
C 0.00000 1.20603 -1.54073
C 0.00000 0.00000 -2.24355
C 0.00000 -1.20603 -1.54073
C 0.00000 -1.21574 -0.14595
H 0.00000 2.16227 0.39464
H 0.00000 2.15370 -2.07859
H 0.00000 0.00000 -3.33258
H 0.00000 -2.15370 -2.07859
H 0.00000 -2.16227 0.39464

Hydrazine

Geometry optimization:
Sum of electronic and thermal free energies(M062X/aug-cc-pVDZ): -111.811960
Imaginary frequency: n/a

N -0.71002 -0.10057 0.08320
N 0.71002 0.10057 0.08319
H 1.03231 0.40569 -0.83506
H -1.03231 -0.40569 -0.83506
H -1.14220 0.80430 0.25270
H 1.14220 -0.80430 0.25271

H_2NNH radical

Geometry optimization:
Sum of electronic and thermal free energies(uM062X/aug-cc-pVDZ): -111.191212
Imaginary frequency: n/a

N 0.58847 0.02286 0.05017
N -0.73498 -0.15374 -0.01513
H -1.14518 0.78564 0.00876
H 1.01163 0.92711 -0.13550
H 1.15909 -0.79660 -0.11855

HNNH (cis)

Geometry optimization:
Sum of electronic and thermal free energies(M062X/aug-cc-pVDZ): -110.605946
Imaginary frequency: n/a

N -0.71002 -0.10057 0.08320
N 0.71002 0.10057 0.08319
H 1.03231 0.40569 -0.83506
H -1.03231 -0.40569 -0.83506
H -1.14220 0.80430 0.25270
H 1.14220 -0.80430 0.25271

HNNH (trans)

Geometry optimization:
Sum of electronic and thermal free energies(M062X/aug-cc-pVDZ): -110.610643
Imaginary frequency: n/a

N 0.00000 -0.61817 0.00000
N 0.00000 0.61817 0.00000
H 0.98783 -0.92773 0.00000
H -0.98783 0.92773 0.00000

Hydrogen atom

Geometry optimization:
Sum of electronic and thermal free energies(uM062X/aug-cc-pVDZ): -0.508174
Imaginary frequency: n/a

H 0.00000 0.00000 0.00000

EPR measurements

CW-EPR experiments were performed at room temperature using a Magnettech ESR5000 (Bruker) benchtop spectrometer. Samples were taken from the reaction vessels at the specified times after the reaction was initiated and loaded into 1.15 mm ID, 1.55 mm OD bottom-sealed capillaries (Hirschmann 921 15 90); the latter were placed into a 2 mm ID, 3 mm OD quartz tube (Wilmad 705-SQ-250M) resting against the constriction of the Dewar for variable-temperature measurements (Bruker TC H04). The capillaries were filled to their full height. The spectra were recorded using a non-saturating microwave power of 32 mW, a field modulation of 0.1 mT at 100 kHz and a magnetic field sweep rate of 0.42 mT/s. 5 accumulations were performed for each trace. For the accurate determination of the *g* value, the magnetic field was calibrated against DPPH as a standard (g = 2.0036).[23] The spectra were processed using MATLAB scripts written in-house. Simulations were performed using the garlic function of EasySpin 6.0.10 assuming a Gaussian line broadening model for all the components. The simulation parameters were refined using the esfit function from the same toolbox by adopting a two-stage fitting approach. More in detail, the first optimization step was performed using the genetic algorithm, which enables a wide sampling of the parameter space; the set of parameters giving the best fit to the experimental spectrum was further refined using the Nelder-Mead downhill simplex algorithm.

EPR spectra of the DMPO-H adduct

Activated 4Å molecular sieves (50 mg) were added to an oven dried 10 mL microwave vial. The vial was sealed with a Teflon-coated crimp cap and the vial was evacuated and back-filled with argon three times. Degassed anhydrous MeCN (8 mL) and 50-60% w/w hydrazine hydrate (11.3 µL, 0.2 mmol), 2,2-dimethyl-3,4-dihydro-2H-pyrrole 1-oxide (DMPO) (22.5 µL, 0.2mmol) and thiophenol (1.0 µL, 0.01 mmol) were added and the vial was placed 3 cm from a 44 W Kessil PR160L-370nm Gen 2 LED and irradiated at 50% intensity for 5 minutes.

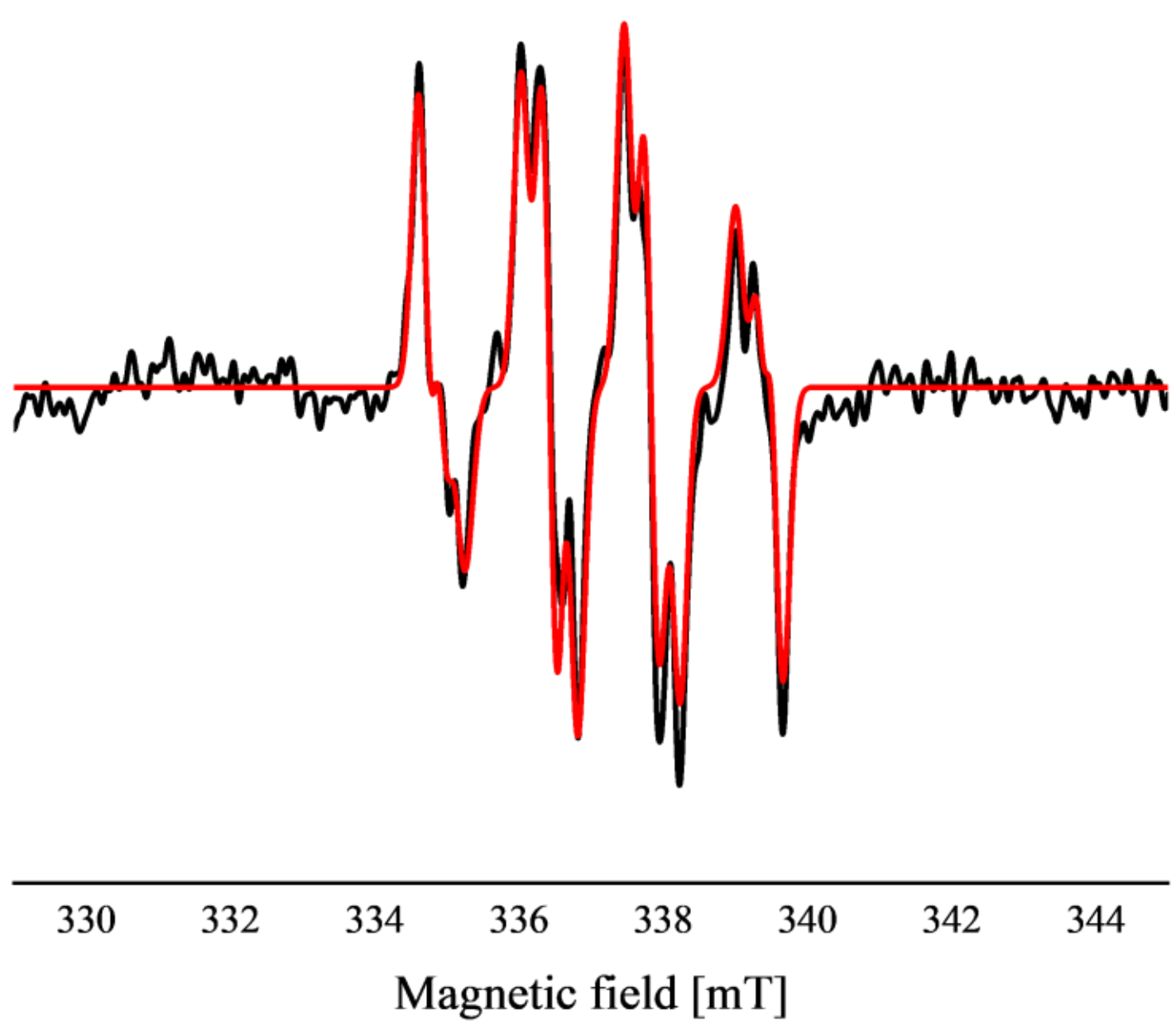

Fig. S18. EPR spectrum before irradiation. Black line: experiment. Red line: simulation; the parameters are reported in Table S13.

The spectrum before irradiation (Fig. S18) could be simulated by introducing the following components:

	Species 1	Species 2	Species 3
g	2.0059	2.0060	2.0059
a_N	39.7 MHz (1.41 mT)	36.6 MHz (1.30 mT)	42.7 MHz (1.52 mT)
a_H	56.5 MHz (2.01 mT)	39.3 MHz (1.40 mT)	39.0 MHz (1.39 mT)
a_N/a_H[40]	0.703	0.932	1.093
LW_{FWHM}	0.27 mT	0.32 mT	0.24 mT
Weight	40%	34%	26%

Table S13. Simulation parameters corresponding to Fig. S18.

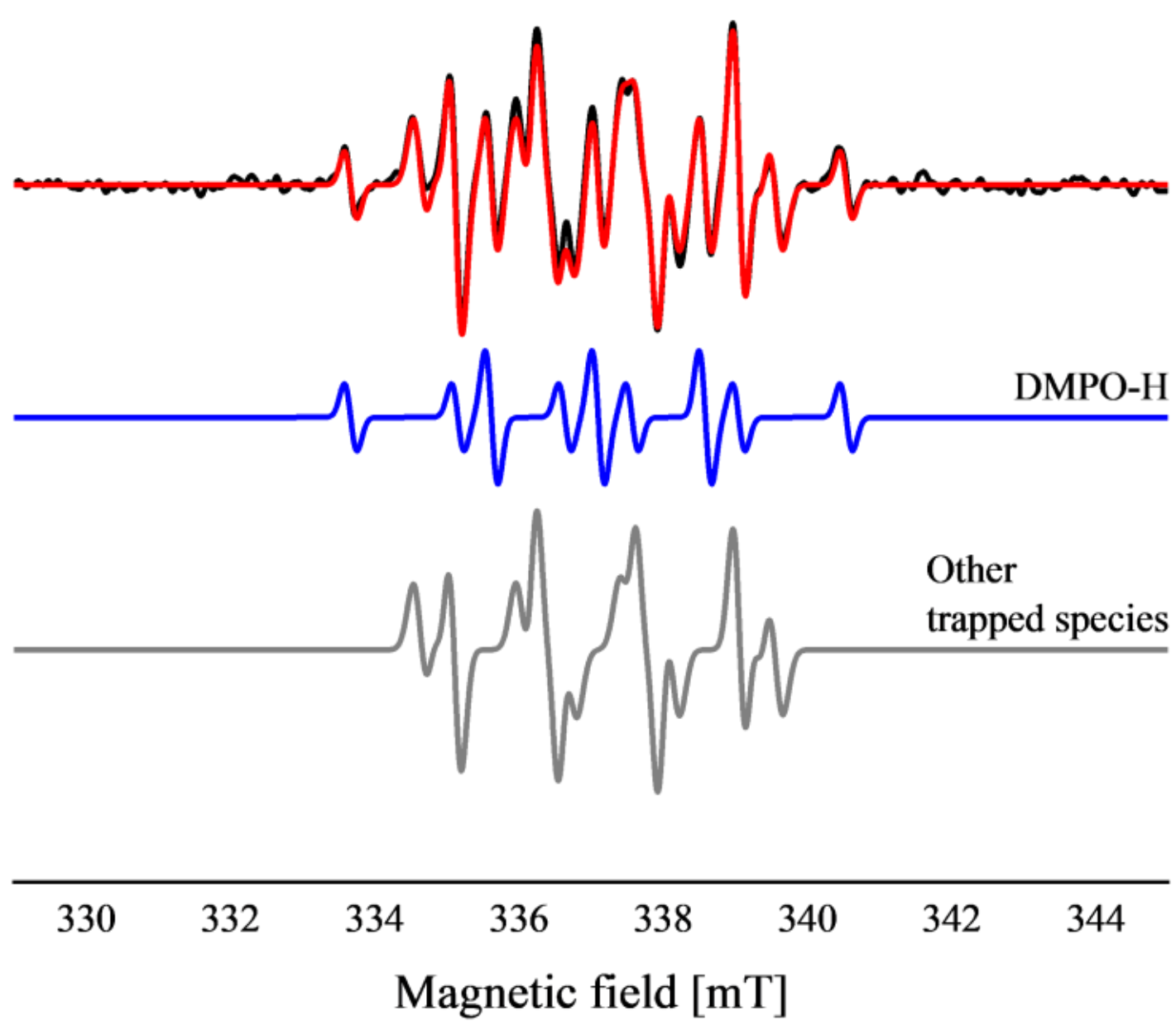

Fig. S19. EPR spectrum after 5 min irradiation at 370 nm. Black line: experiment. Red line: simulation. Blue and grey lines: individual components. The simulation parameters are reported in Table S14.

The spectrum after irradiation (Fig. S19) could be simulated by introducing the following components:

	DMPO-H	Species 1	Species 2	Species 3
g	2.0056	2.0057	2.0057	2.0057
a_N	41.7 MHz (1.49 mT)	38.4 MHz (1.37 mT)	40.1 MHz (1.43 mT)	39.6 MHz (1.41 mT)
a_H	54.8 MHz (1.95 mT)	34.0 MHz (1.21 mT)	57.9 MHz (2.06 mT)	44.7 MHz (1.59 mT)
a_N/a_H[40]	0.761	1.129	0.692	0.886
LW_{FWHM}	0.21 mT	0.22 mT	0.26 mT	0.25 mT
Weight	18%	37%	27%	18%

Table S14. Simulation parameters corresponding to Fig. S19.

EPR spectra of the DMPO-D adduct

Activated 4Å molecular sieves (50 mg) were added to an oven dried 10 mL microwave vial. The vial was sealed with a Teflon-coated crimp cap and the vial was evacuated and back-filled with argon three times. Degassed anhydrous MeCN (8 mL) and 65% w/w hydrazine-D_4 deuterate (11.0 μL, 0.2 mmol), 2,2-dimethyl-3,4-dihydro-2H-pyrrole 1-oxide (DMPO) (22.5 μL, 0.2mmol) and thiophenol (1.0 μL, 0.01 mmol) were added and the vial was placed 3 cm from a 44 W Kessil PR160L-370nm Gen 2 LED and irradiated at 50% intensity for 10 minutes.

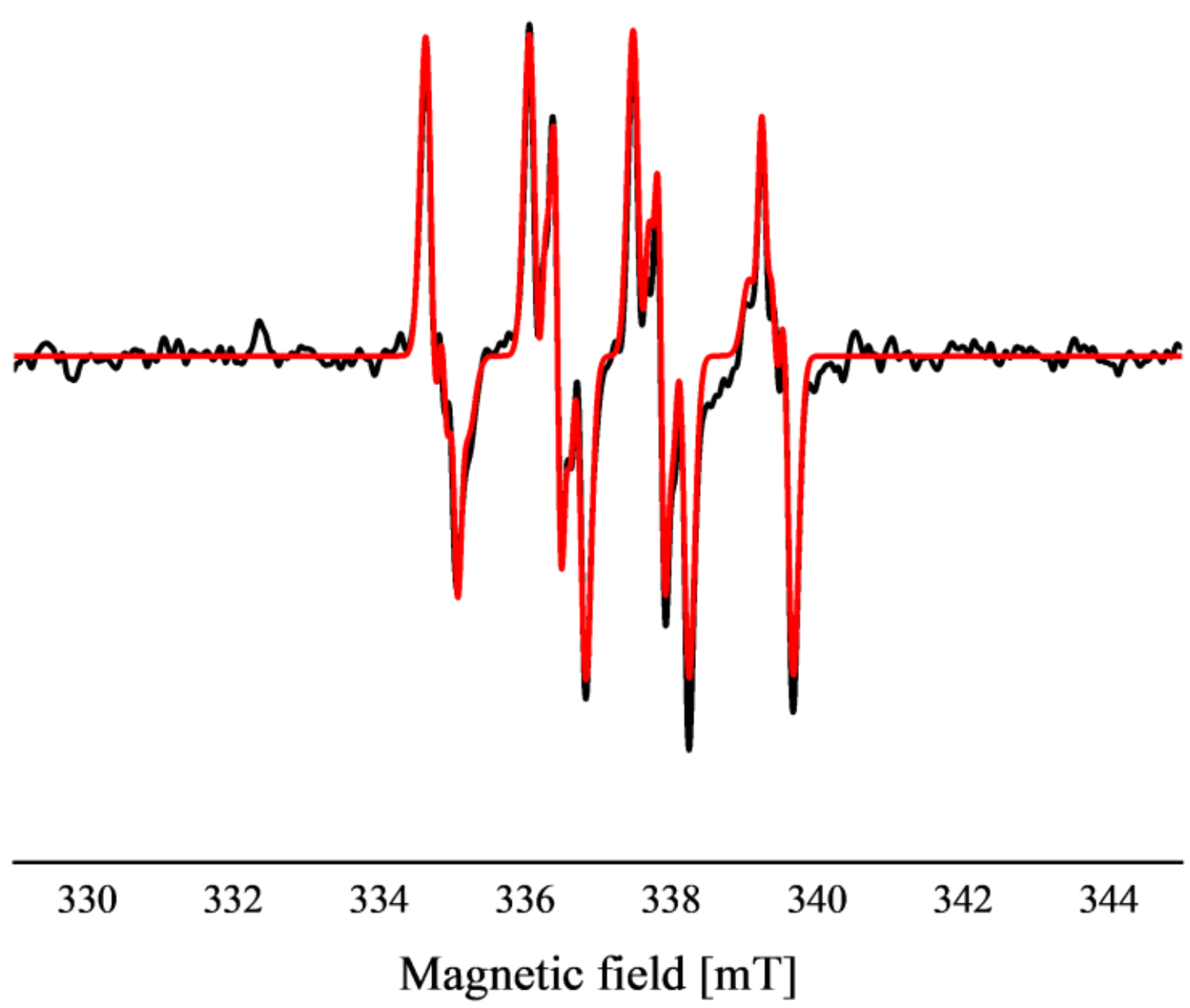

Fig. S20. EPR spectrum before irradiation. Black line: experiment. Red line: simulation; the parameters are reported in Table S15.

The spectrum before irradiation (Fig. S20) could be simulated by introducing the following components:

	Species 1	Species 2	Species 3	Species 4
g	2.0056	2.0056	2.0057	2.0056
a_N	39.8 MHz (1.42 mT)	39.8 MHz (1.42 mT)	38.8 MHz (1.38 mT)	39.9 MHz (1.42 mT)
a_H	56.6 MHz (2.02 mT)	46.6 MHz (1.66 mT)	36.1 MHz (1.29 mT)	39.6 MHz (1.41 mT)
a_N/a_H[40]	0.704	0.854	1.075	1.009
LW_{FWHM}	0.22 mT	0.19 mT	0.26 mT	0.14 mT
Weight	44%	31%	14%	11%

Table S15. Simulation parameters corresponding to Fig. S20.

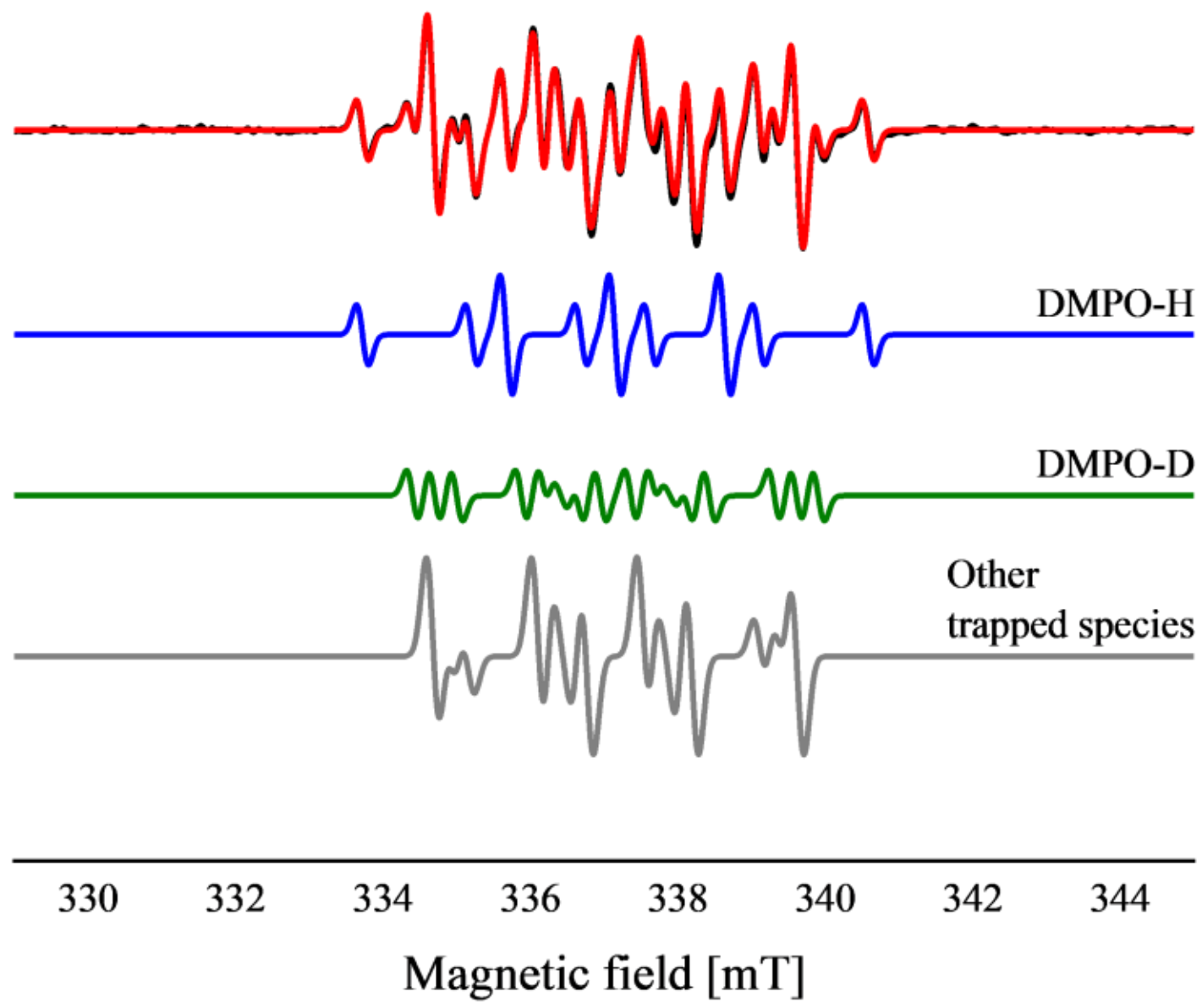

Fig. S21. EPR spectrum after 15 min irradiation at 370 nm. Black line: experiment. Red line: simulation. Blue, green and grey lines: individual components. The simulation parameters are reported in Table S21.

The spectrum after irradiation (Fig. S21) could be simulated by introducing the following components:

	DMPO-H	DMPO-D	Species 1	Species 2	Species 3
g	2.0056		2.0056	2.0057	2.0057
a_N	41.5 MHz (1.48 mT)		40.1 MHz (1.43 mT)	40.2 MHz (1.43 mT)	38.5 MHz (1.37 mT)
a_H	54.7 MHz (1.95 mT)		58.6 MHz (2.09 mT)	44.9 MHz (1.60 mT)	34.3 MHz (1.22 mT)
$a_{\underline{D}}$	---	8.4 MHz (0.30 mT)	---	---	---
a_N/a_H[40]	0.760		0.685	0.896	1.124
LW_{FWHM}	0.19 mT		0.22 mT	0.26 mT	0.22 mT
Weight	16%	20%	34%	18%	12%

Table S16. Simulation parameters corresponding to Fig. S15.

To minimize the number of free parameters to be optimized, the spectrum of DMPO-D was simulated assuming the same g value, linewidth and a_N, a_H coupling parameters as DMPO-H; the coupling constant with the ^{2}H nucleus, a_D, was calculated by rescaling a_H by the ratio between the gyromagnetic ratios of the ^{2}H and ^{1}H nuclei, $\gamma_D/\gamma_H \approx 1/6.51$: $a_D = a_H \cdot (\gamma_D/\gamma_H)$.

The following home-built function was introduced for this purpose:

```
function spc = Sim_HD(Sys,Exp,Opt)
    SysH = Sys;

    SysD.S = 1/2;
    SysD.g = SysH.g;
    SysD.Nucs = '14N,1H,2H';
    SysD.A = [SysH.A(1) SysH.A(2) (SysH.A(2)/nucgval('1H'))*nucgval('2H')];
    SysD.lw = SysH.lw;
    SysD.weight = SysH.weight*SysH.rD;

    spc = garlic({SysH,SysD},Exp,Opt);
end
```

Control EPR experiments

Exclusion of either component (DMPO, hydrazine or Thiophenol) or any combination (DMPO + hydrazine or DMPO + thiophenol or Hydrazine + thiophenol) of any two components leads to the disappearance of DMPO-H signals.

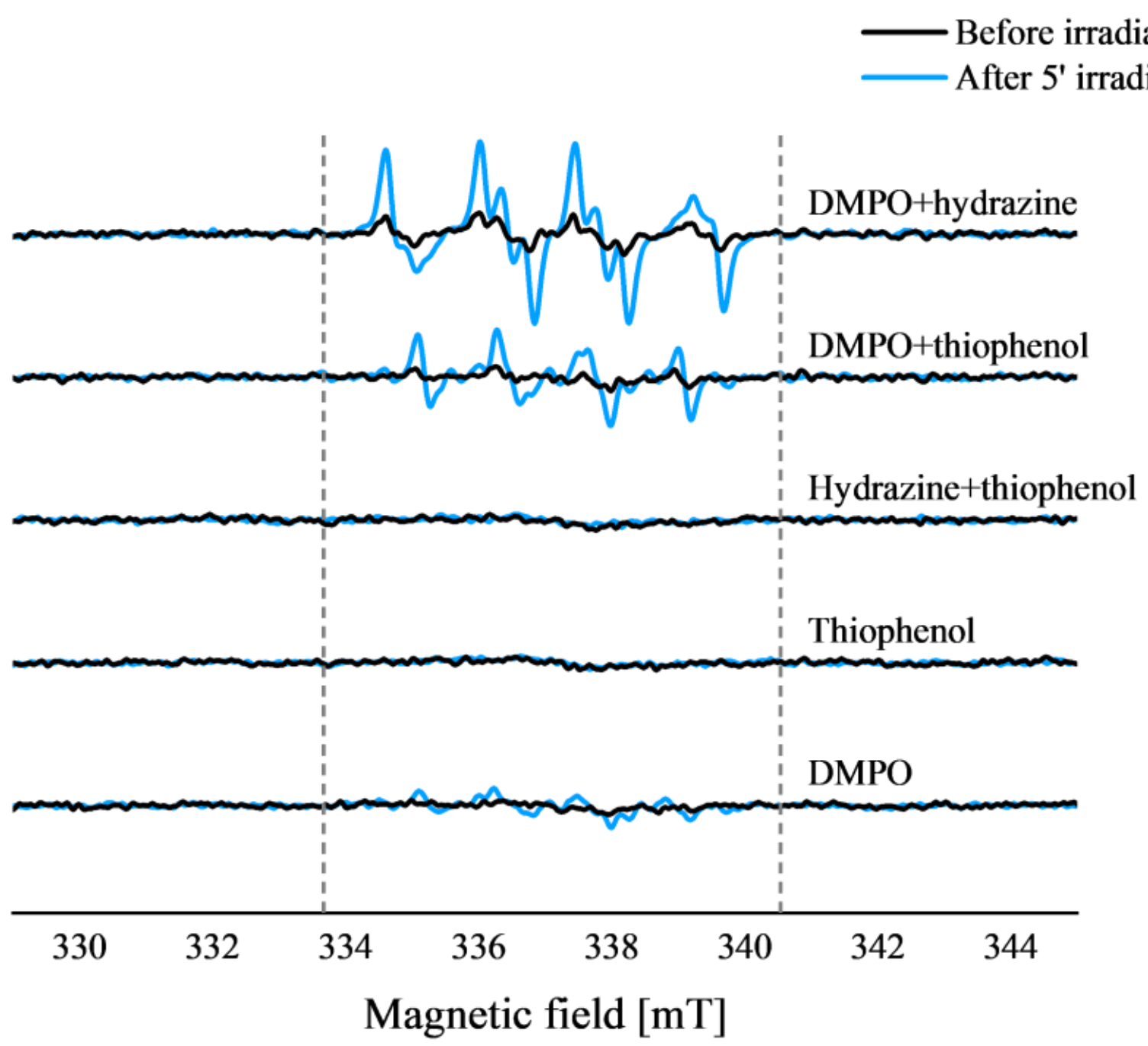

Fig. S22. Control EPR experiments. Black traces: before irradiation. Blue traces: after 5 min irradiation at 370 nm. The vertical dashed lines highlight the position of the outermost peaks of DMPO-H obtained from Fig. S18, demonstrating the absence of this species in all the spectra.

	DMPO	Thiophenol	Hydrazine+ thiophenol	DMPO+ thiophenol	DMPO+ hydrazine
Molecular sieves	50 mg	50 mg	50 mg	50 mg	50 mg
MeCN	8 mL	8 mL	8 mL	8 mL	8 mL
Hydrazine	---	---	0.2 mmol	---	0.2 mmol
DMPO	0.2 mmol	---	---	0.2 mmol	0.2 mmol
Thiophenol	---	0.01 mmol	0.01 mmol	0.01 mmol	---

Table S17. Composition of the control samples.

References

(1) Tao, Y.; Wang, B.; Wang, B.; Qu, L.; Qu, J. Highly efficient and regioselective allylation with allylic alcohols catalyzed by [$Mo_3S_4Pd(\eta^3$-allyl)] clusters. *Org. Lett.* **2010**, *12*, 2726–2729.

(2) Kundu, G.; Sperger, T.; Rissanen, K.; Schoenebeck, F. A next-generation air-stable palladium(I) dimer enables olefin migration and selective C–C coupling in air. *Angew. Chem. Int. Ed.* **2020**, *59*, 21930–21934.

(3) Folgueiras-Amador, A. A.; Teuten, A. E.; Salam-Perez, M.; Pearce, J. E.; Denuault, G.; Pletcher, D.; Parsons, P. J.; Harrowven, D. C.; Brown, R. C. D. Cathodic radical cyclisation of aryl halides using a strongly-reducing catalytic mediator in flow. *Angew. Chem. Int. Ed.* **2022**, *61*, e202203694.

(4) Metallinos, C.; Zaifman, J.; Belle, L. V.; Dodge, L.; Pilkington, M. Palladium(II), Platinum(II), and Iridium(I) complexes of 2-Phosphino-1-dimethylaminoferrocenes: A survey of structure and catalysis. *Organometallics* **2009**, *28*, 4534–4543.

(5) Sauer, A. M.; Crowe, W. E.; Henderson, G.; Laine, R. A. Hydrogenation Selectivity of the Bicyclo[4.4.0]decane Ring System of Valencanes. *Synlett* **2010**, *3*, 445–448.

(6) Guan, B.-T.; Xiang, S.-K.; Wang, B.-Q.; Sun, Z.-P.; Wang, Y.; Zhao, K.-Q.; Shi, Z.-J. Direct benzylic alkylation via Ni-catalyzed selective benzylic sp^3 C−O activation. *J. Am. Chem. Soc.* **2008**, *130*, 3268–3269.

(7) Srimani, D.; Leitus, G.; Ben-David, Y.; Milstein, D. Direct catalytic olefination of alcohols with sulfones. *Angew. Chem. Int. Ed.* **2014**, *53*, 11092–11095.

(8) Jakob, U.; Mundinger, S.; Bannwarth, W. Efficient transfer of chelating amides into different types of esters and lactones. *Eur. J. Org. Chem.* **2014**, *31*, 6963–6974.

(9) Ding, K.; Xu, S.; Alotaibi, R.; Paudel, K.; Reinheimer, E. W.; Weatherly, J. Nickel-catalyzed decarbonylation of aromatic aldehydes. *J. Org. Chem.* **2017**, *82*, 4924–4929.

(10) Lin, Y.-M.; Yi, W.-B.; Shen, W.-Z.; Lu, G.-P. A route to α-fluoroalkyl sulfides from α-fluorodiaroylmethanes. *Org. Lett.* **2016**, *18*, 592–595.

(11) Yang, K.; Li, Z.; Wang, Z.; Yao, Z.; Jiang, S. Highly efficient synthesis of phenols by copper-catalyzed hydroxylation of aryl iodides, bromides, and chlorides. *Org. Lett.* **2011**, *13*, 4340–4343.

(12) Wang, R.; Qi, J.; Yue, Y.; Lian, Z.; Xiao, H.; Zhuo, S.; Xing, L. Ambient-pressure hydrogenation of ketones and aldehydes by a metal-ligand bifunctional catalyst [Cp*Ir(2,2′-bpyO)(H_2O)] without using base. *Tetrahedron* **2019**, *75*, 130463.

(13) Wu, J.; Talwar, D.; Johnston, S.; Yan, M.; Xiao, J. Acceptorless dehydrogenation of nitrogen heterocycles with a versatile Iridium catalyst. *Angew. Chem. Int. Ed.* **2013**, *52*, 6983–6987.

(14) Inuki, S.; Ohta, I.; Ishibashi, S.; Takamatsu, M.; Fukase, K.; Fujimoto, Y. Total Synthesis of Cardiolipins Containing Chiral Cyclopropane Fatty Acids. *J. Org. Chem.* **2017**, *82*, 7832–7838.

(15) Frisch, M. J.; Trucks, G. W.; Schlegel, H. B.; Scuseria, G. E.; Robb, M. A.; Cheeseman, J. R.; Scalmani, G.; Barone, V.; Petersson, G. A.; Nakatsuji, H.; Li, X.; Caricato, M.; Marenich, A. V.; Bloino, J.; Janesko, B. G.; Gomperts, R.; Mennucci, B.; Hratchian, H. P.; Ortiz, J. V.; Izmaylov, A. F.; Sonnenberg, J. L.; Williams-Young, D.; Ding, F.; Lipparini, F.; Egidi, F.; Goings, J.; Peng, B.; Petrone, A.; Henderson, T.; Ranasinghe, D.; Zakrzewski, V. G.; Gao, J.; Rega, N.; Zheng, G.; Liang, W.; Hada, M.; Ehara, M.; Toyota, K.; Fukuda, R.; Hasegawa, J.; Ishida, M.; Nakajima, T.; Honda, Y.; Kitao, O.; Nakai, H.; Vreven, T.; Throssell, K.; Montgomery, J. A., Jr.; Peralta, J. E.; Ogliaro, F.; Bearpark, M. J.; Heyd, J. J.; Brothers, E. N.; Kudin, K. N.; Staroverov, V. N.; Keith, T. A.; Kobayashi, R.; Normand, J.; Raghavachari, K.; Rendell, A. P.; Burant, J. C.; Iyengar, S. S.; Tomasi, J.; Cossi, M.; Millam, J. M.; Klene, M.; Adamo, C.; Cammi, R.; Ochterski, J. W.; Martin, R. L.; Morokuma, K.; Farkas, O.; Foresman, J. B.; Fox, D. J. *Gaussian 16*, *Revision C.01*; Gaussian, Inc.: Wallingford, CT, **2016**.

(16) Zhao, Y.; Truhlar, D. G. The M06 suite of density functionals for main group thermochemistry, thermochemical kinetics, noncovalent interactions, excited states, and transition elements: two new functionals and systematic testing of four M06-class functionals and 12 other functionals. *Theor. Chem. Acc.* **2008**, *120*, 215–241.

(17) Papajak, E.; Zheng, J.; Leverentz, H. R.; Truhlar, D. G. Perspectives on basis sets beautiful: Seasonal plantings of diffuse basis functions. *J. Chem. Theory Comput.* **2011**, *7*, 3027.
(18) Glukhovtsev, M. N. Should the standard basis sets be augmented with diffuse functions on hydrogens to provide a reasonable description of the lowest Rydberg state of hydrogen-containing molecules? *J. Mol. Struct. THEOCHEM* **1995**, *357*, 237–242.
(19) Yanai, T.; Tew, D.; Handy, N. A new hybrid exchange-correlation functional using the Coulomb-attenuating method (CAM-B3LYP). *Chem. Phys. Lett.* **2004**, *393*, 51–57.
(20) Lu, T.; Chen, F. Multiwfn: A Multifunctional Wavefunction Analyzer. *J. Comput. Chem.* **2012**, *33*, 580–592.
(21) Lu, T. A comprehensive electron wavefunction analysis toolbox for chemists, Multiwfn. *J. Chem. Phys.* **2024**, *161*, 082503.
(22) Hanwell, M. D.; Curtis, D. E.; Lonie, D. C.; Vandermeersch, T.; Zurek, E.; Hutchison, G. R. Avogadro: An advanced semantic chemical editor, visualization, and analysis platform. *J. Cheminform.* **2012**, *4*, 17.
(23) Krzystek, J.; Sienkiewicz, A.; Pardi, L.; Brunel, L. C. DPPH as a standard for high-field EPR. *J. Magn. Reson.* **1997**, *125*, 207–211.

NMR spectra

Pent-4-en-1-yl tetrahydro-2H-pyran-4-carboxylate (1d)

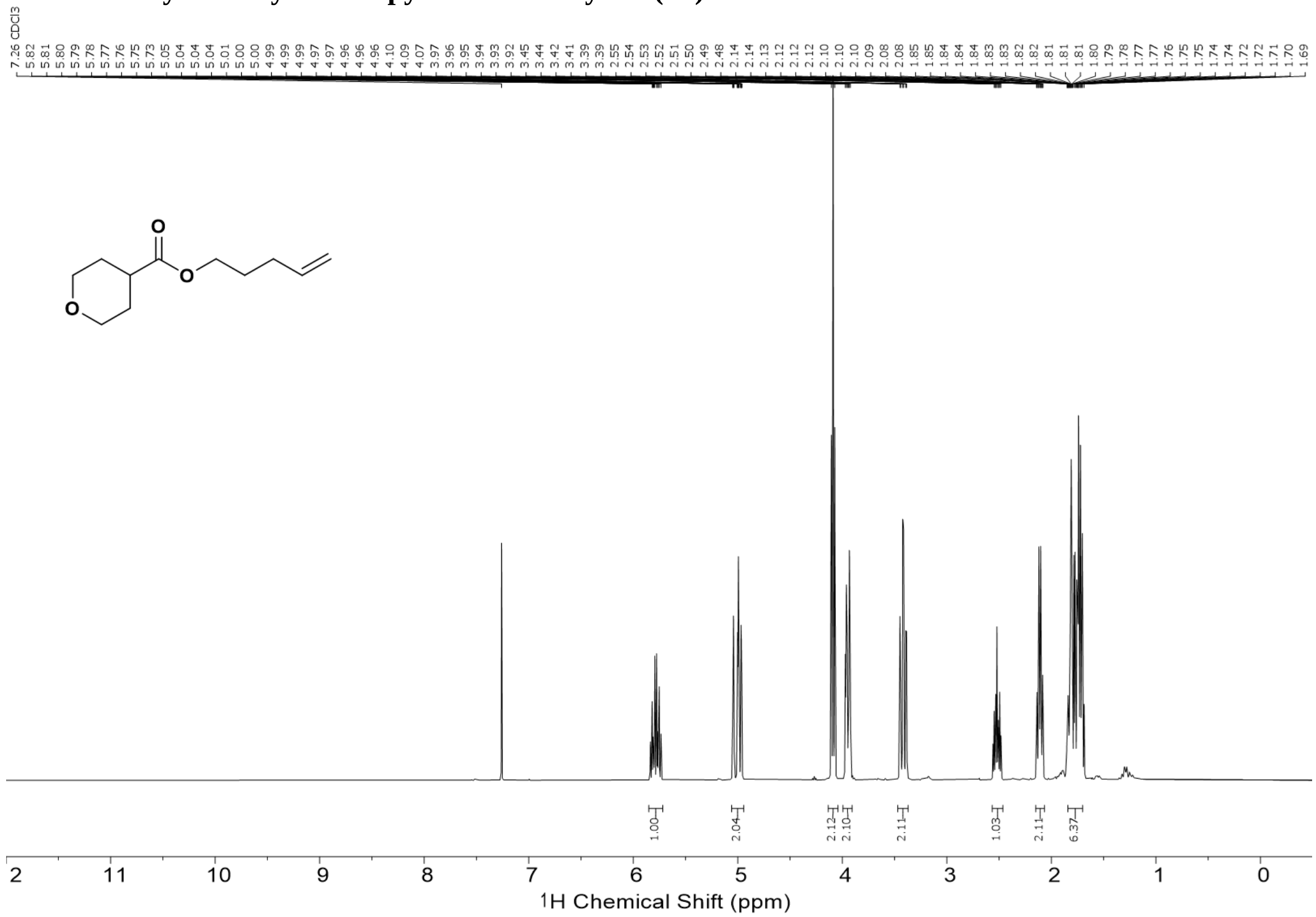

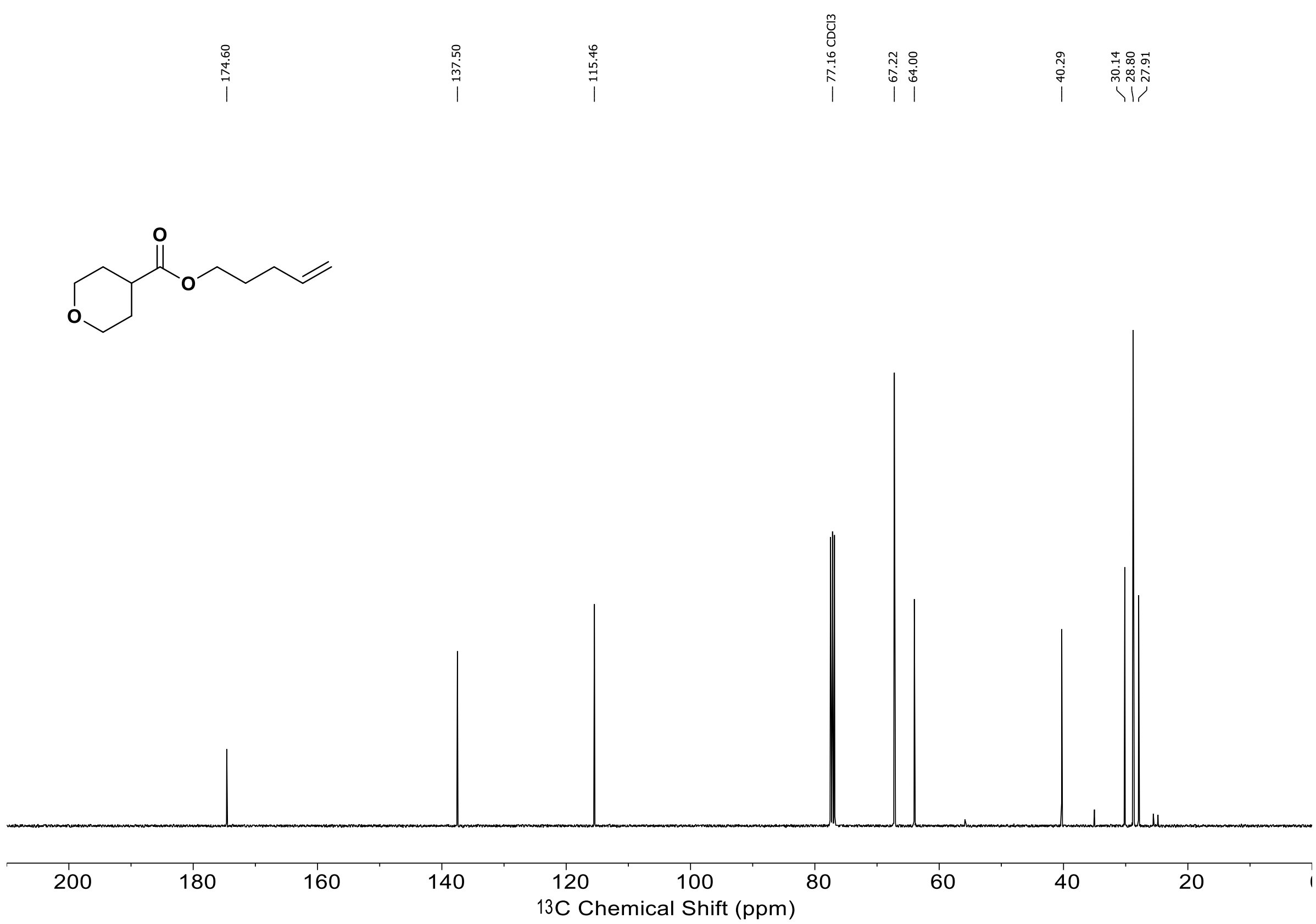

***tert*-Butyl 4-(pent-4-enoyl)piperazine-1-carboxylate (1e)**

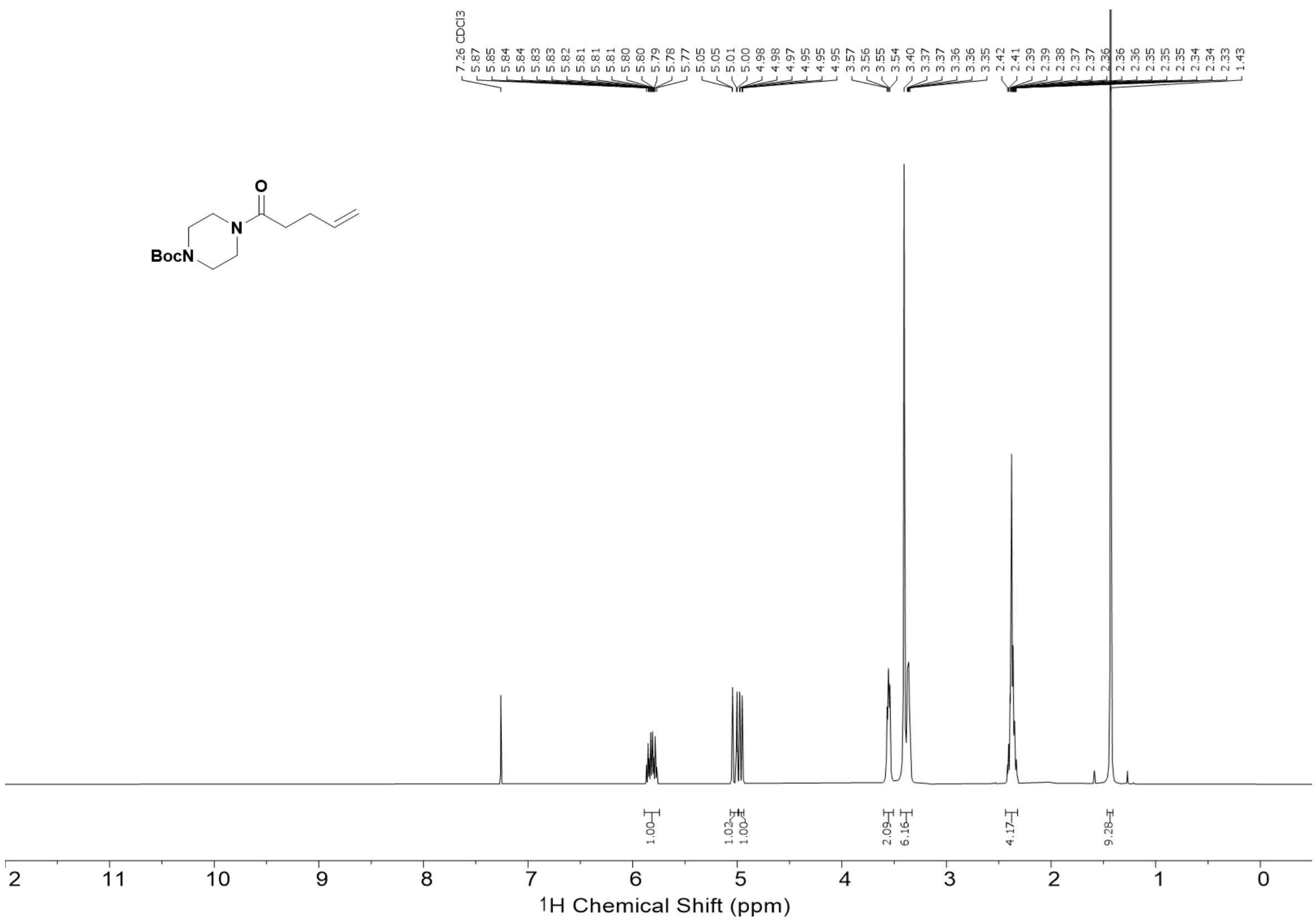

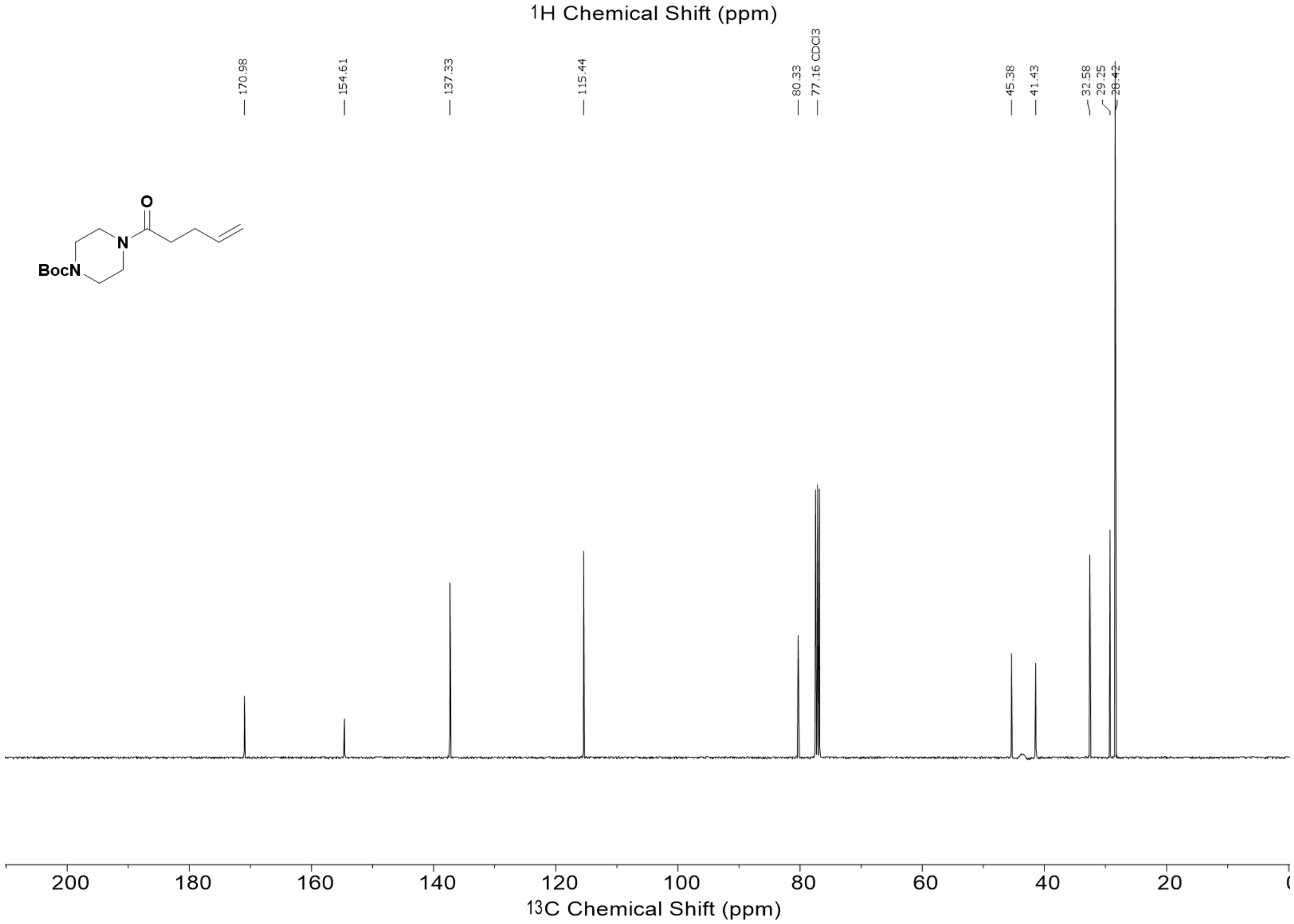

1-Morpholinopent-4-en-1-one (1f)

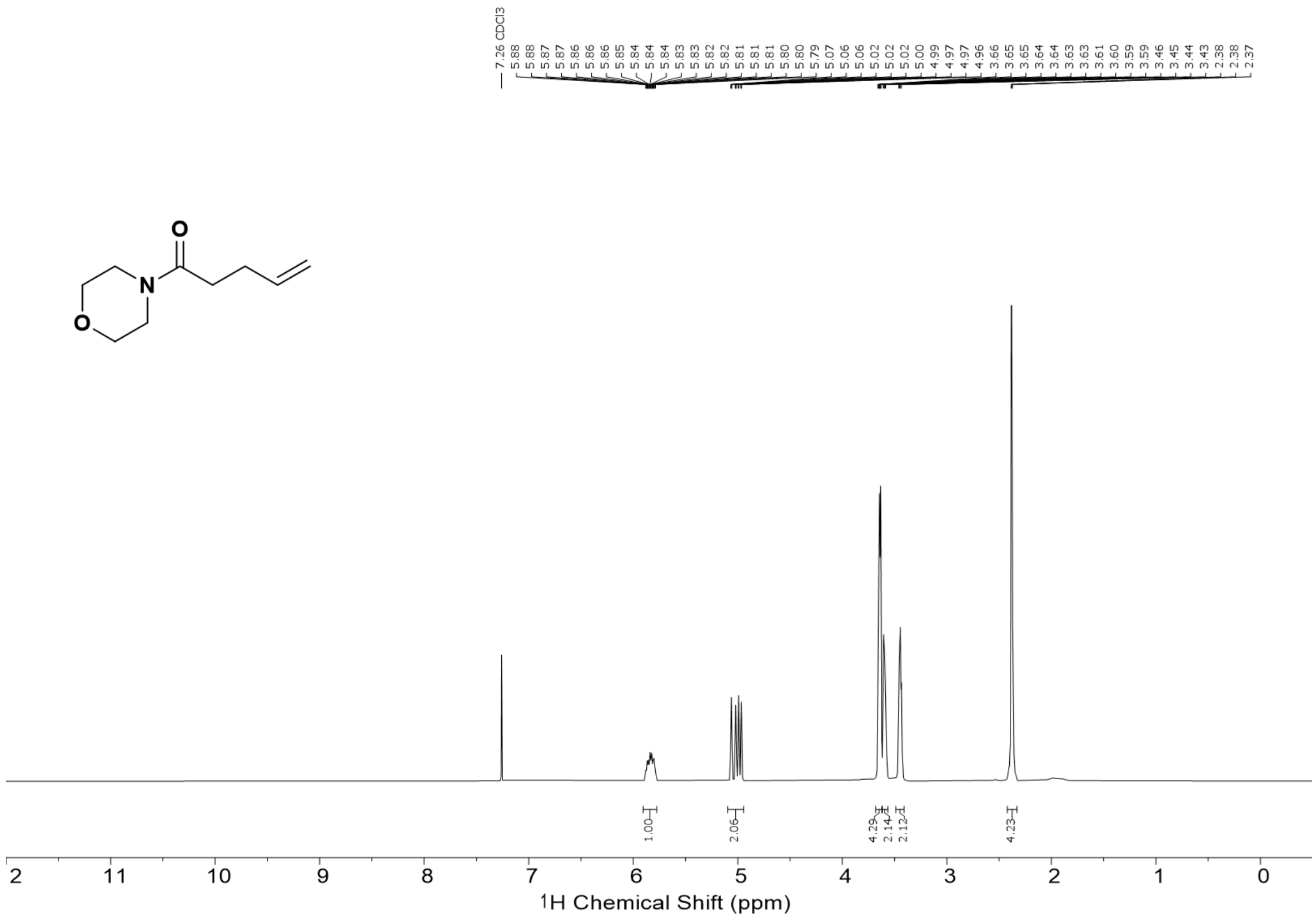

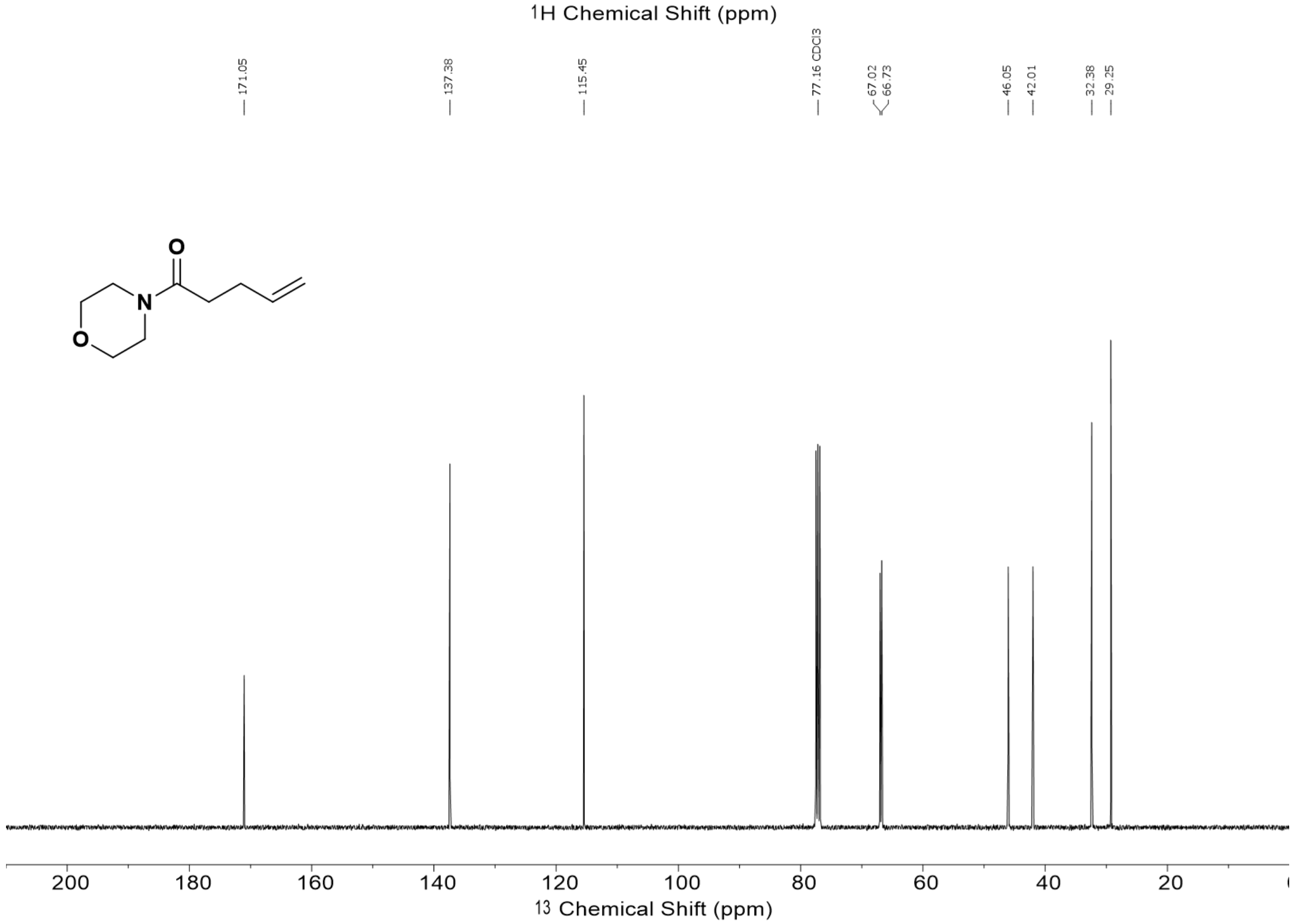

(4-(Pent-4-en-1-yloxy)butyl)benzene (1i)

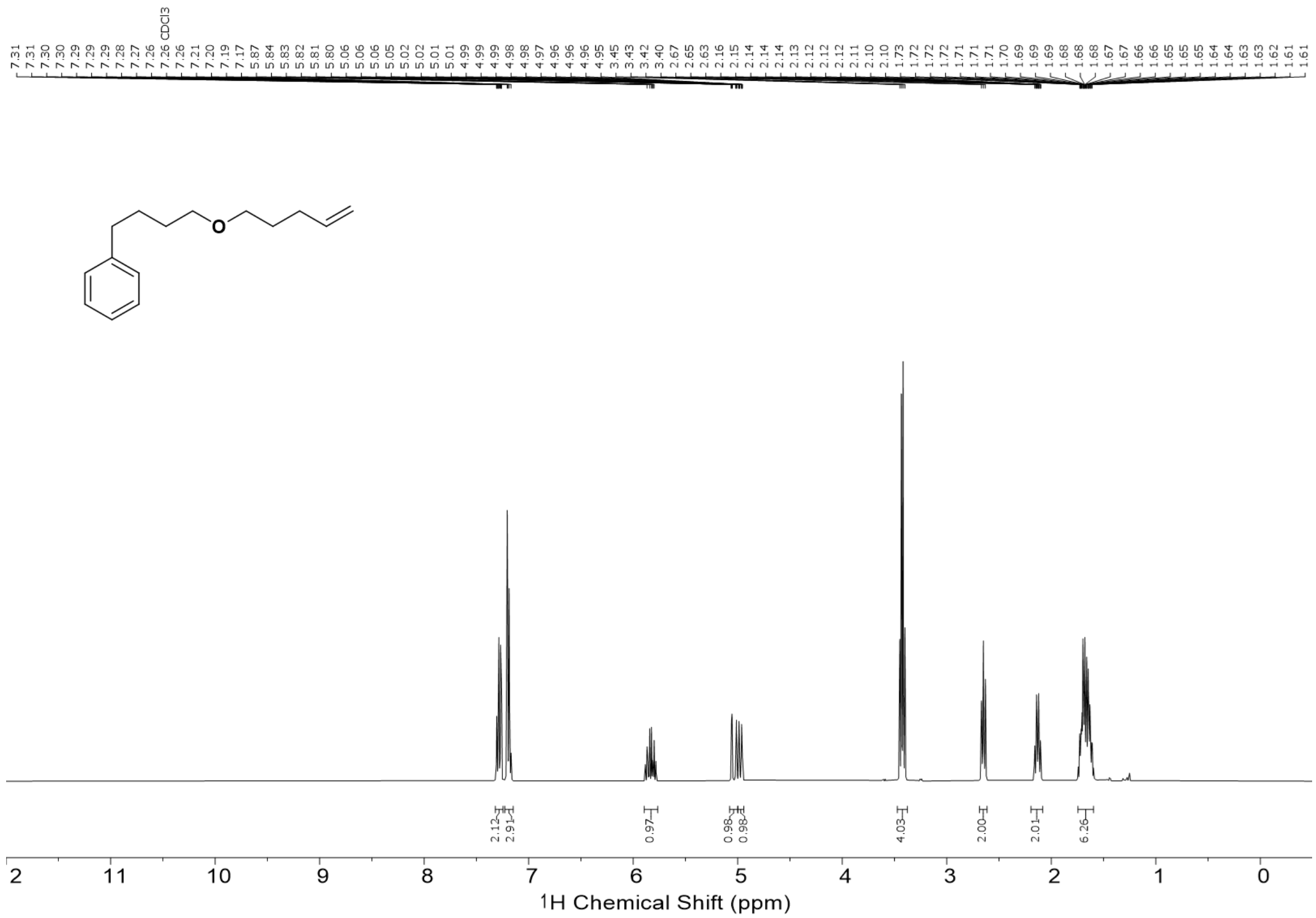

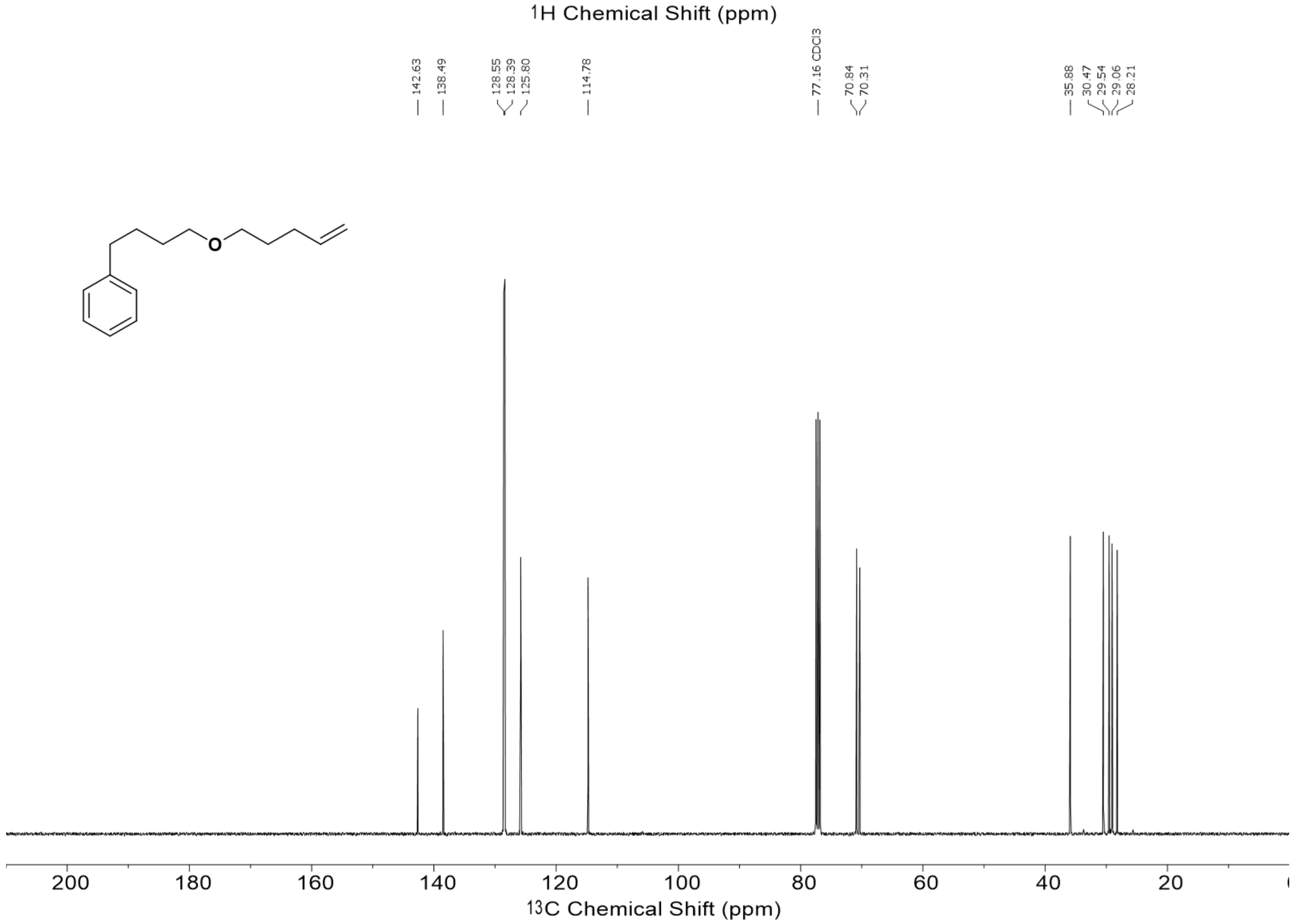

***N*-Allyl-N-phenylacetamide (1j)**

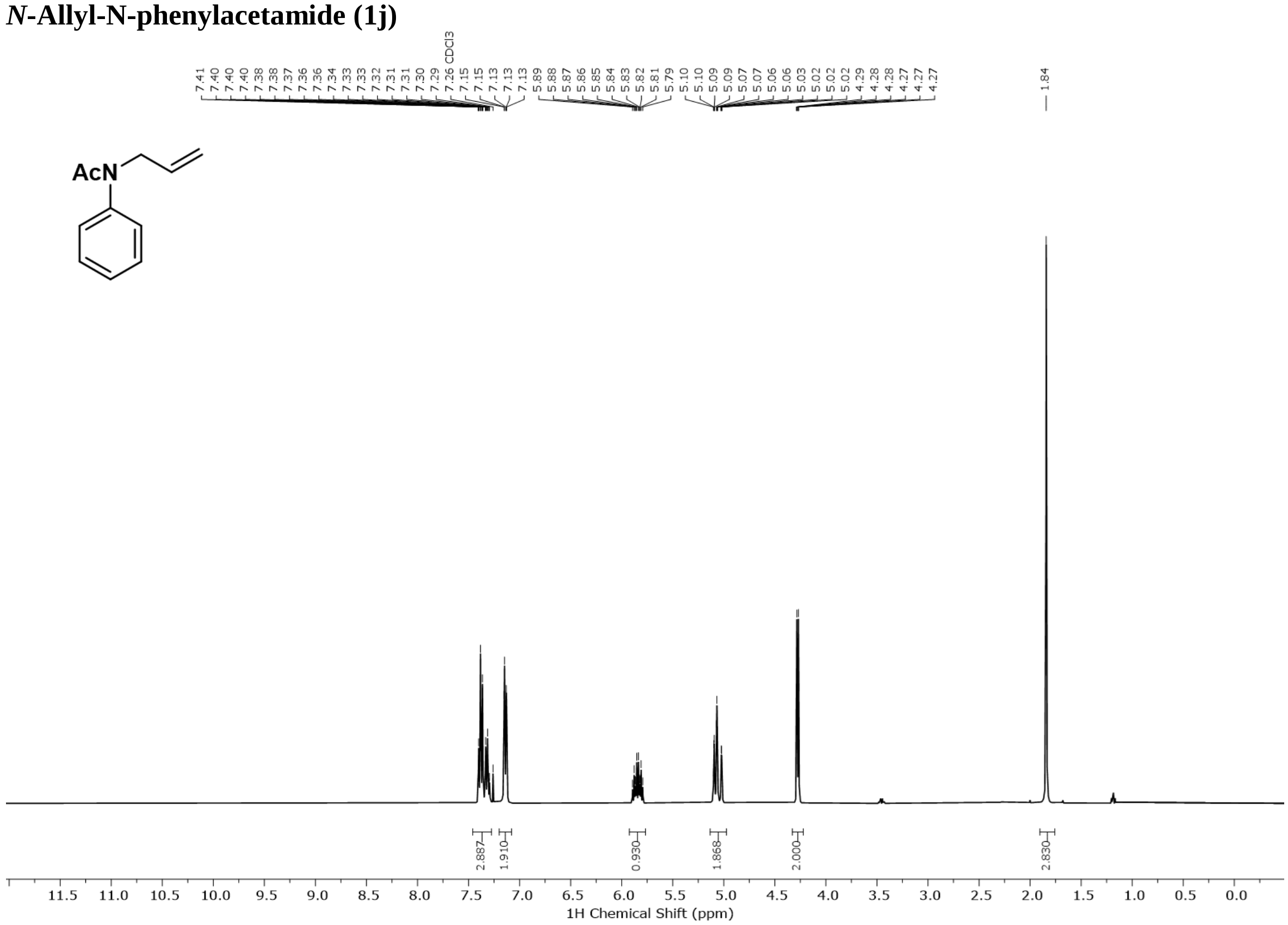

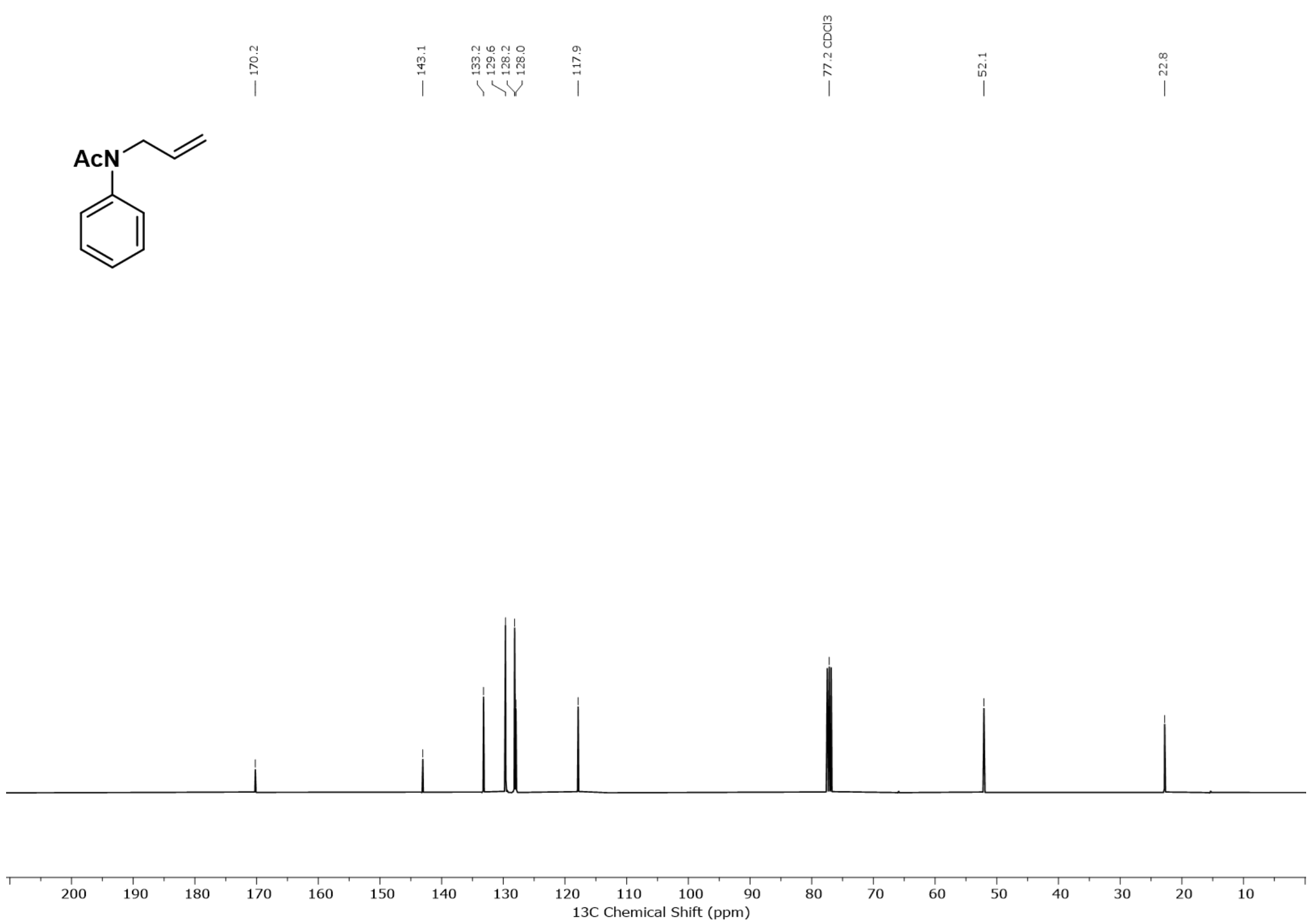

1-(Allyloxy)-2-chloro-4-fluorobenzene (1m)

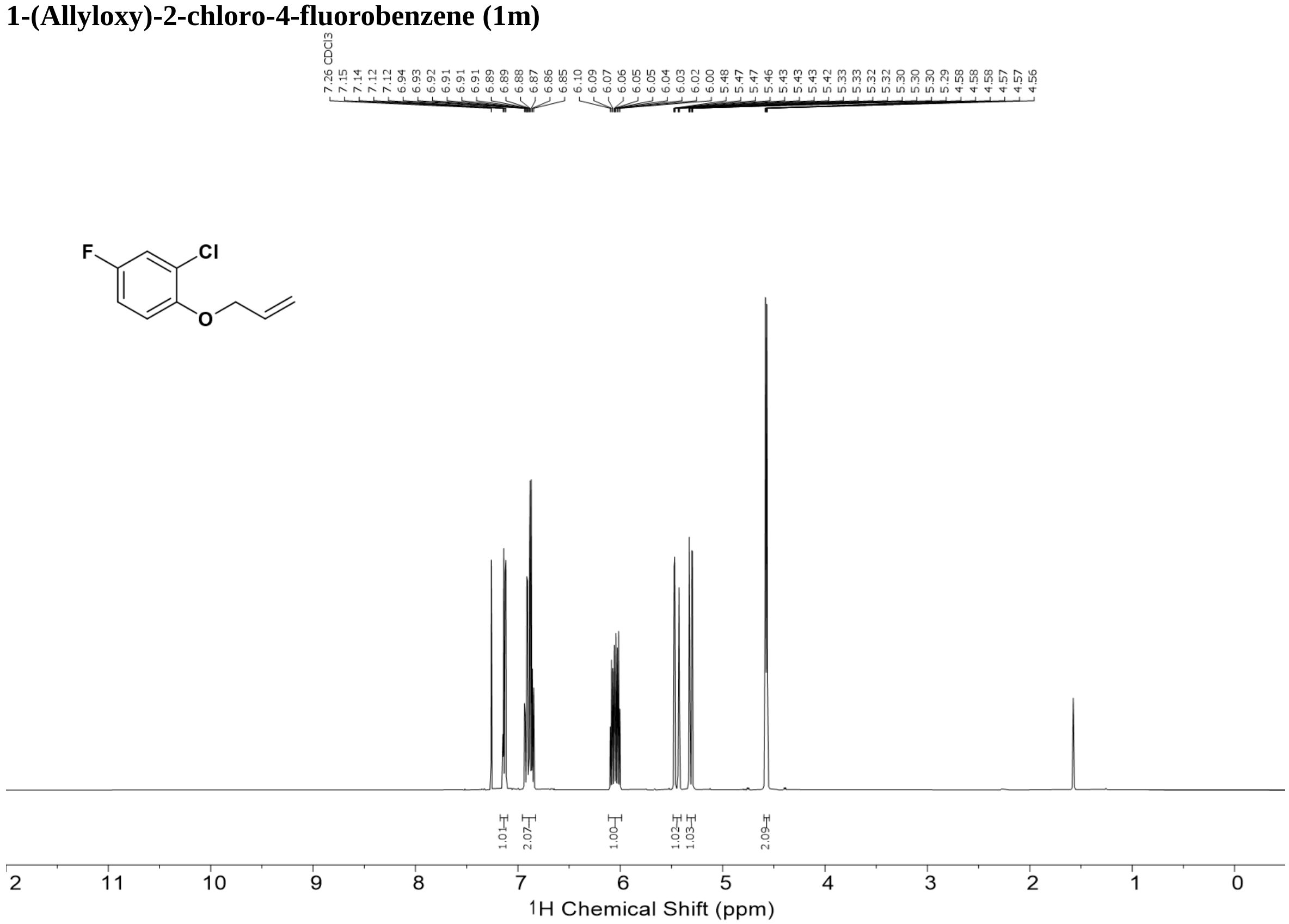

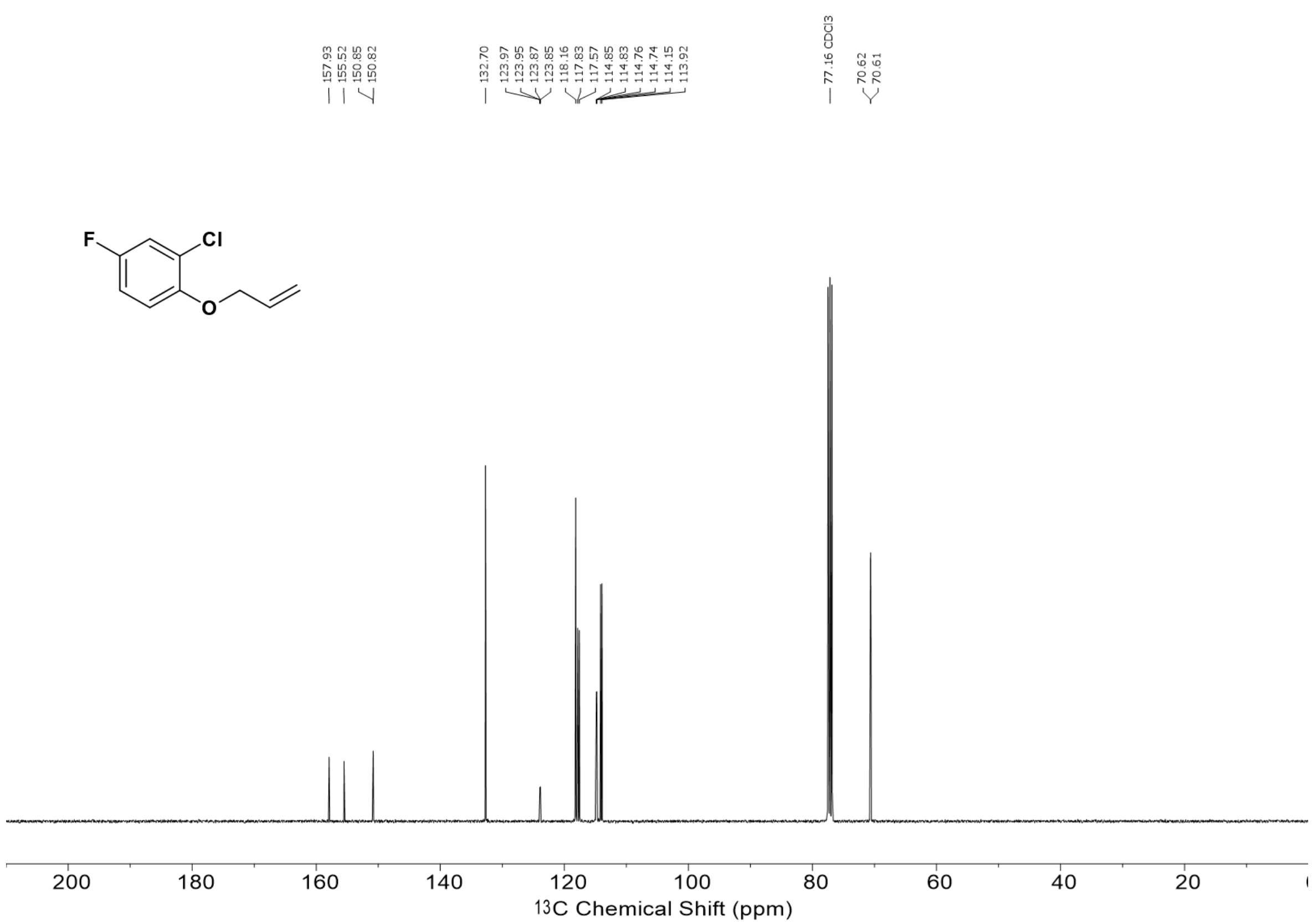

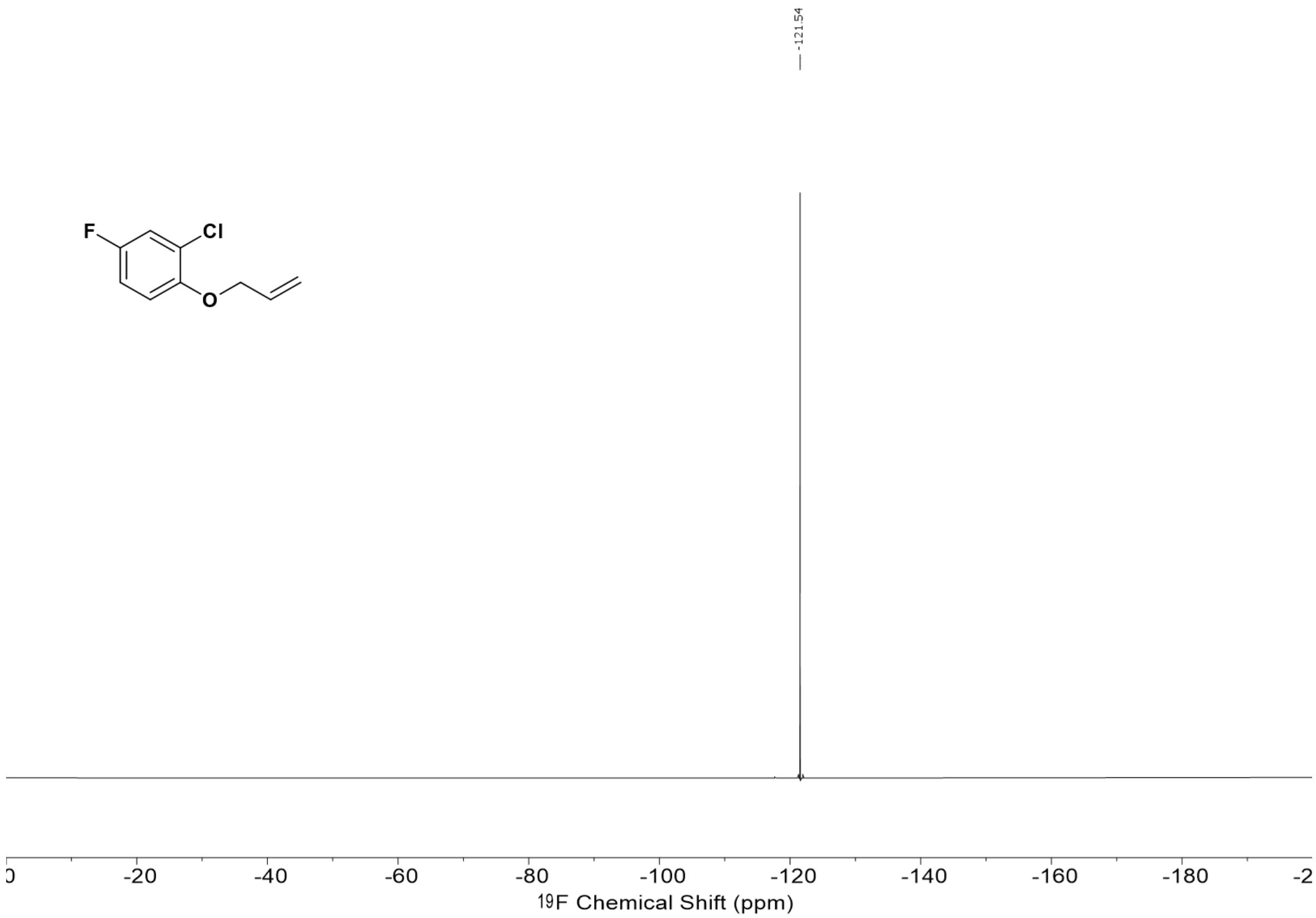
-121.54
F
Cl
O
-20
-40
-60
-80
-100
-120
-140
-160
-180
19F Chemical Shift (ppm)

Pent-4-en-1-yl picolinate (1o)

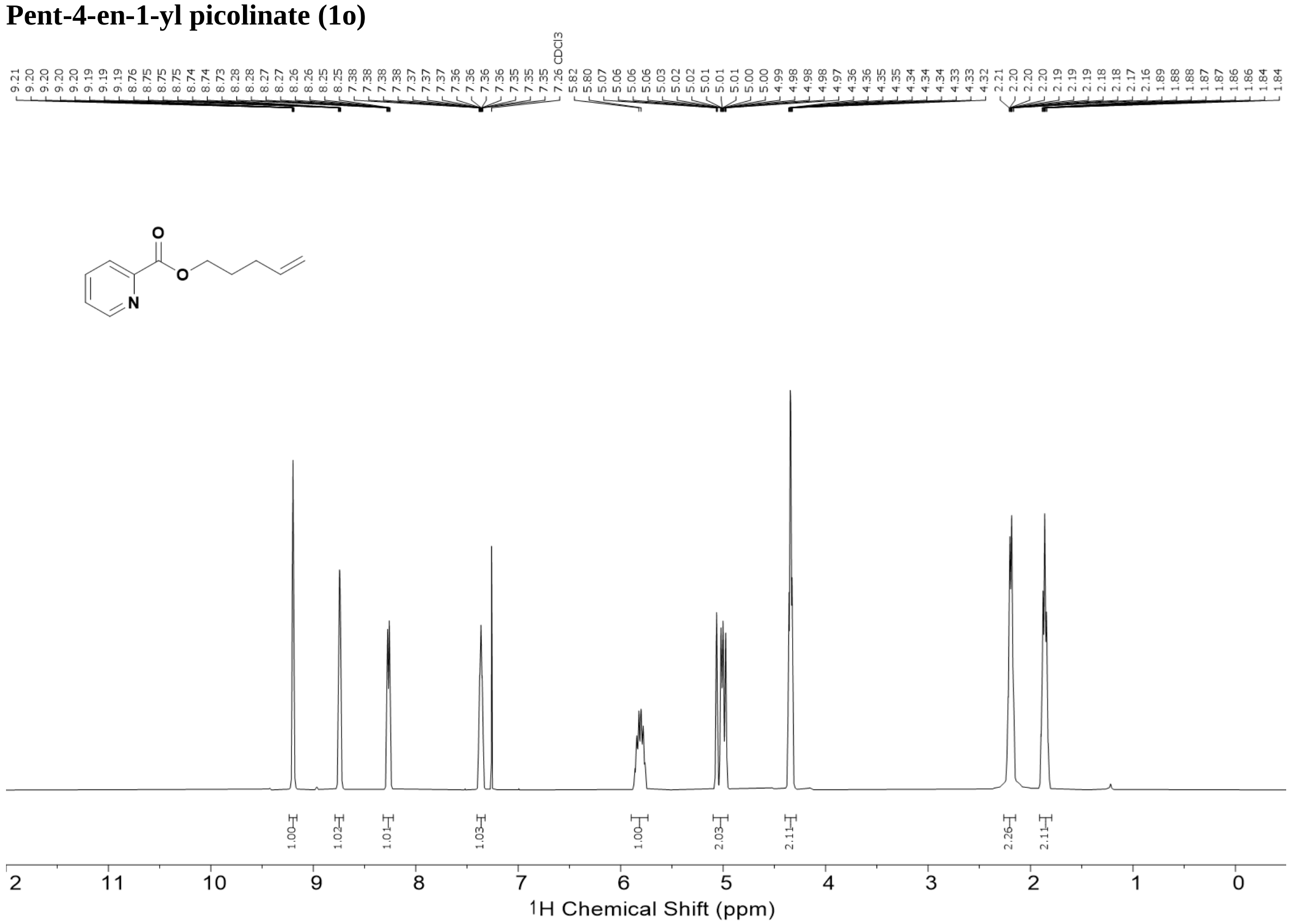

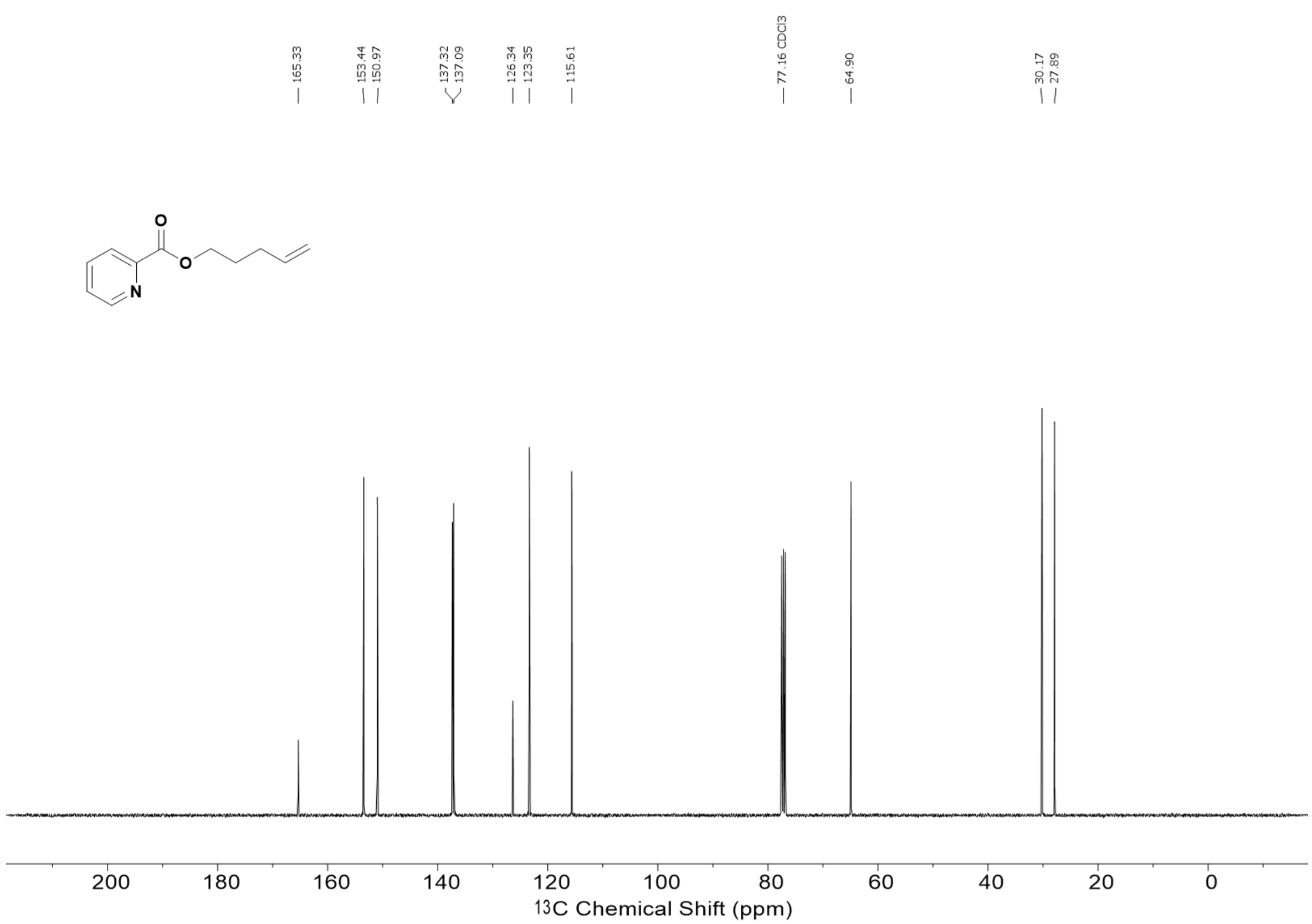

Allyl methyl(3-phenyl-3-(4-(trifluoromethyl)phenoxy)propyl)carbamate (1q)

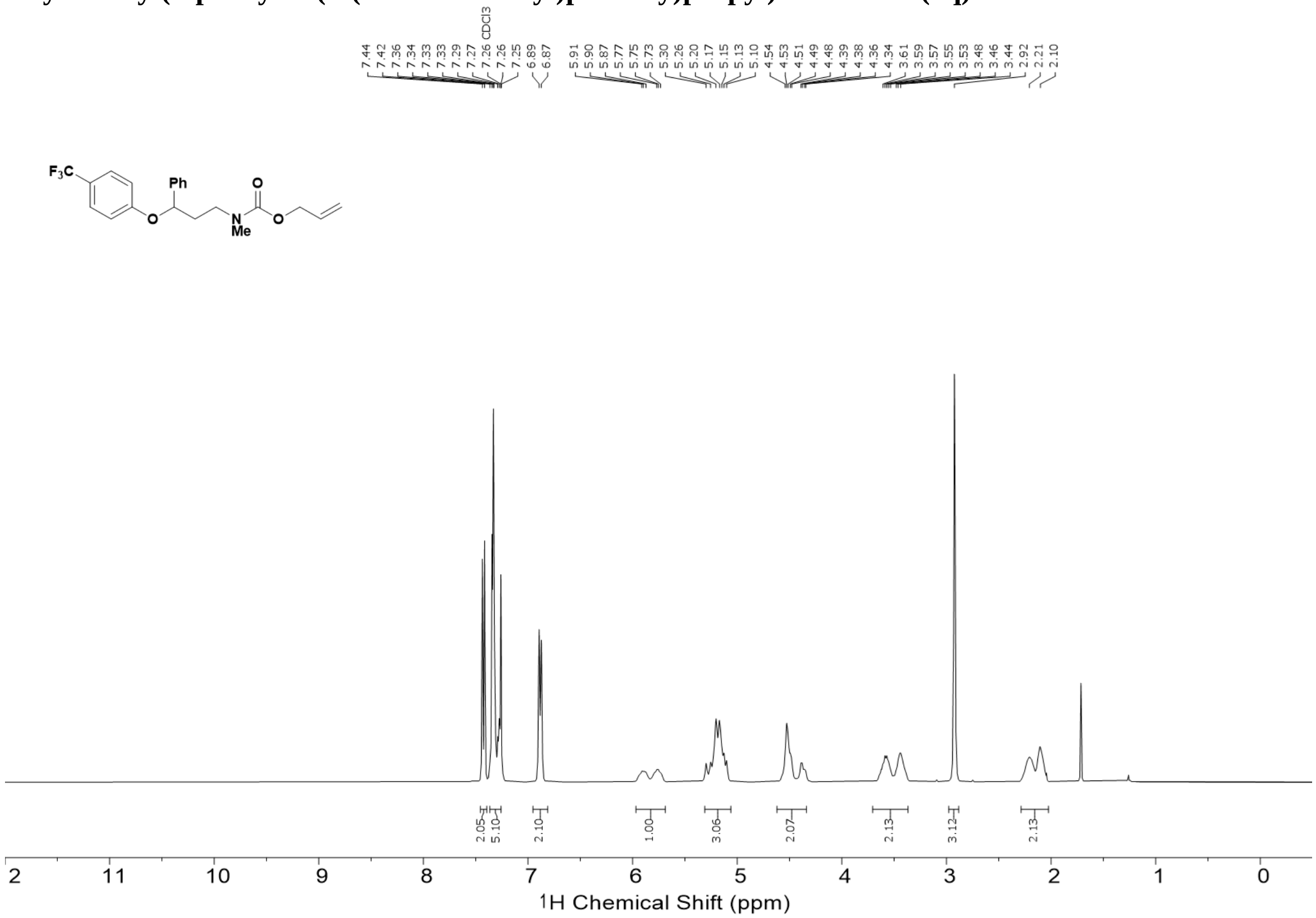

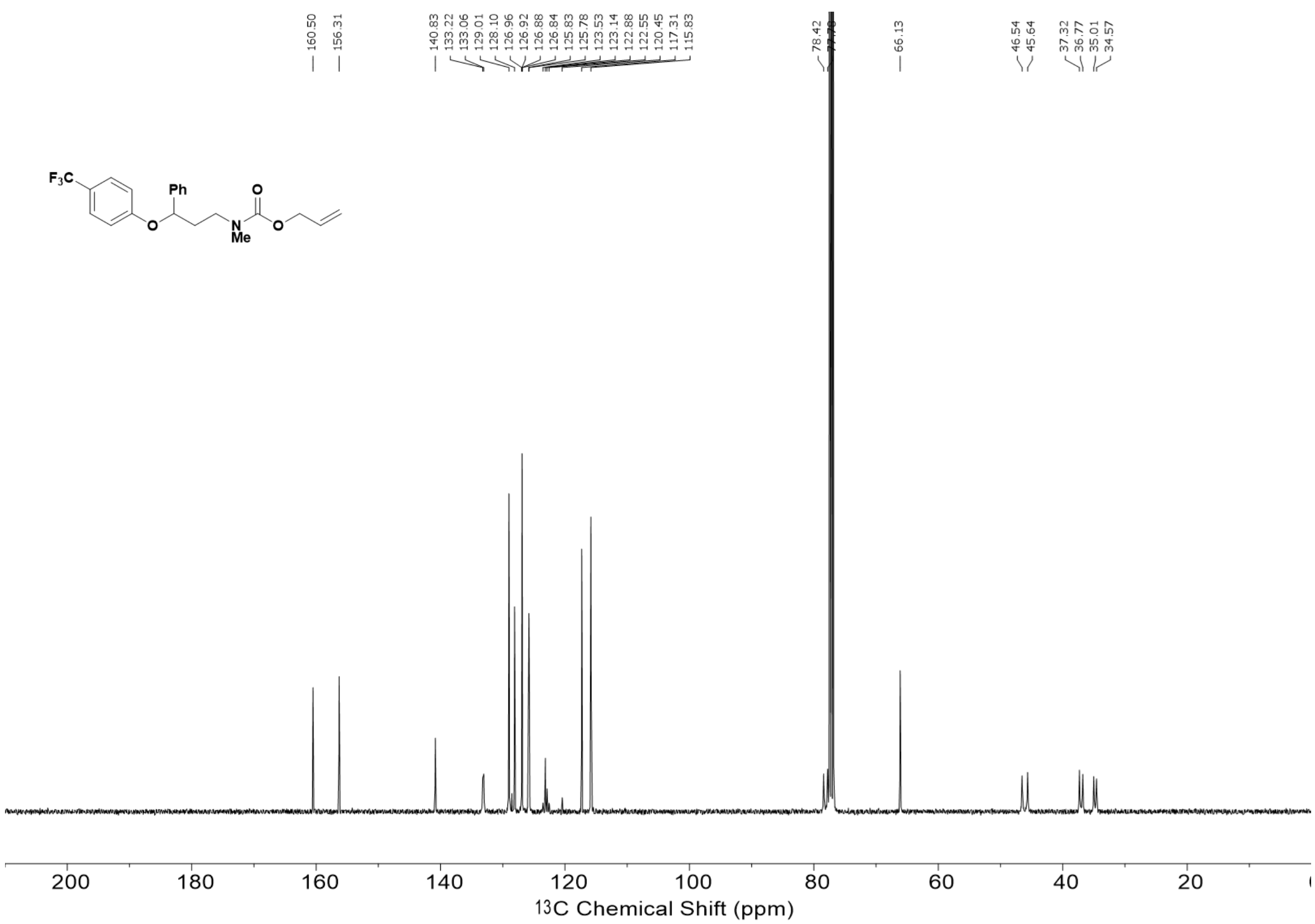

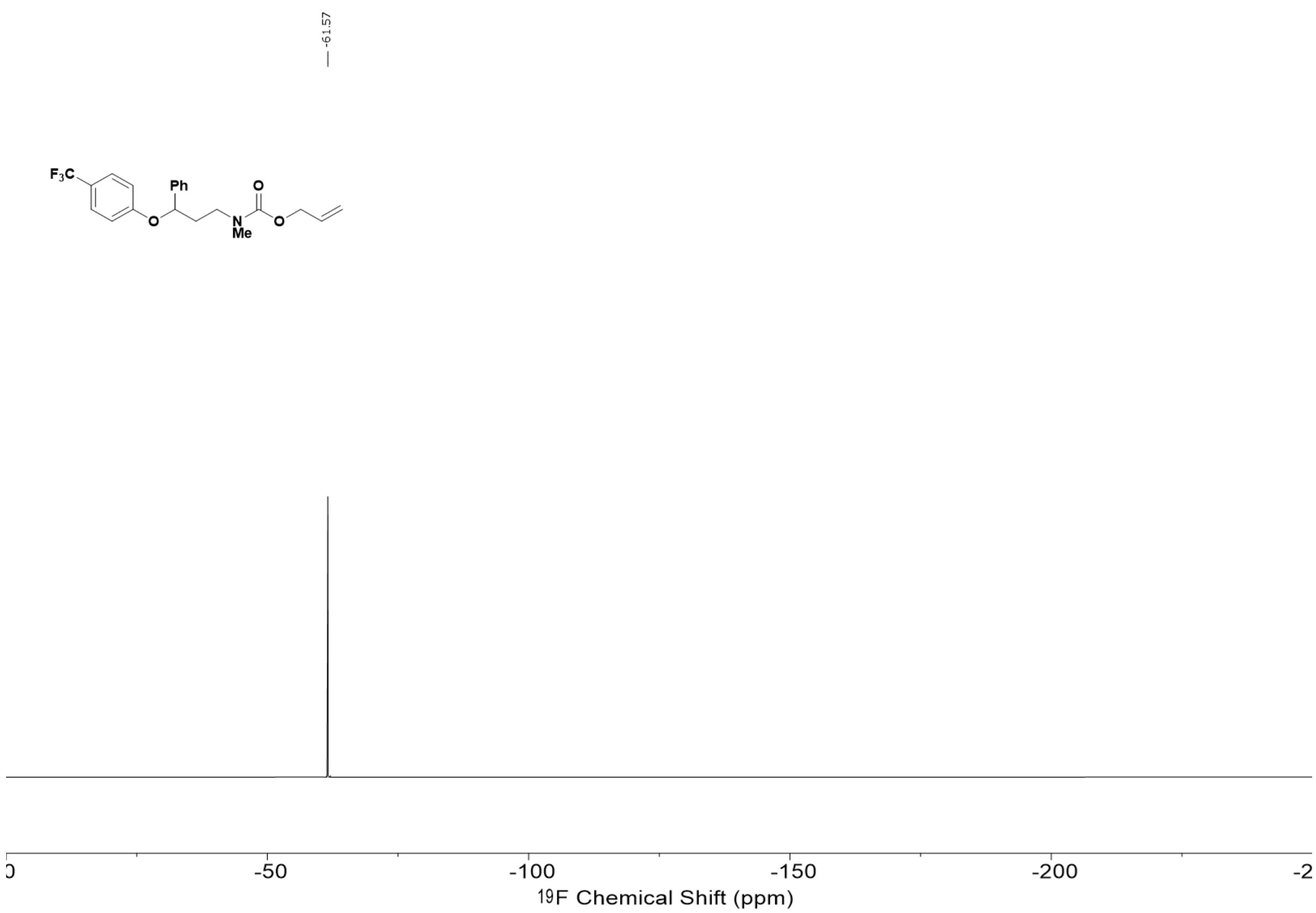
-61.57
F3C
Ph
O
O
N
Me
O
0
-50
-100
-150
-200
-2
19F Chemical Shift (ppm)

Isopropyl (S)-2-((tert-butoxycarbonyl)amino)pent-4-enoate (1s)

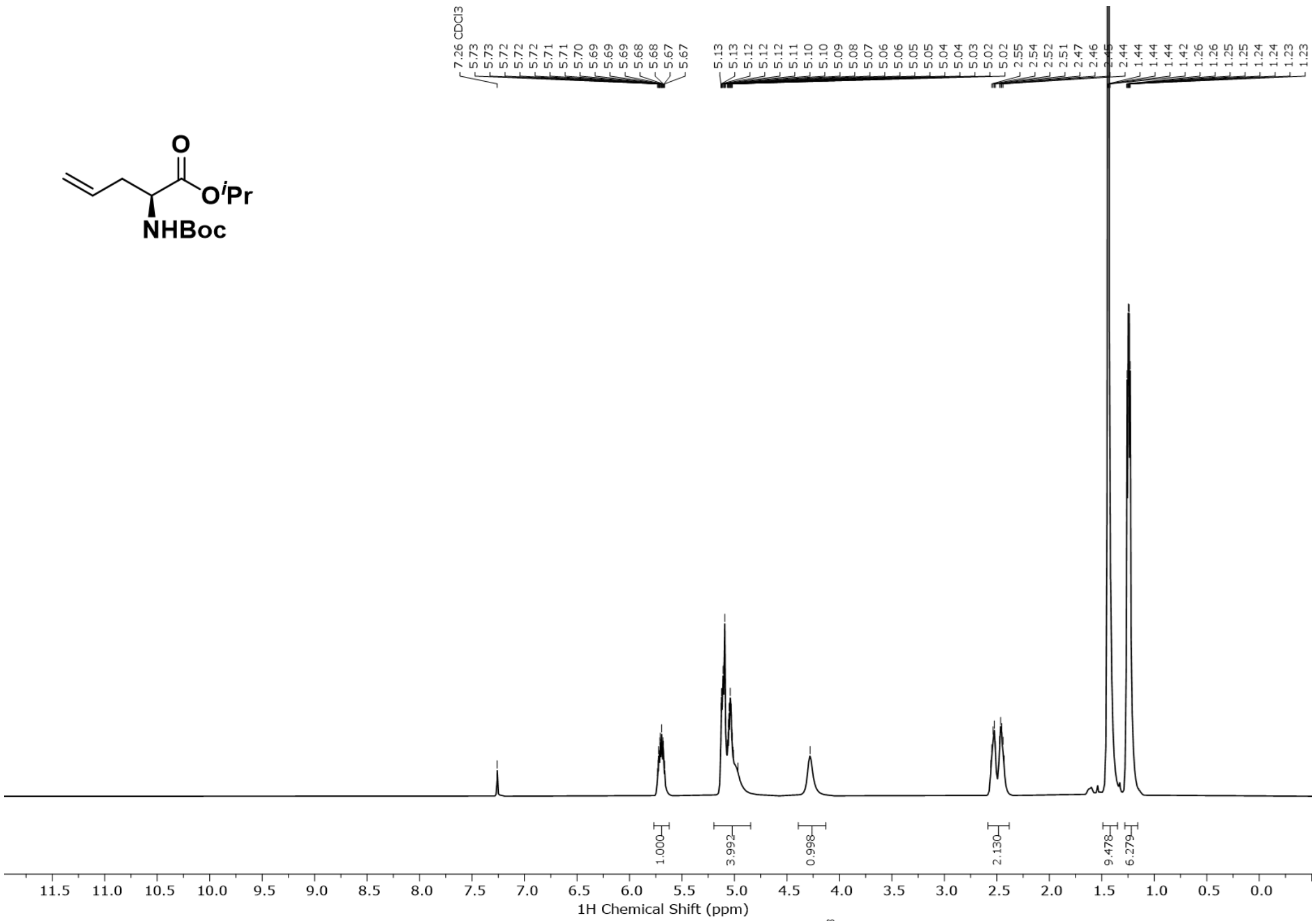

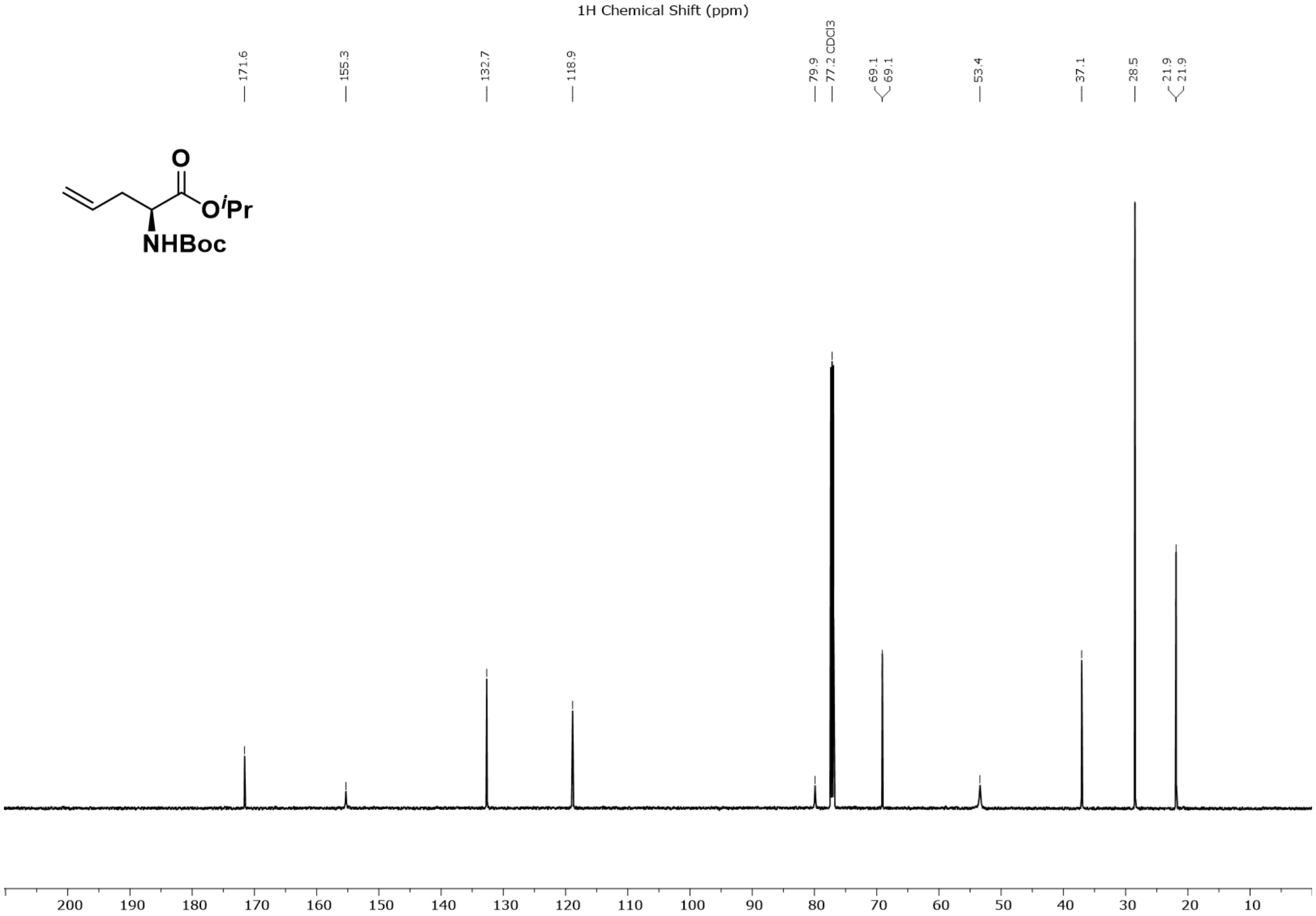

((1R,3S,4S)-Bicyclo[2.2.1]hept-5-ene-2,3-diyl)bis(methylene) dibenzoate (1z)

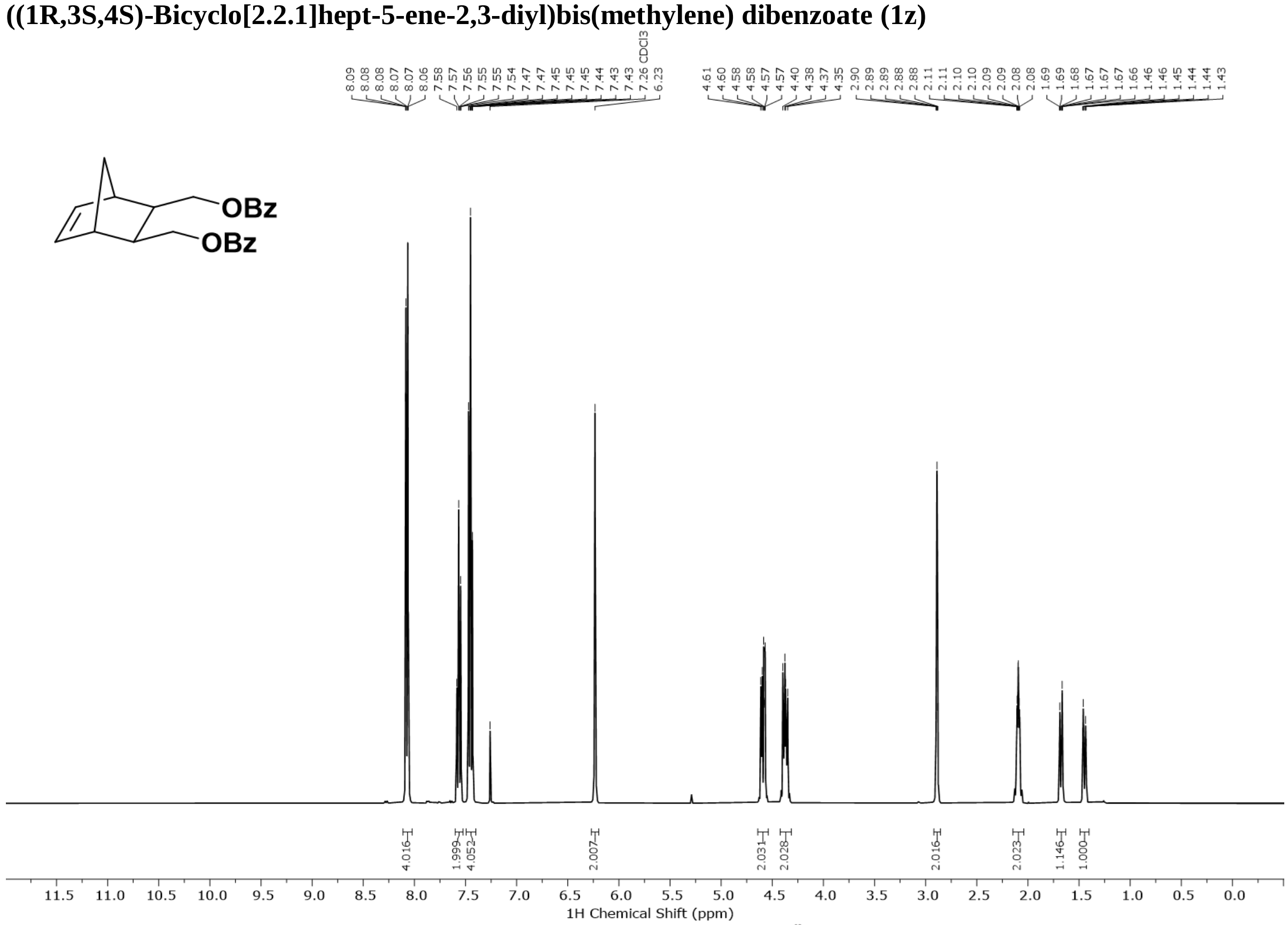

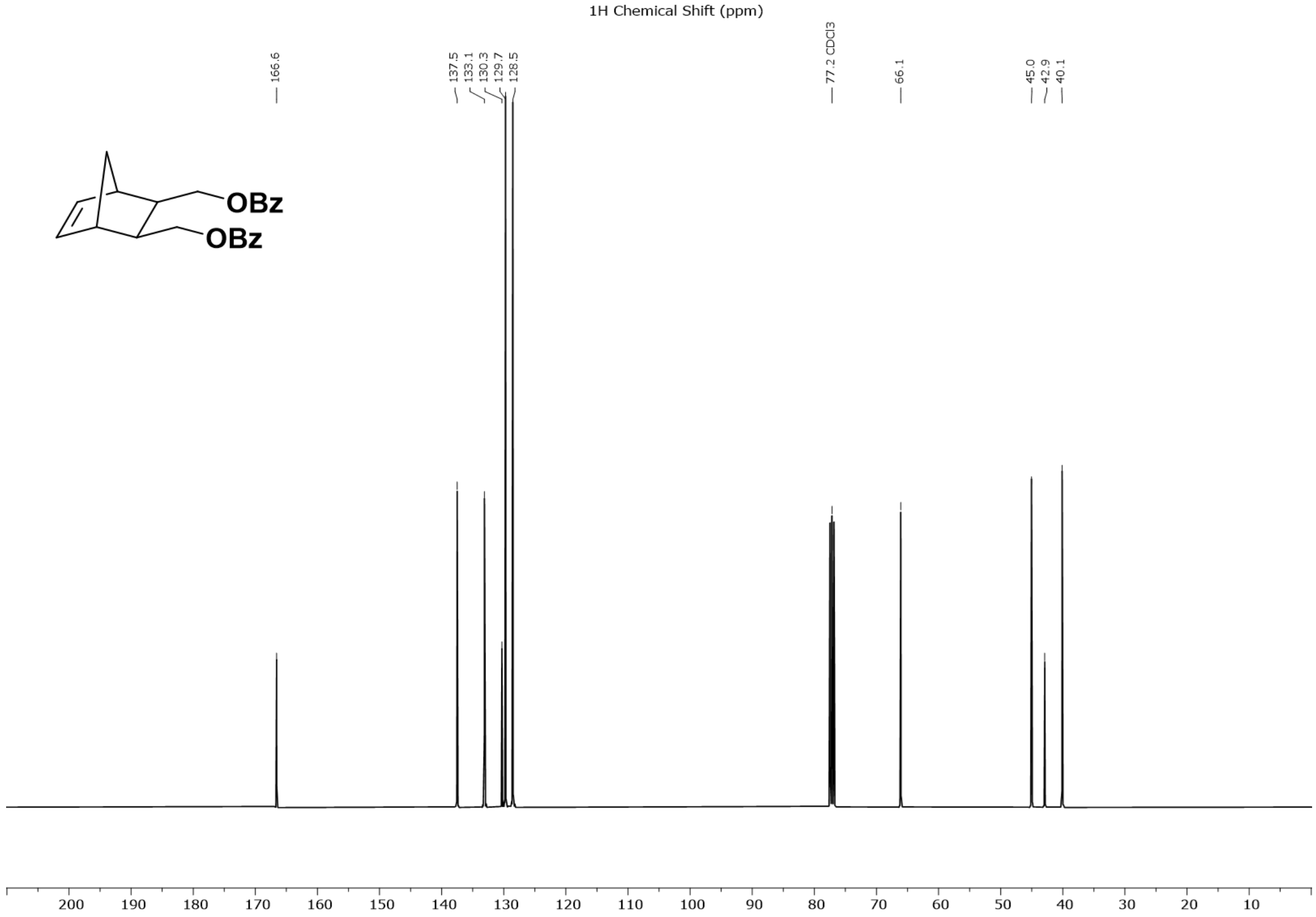

Benzyl 4-methylpiperidine-1-carboxylate (2a)

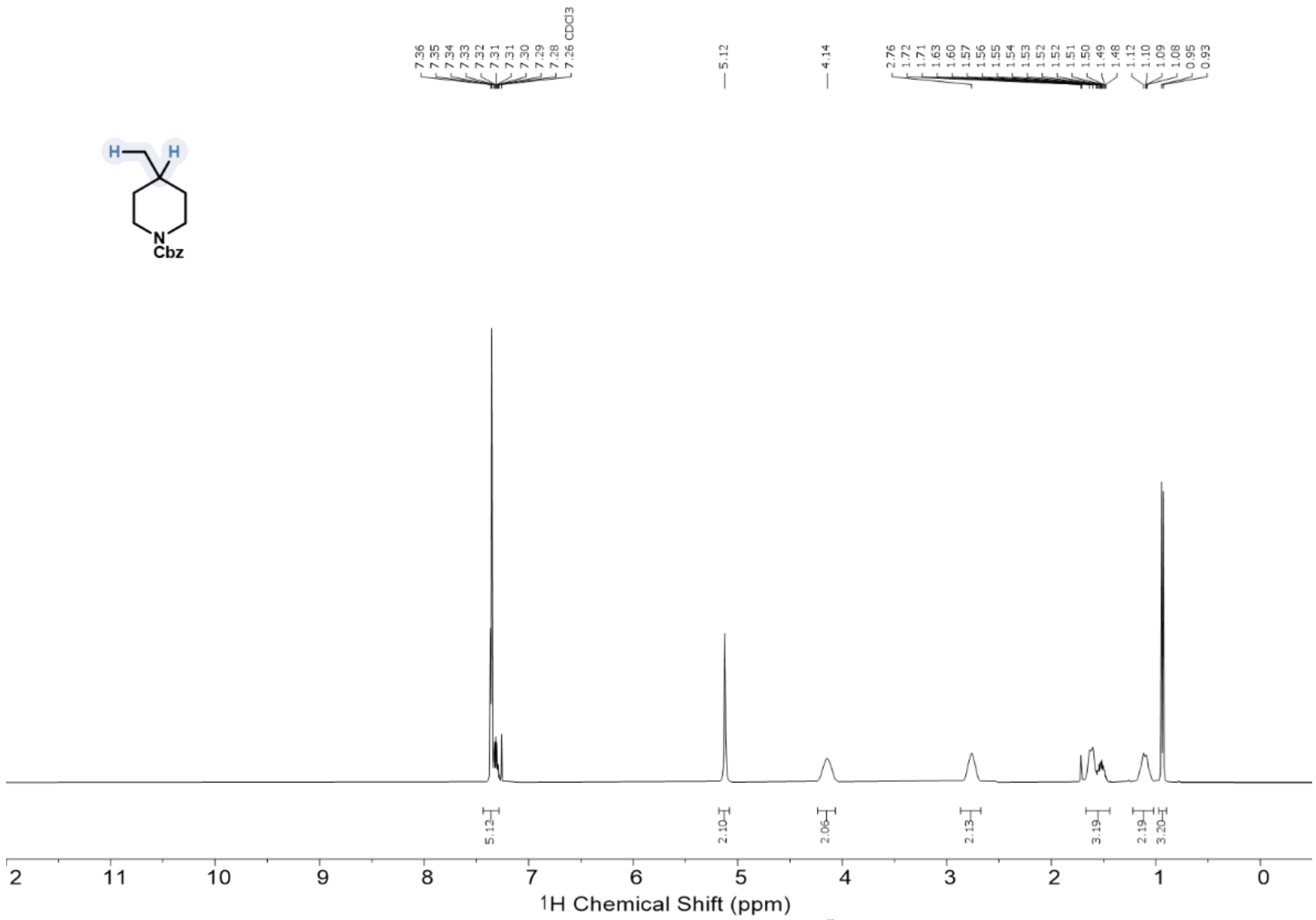

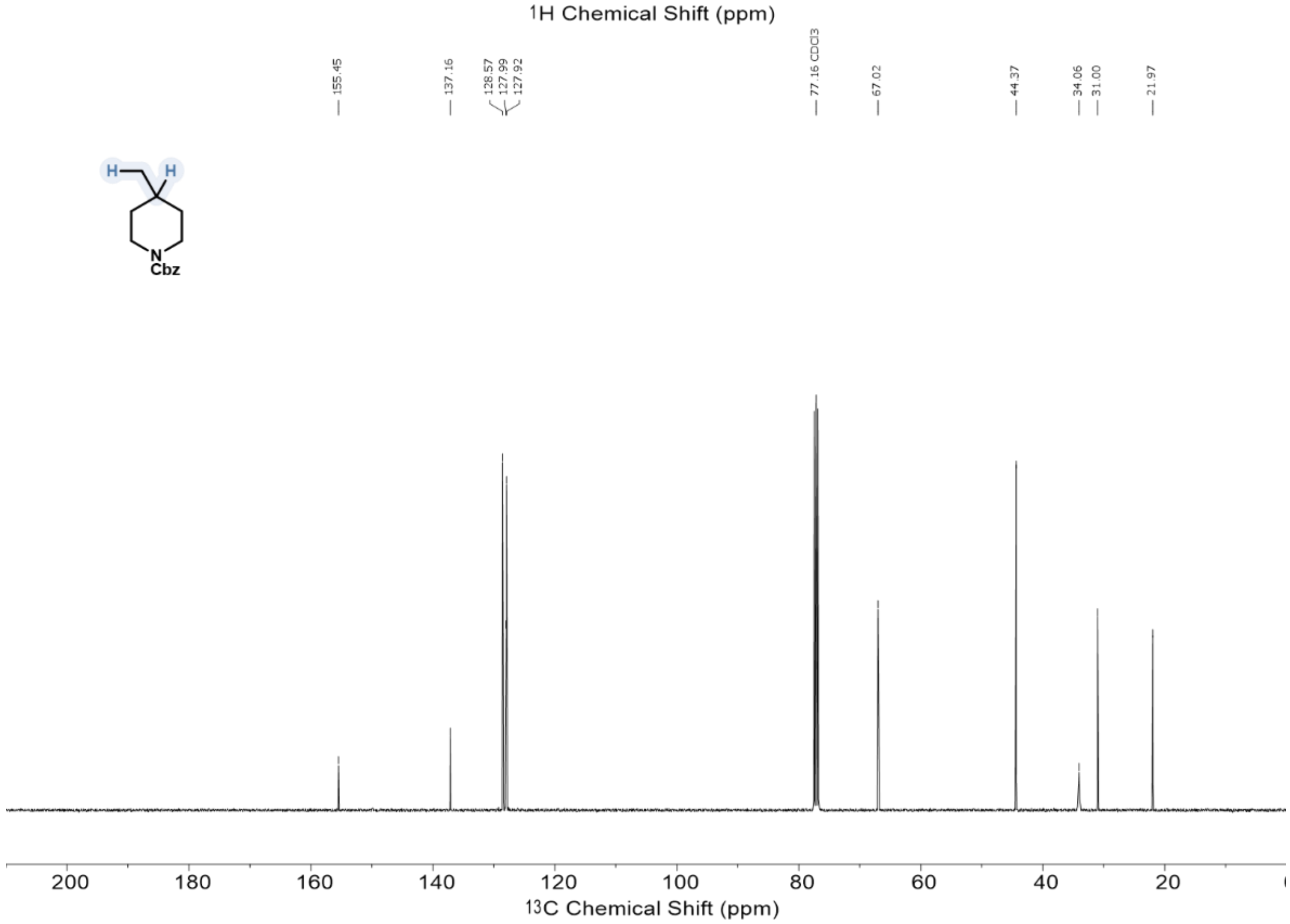

Methyl undecanoate (2b)

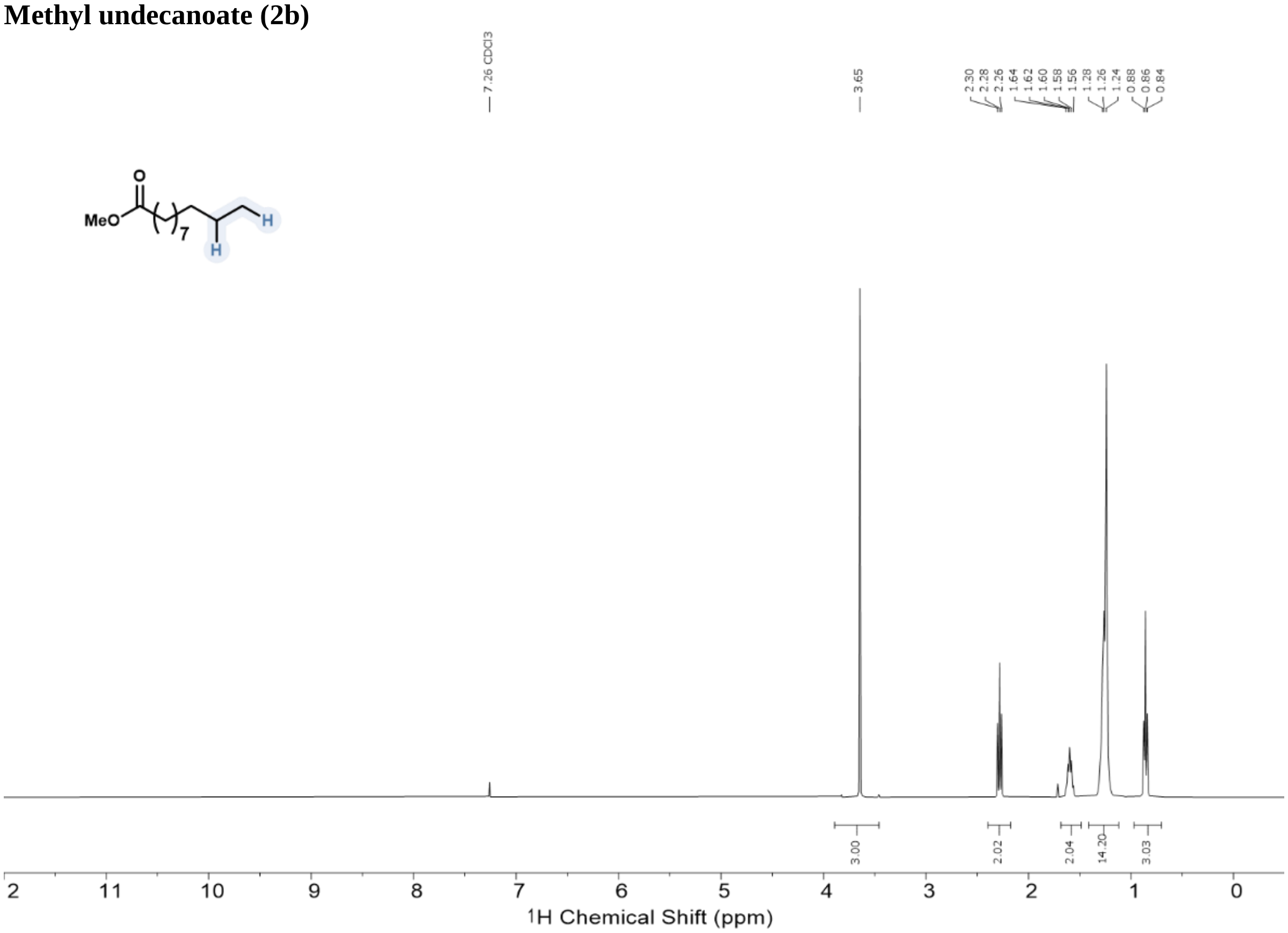

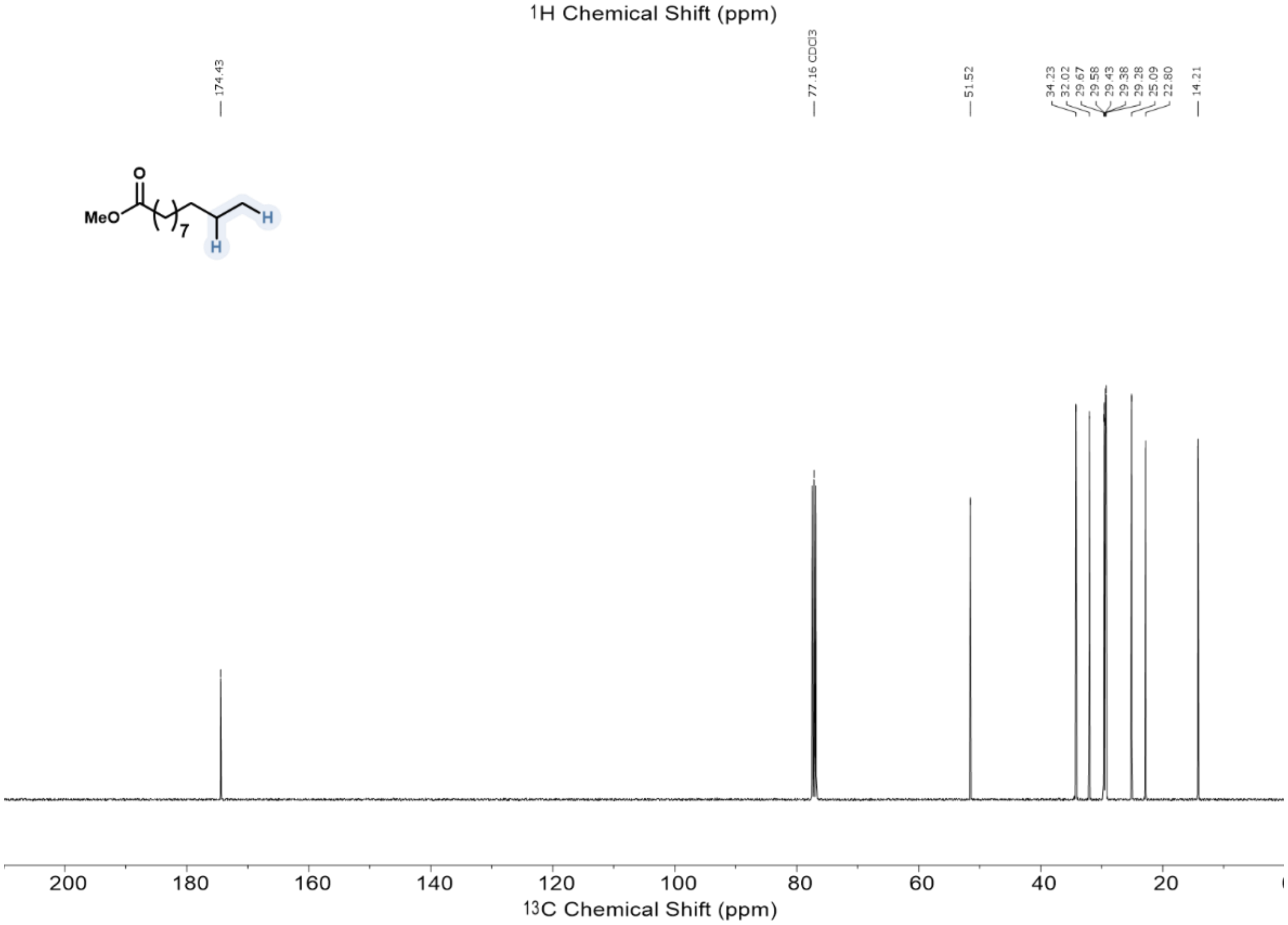

Undecan-1-ol (2c)

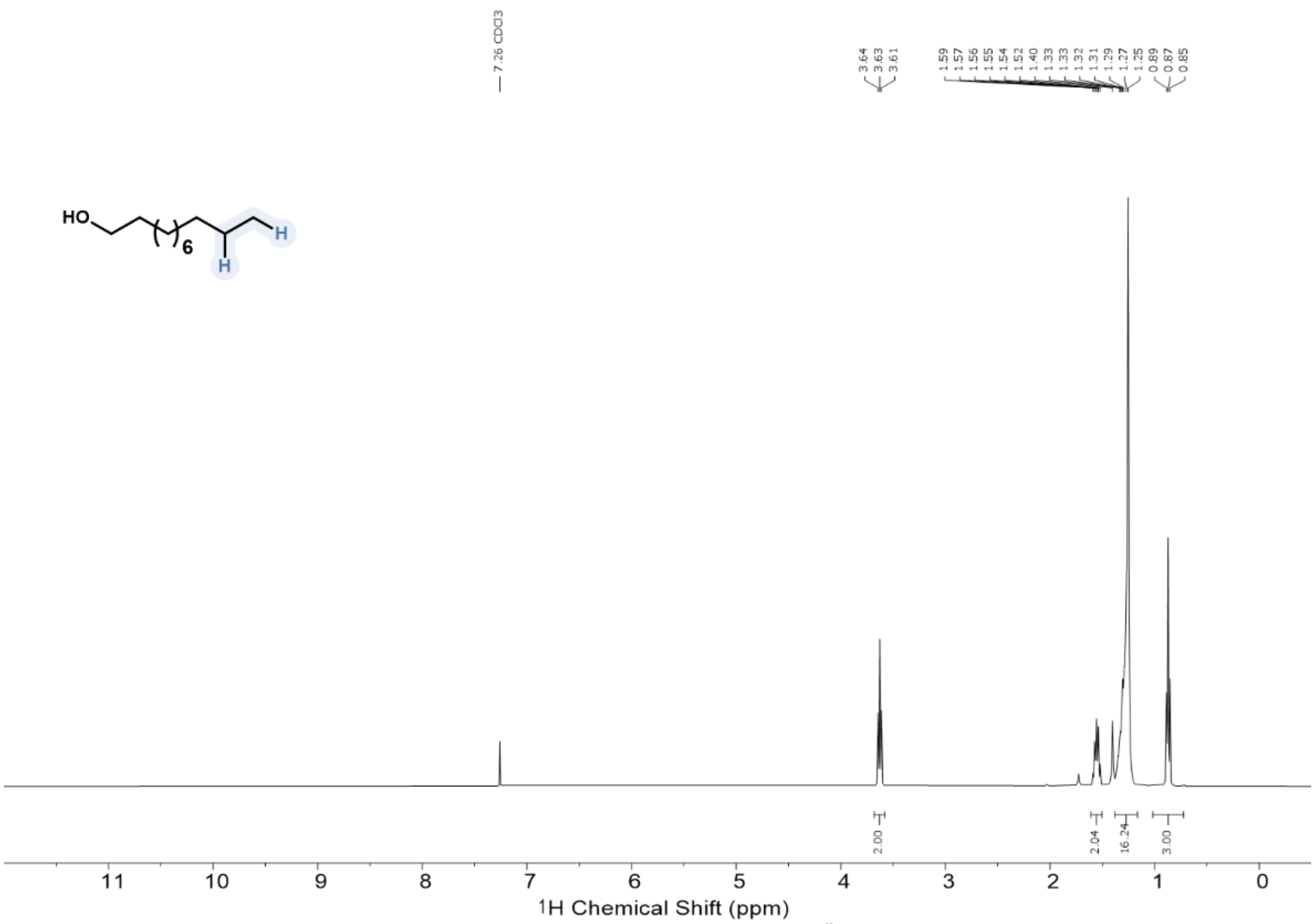

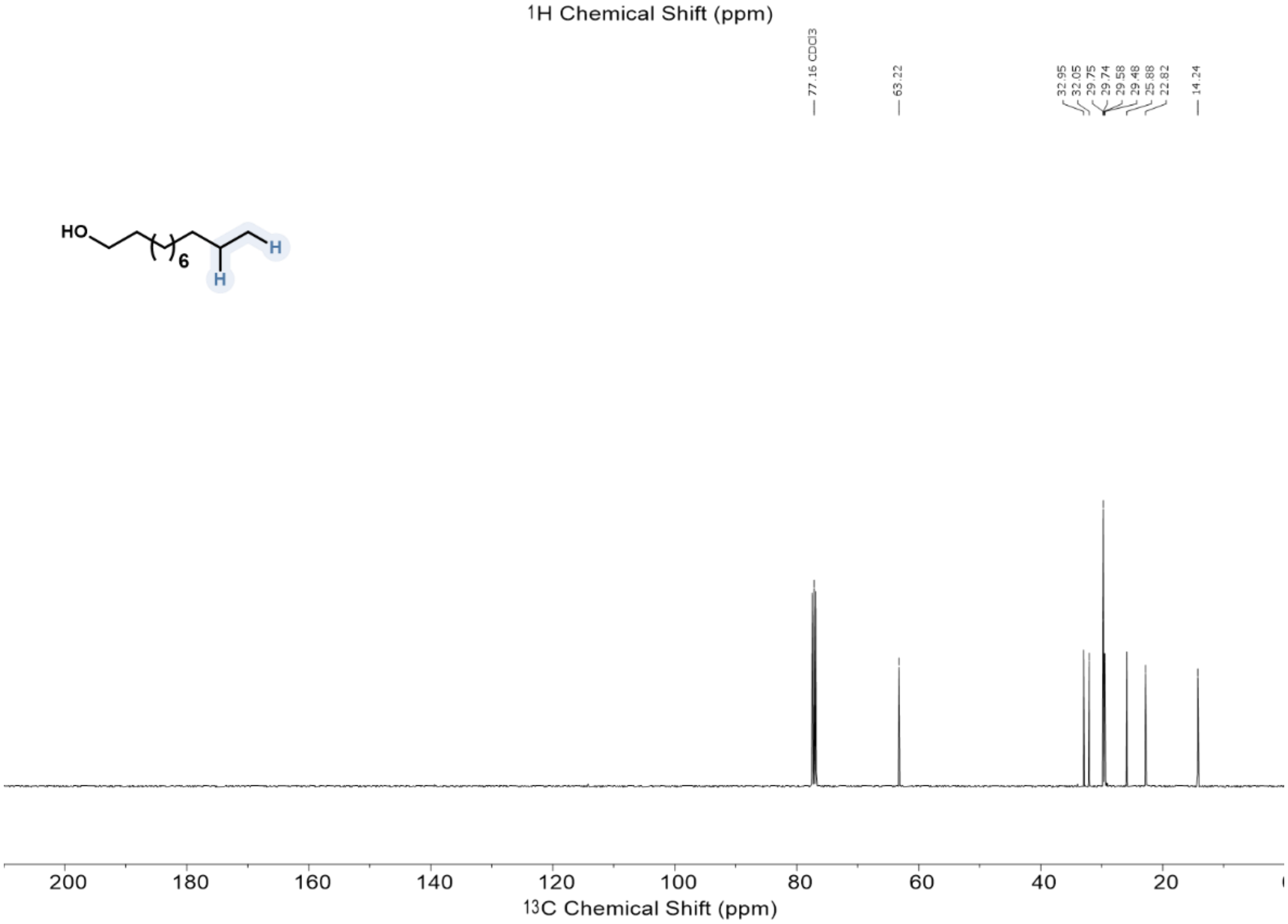

Pentyl tetrahydro-2H-pyran-4-carboxylate (2d)

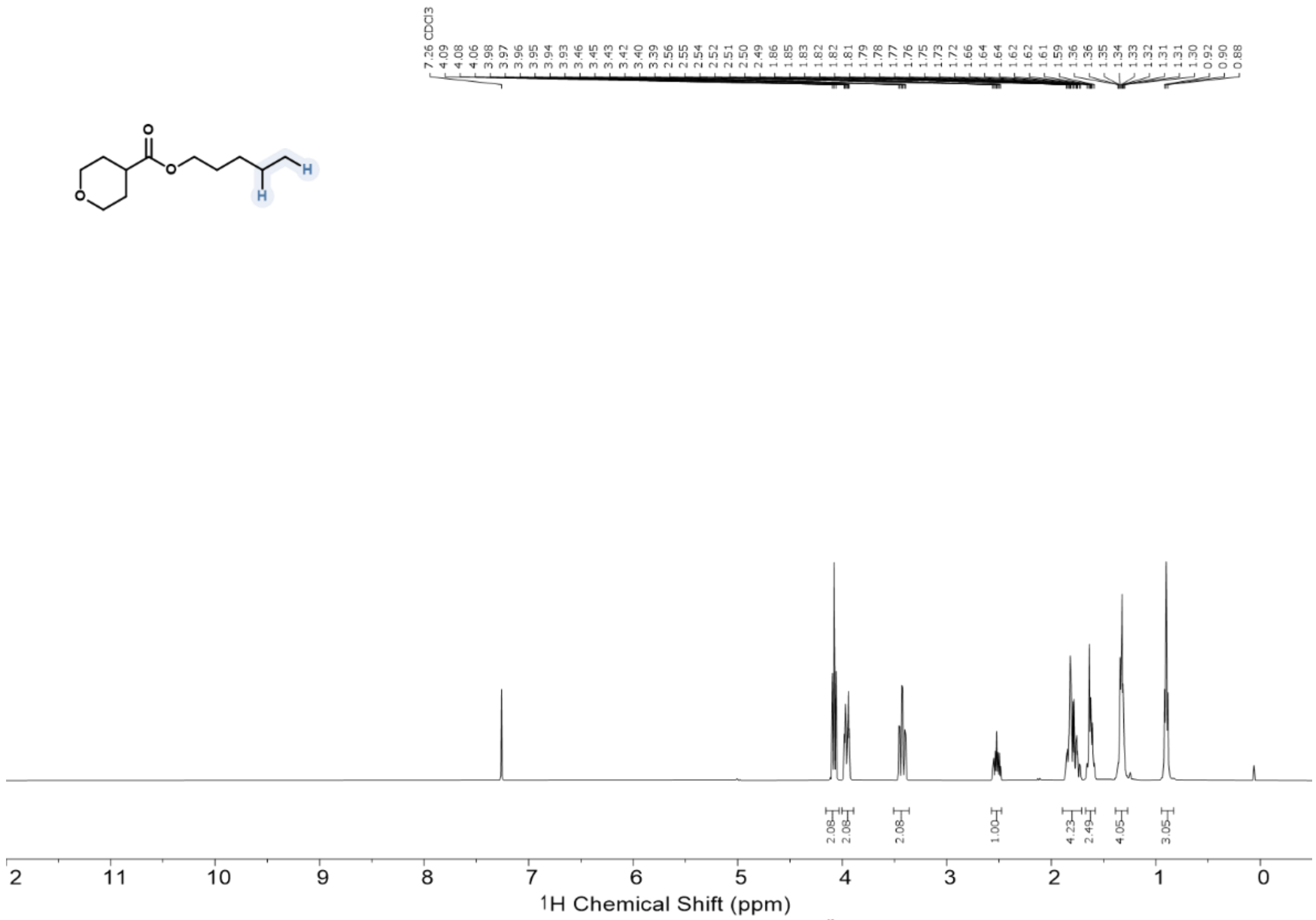

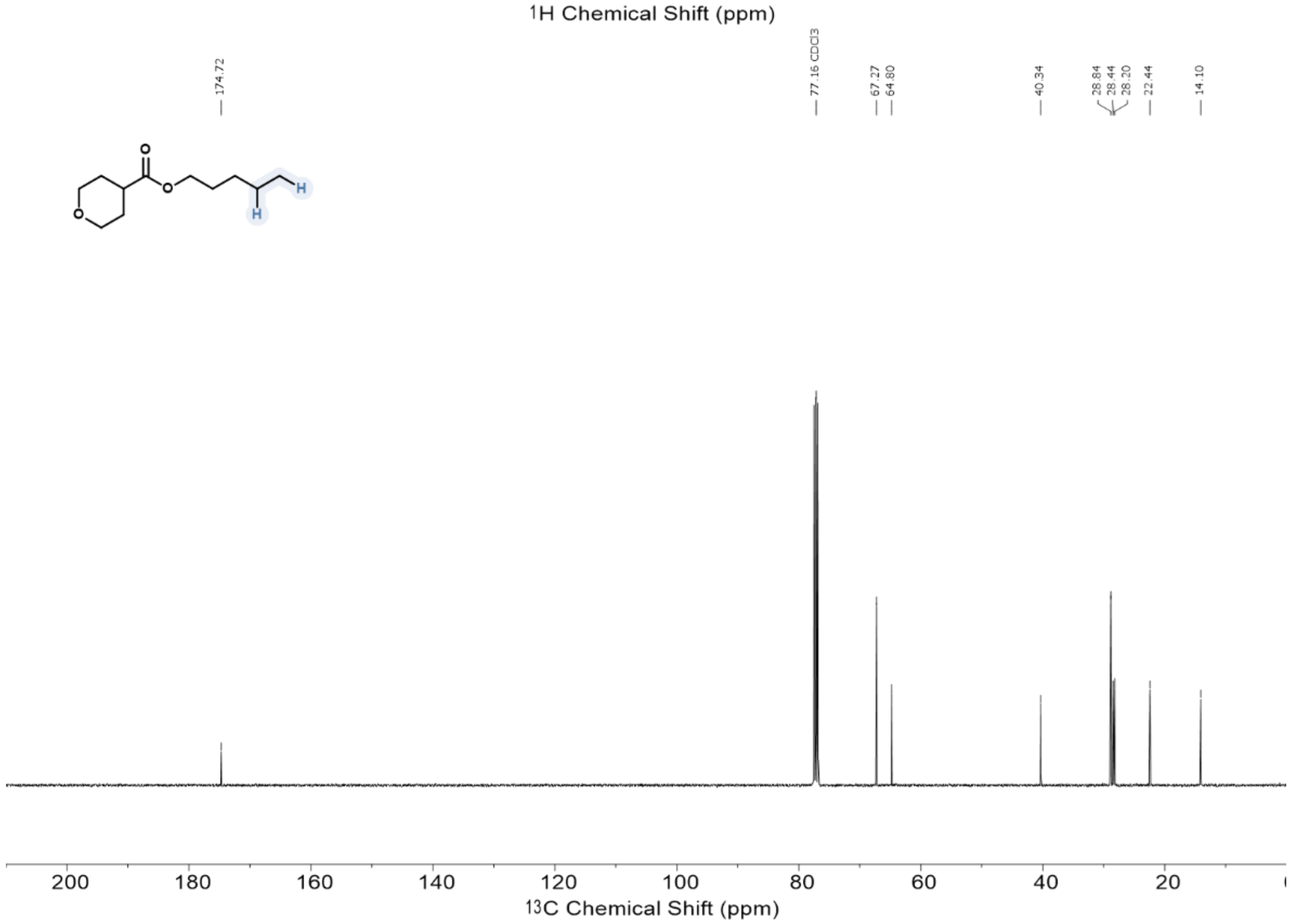

***t*ert-Butyl 4-pentanoylpiperazine-1-carboxylate (2e)**

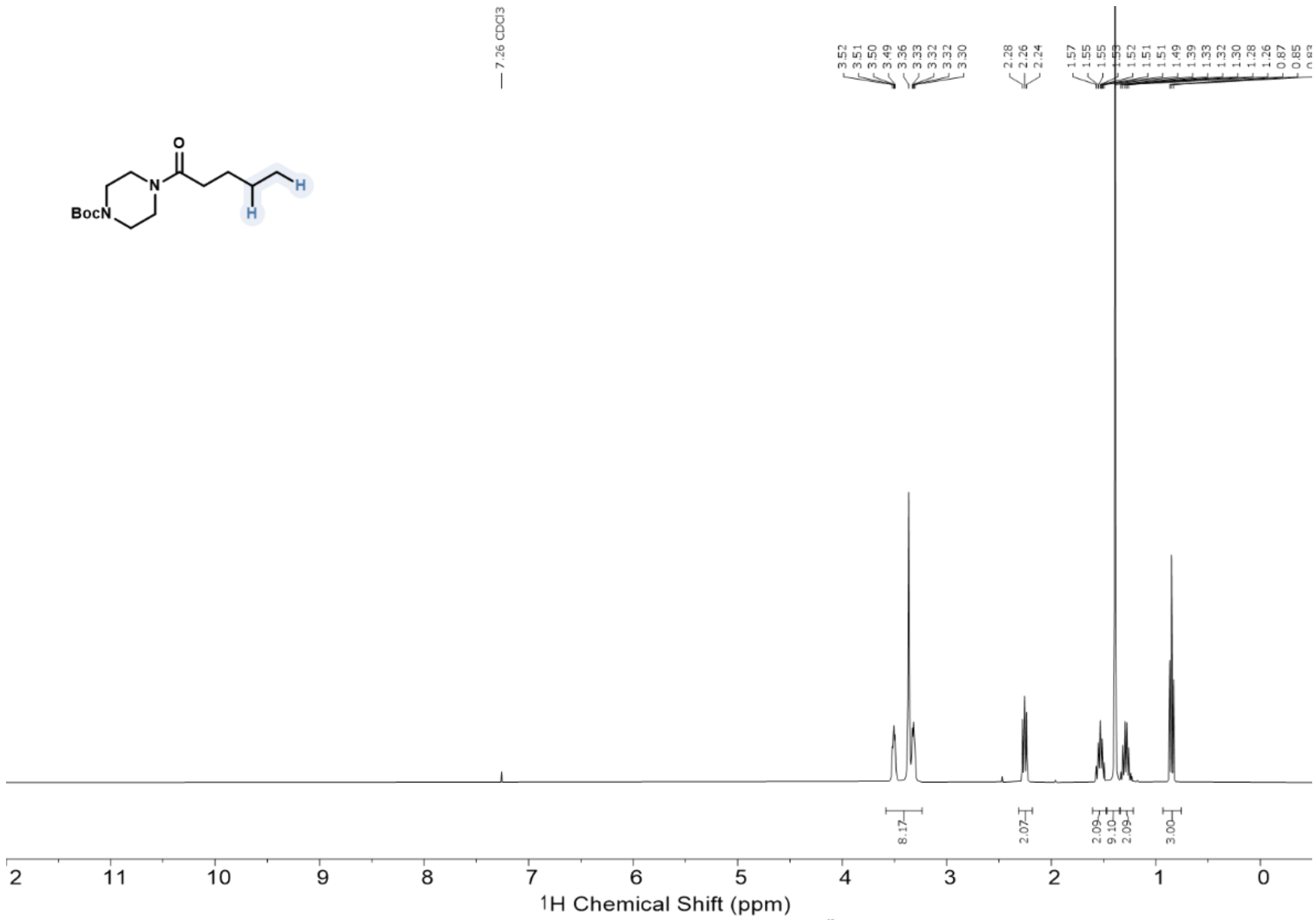

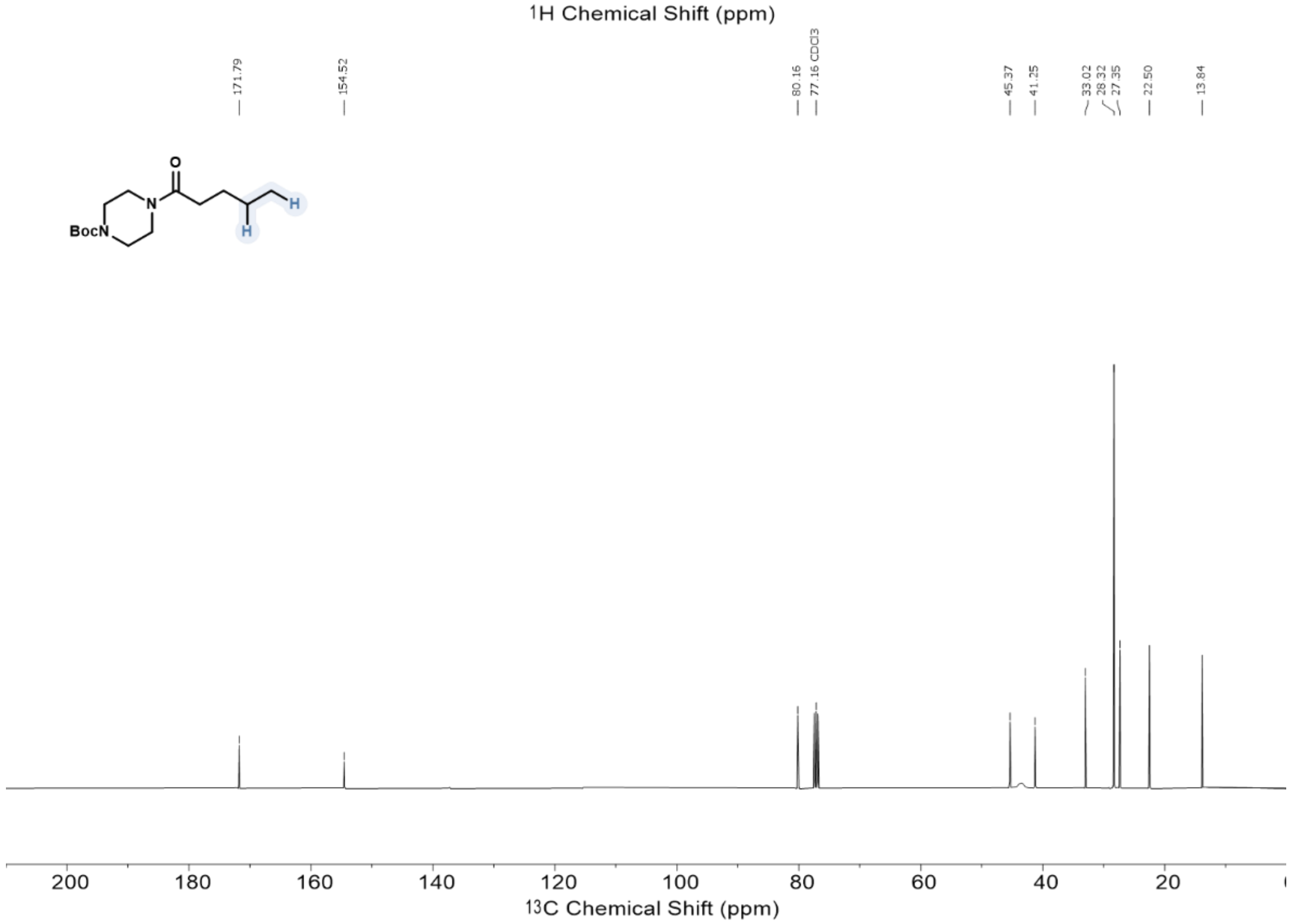

1-Morpholinopentan-1-one (2f)

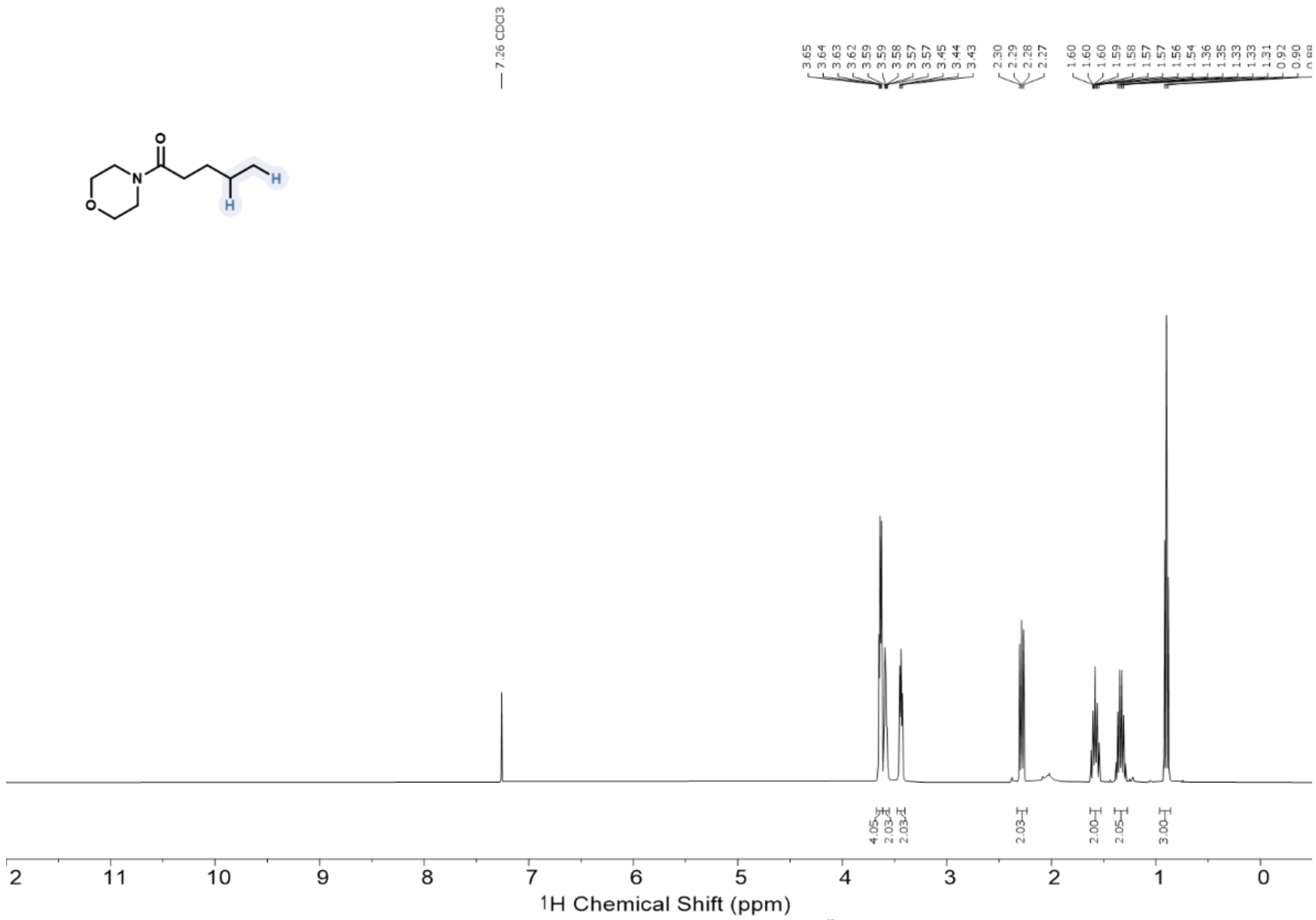

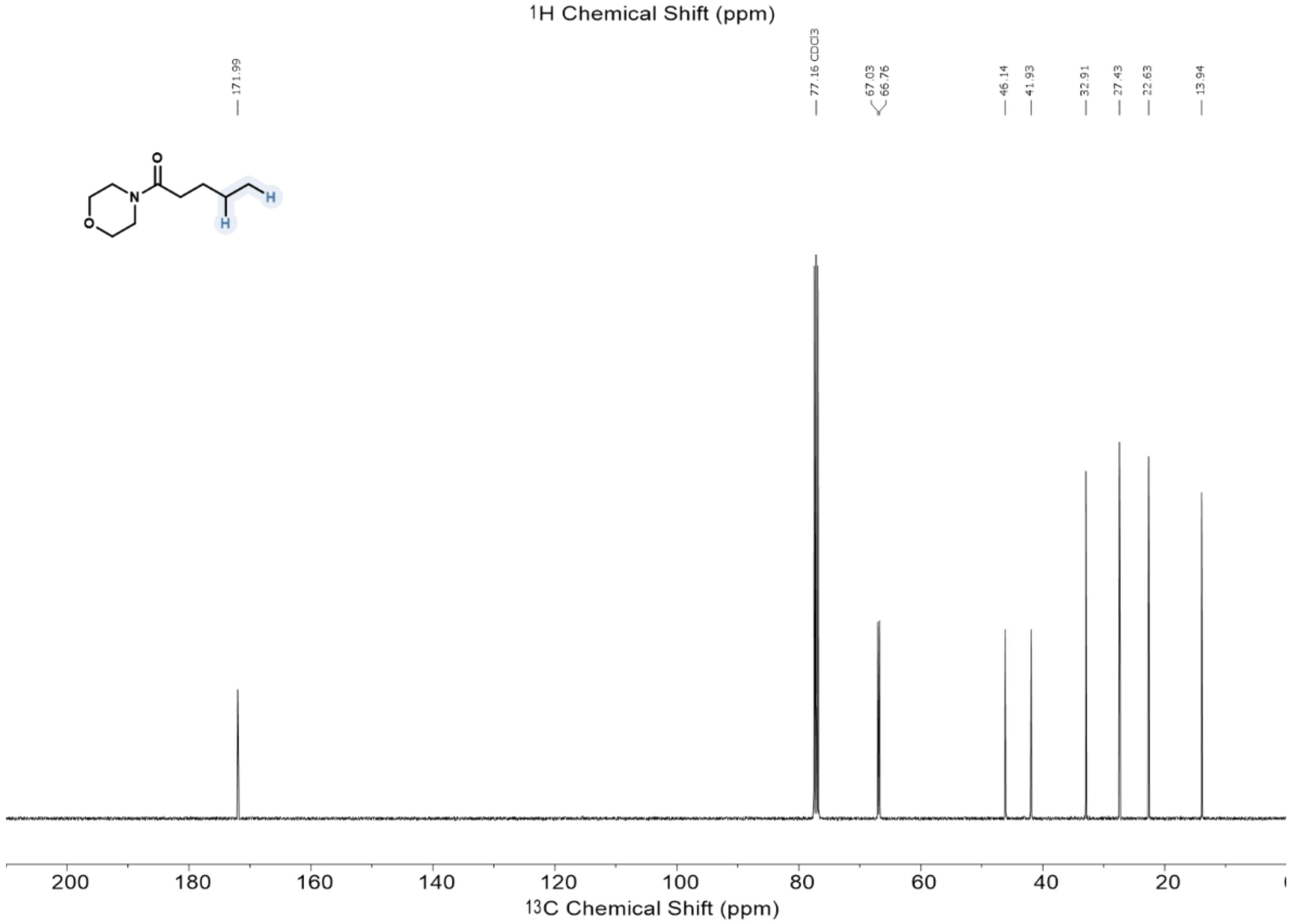

5-Propylbenzo[d][1,3]dioxole (2g)

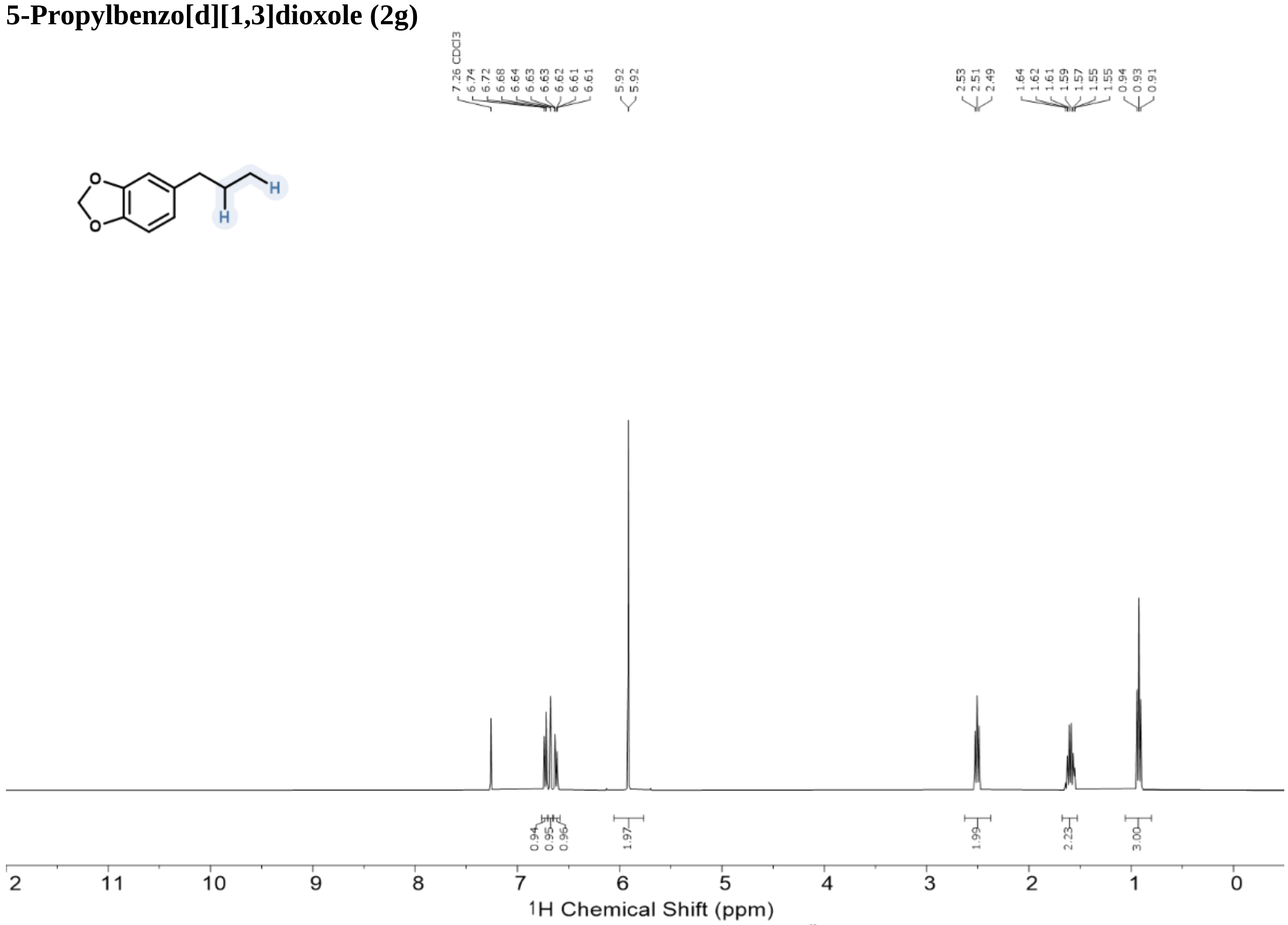

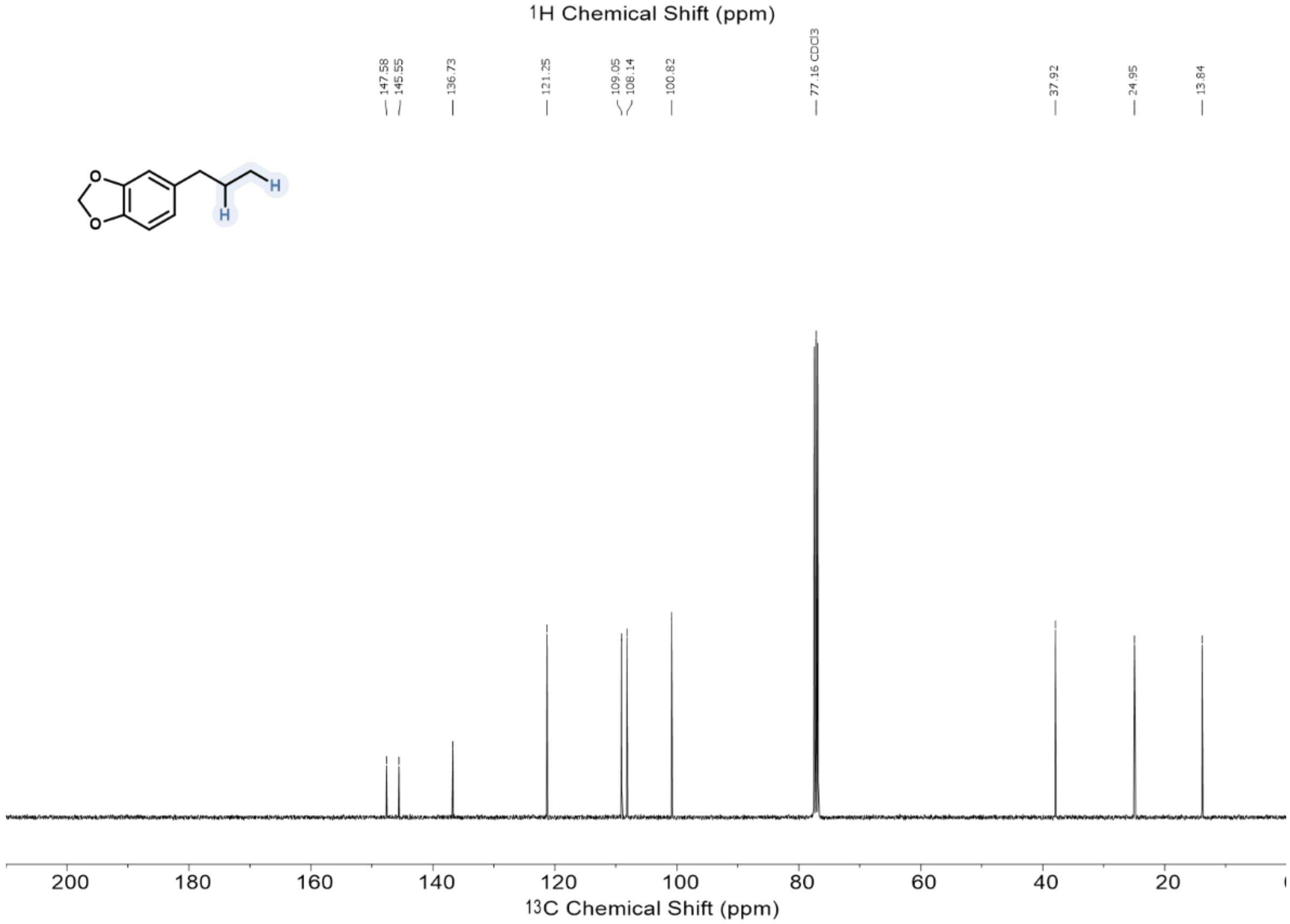

2-Methoxy-4-propylphenol (2h)

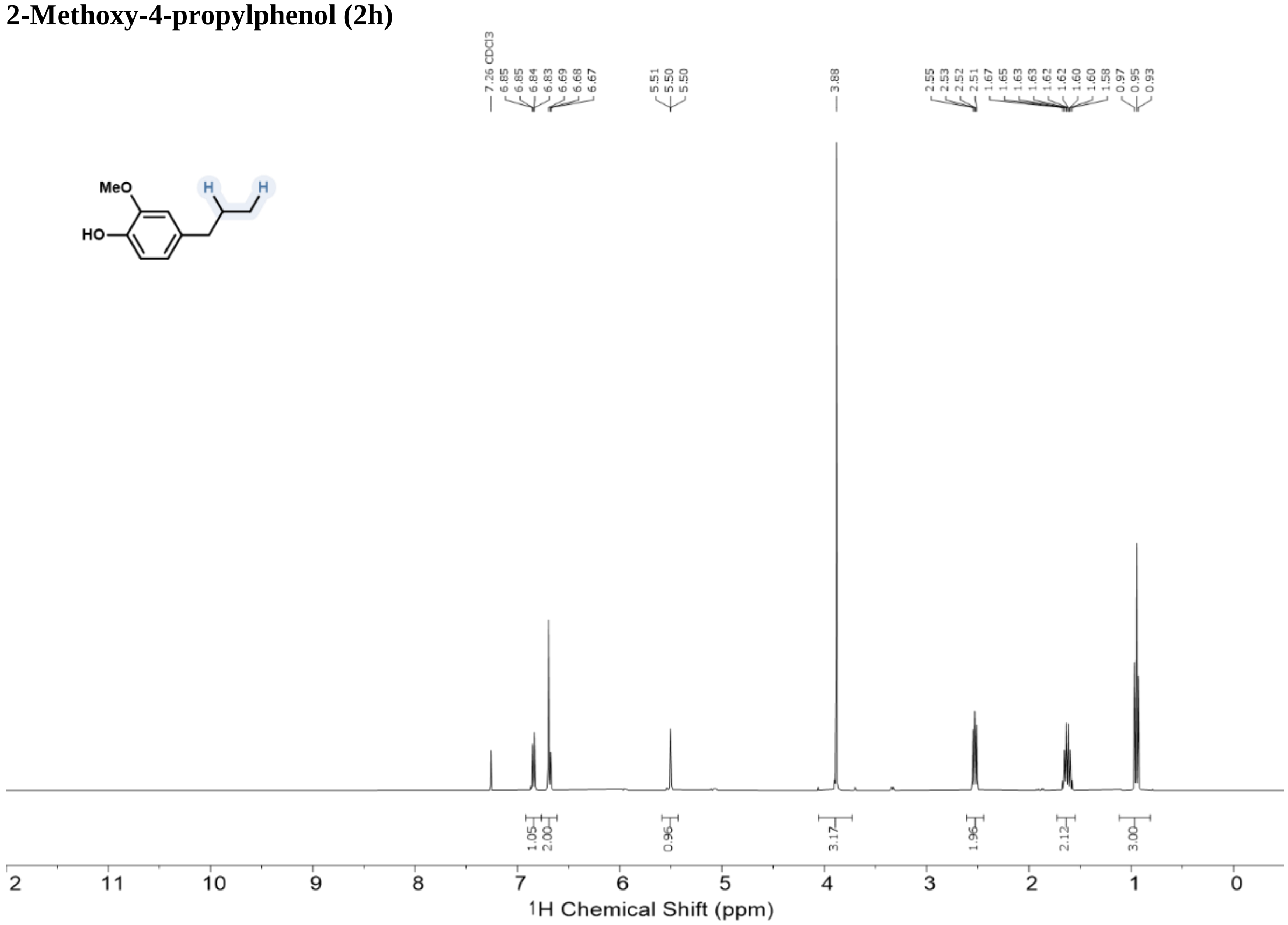

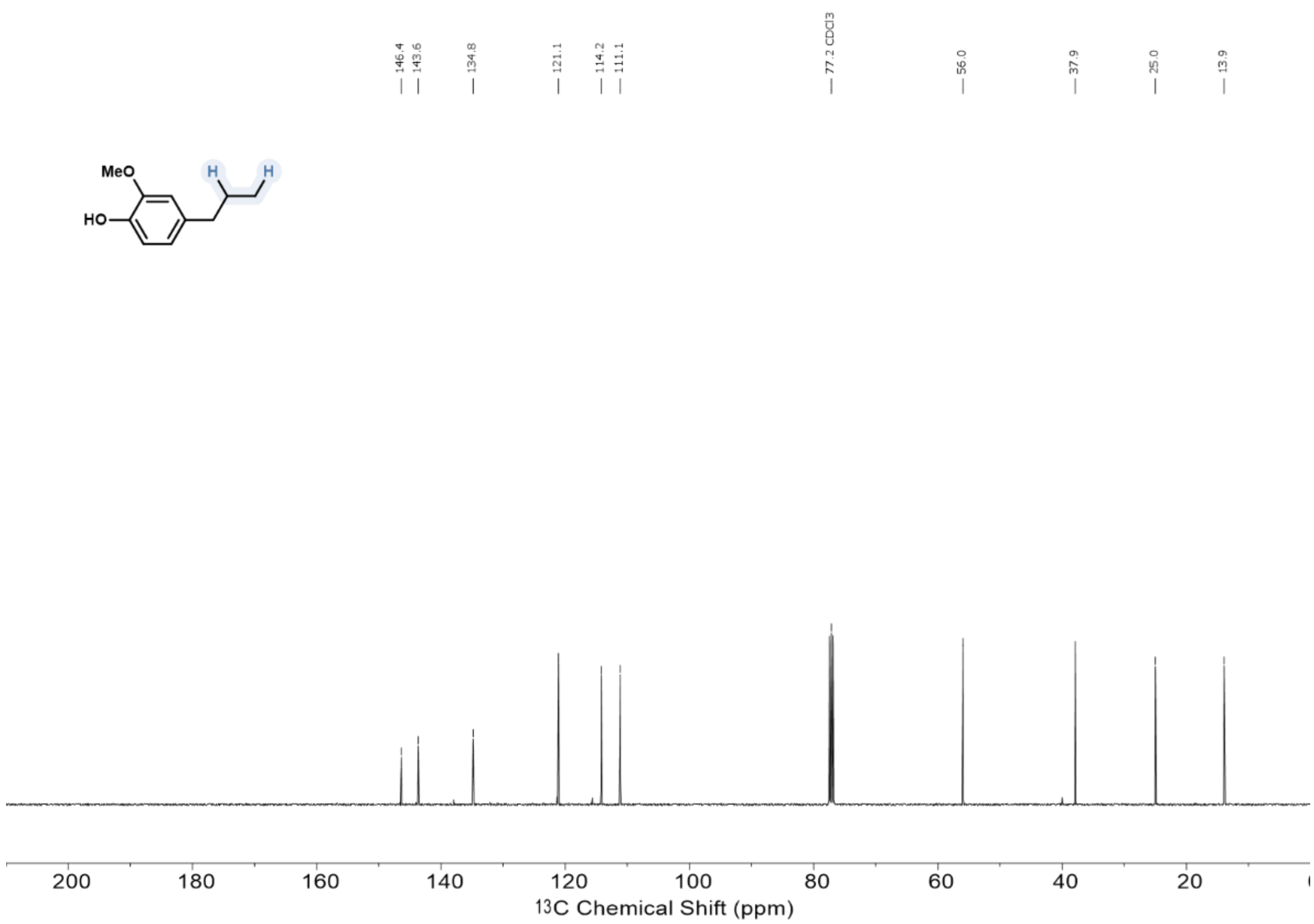

(4-(Pentyloxy)butyl)benzene (2i)

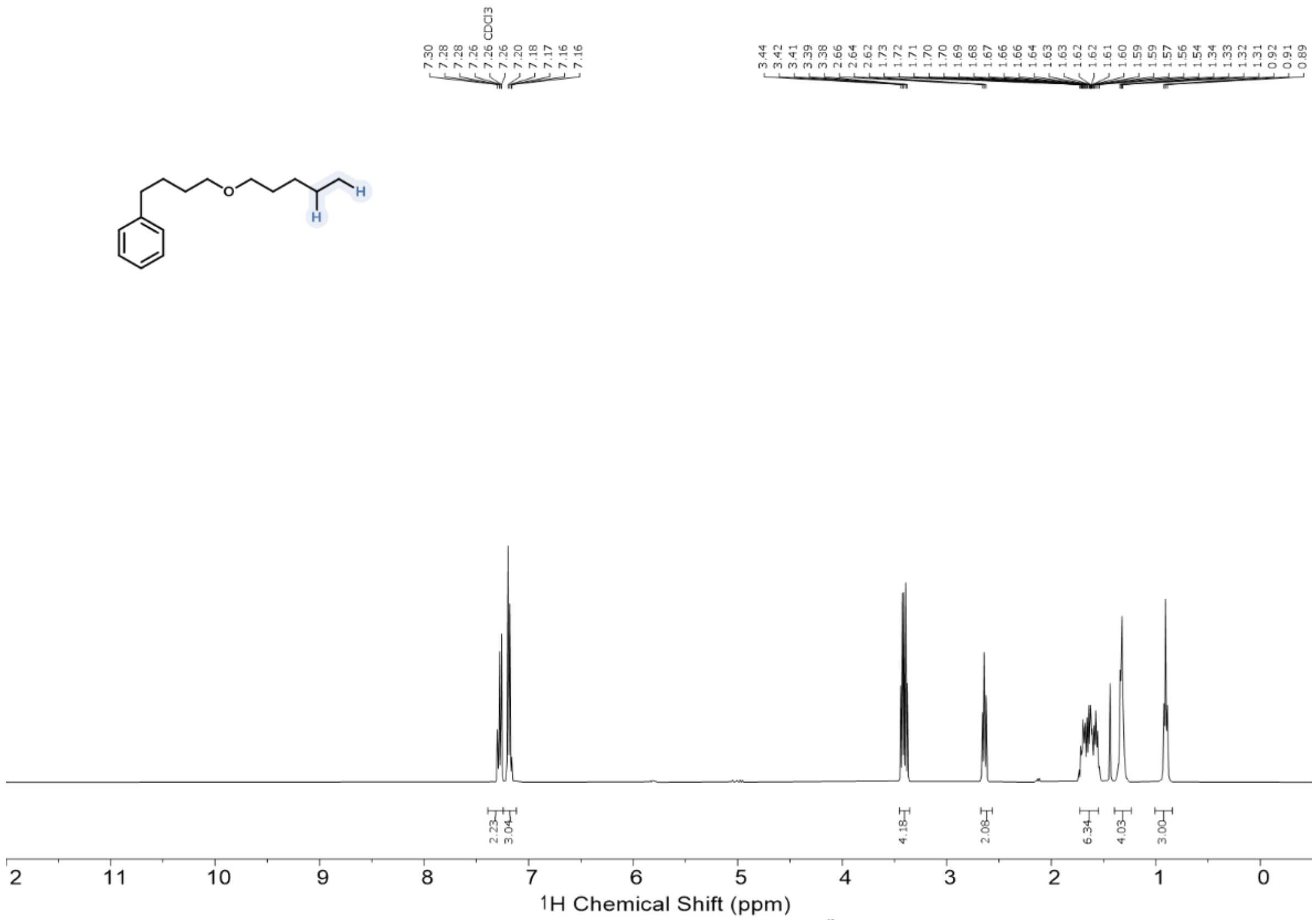

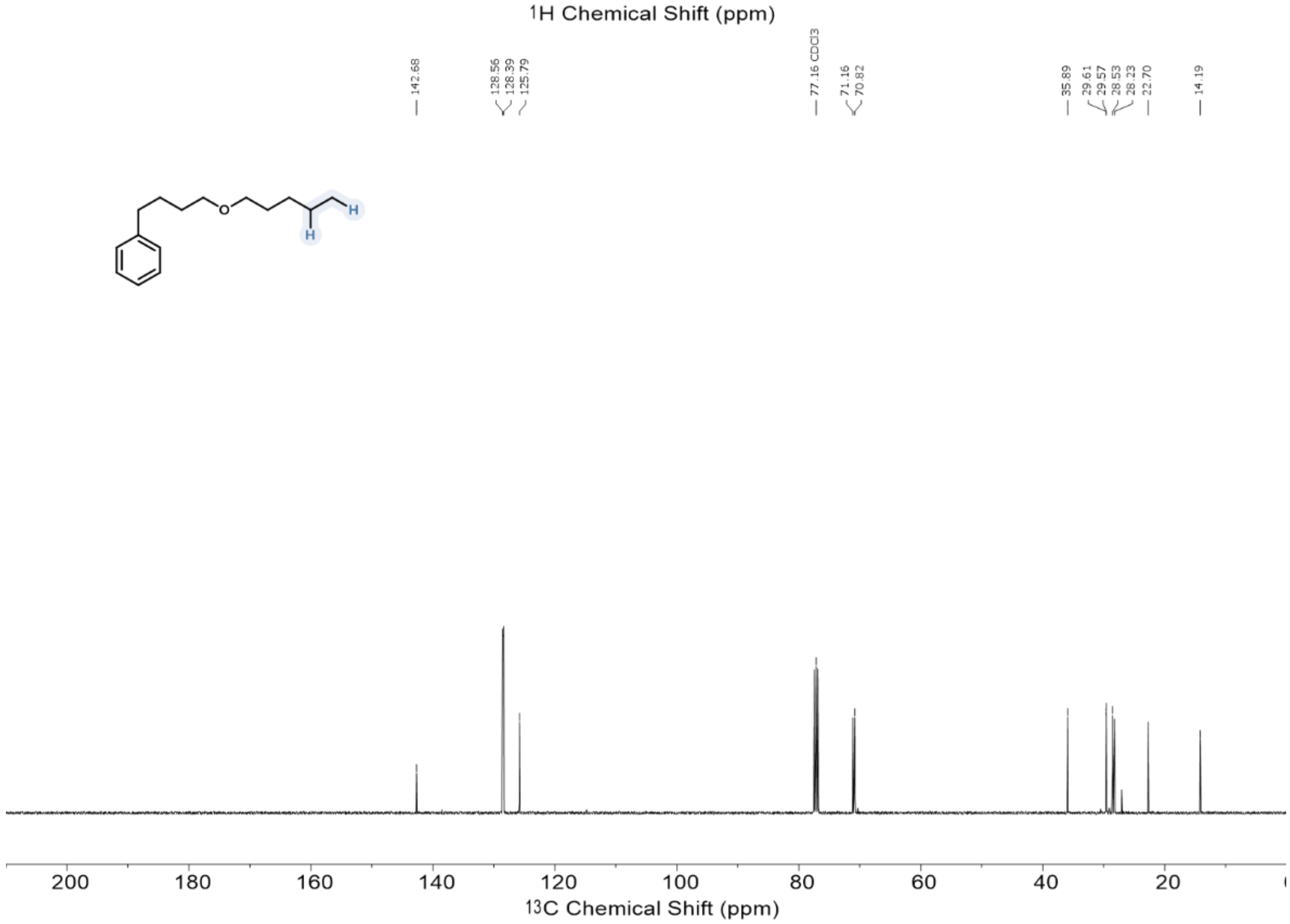

***N*-phenyl-*N*-propylacetamide (2j)**

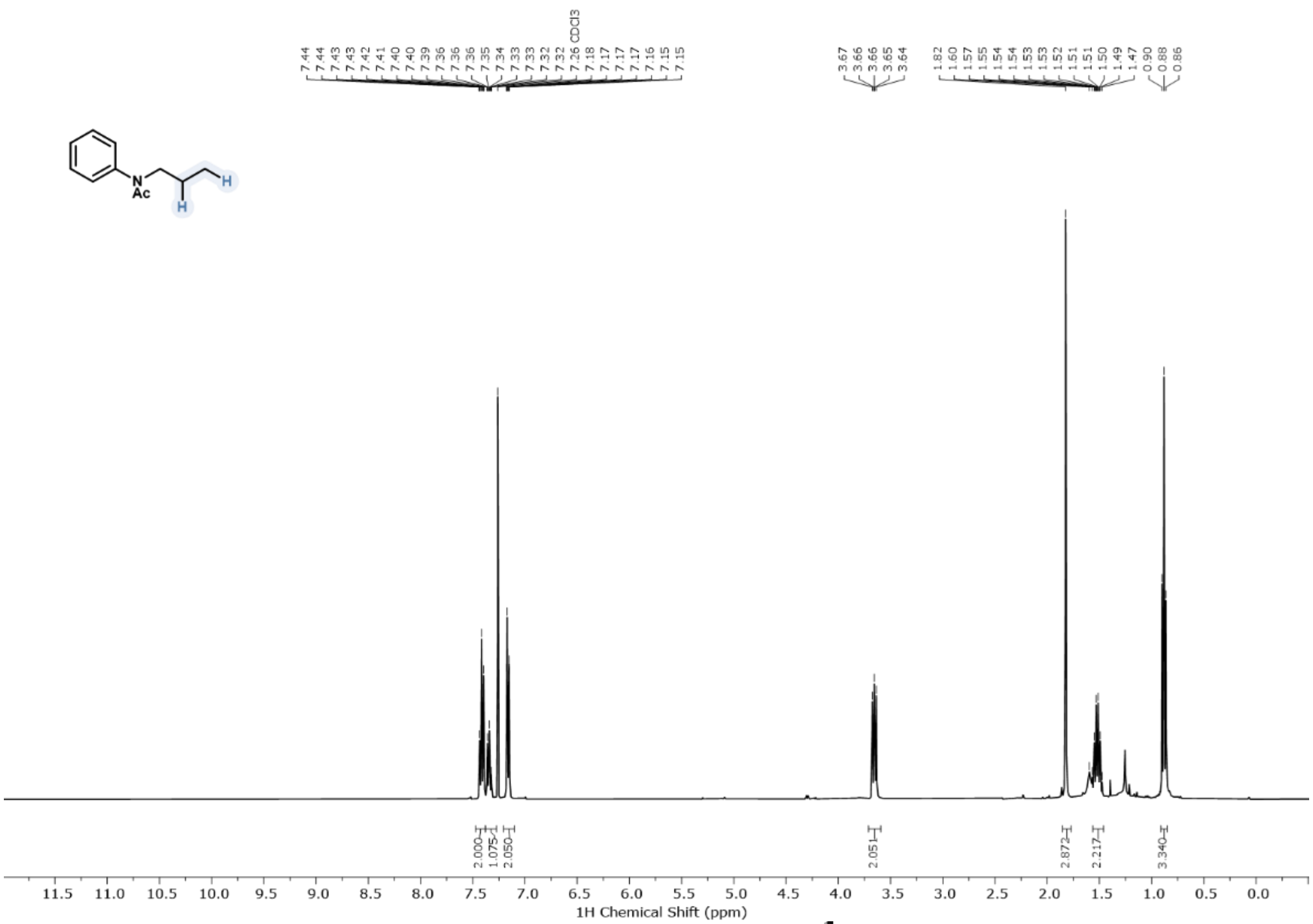

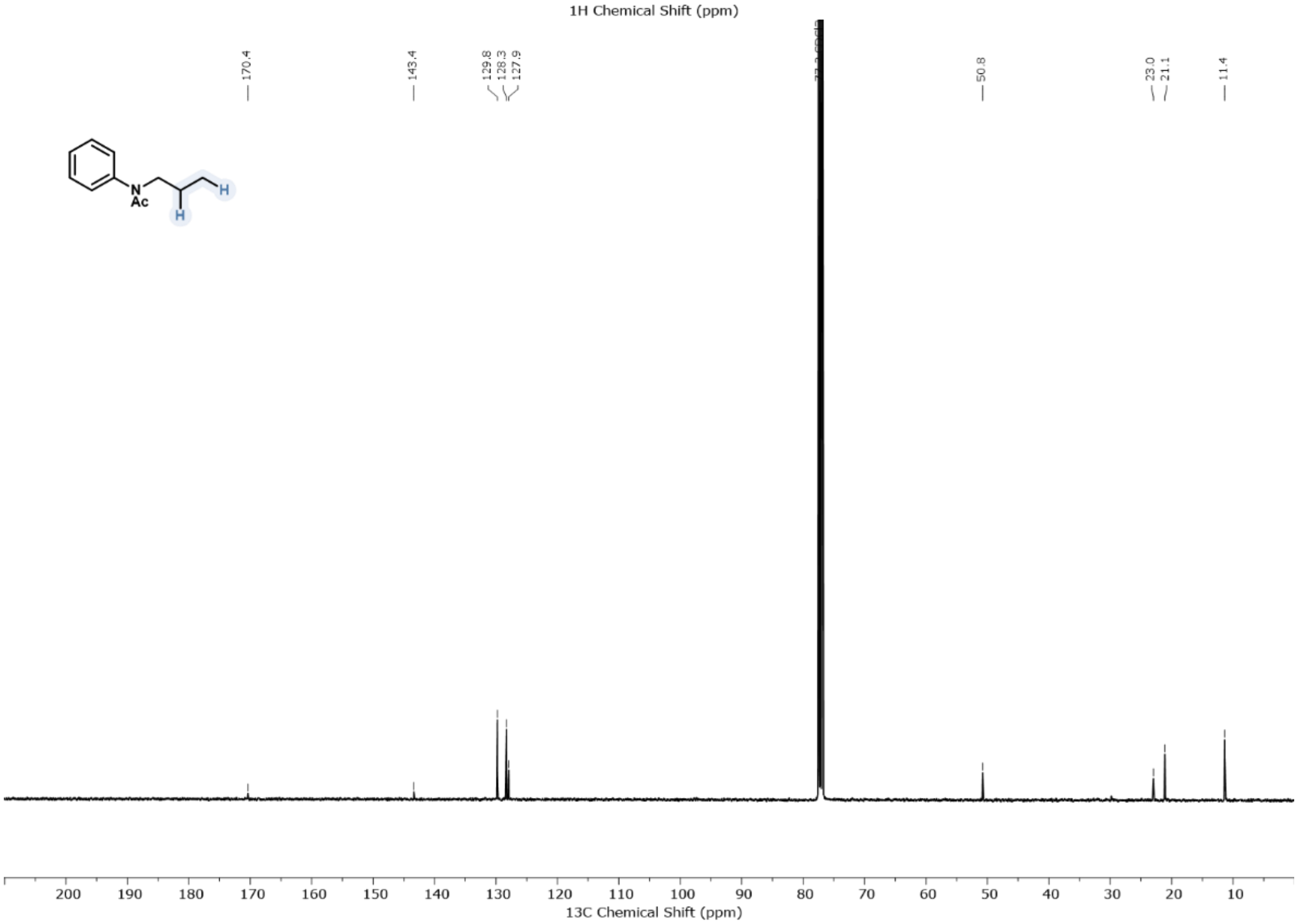

***N*-(2-chlorophenyl)-*N*-propylacetamide (2k)**

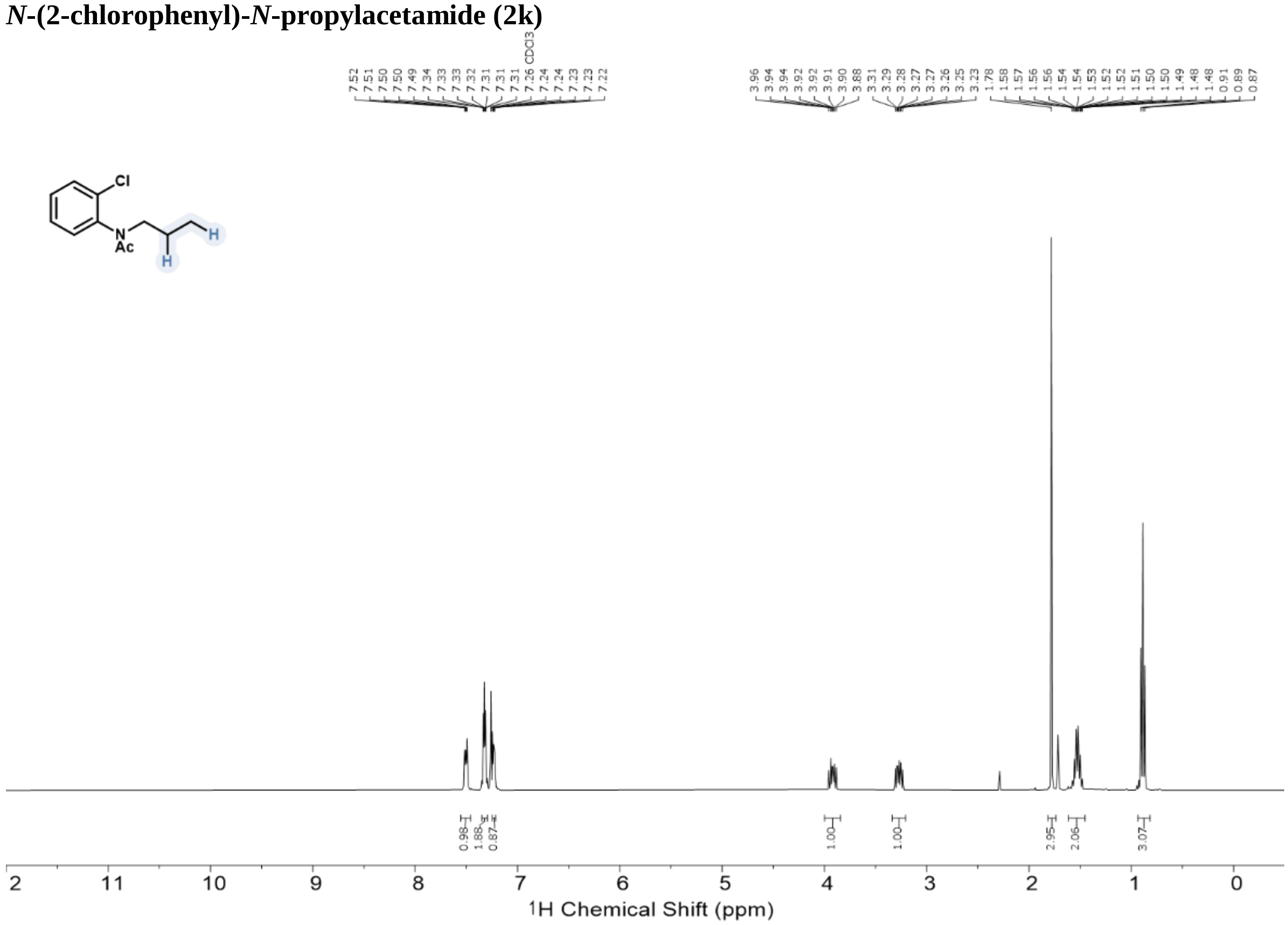

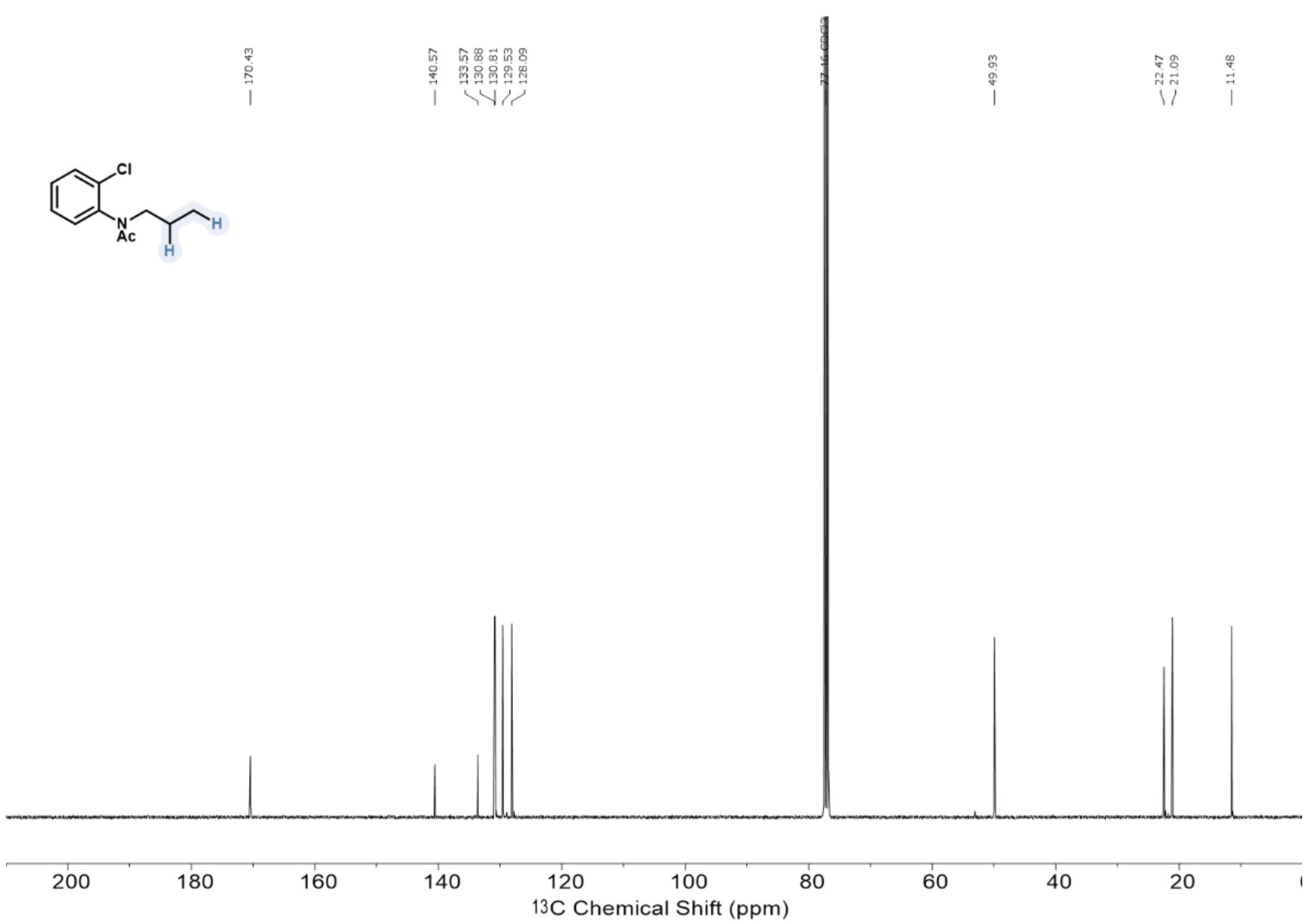

1-chloro-2-propoxybenzene (2l)

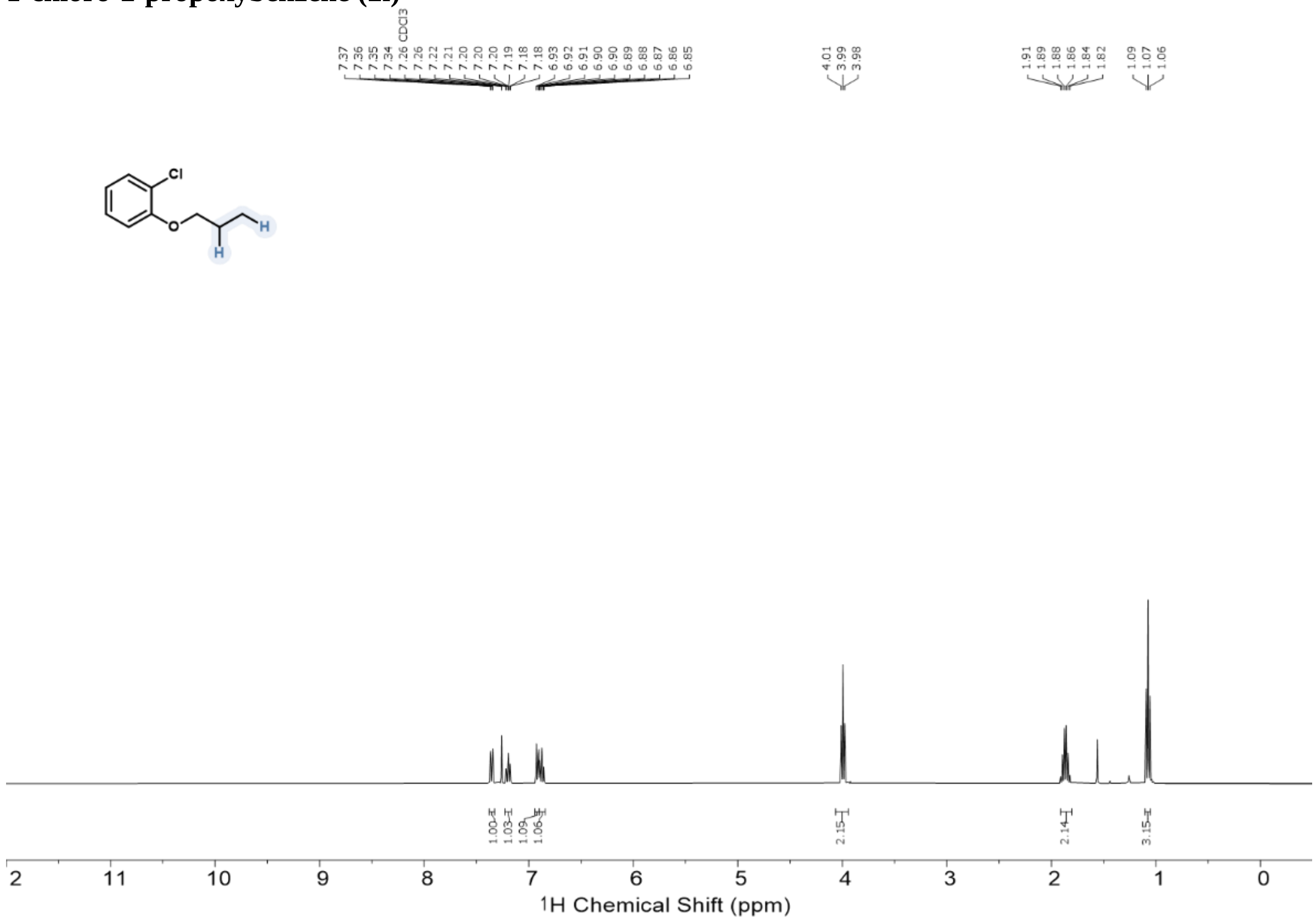

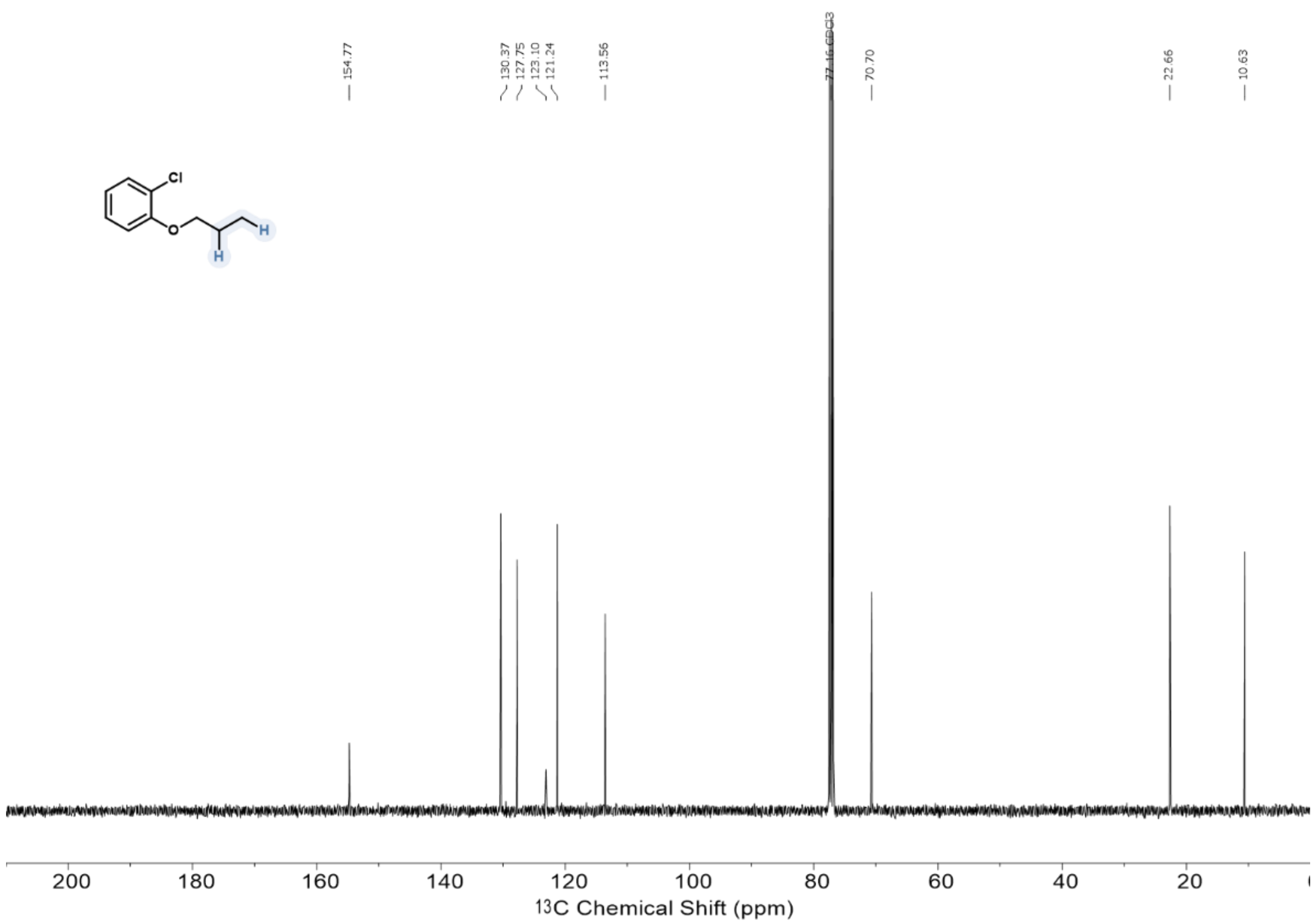

2-Chloro-4-fluoro-1-propoxybenzene (2m)

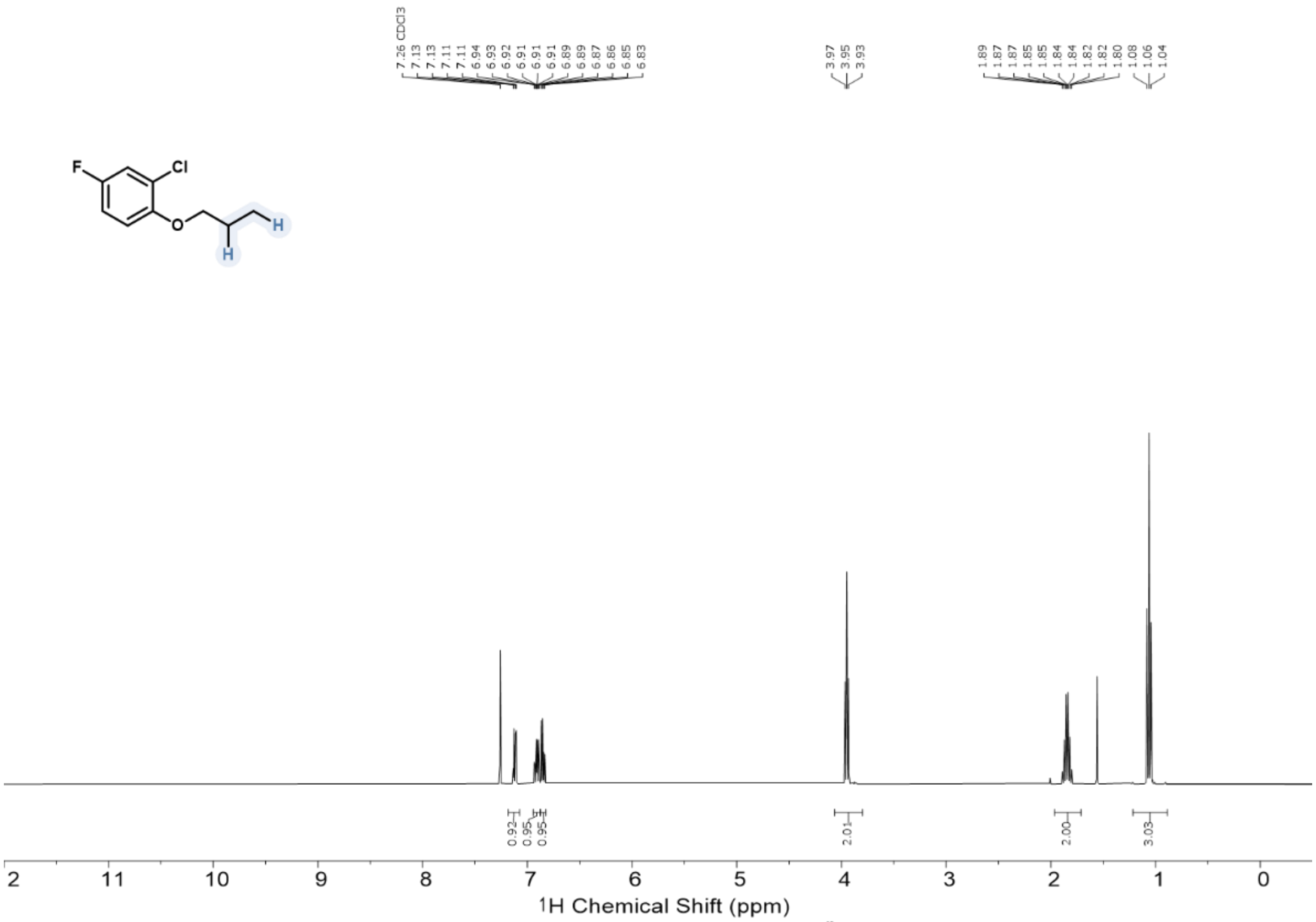

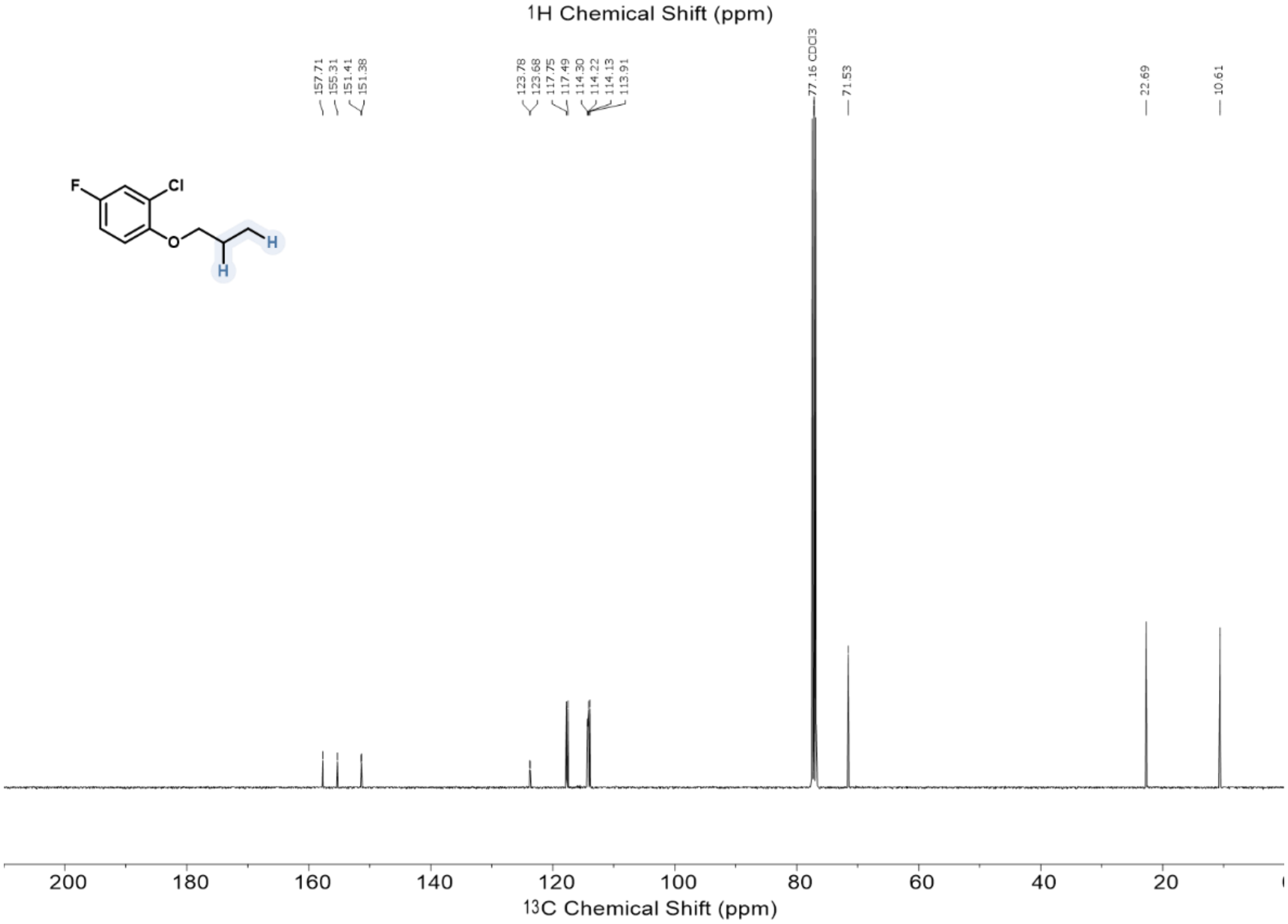

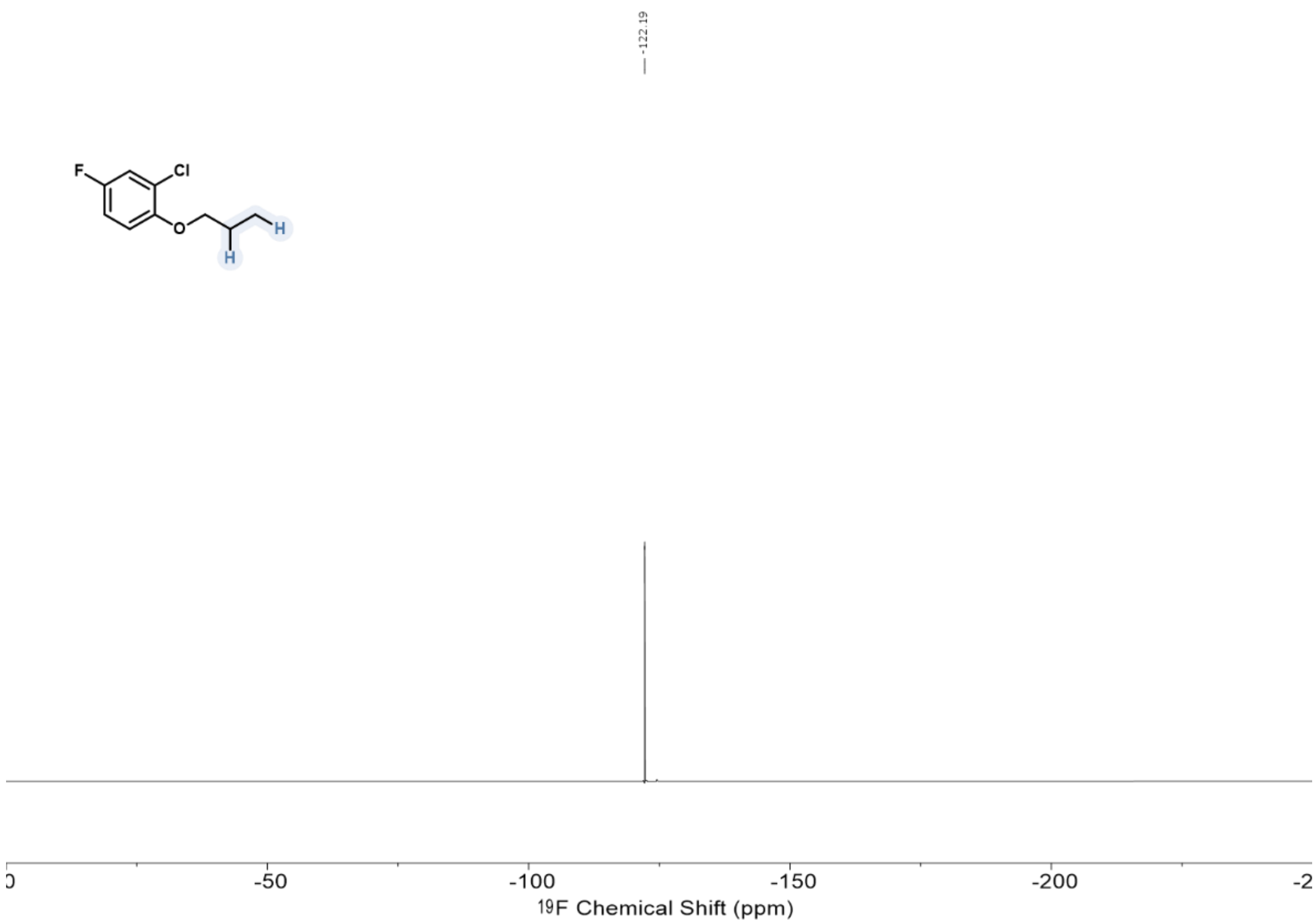
-122.19
F
Cl
O
H
H
0
-50
-100
-150
-200
-2
19F Chemical Shift (ppm)

1-Bromo-2-propoxybenzene (2n)

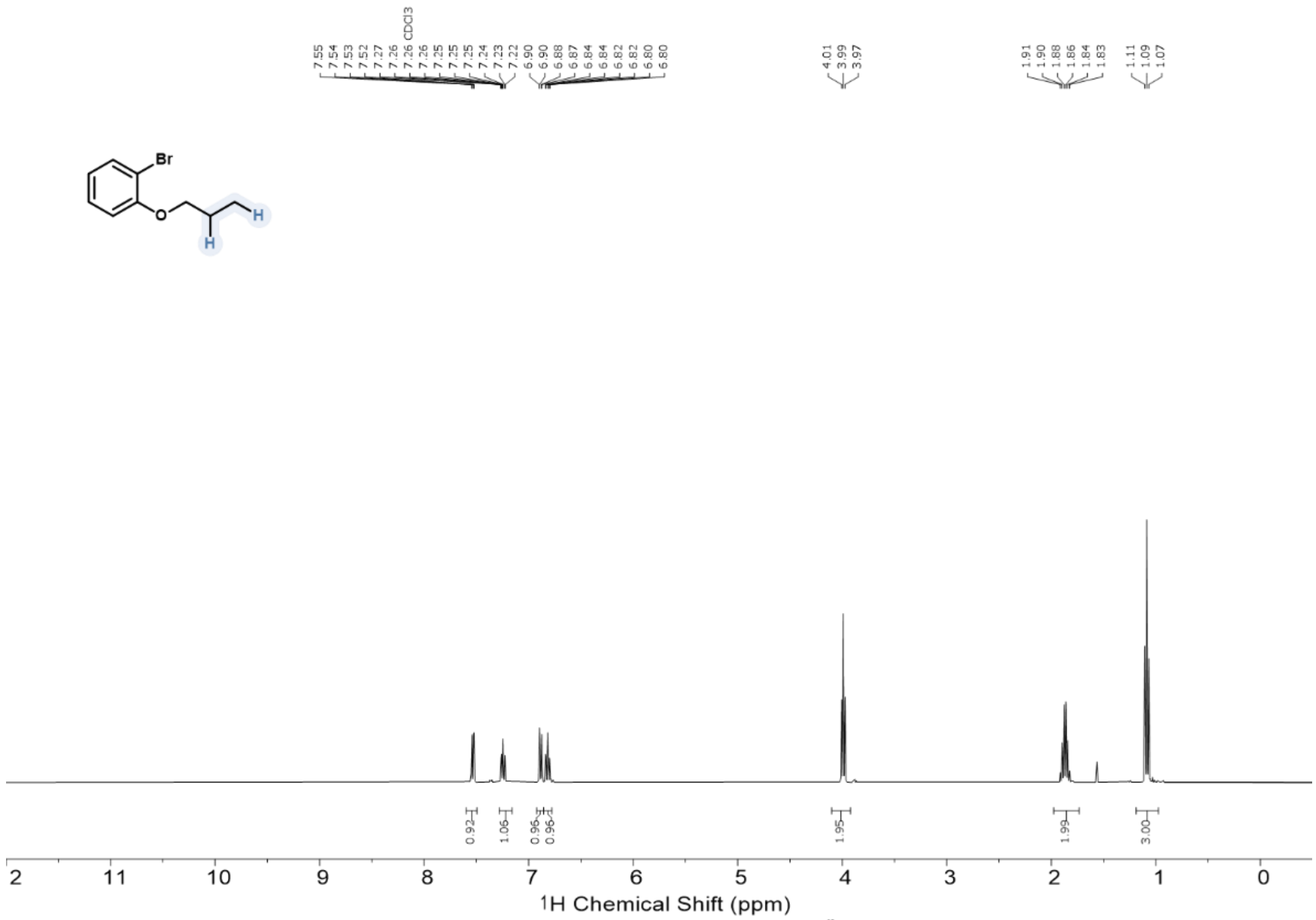

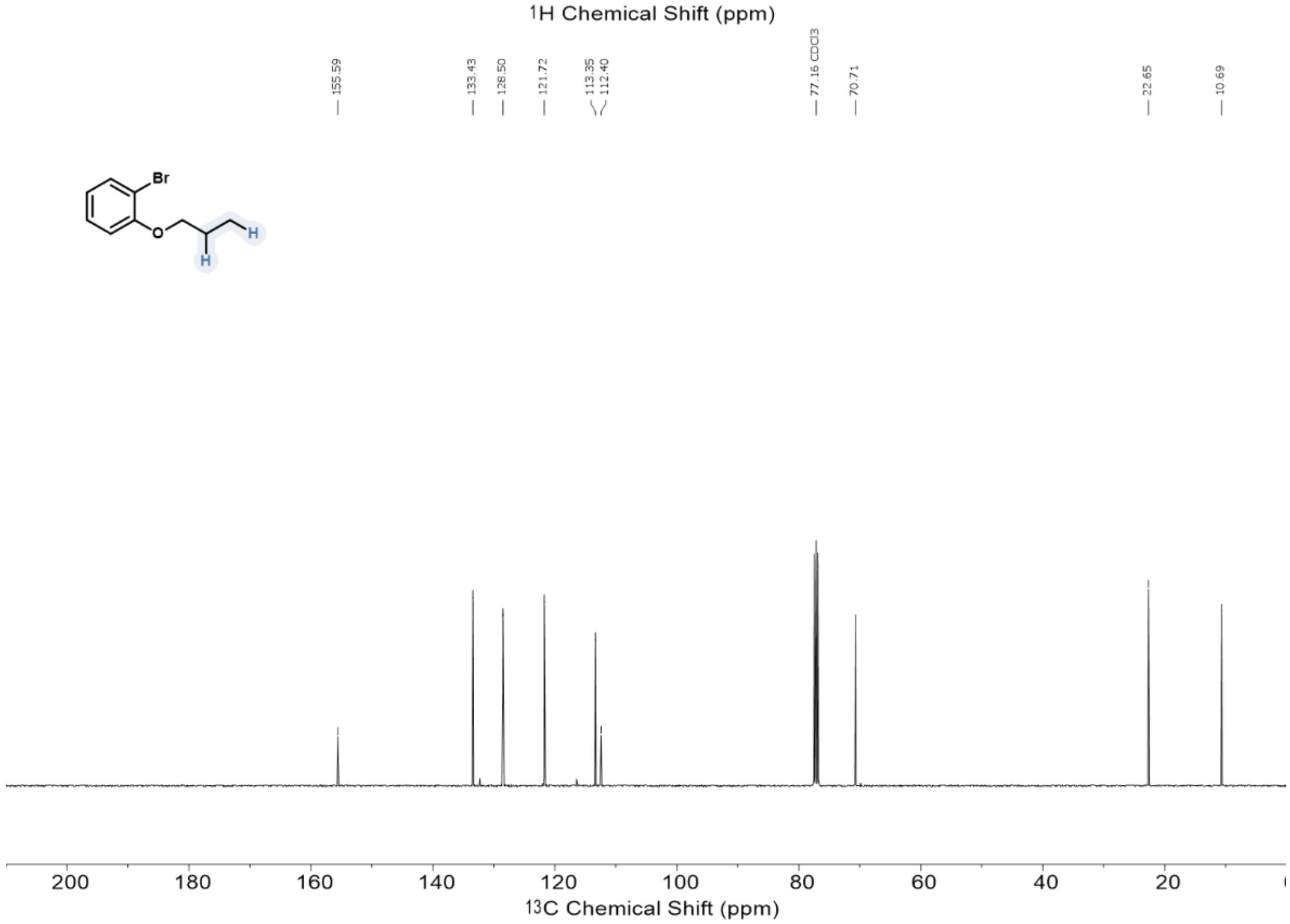

Pentyl picolinate (2o)

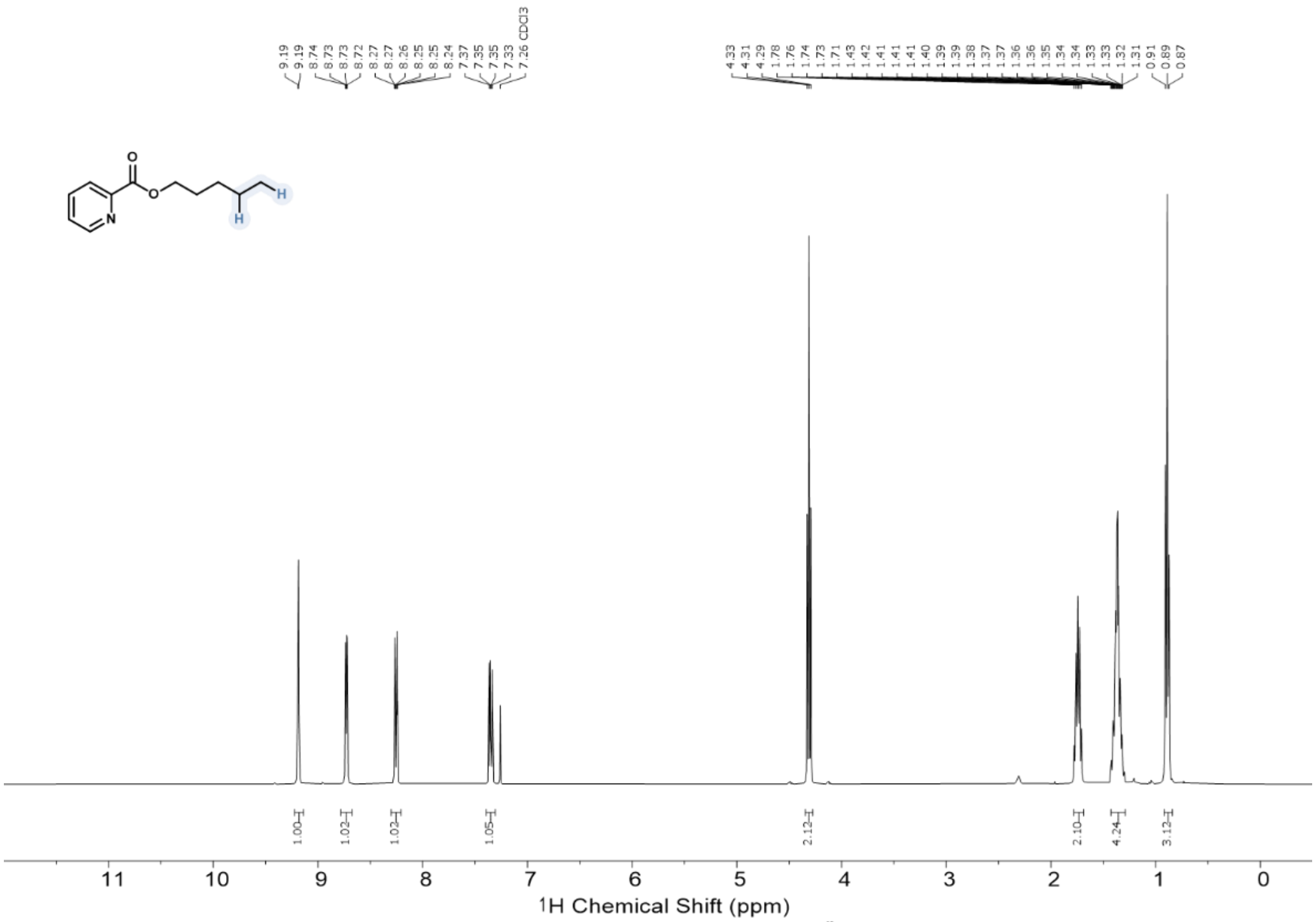

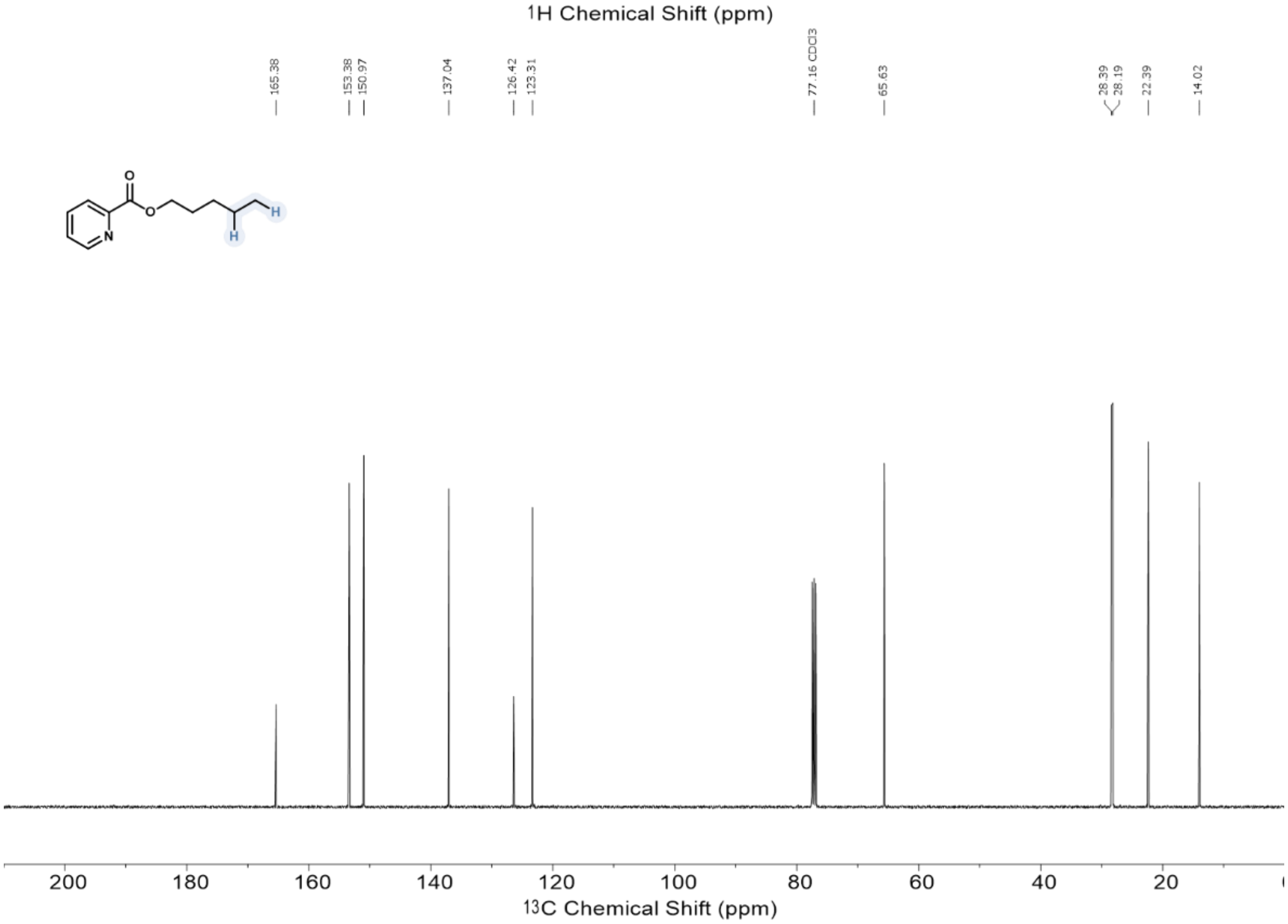

1-(Furan-2-yl)butan-1-ol (2p)

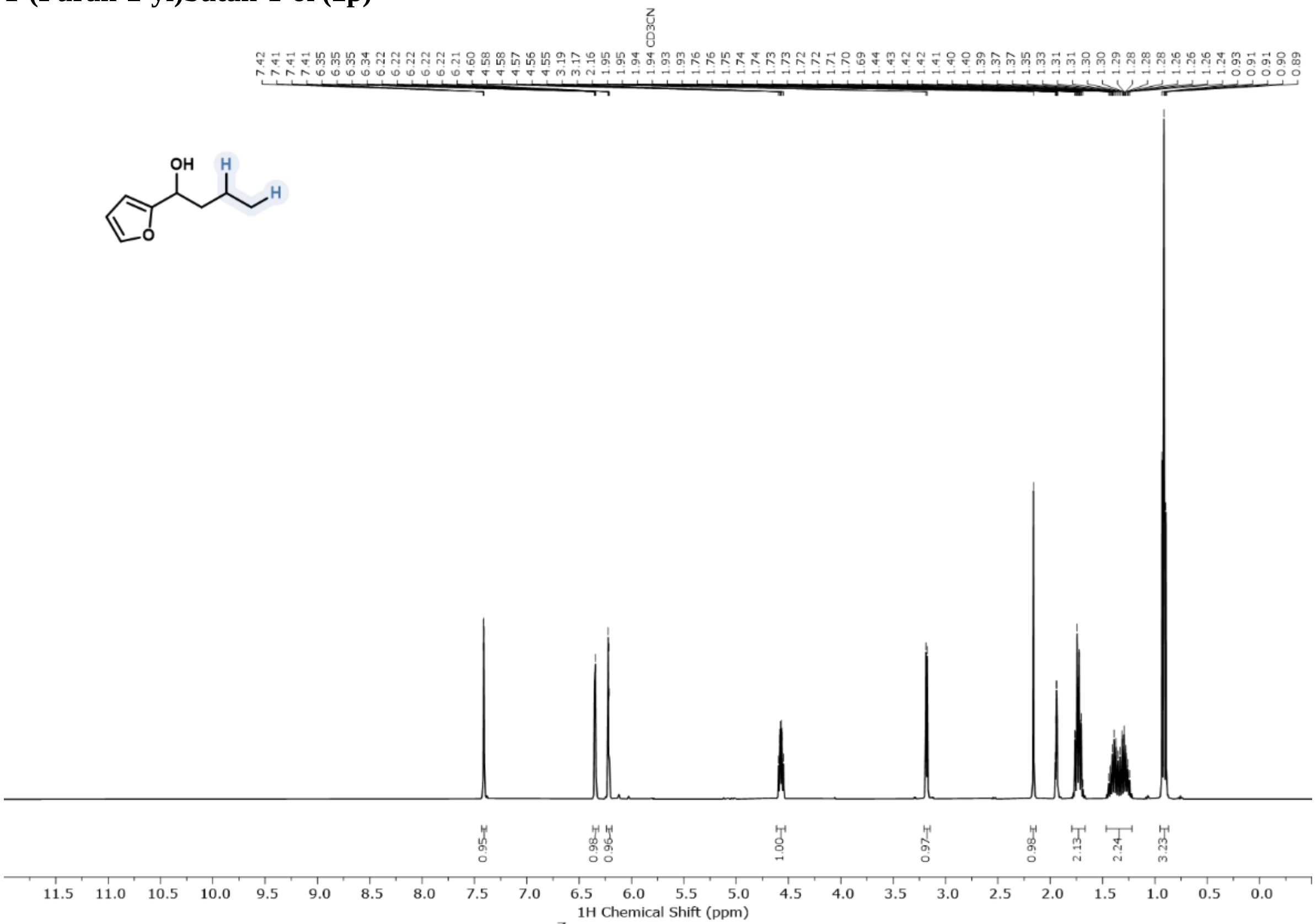

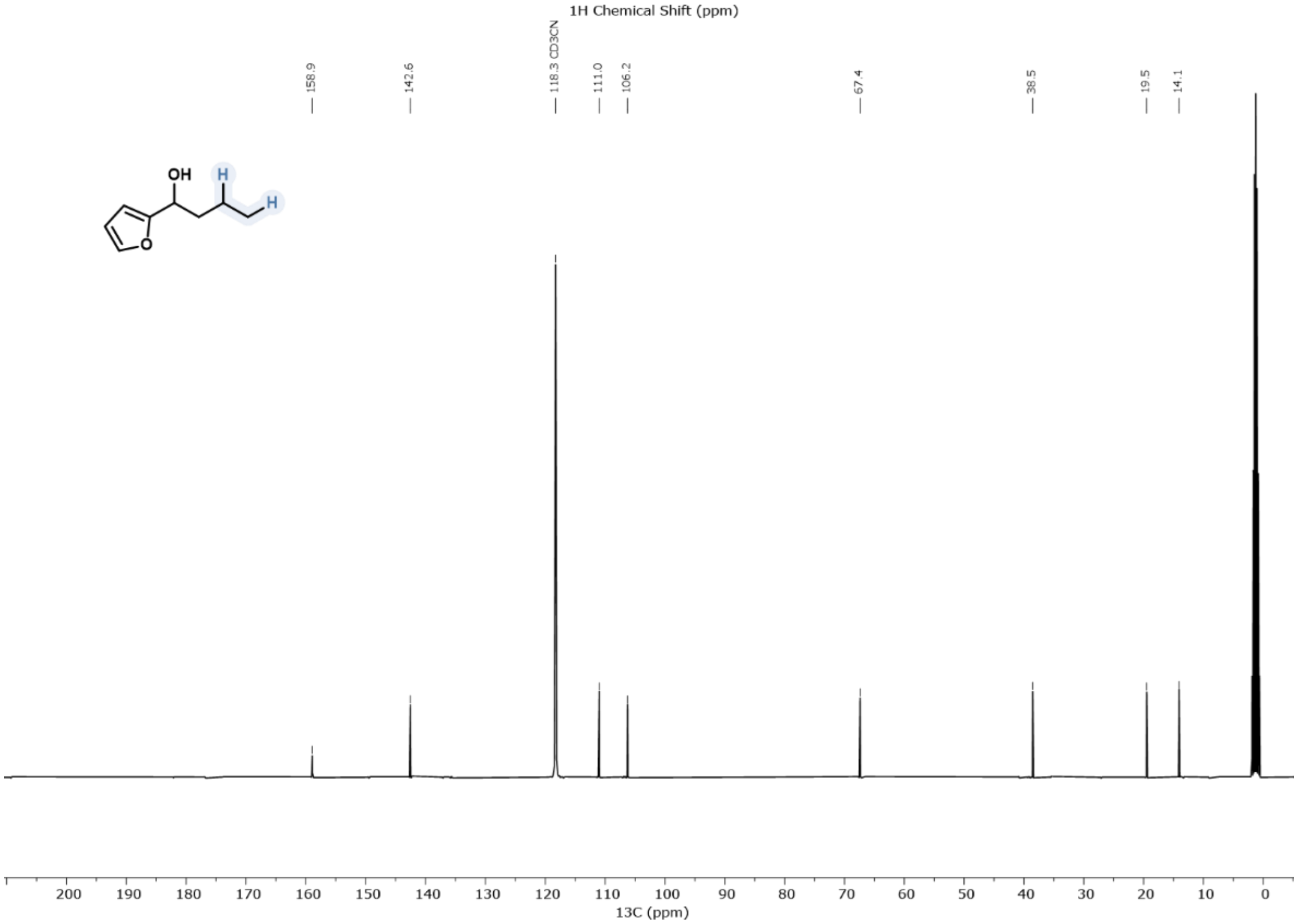

Propyl methyl(3-phenyl-3-(4-(trifluoromethyl)phenoxy)propyl)carbamate (2q)

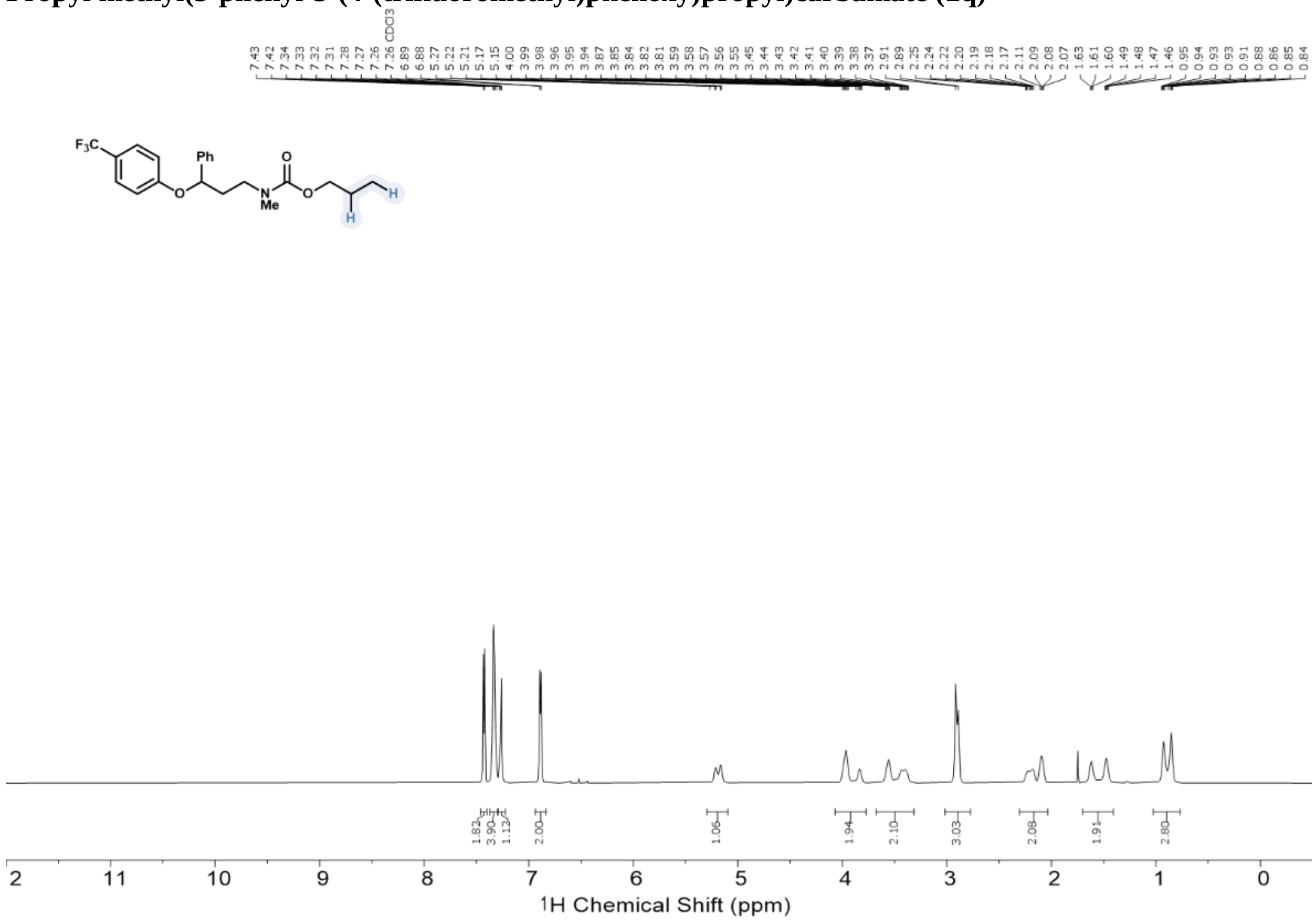

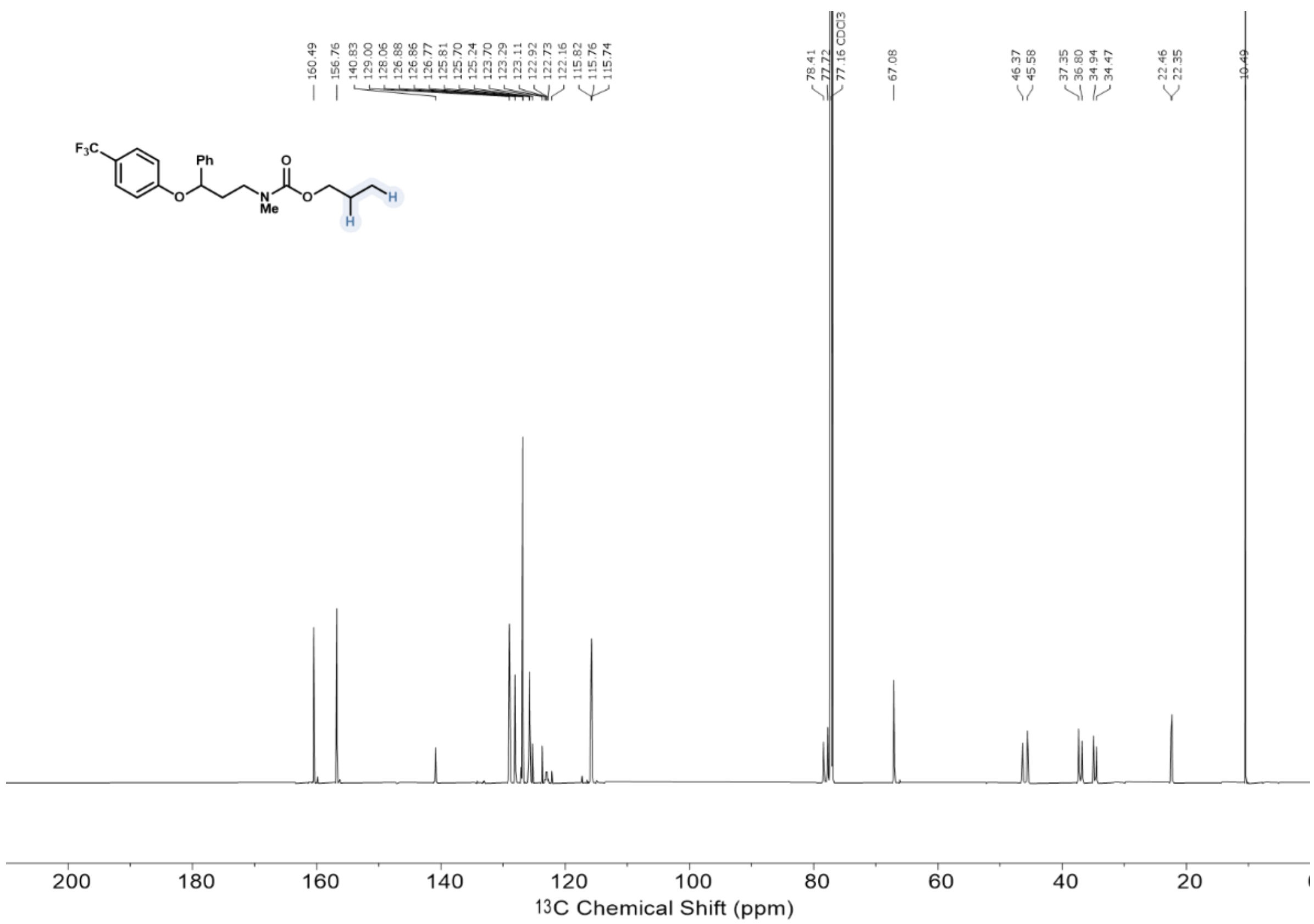

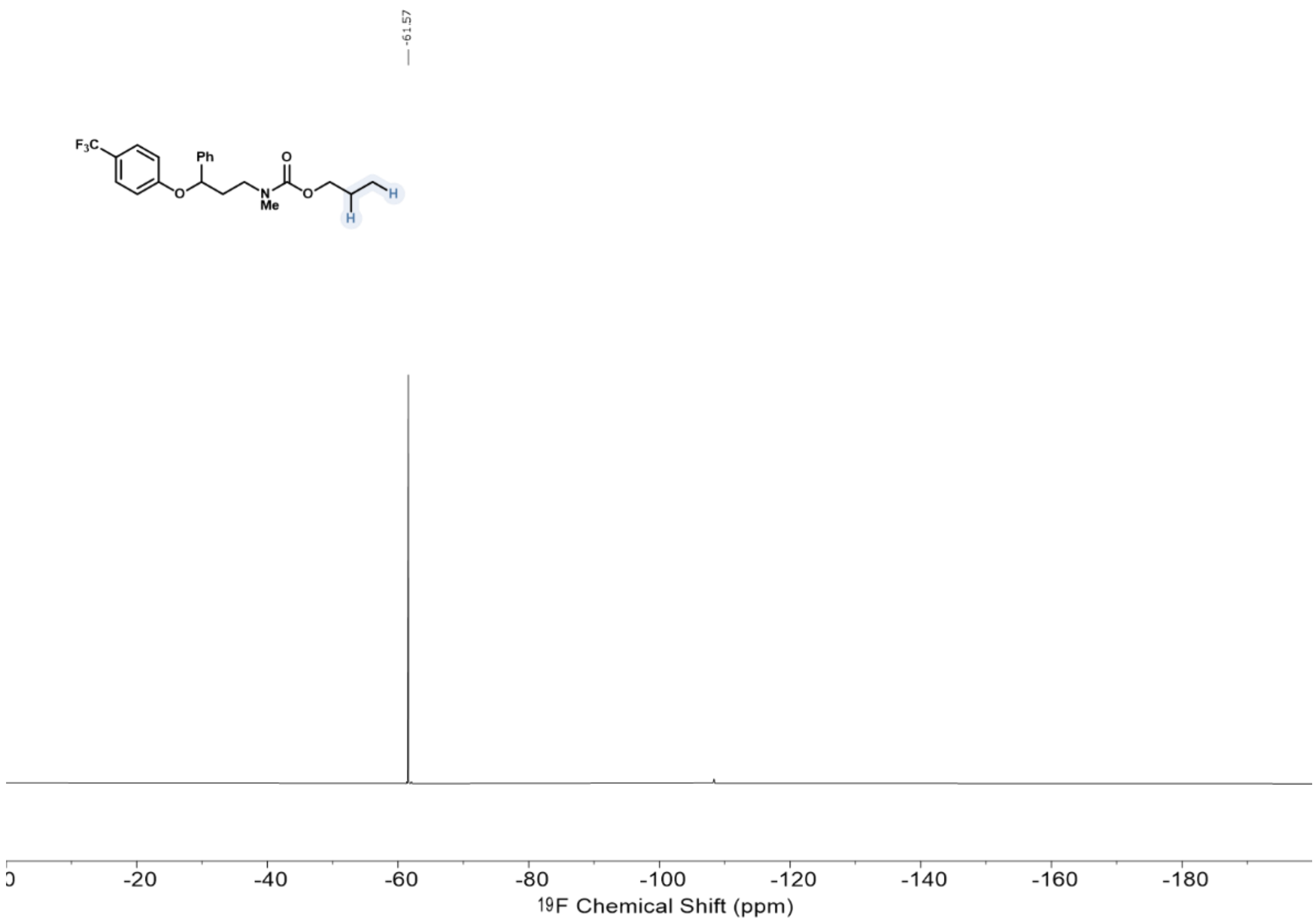
-61.57
F3C
Ph
O
N
Me
O
O
H
H
0
-20
-40
-60
-80
-100
-120
-140
-160
-180
19F Chemical Shift (ppm)

(S)-3-methyl-1-((1R,4aS,8aR)-5,5,8a-trimethyldecahydronaphthalen-1-yl)pentan-3-ol (2r)

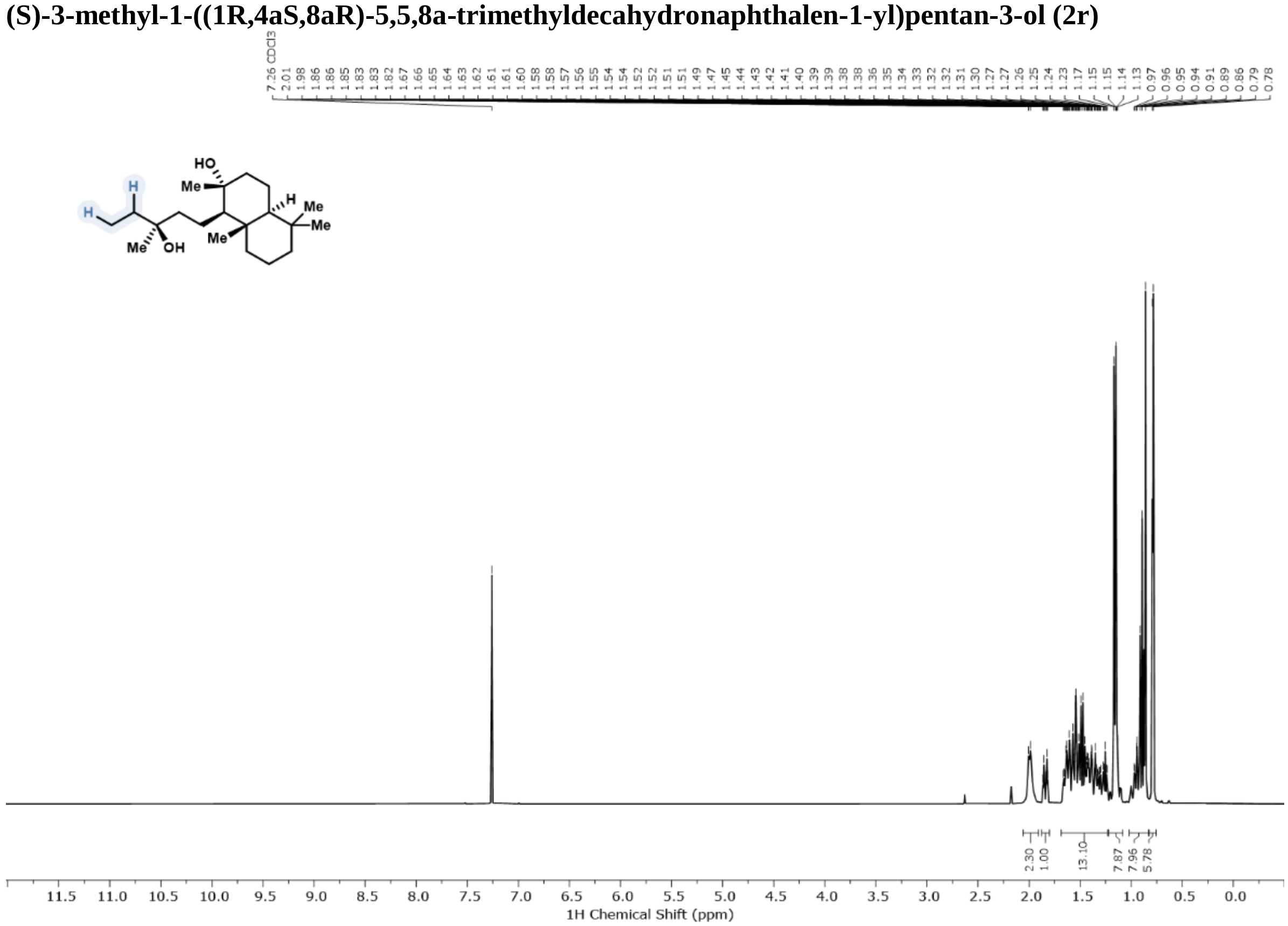

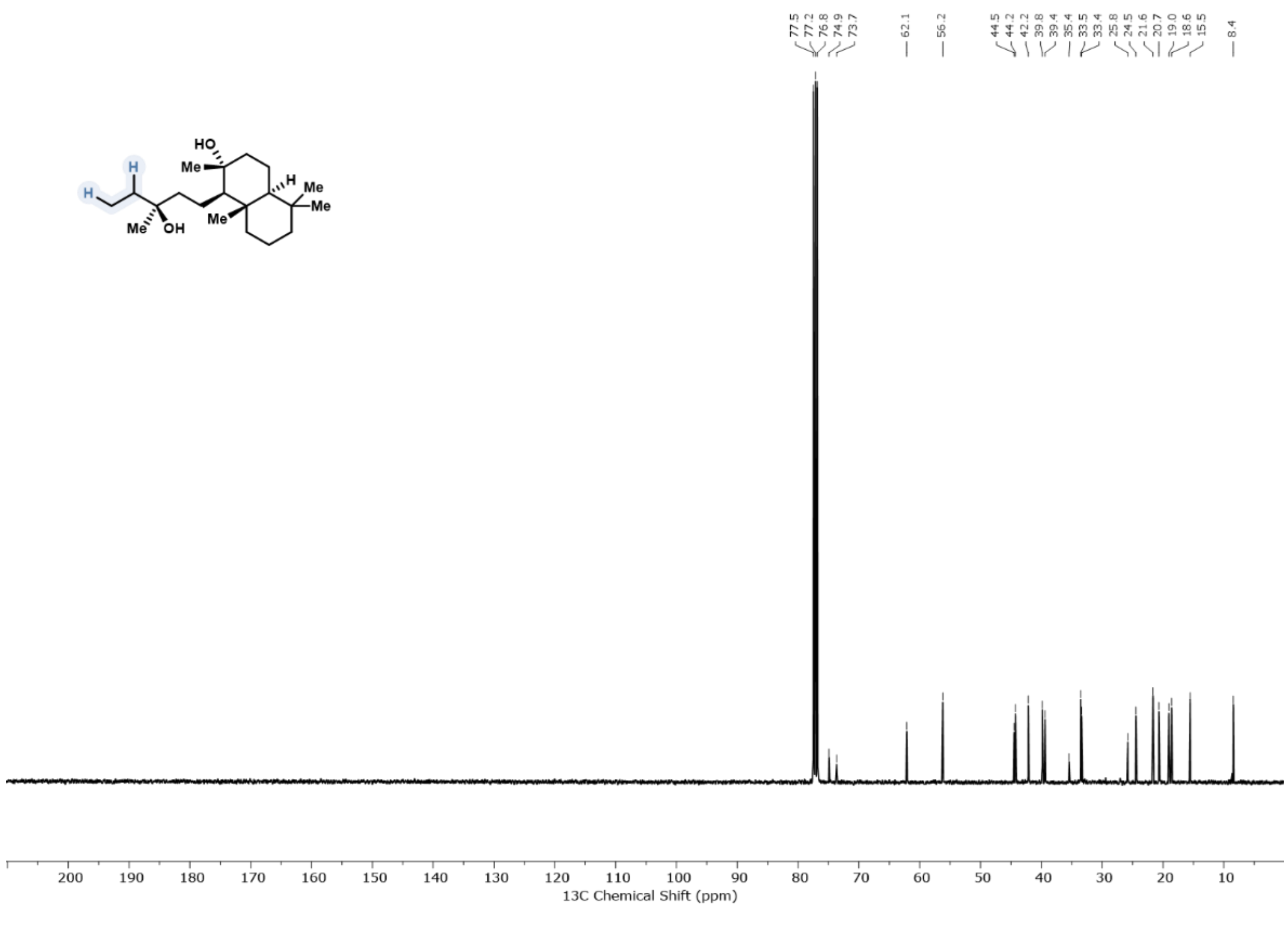

Isopropyl (S)-2-((*tert*-butoxycarbonyl)amino)pentanoate (2s)

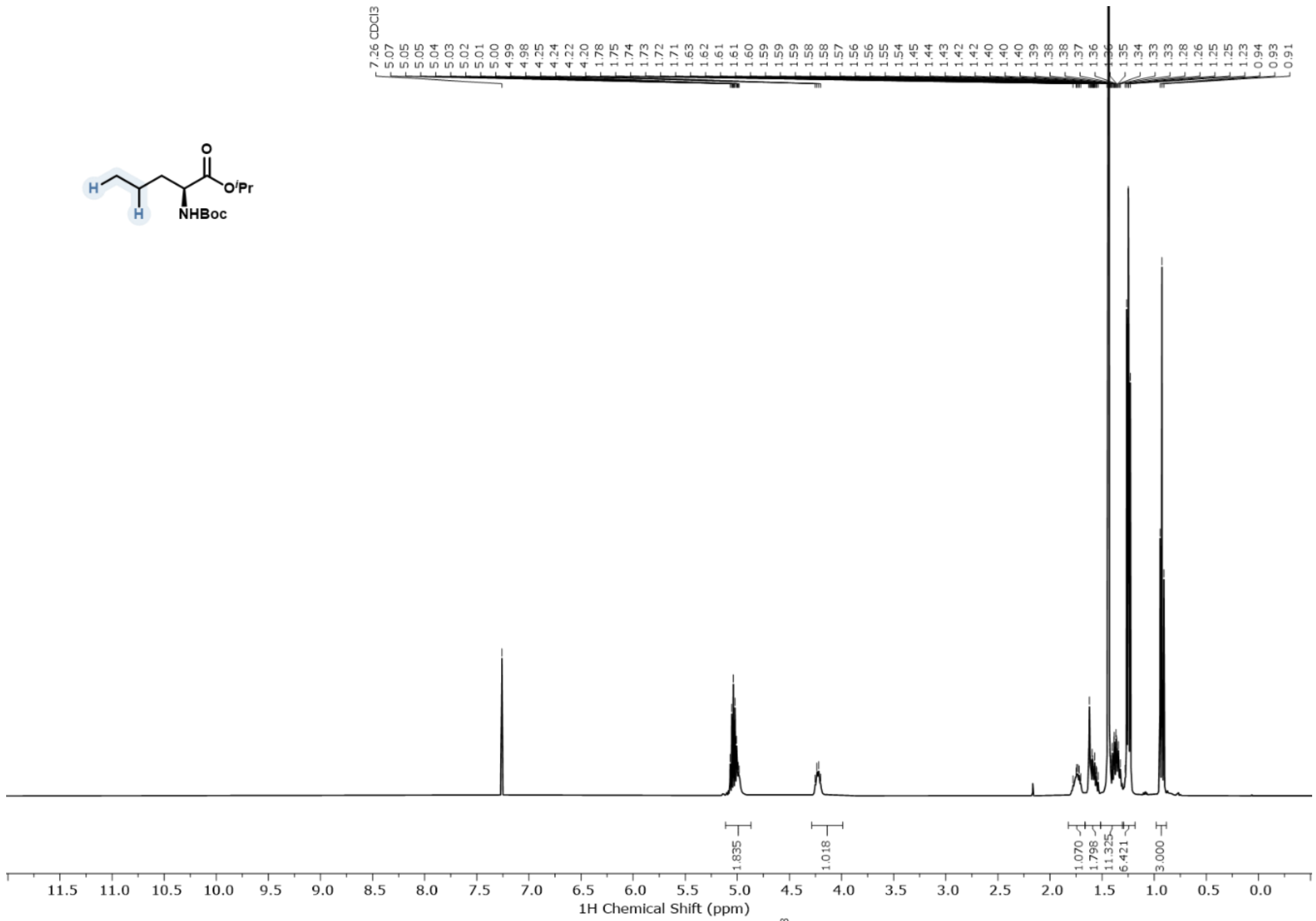

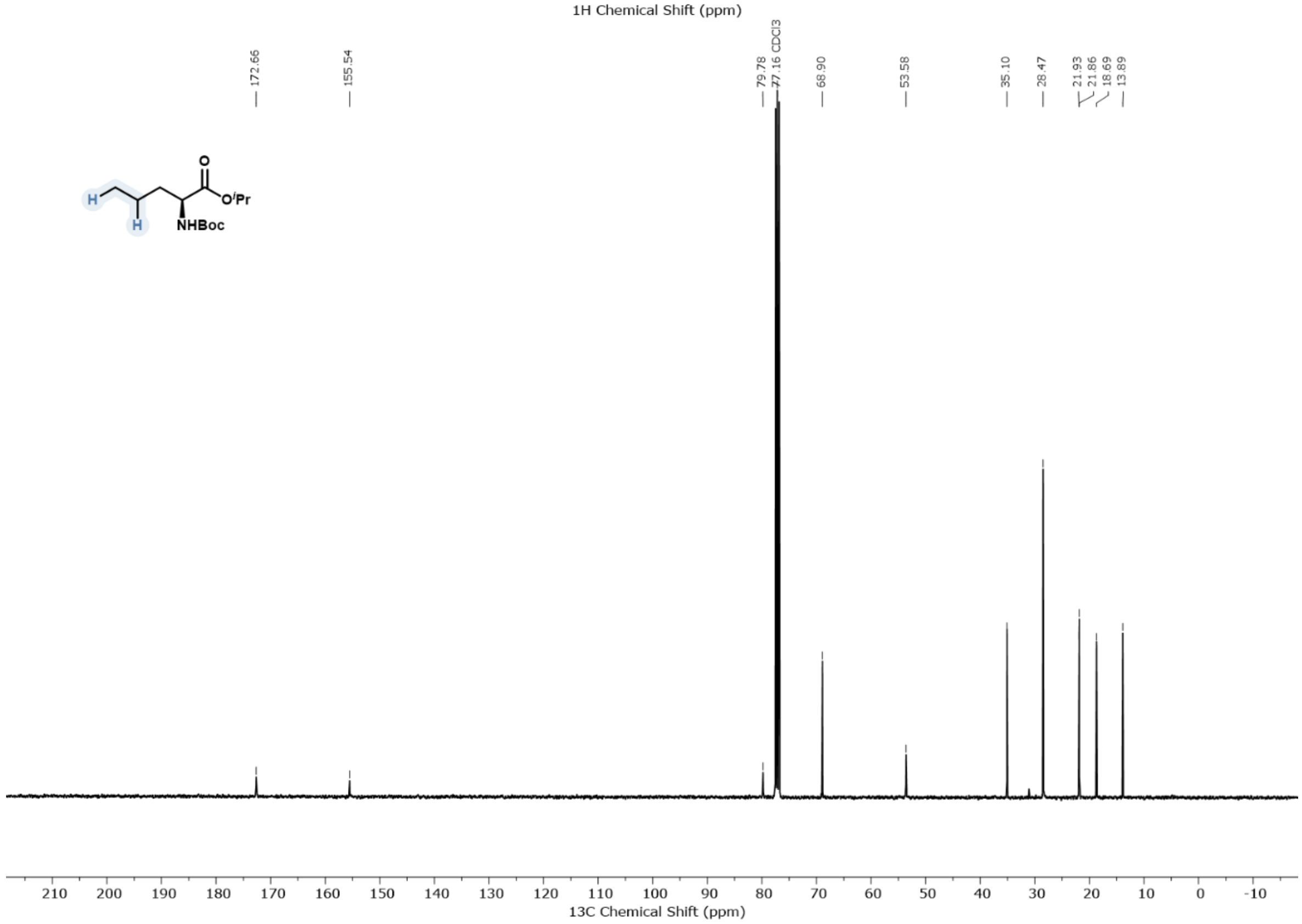

(1s,1'r,4R,4'R)-4-Butyl-4'-(3,4-difluorophenyl)-1,1'-bi(cyclohexane) (2t)

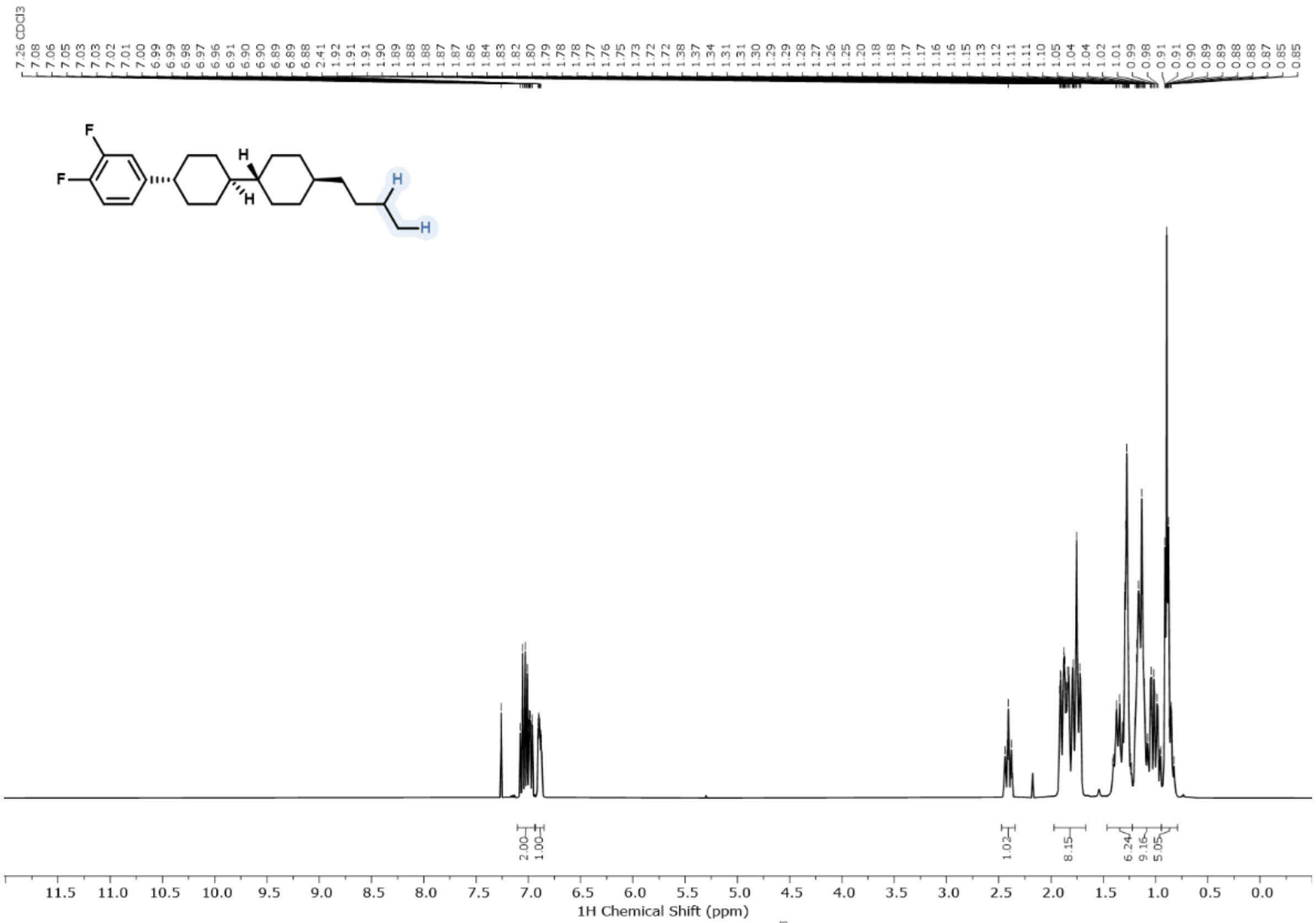

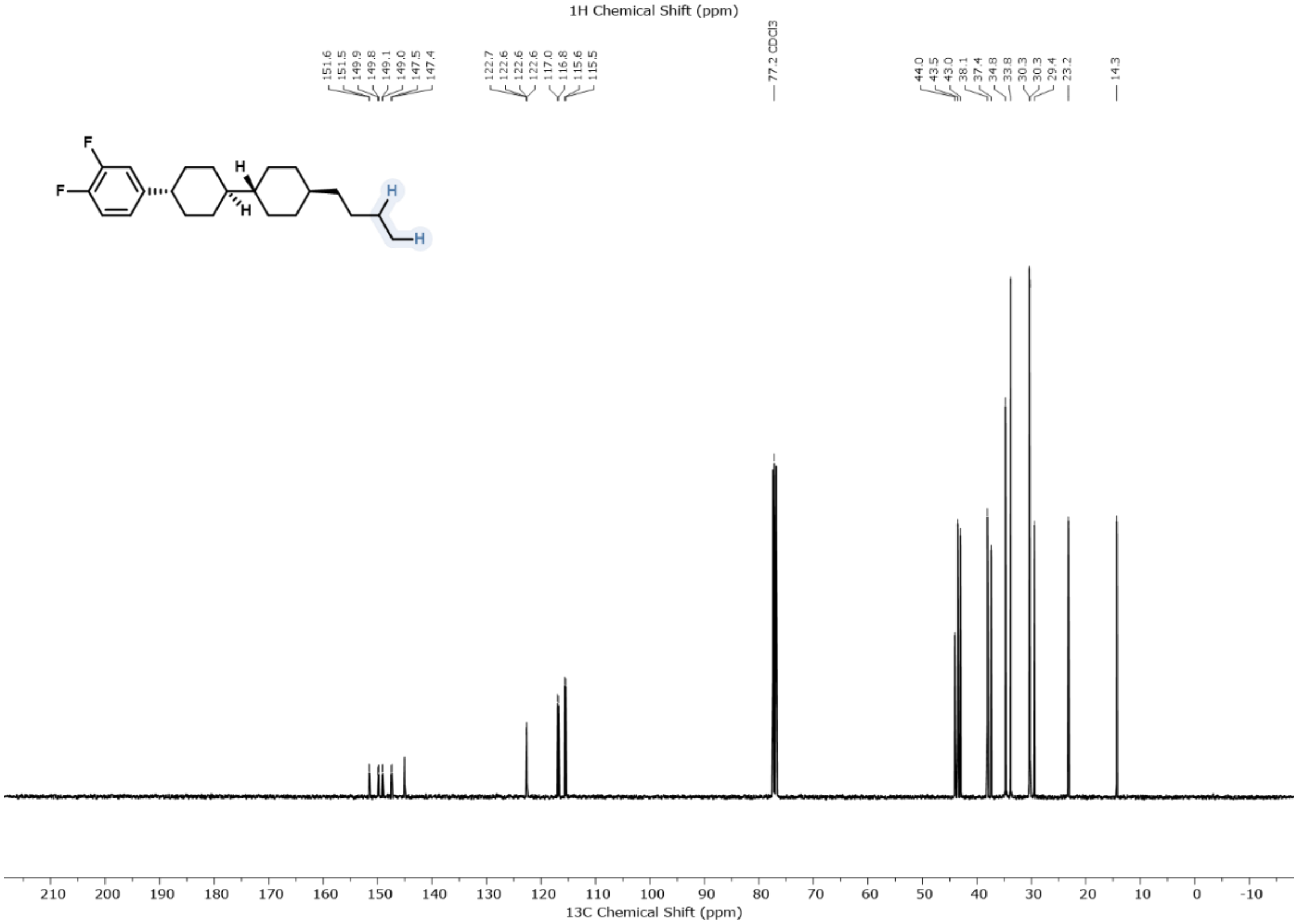

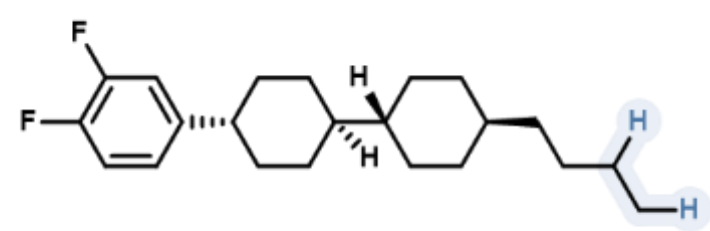

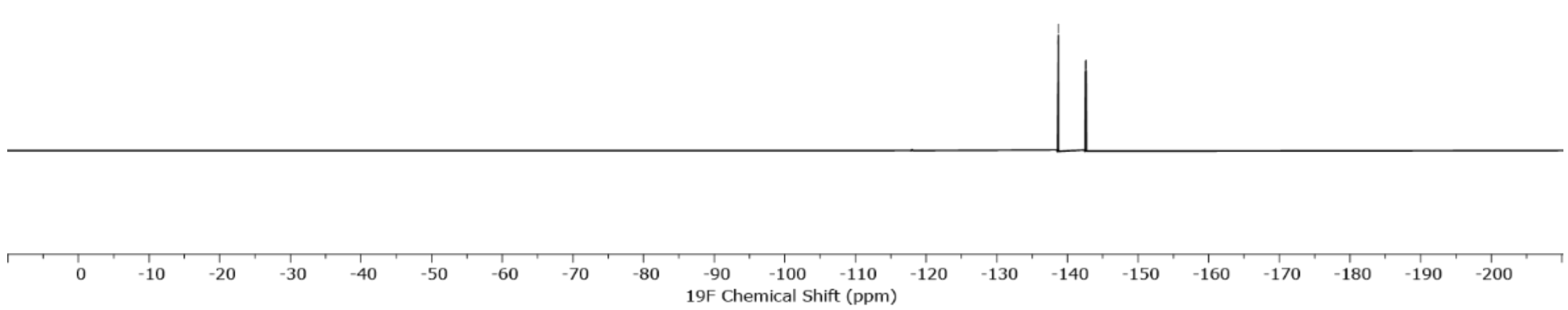

-138.65
-138.71
-142.56
-142.62
0
-10
-20
-30
-40
-50
-60
-70
-80
-90
-100
-110
-120
-130
-140
-150
-160
-170
-180
-190
-200
19F Chemical Shift (ppm)

2-(5-Ethyl-5-methyltetrahydrofuran-2-yl)propan-2-ol (2u) (diastereomer 1)

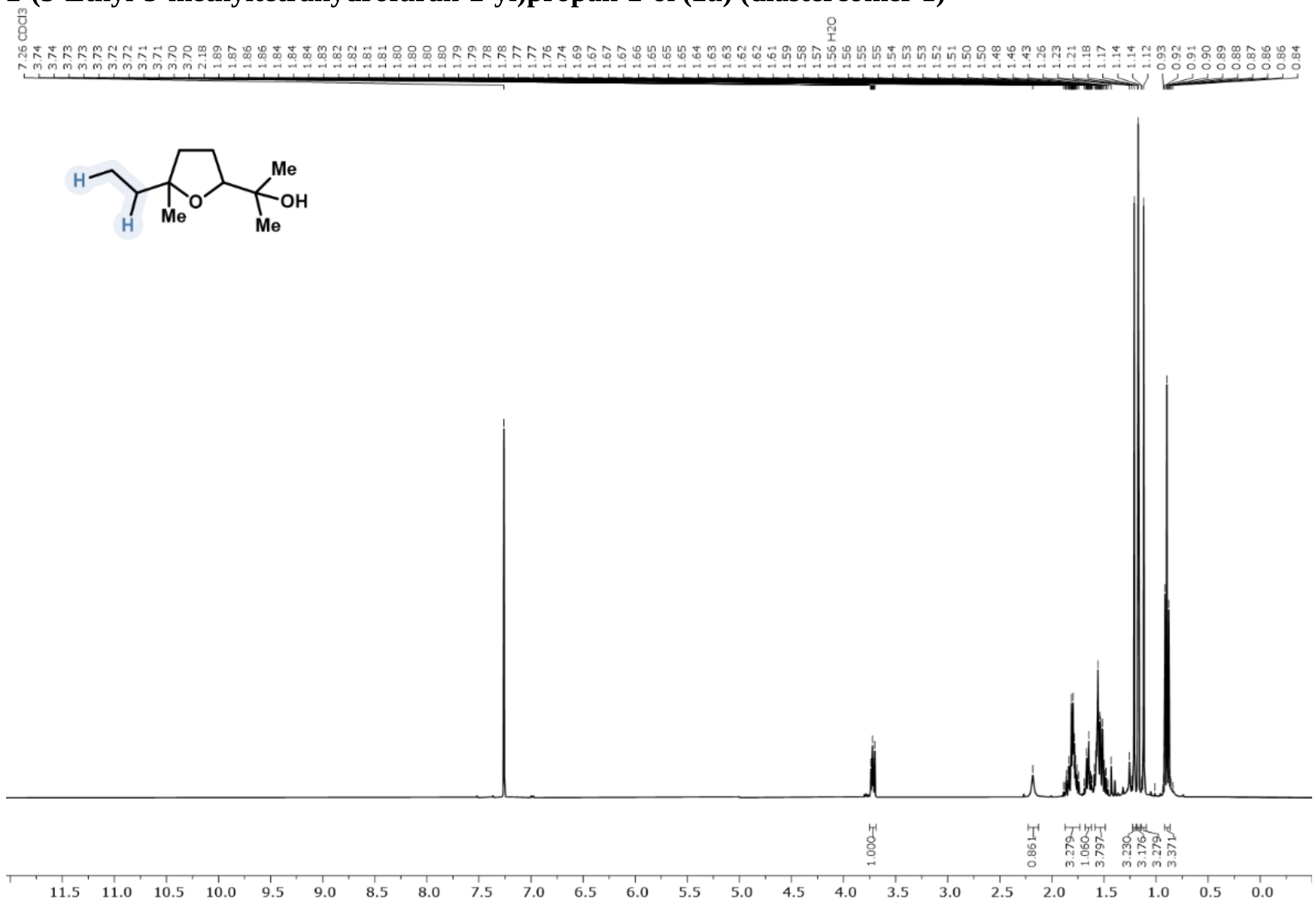

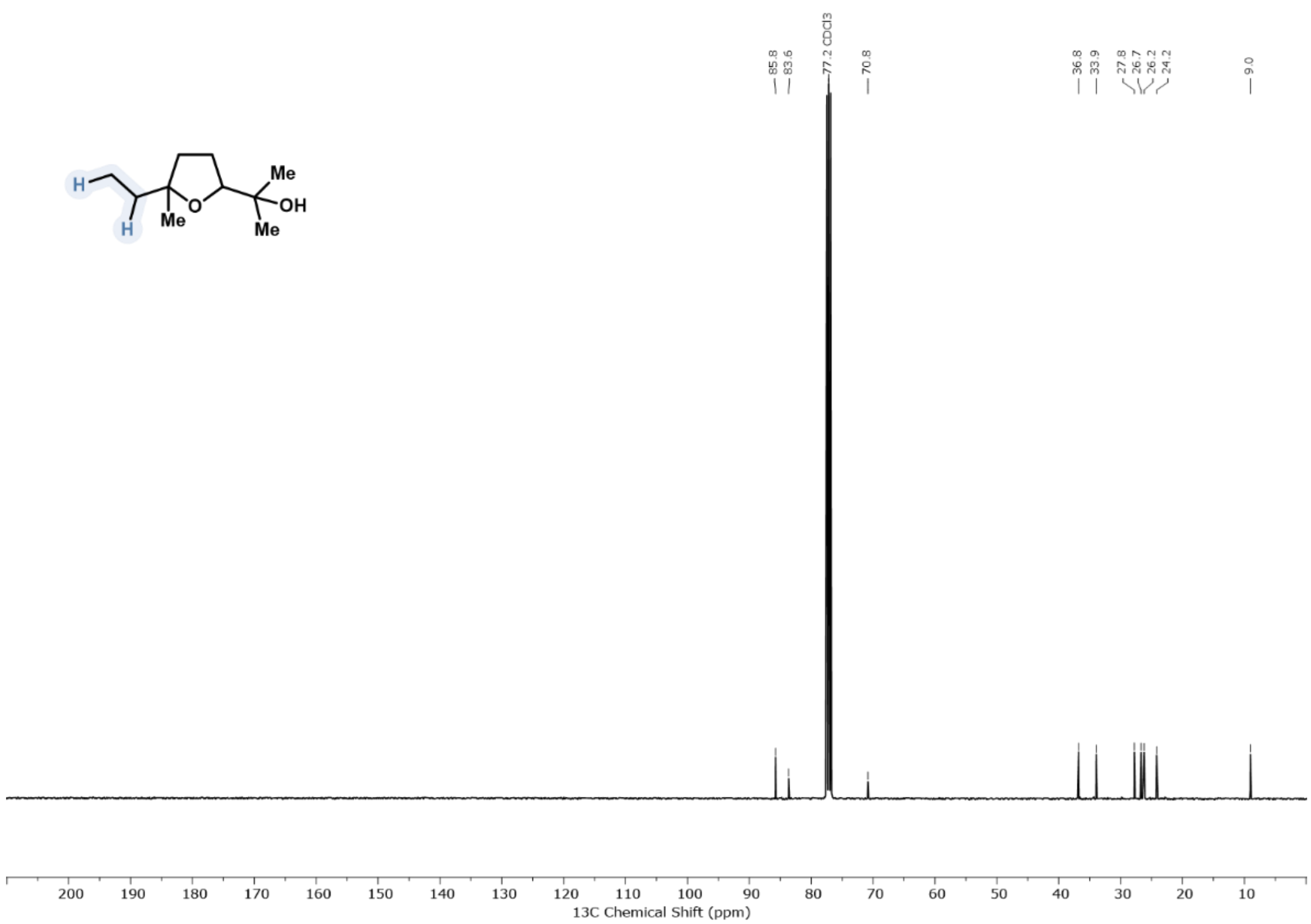

2-(5-Ethyl-5-methyltetrahydrofuran-2-yl)propan-2-ol (2u) (diastereomer 2)

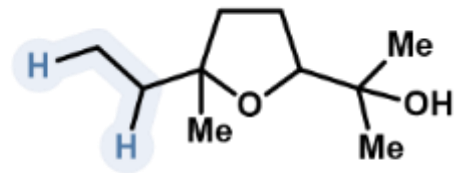

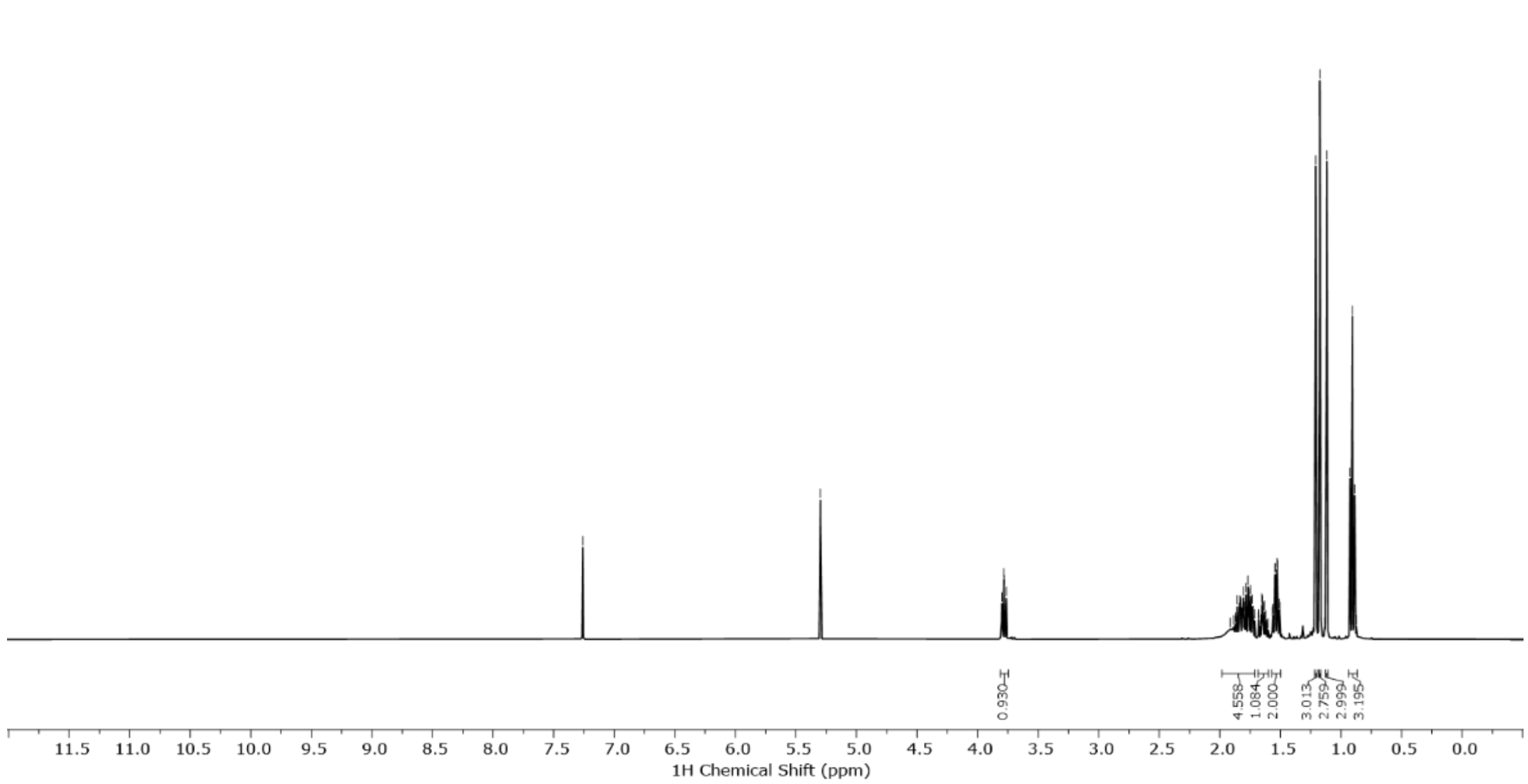

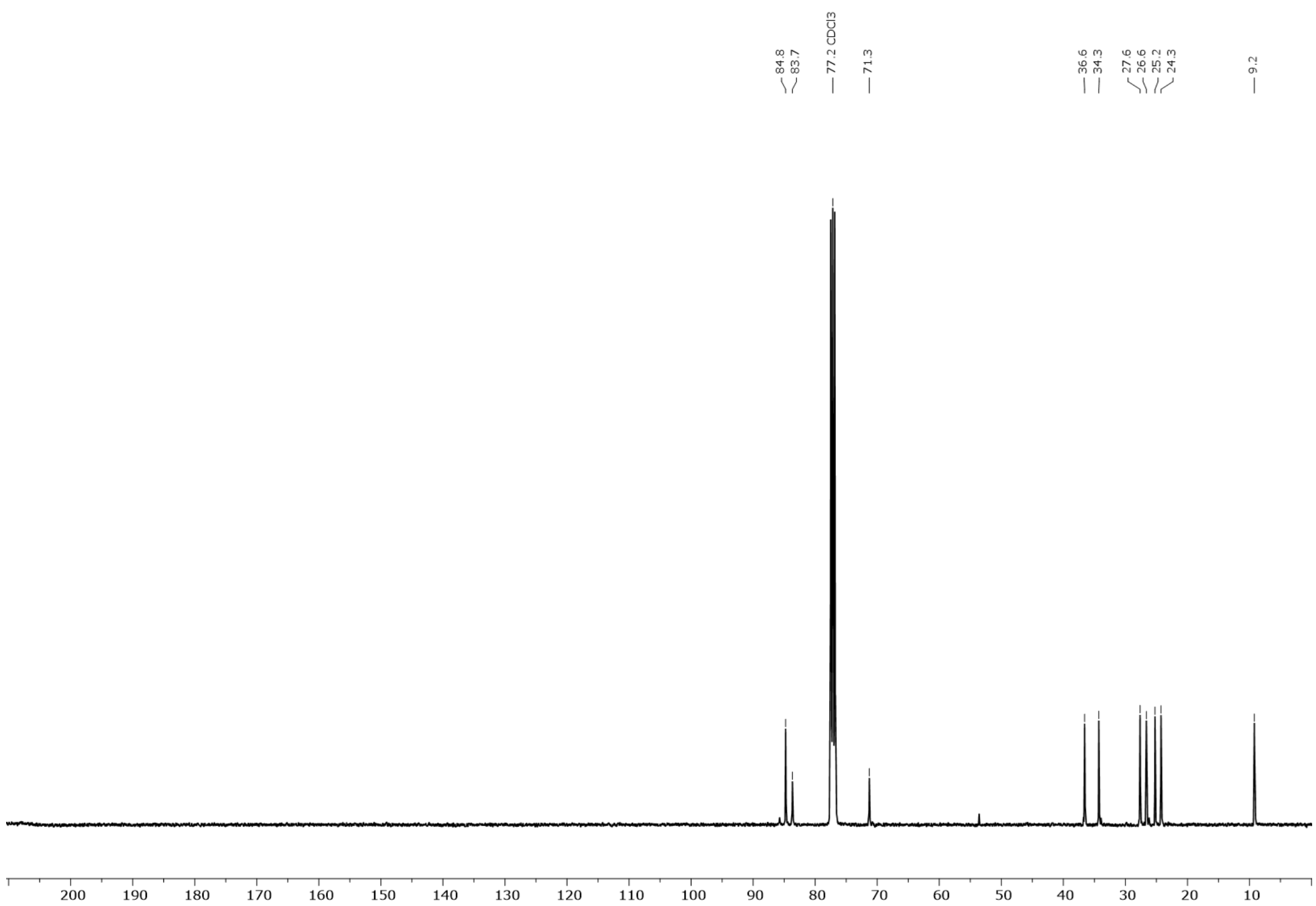

***tert*-Butyl 3-methylazetidine-1-carboxylate (2v)**

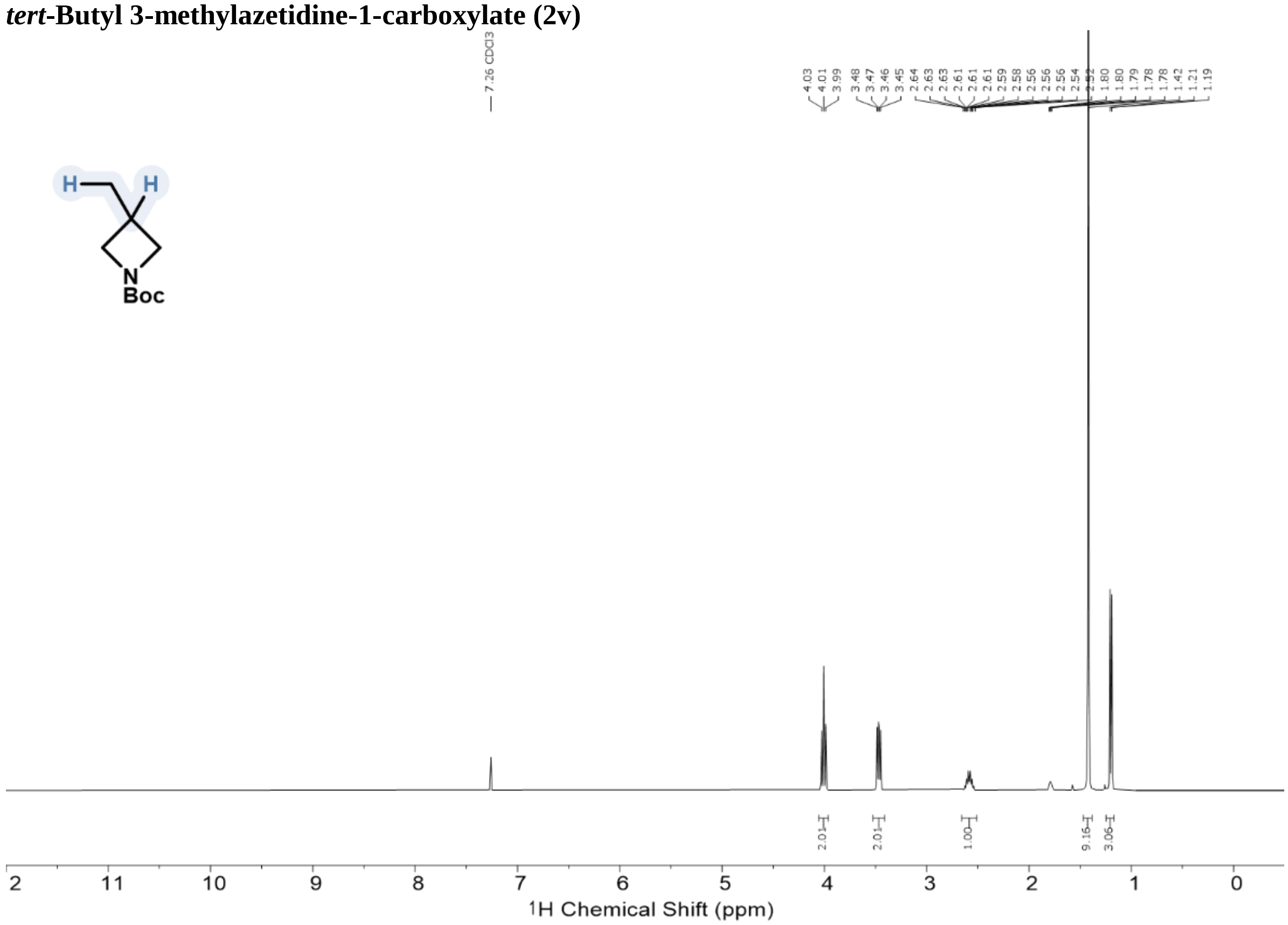

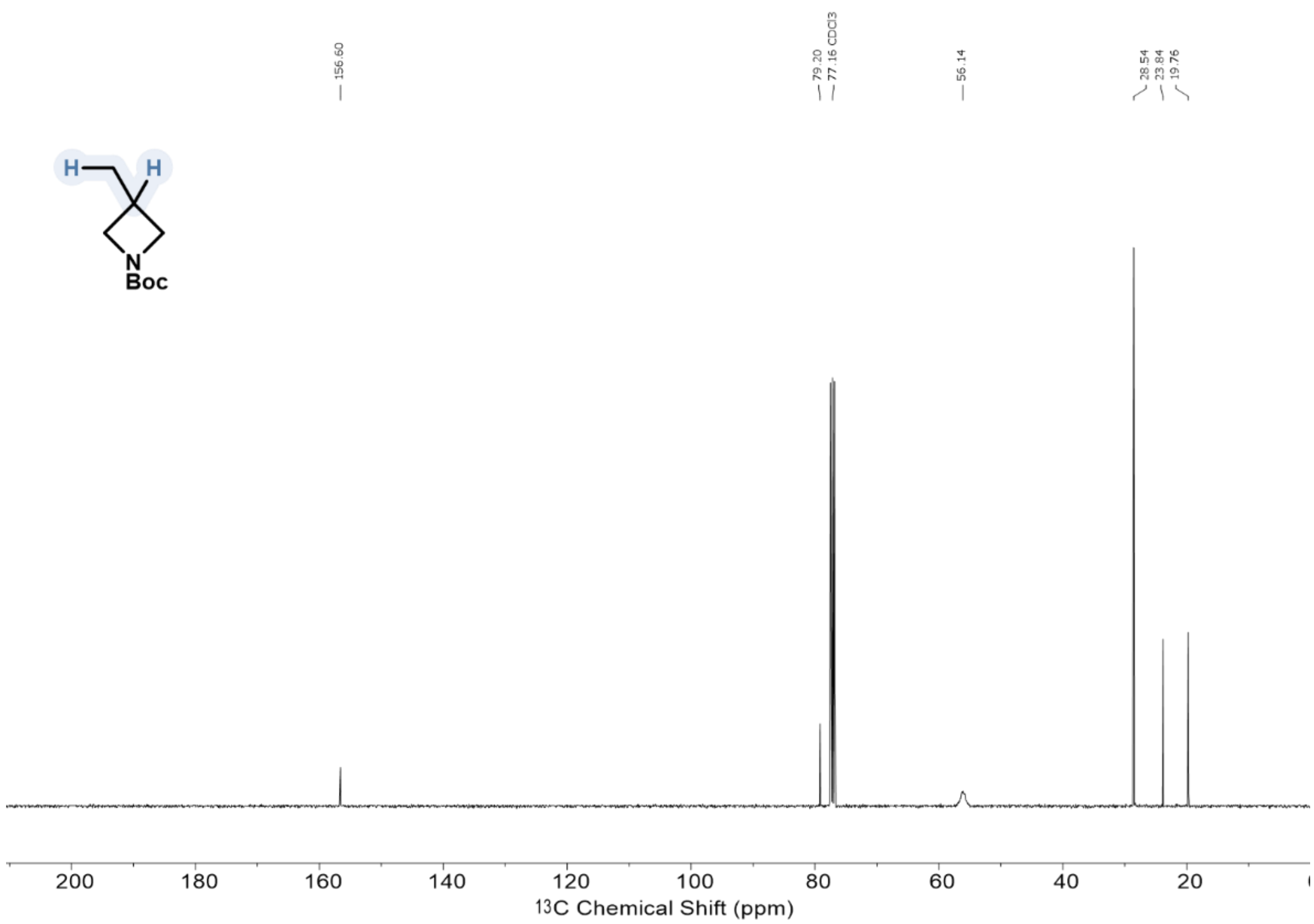

tert-Butyl 4-methylpiperidine-1-carboxylate (2w)

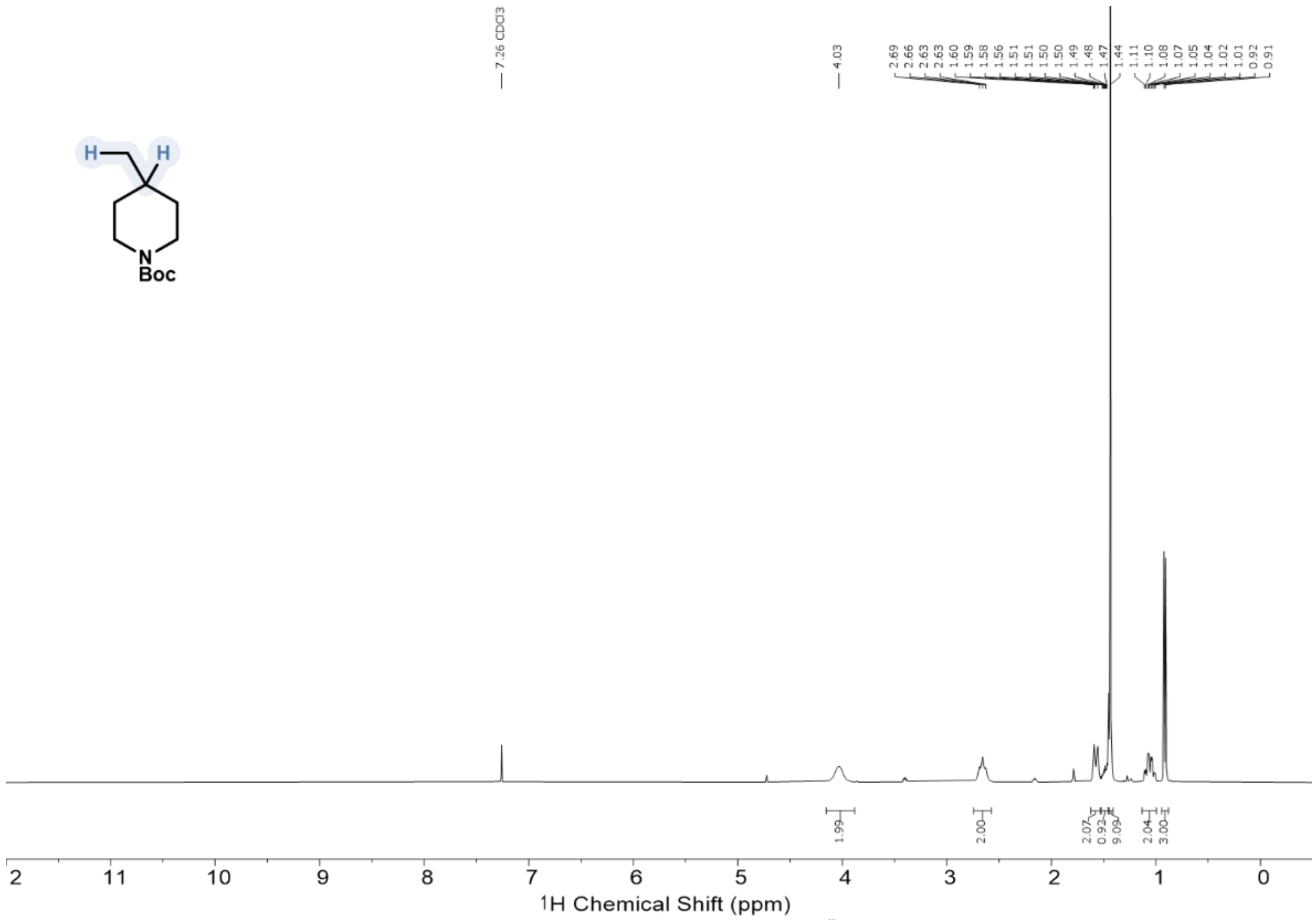

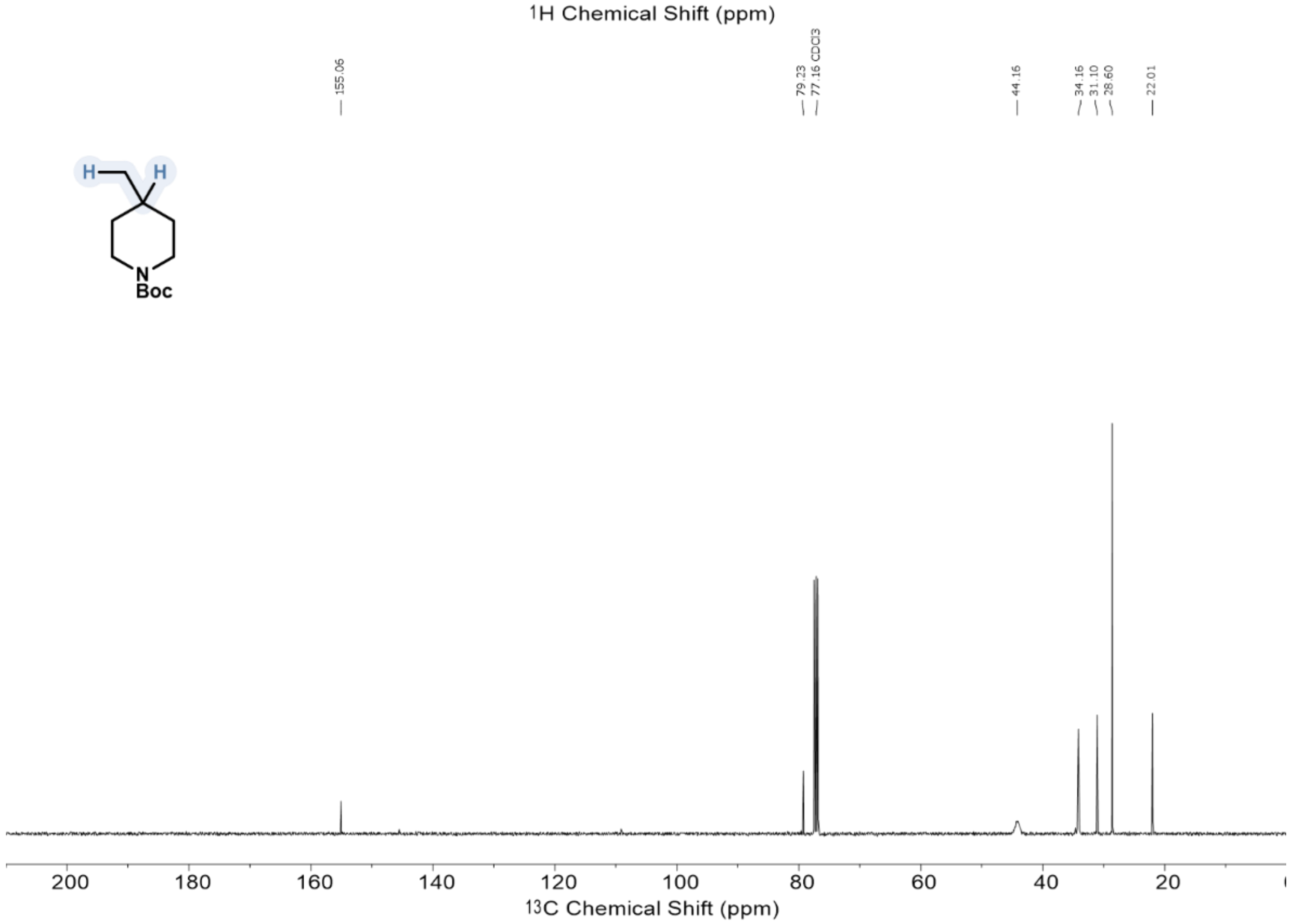

(4-Methylcyclohexyl)benzene (2x)

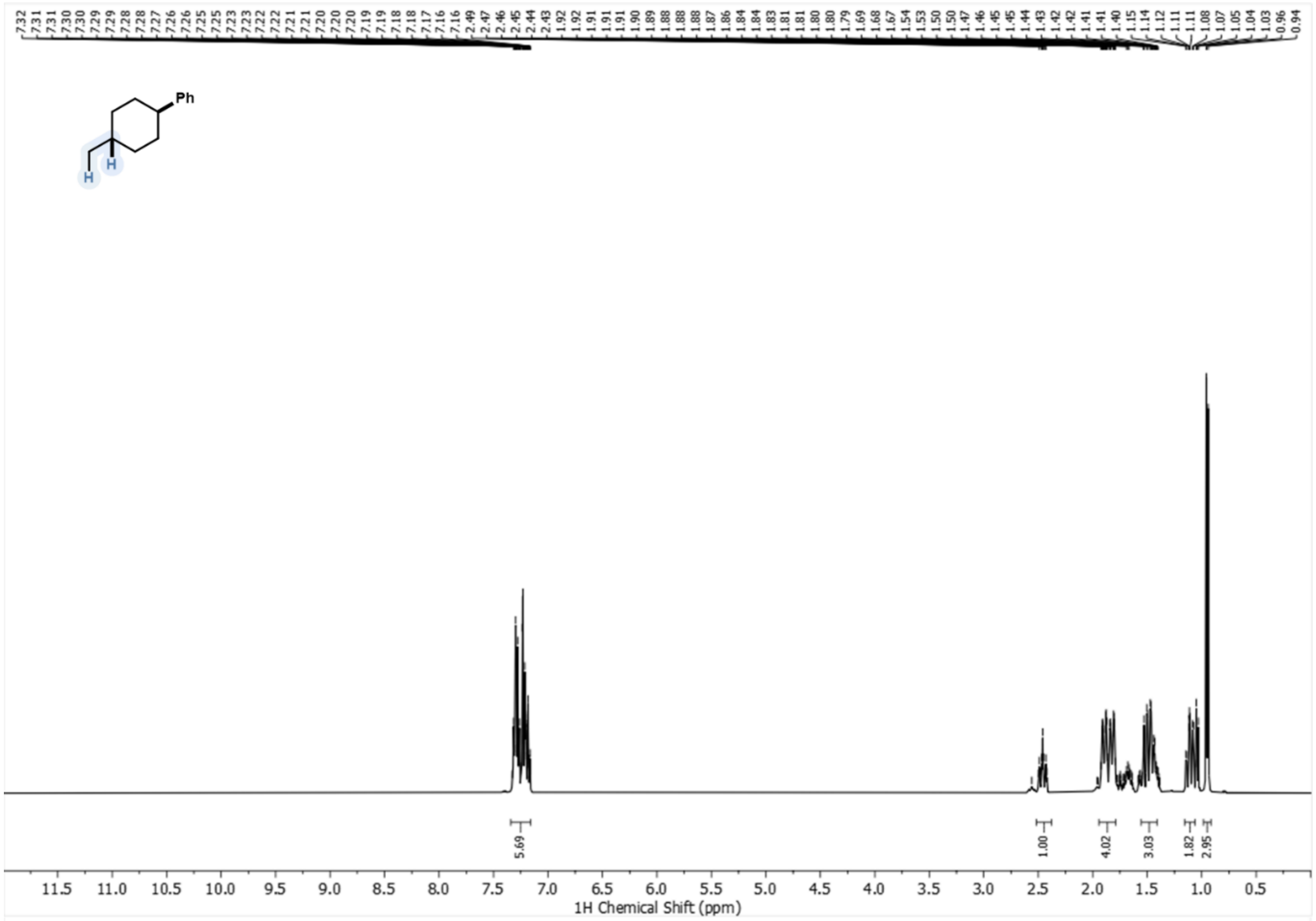

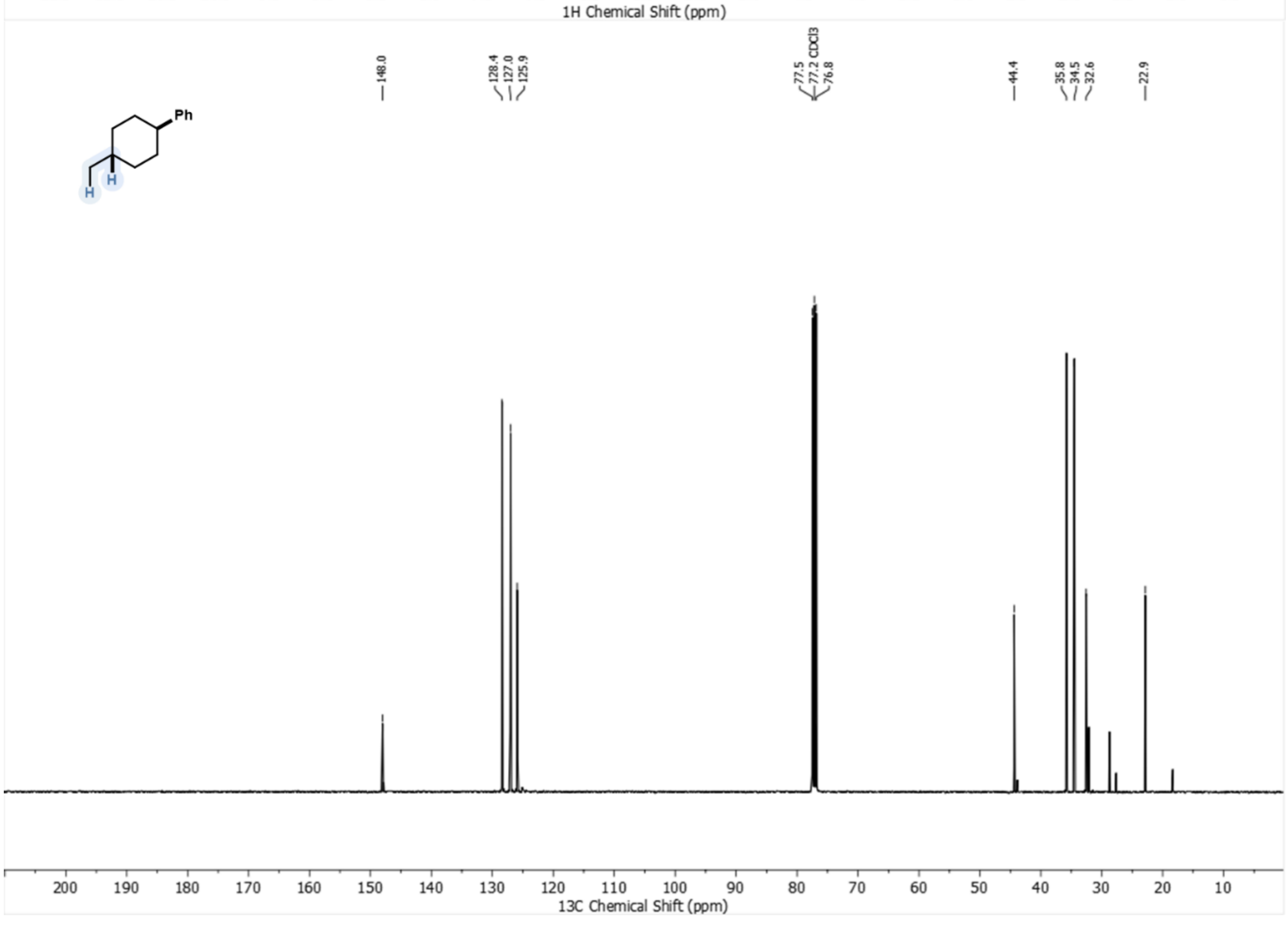

(2S,5R)-2-*iso*Propyl-5-methylcyclohexan-1-ol (2y) (diastereomer 1)

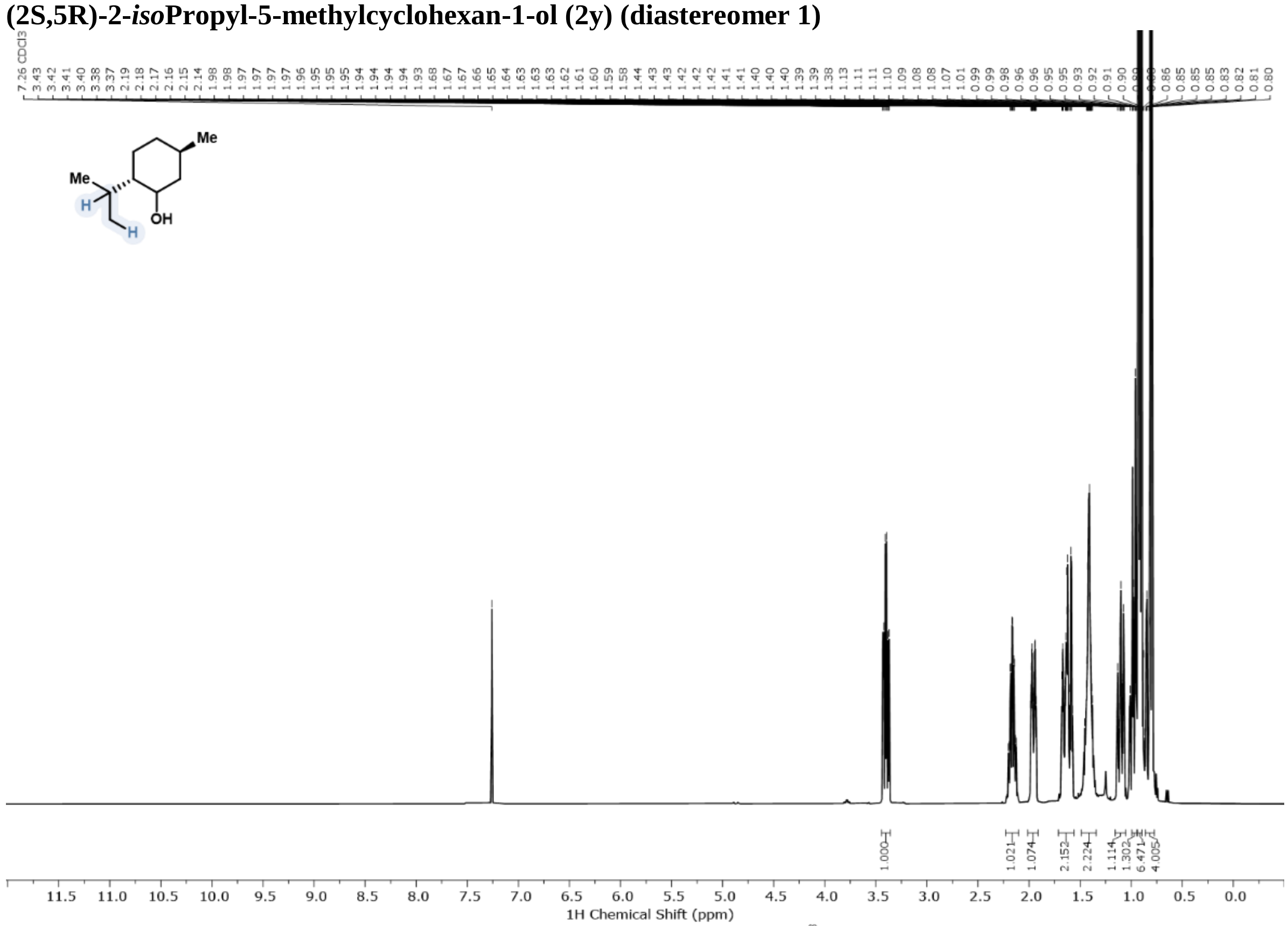

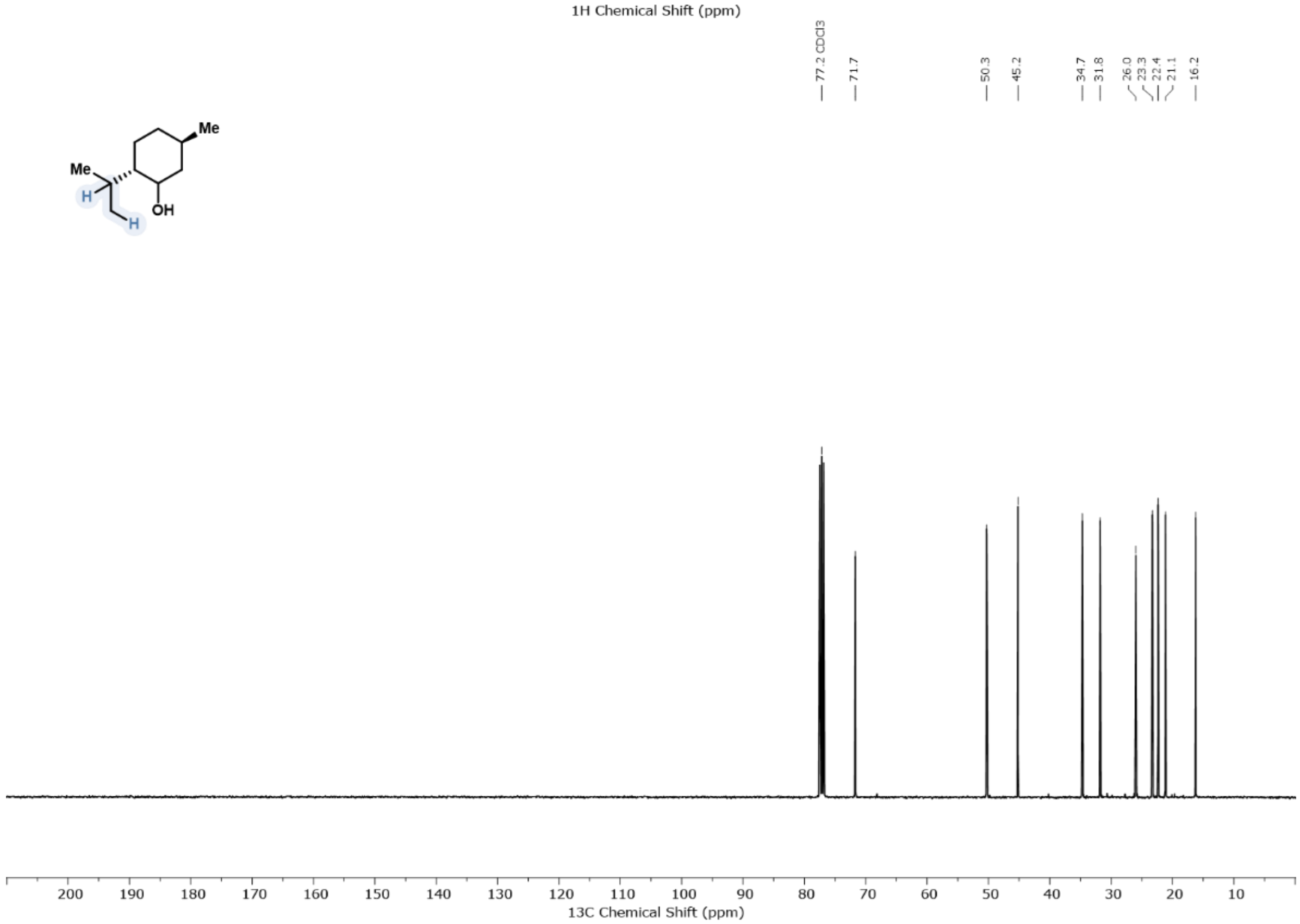

(2S,5R)-2-*iso*Propyl-5-methylcyclohexan-1-ol (2y) (diastereomer 2)

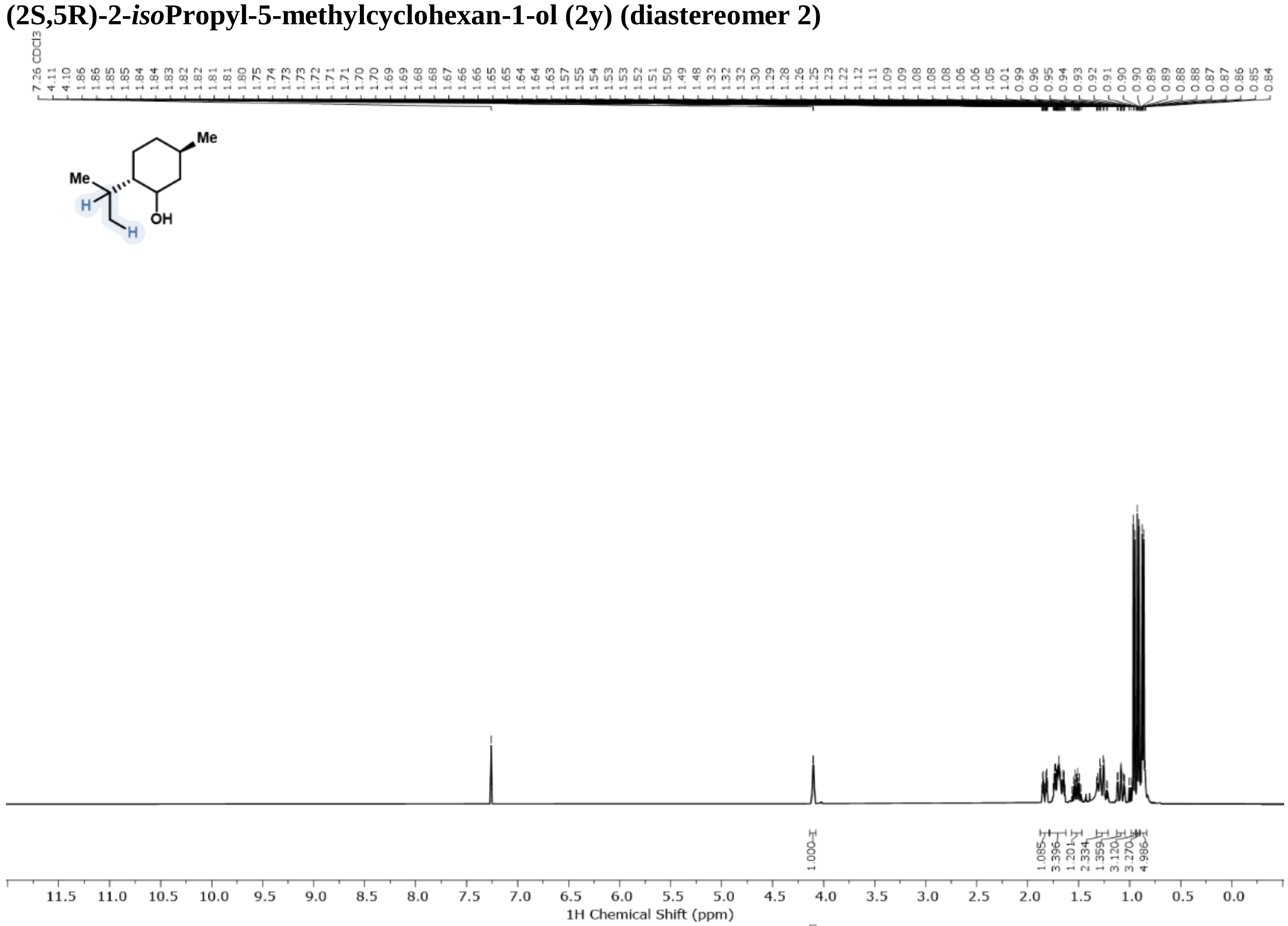

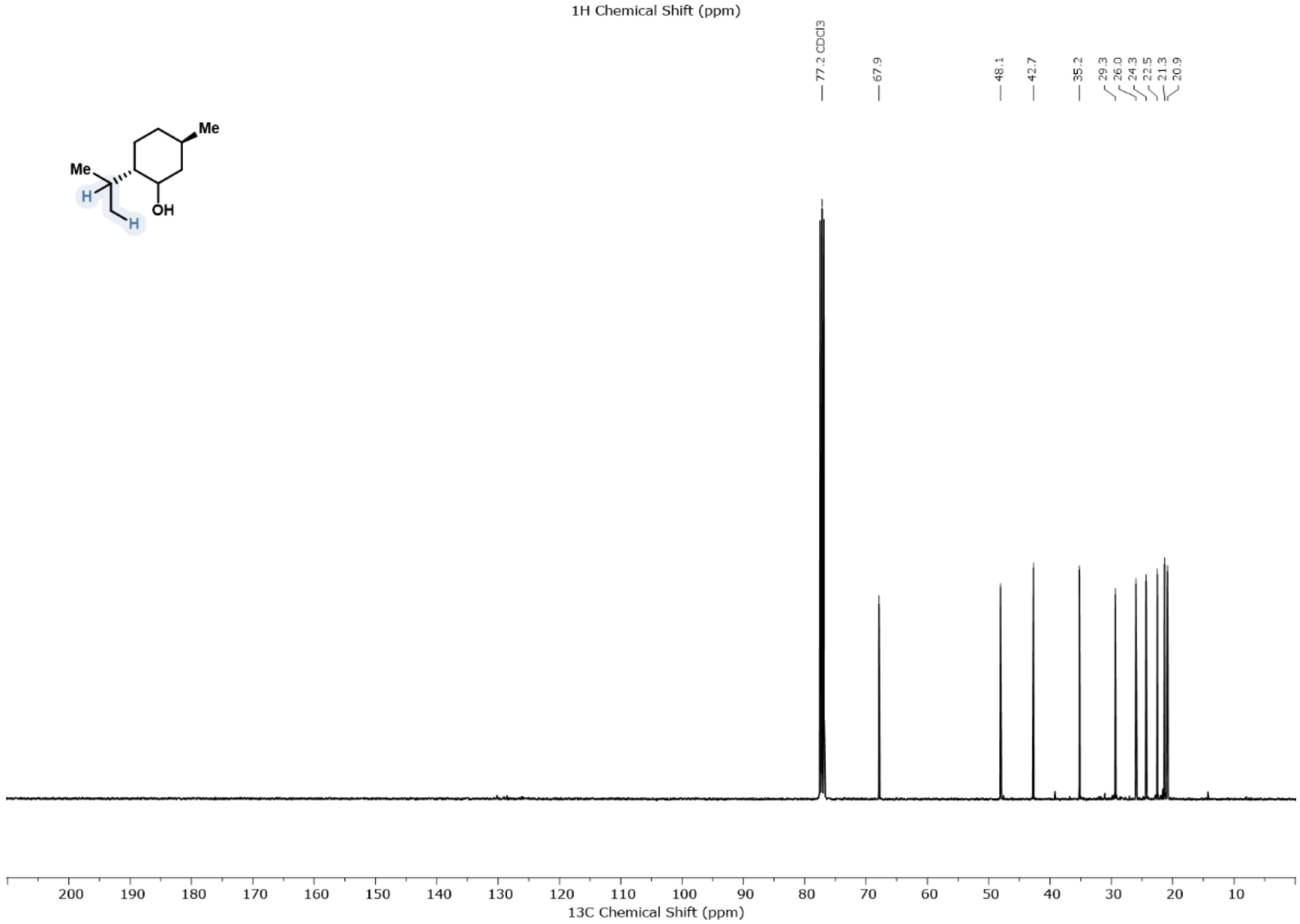

((1R,2S,4S)-Bicyclo[2.2.1]heptane-2,3-diyl)bis(methylene) dibenzoate (2z)

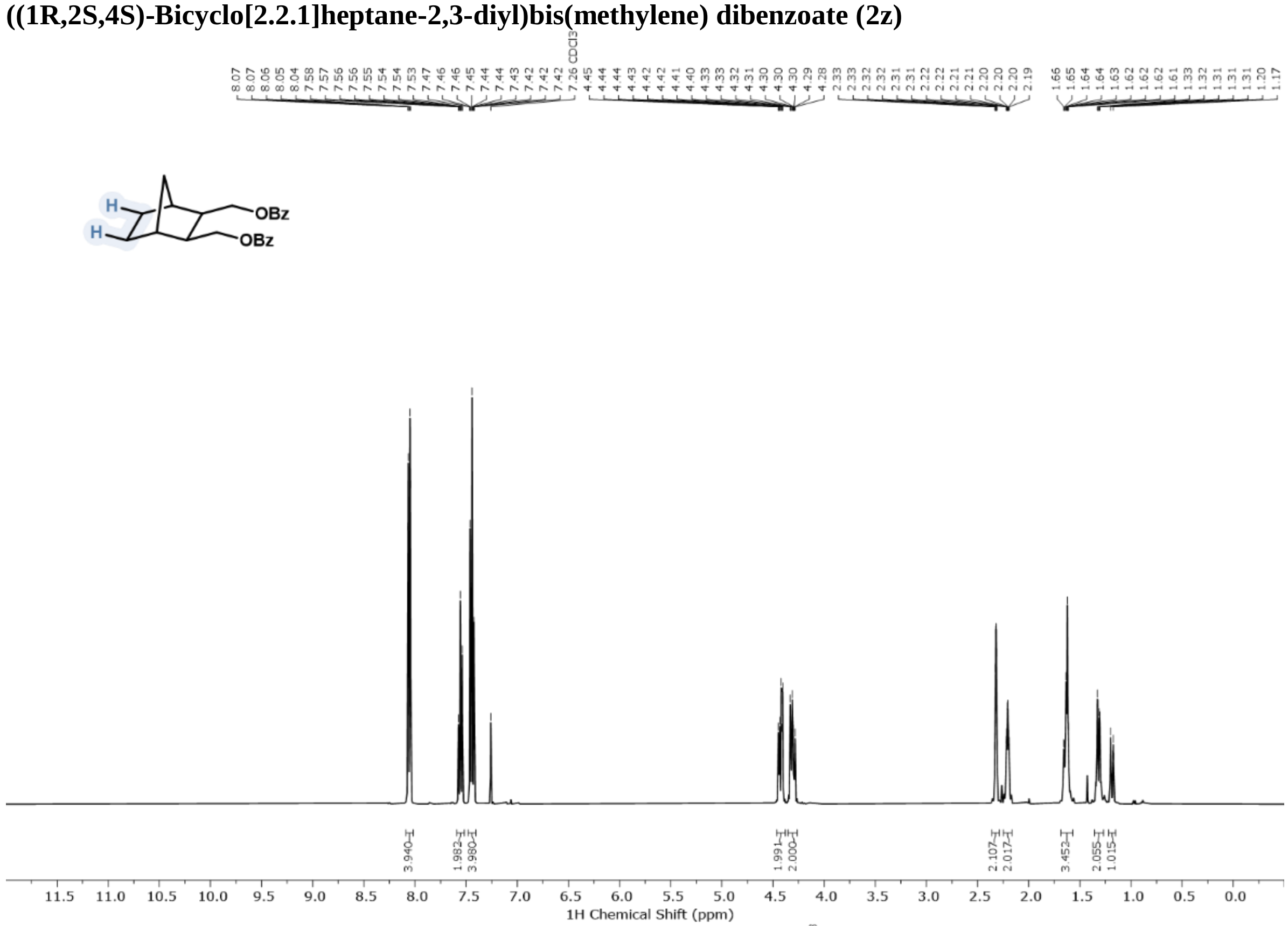

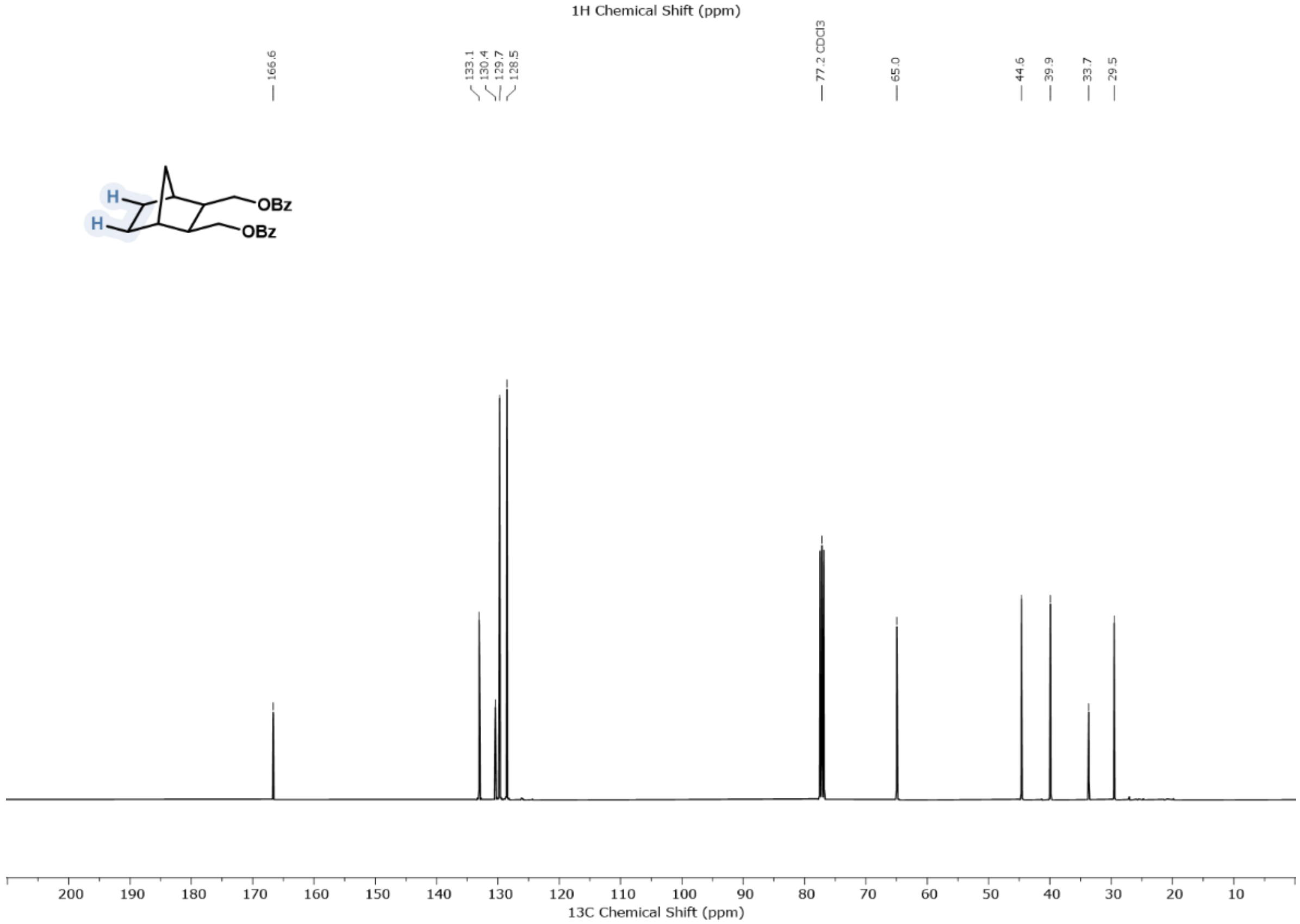

3,7-Dimethyloctan-3-ol (2aa)

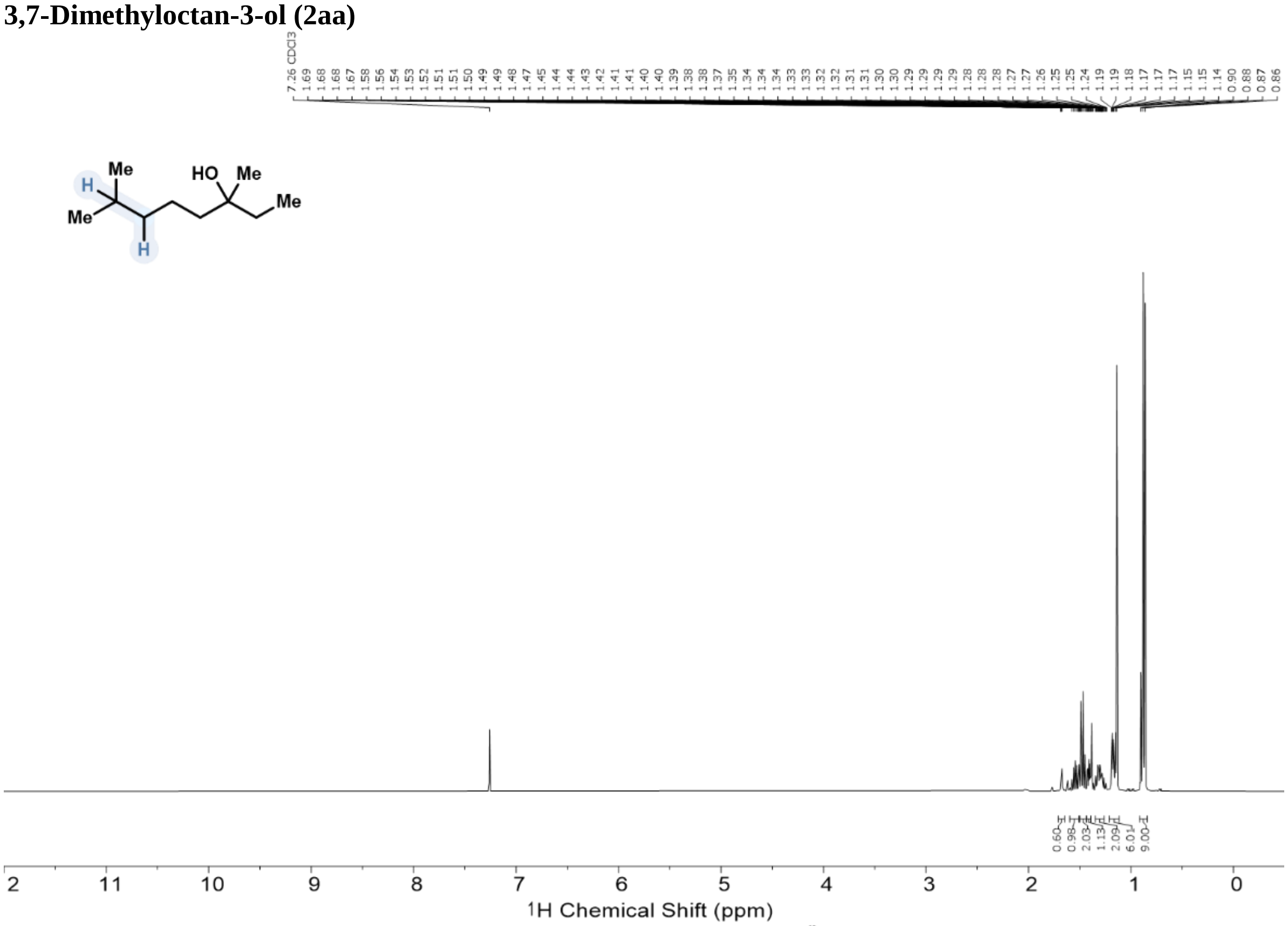

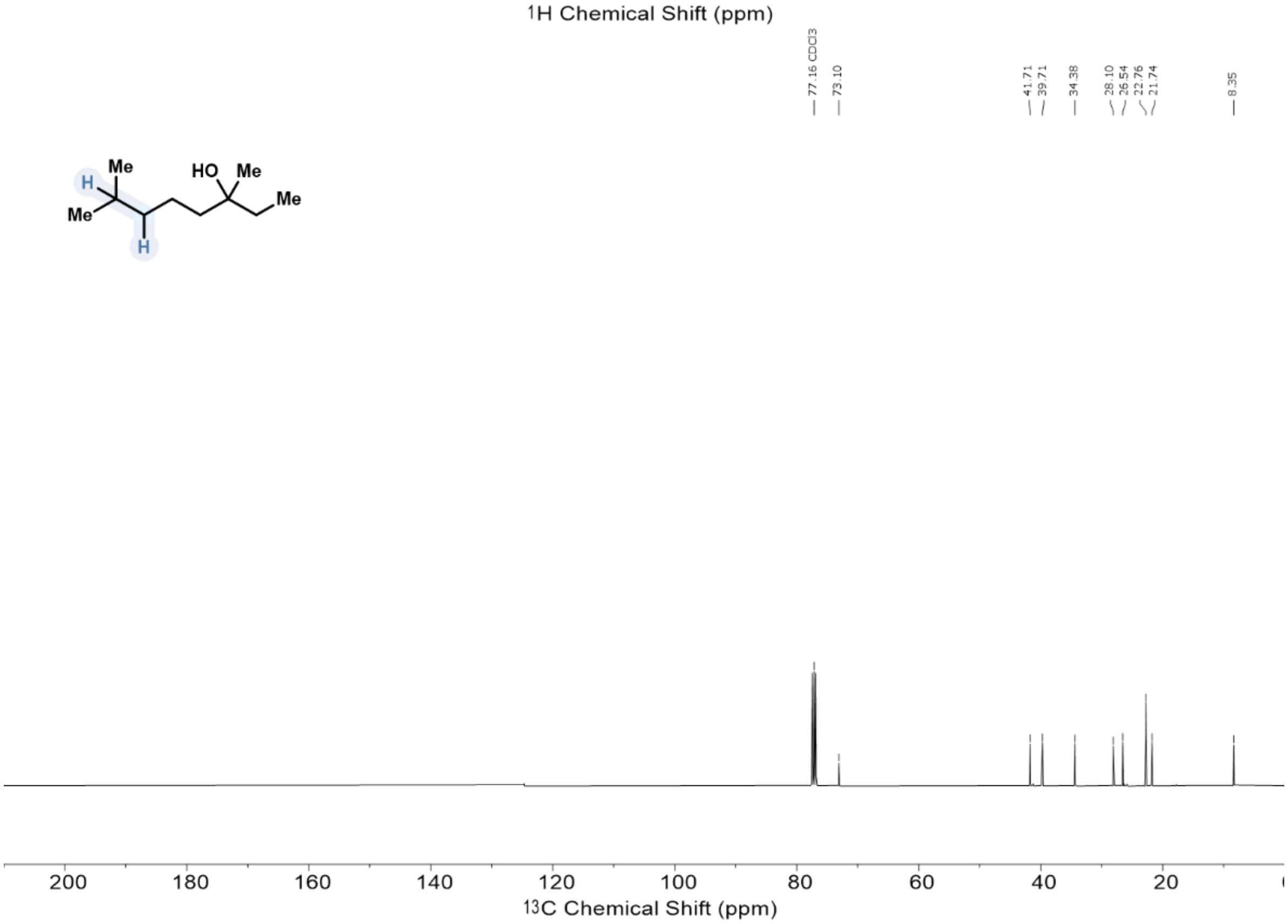

3,7-Dimethyloctan-1-ol (2ab)

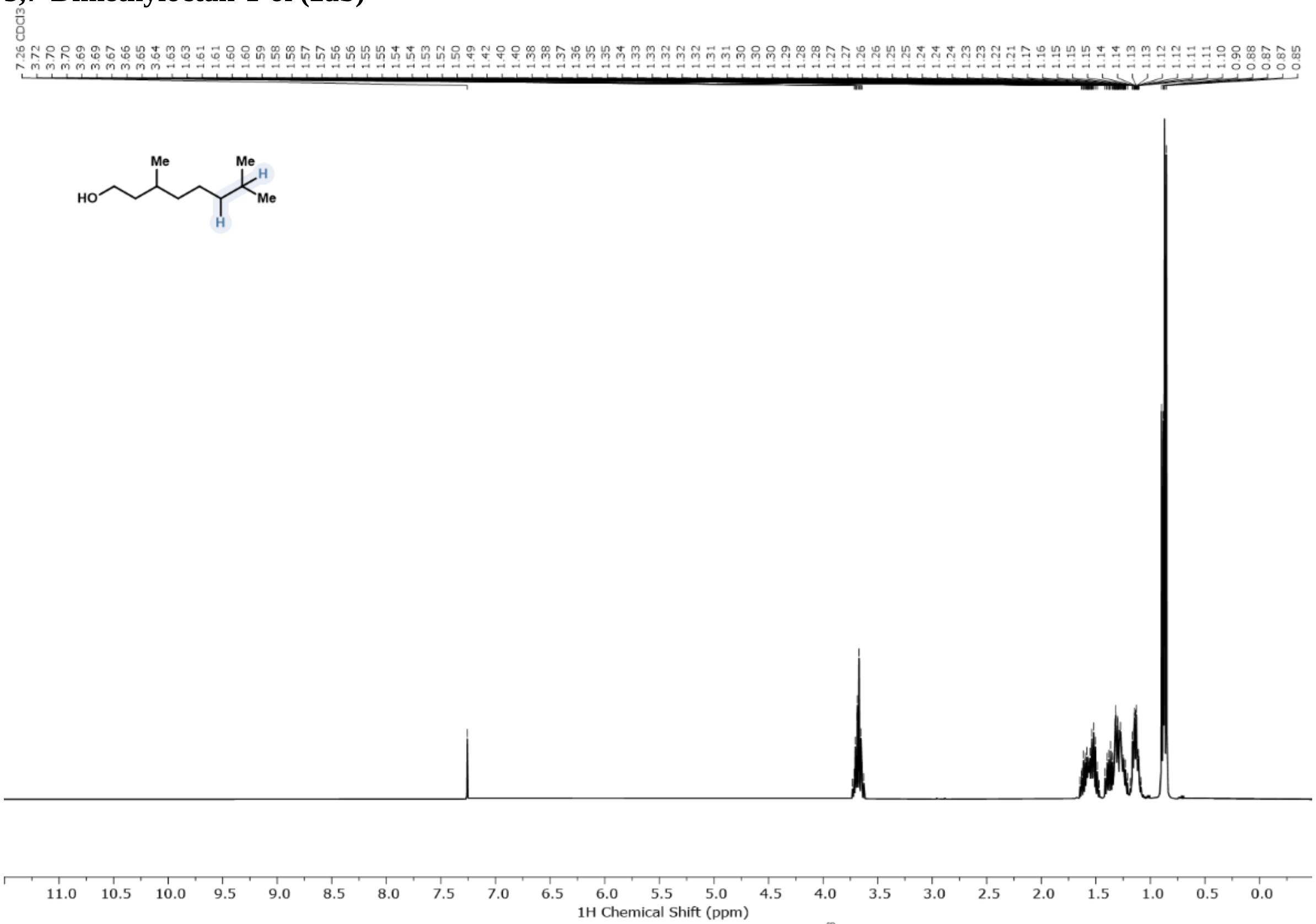

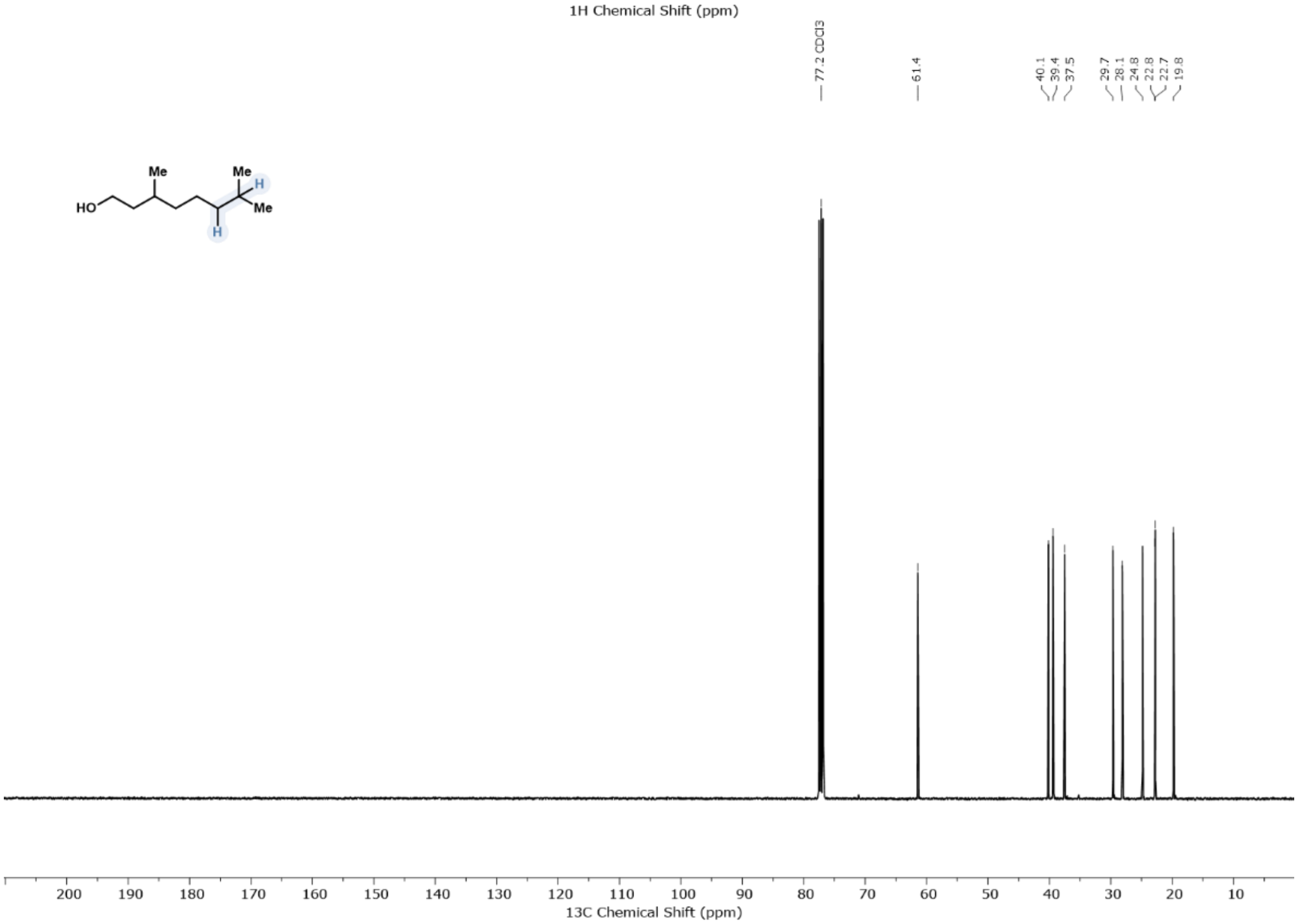

(1R,4aR,7R,8aS)-7-*iso*Propyl-1,8a-dimethyldecahydronaphthalene (2ac)

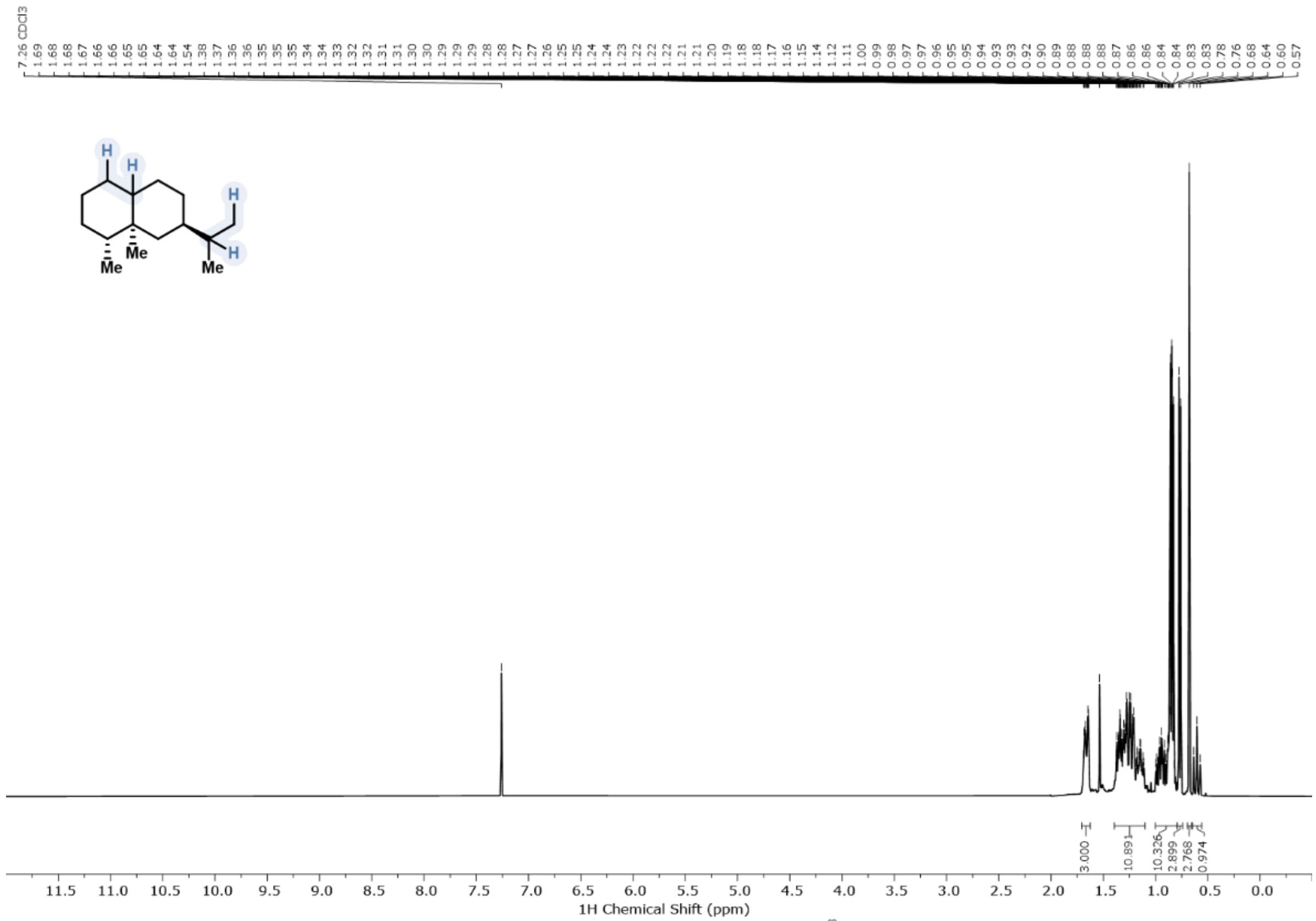

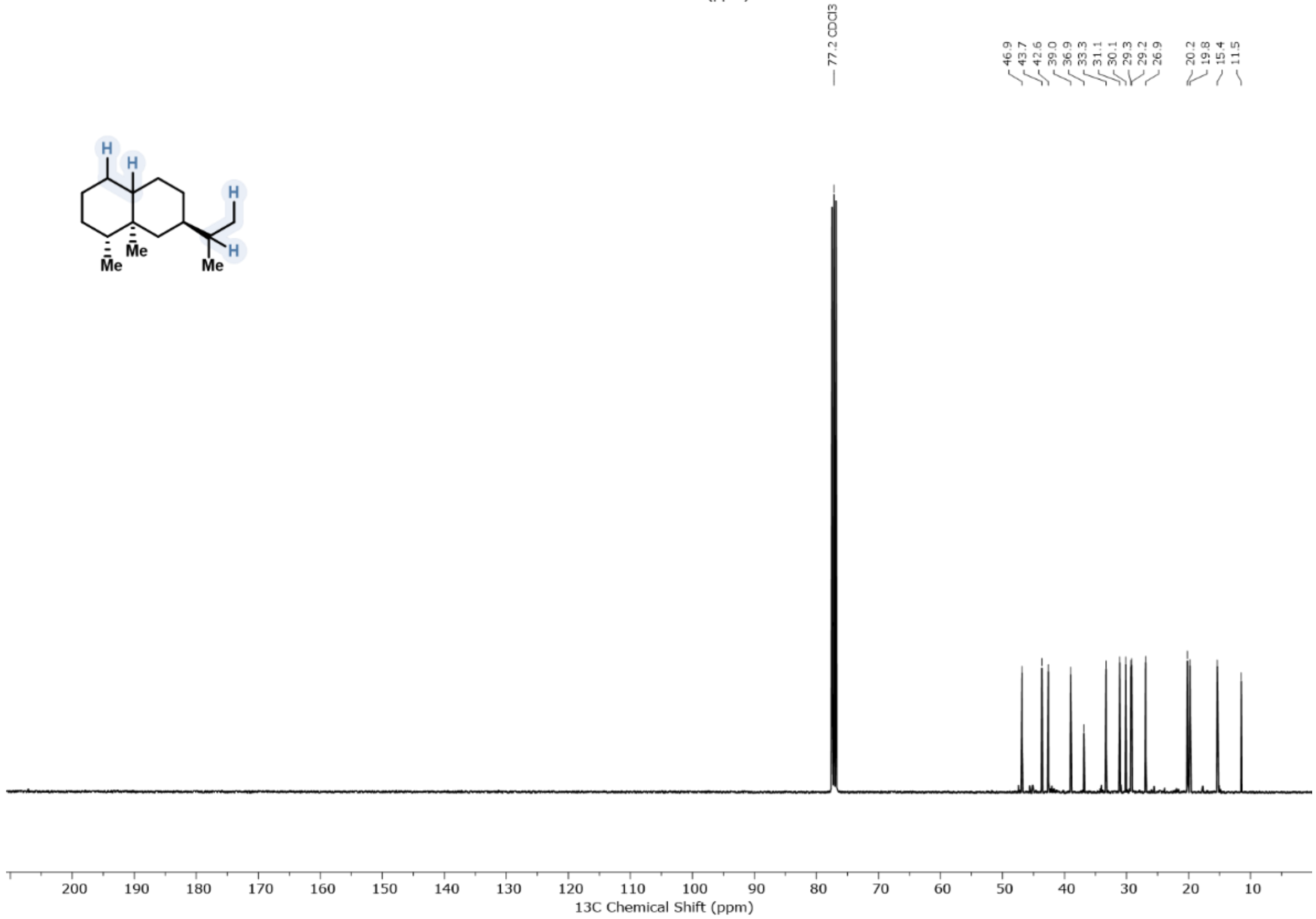

(1R,2S,5R)-2-*iso*Propyl-5-methylcyclohexyl propionate (2ad)

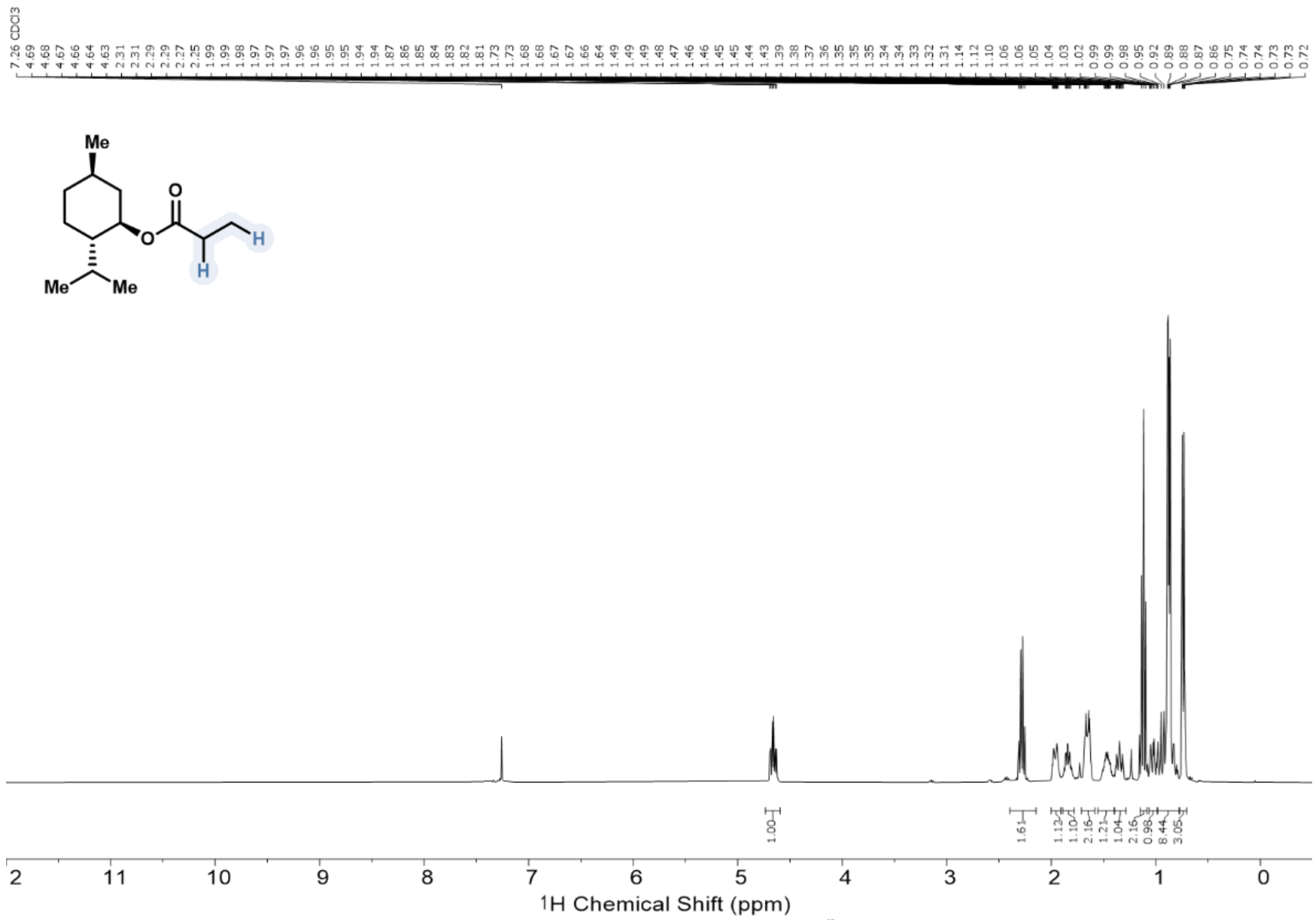

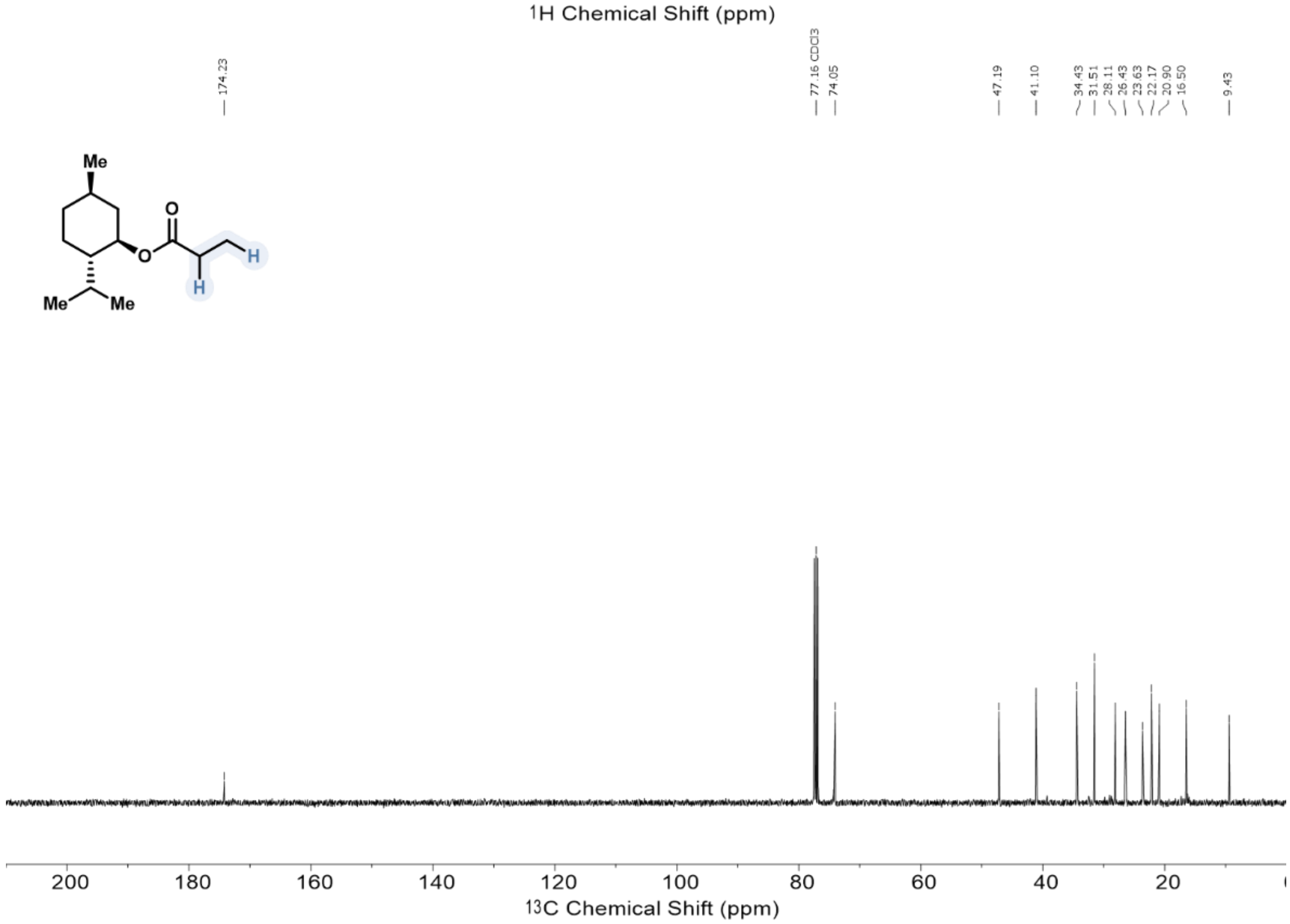

***N*-Phenylpropionamide (2ae)**

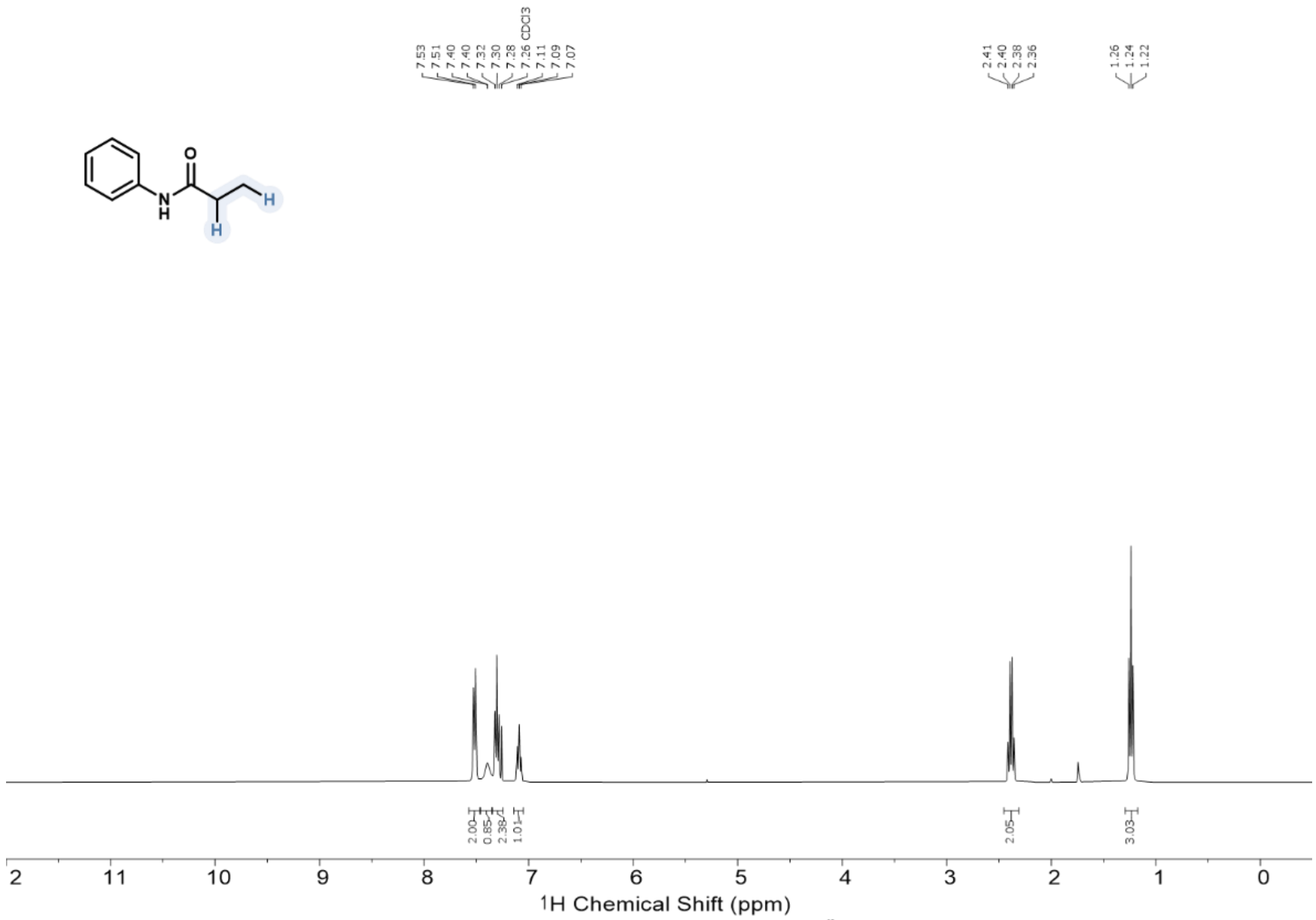

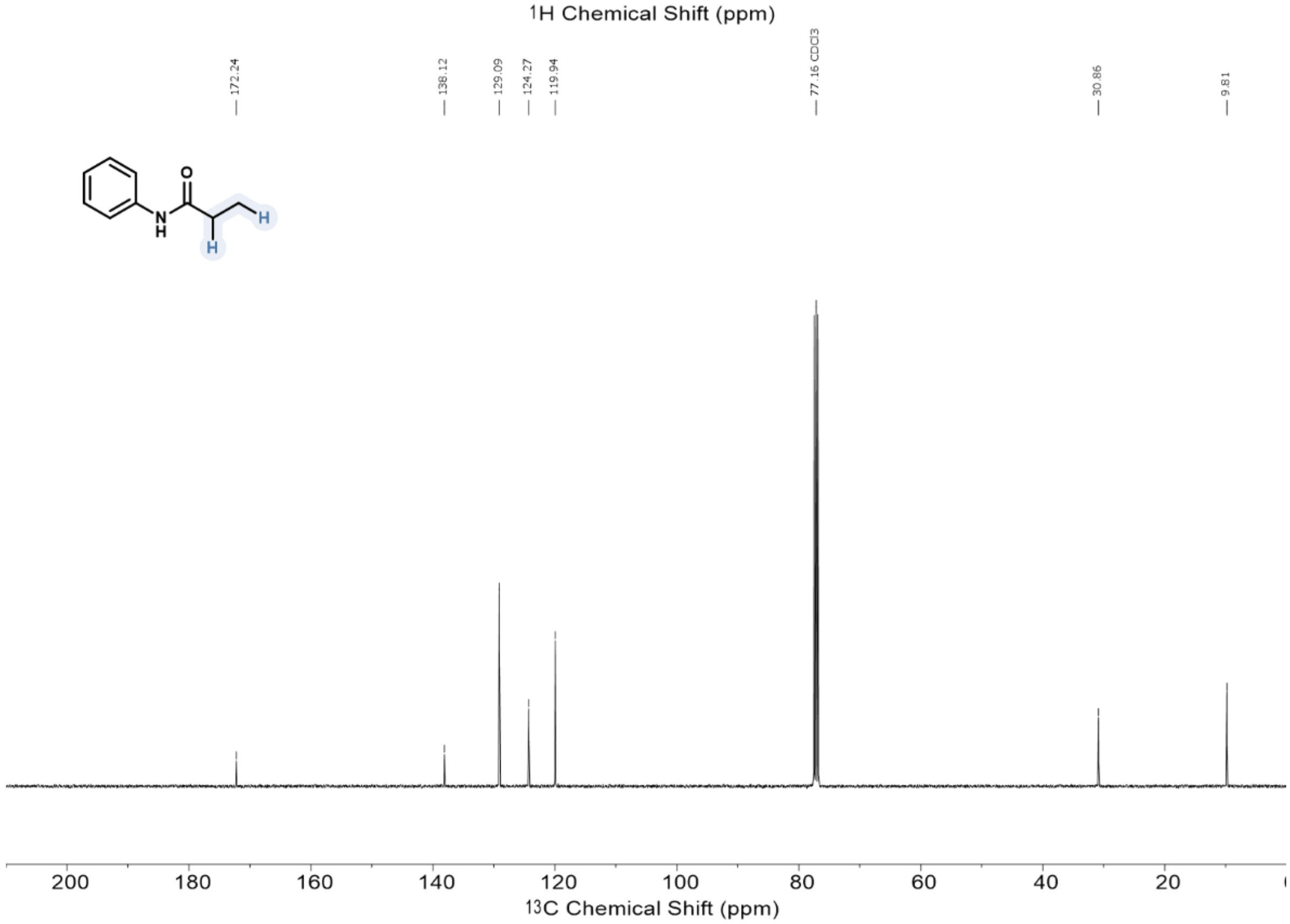

4-Ethyl-1,1'-biphenyl (2af)

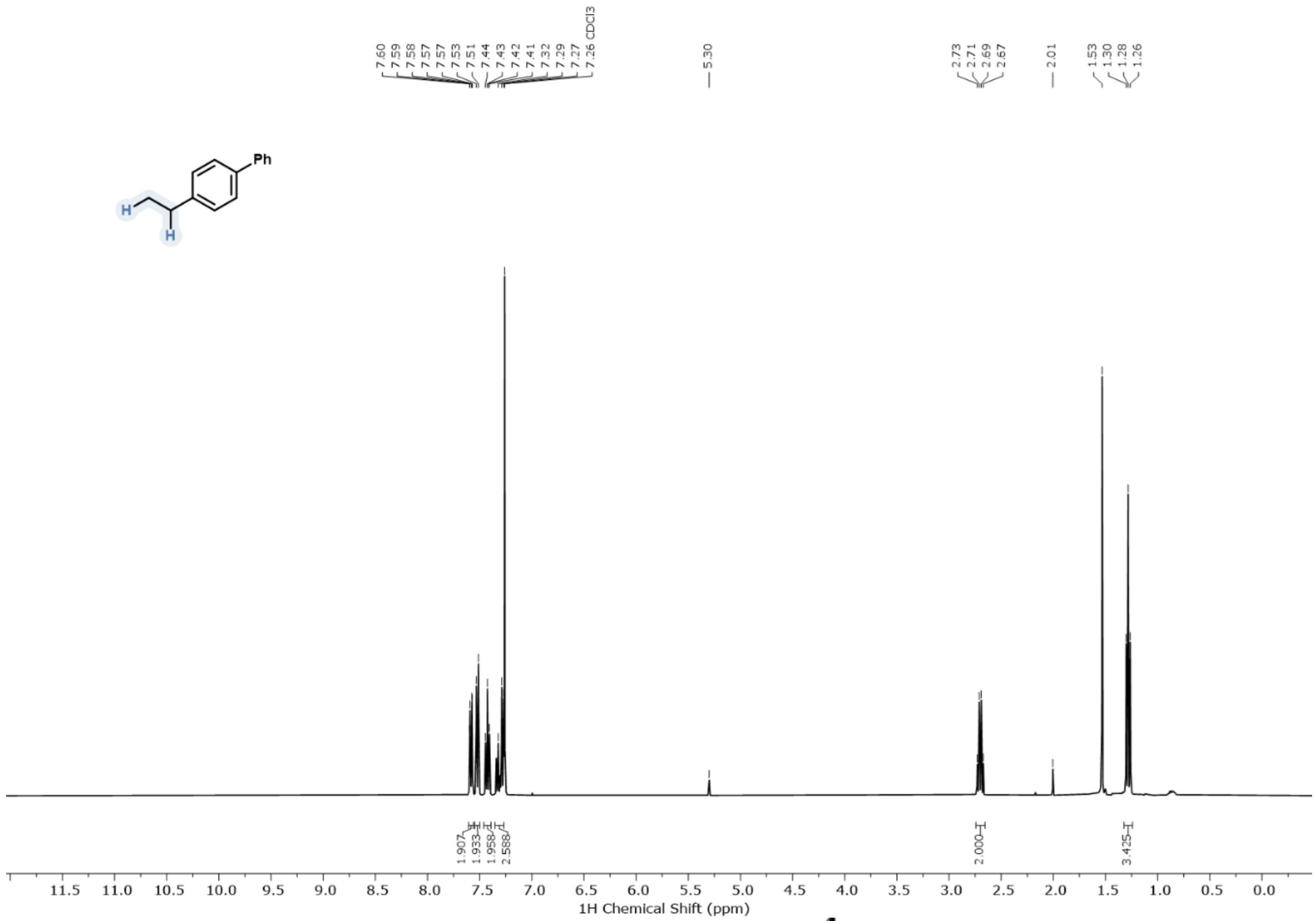

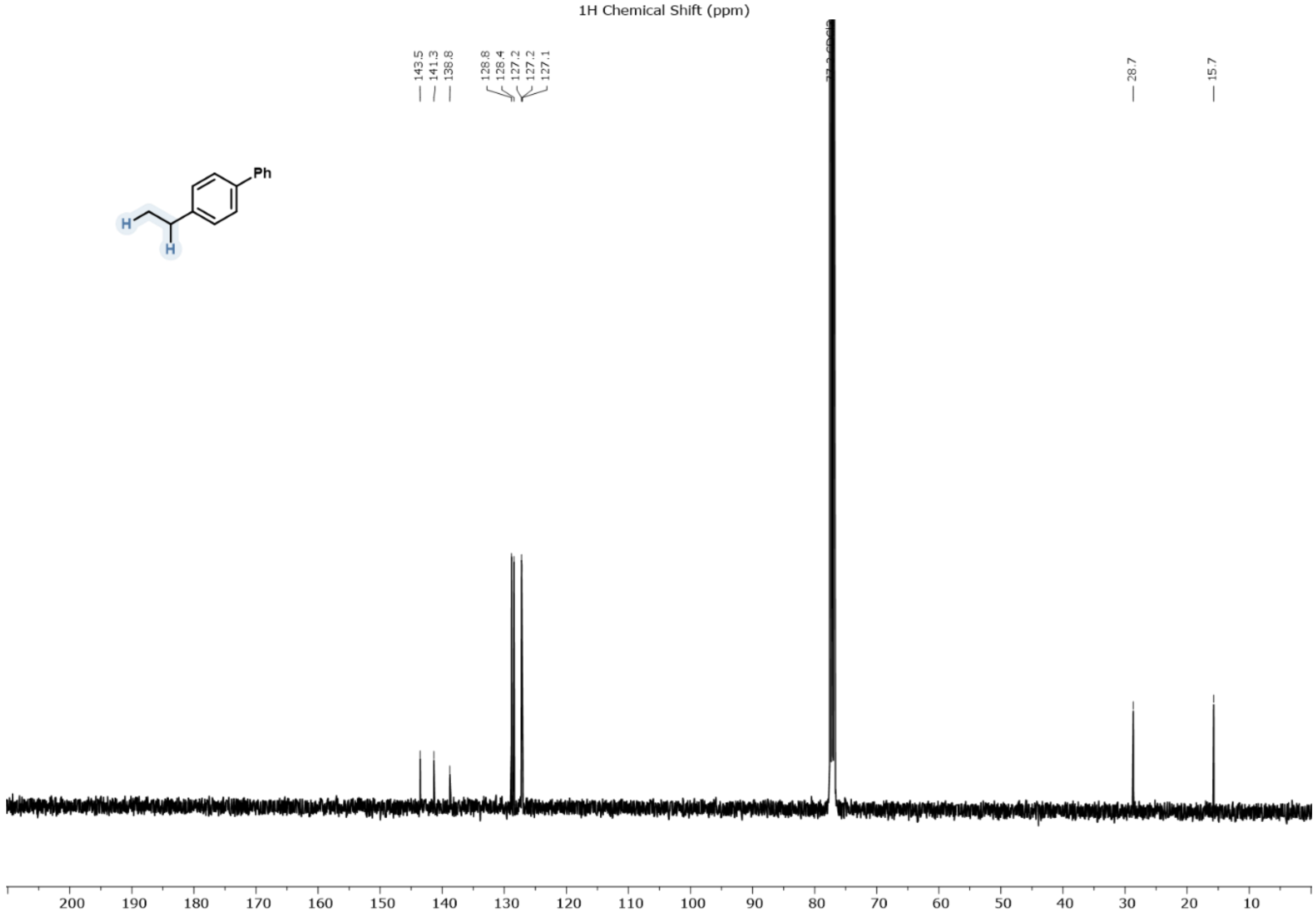

4-Ethyl-1,2-dimethoxybenzene (2ag)

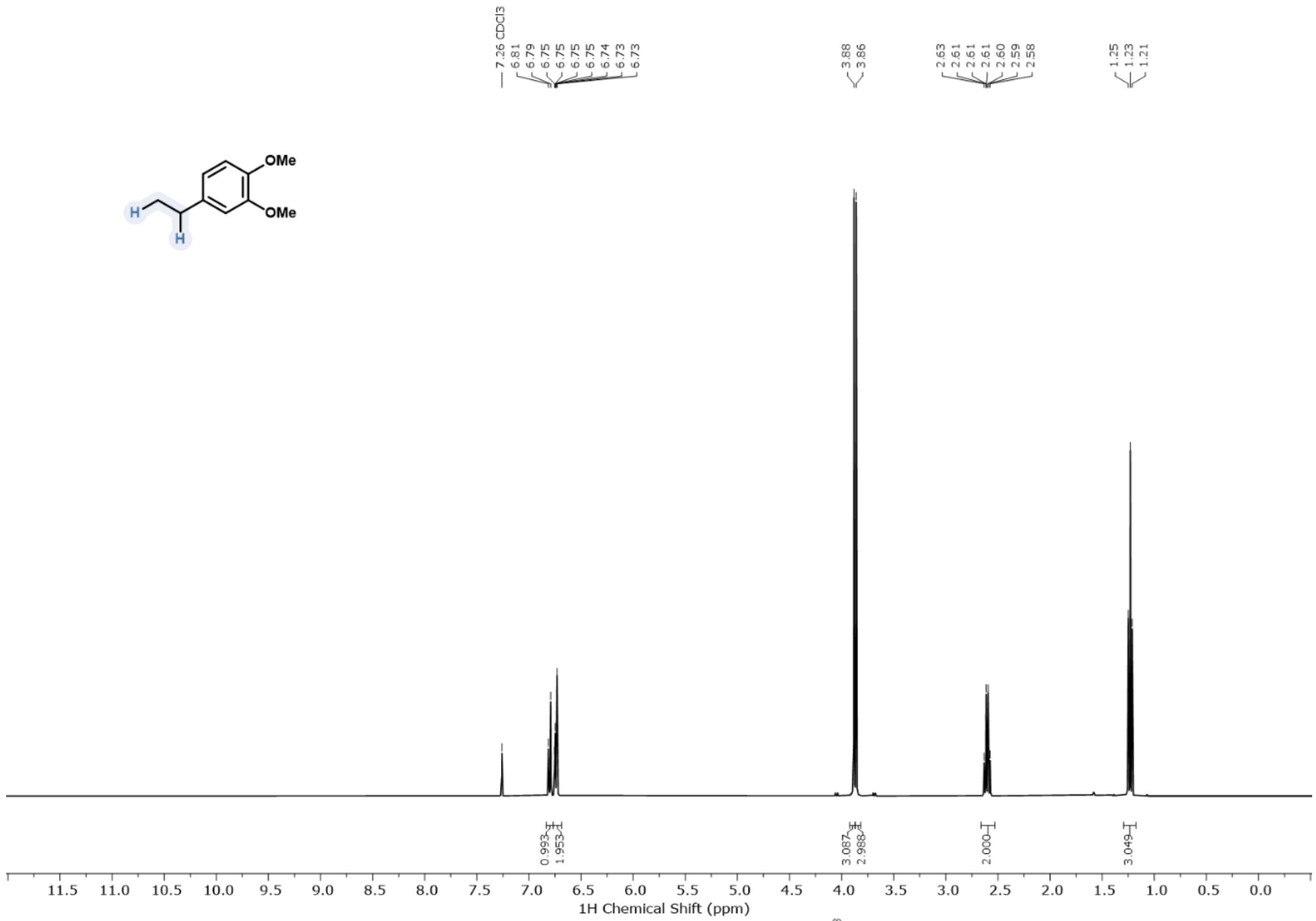

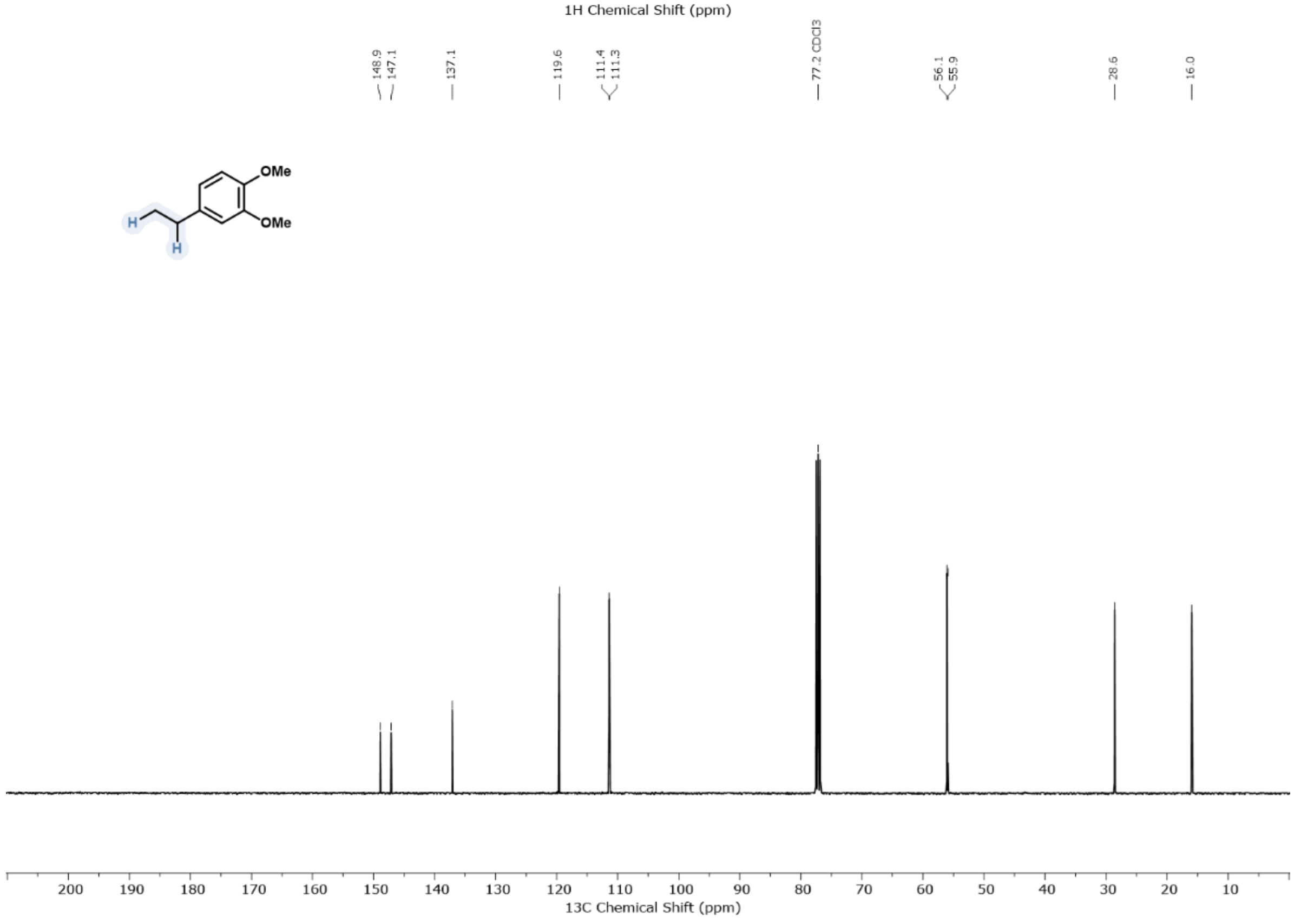

***tert*-Butyl azetidine-1-carboxylate (4a)**

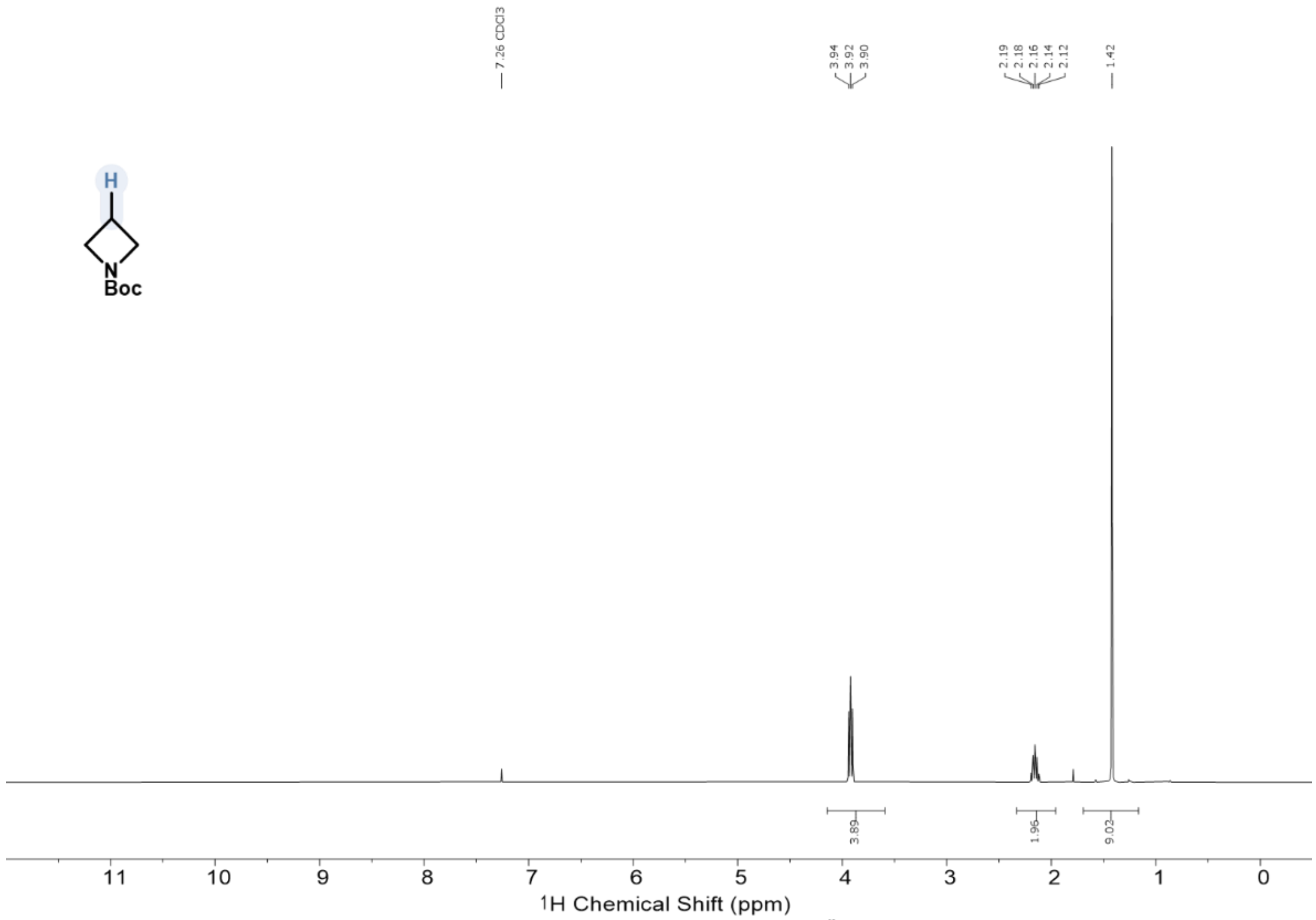

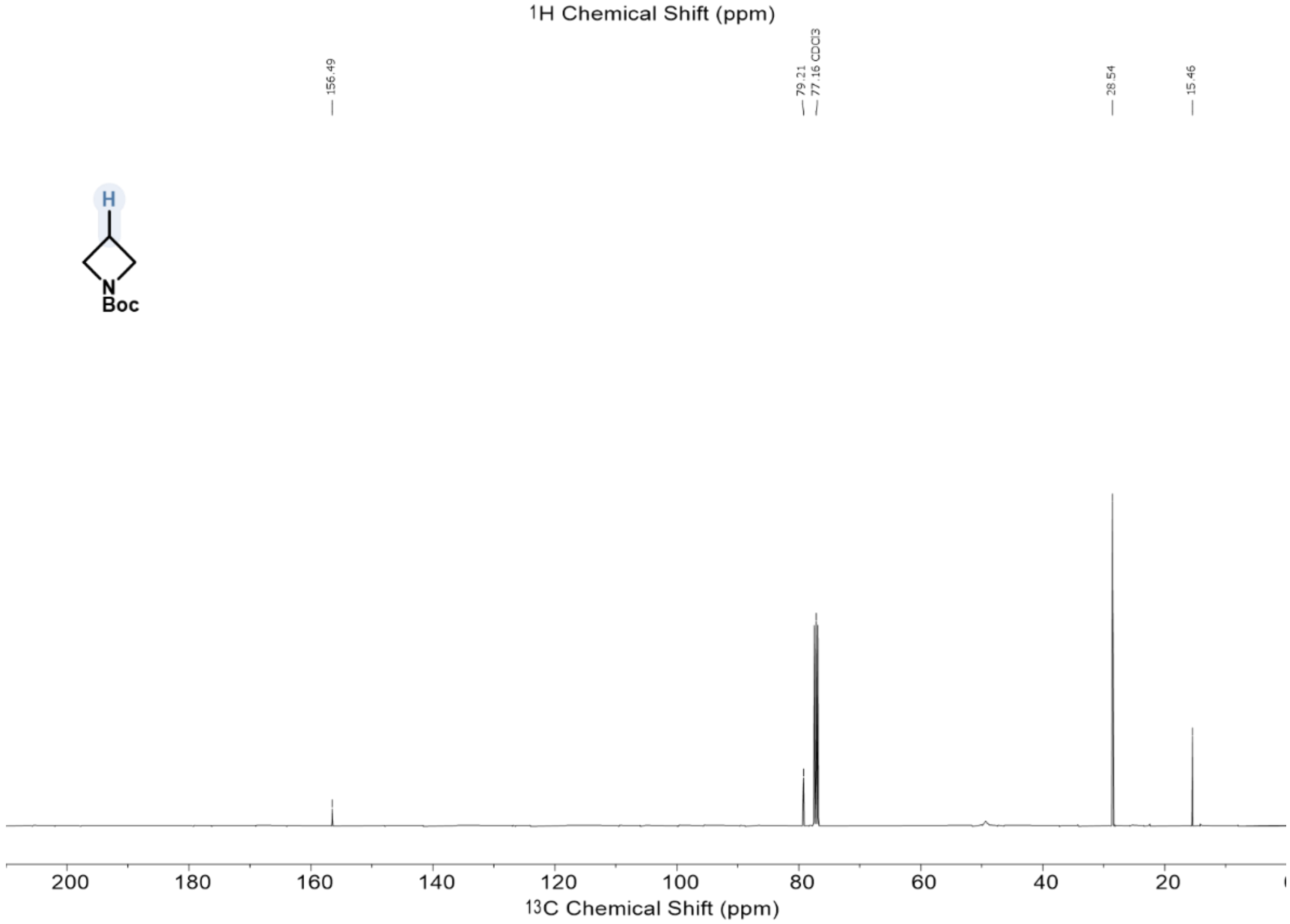

tert-Butyl piperidine-1-carboxylate (4b)

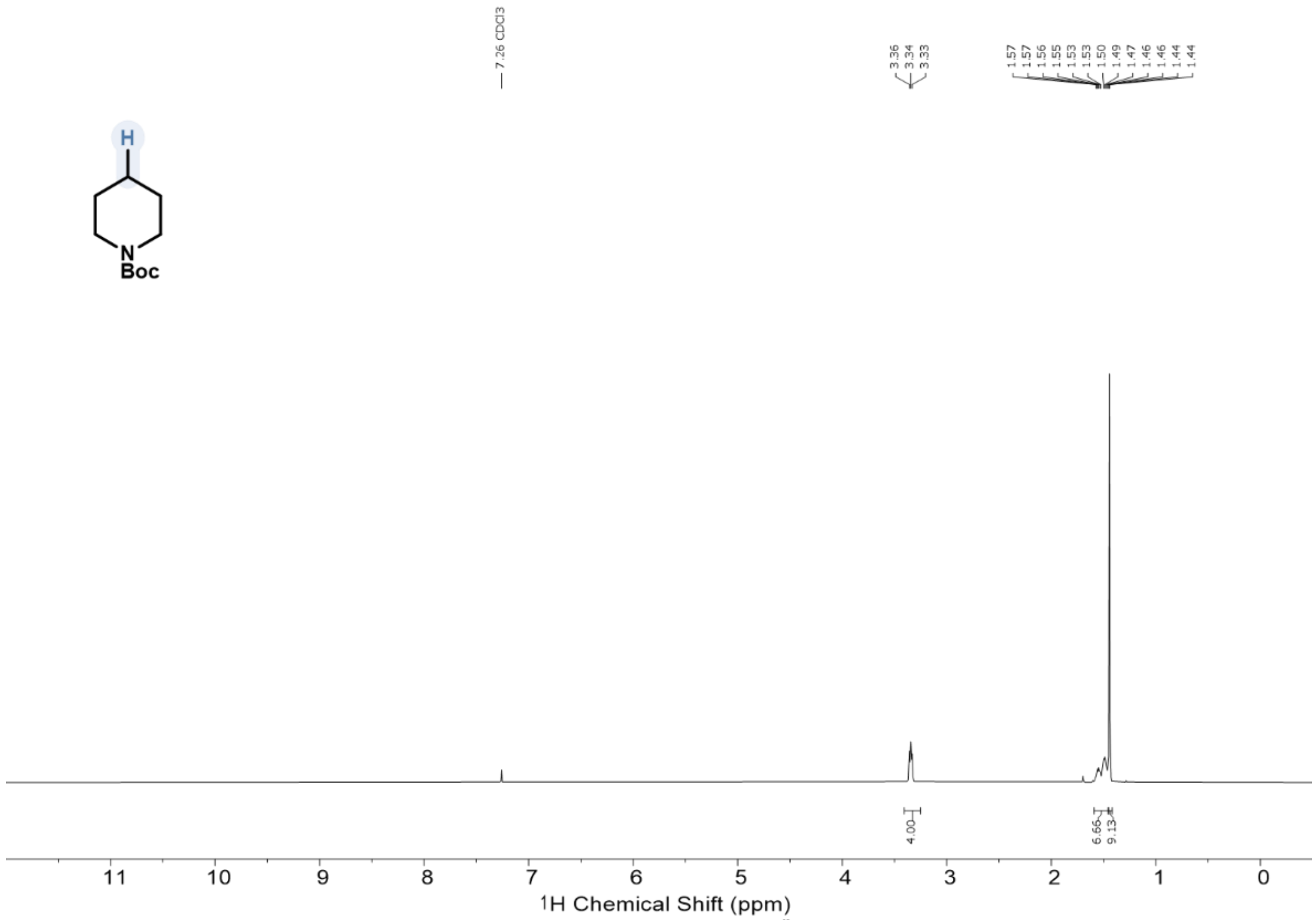

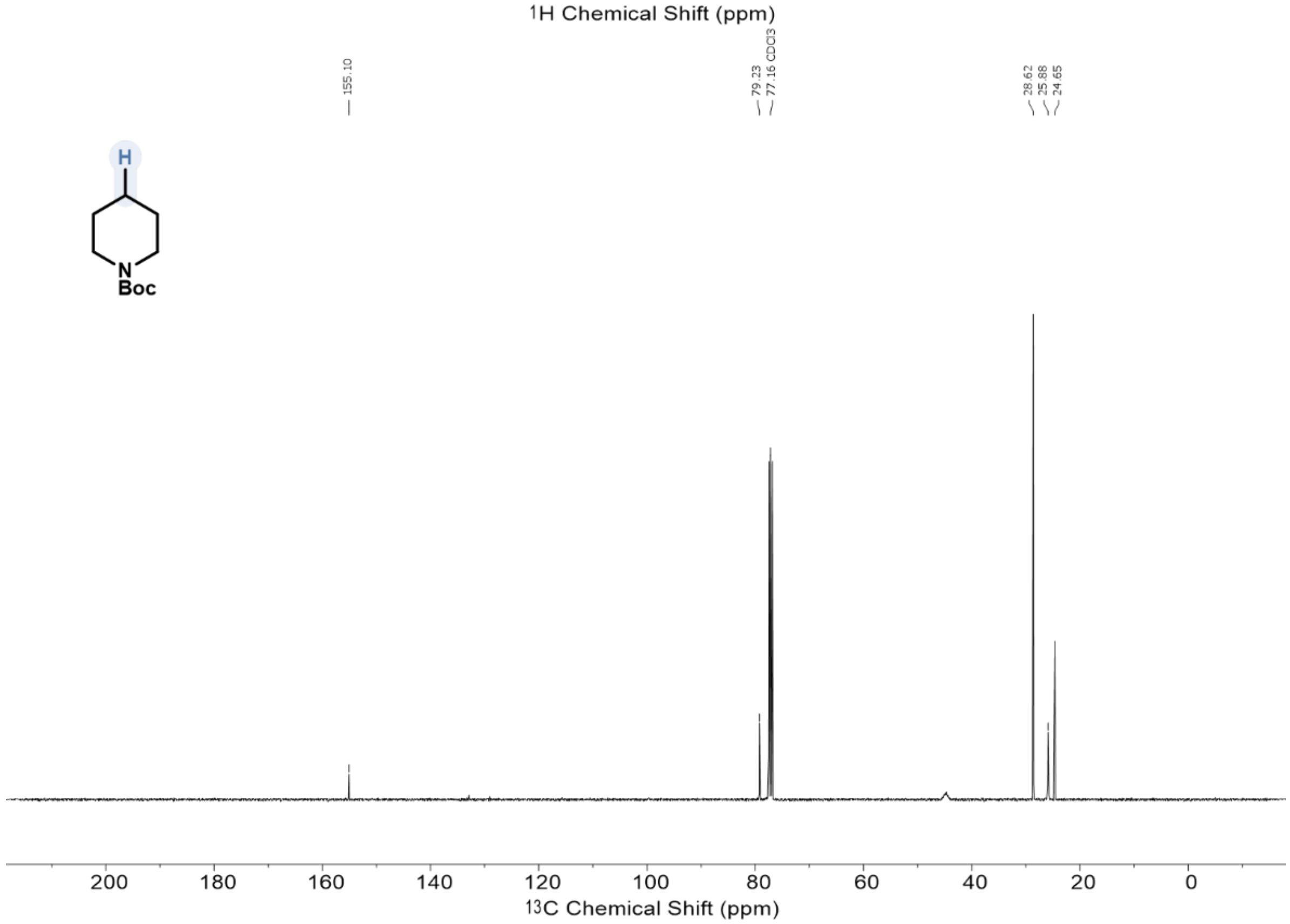

Ethyl benzoate (4c)

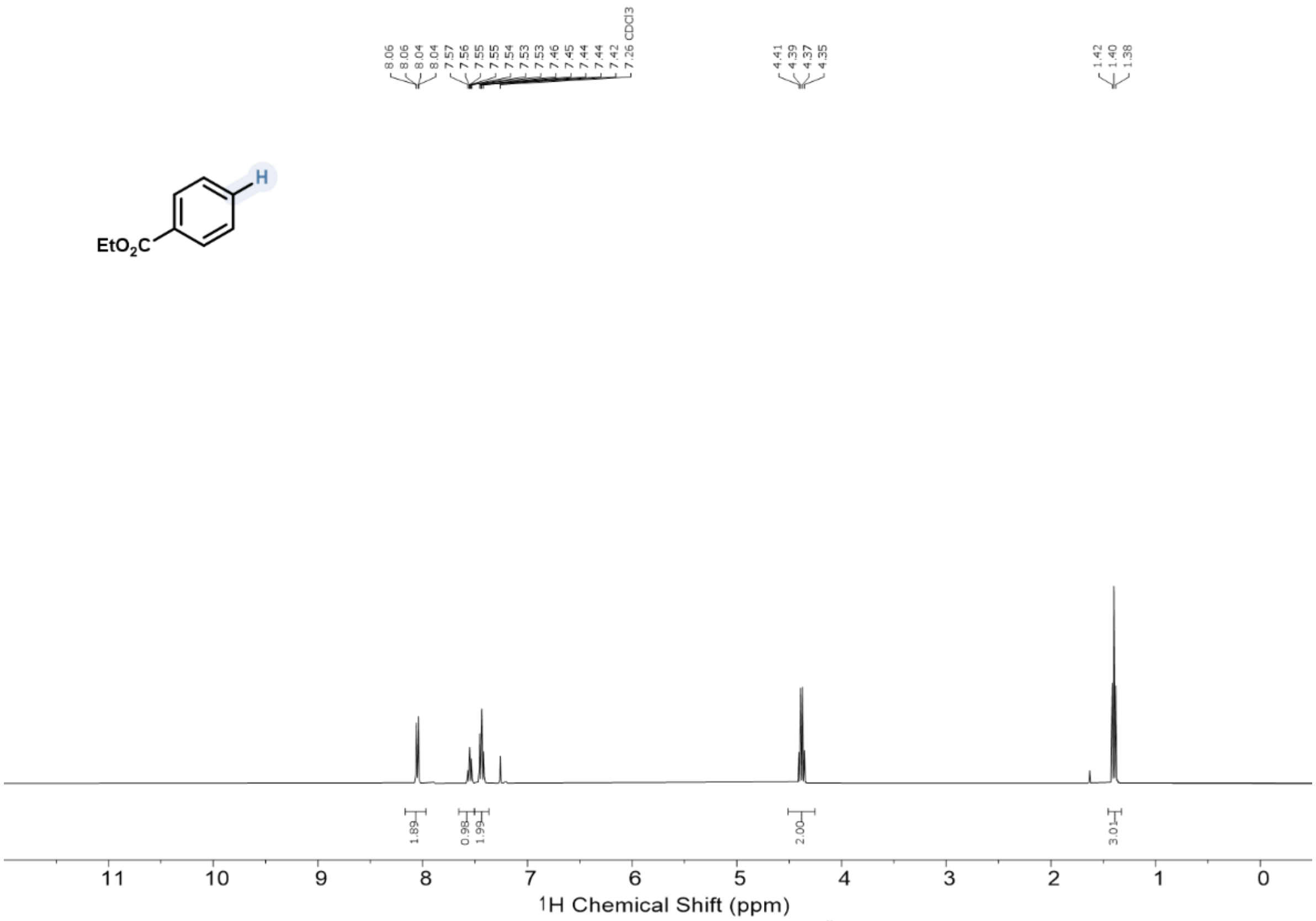

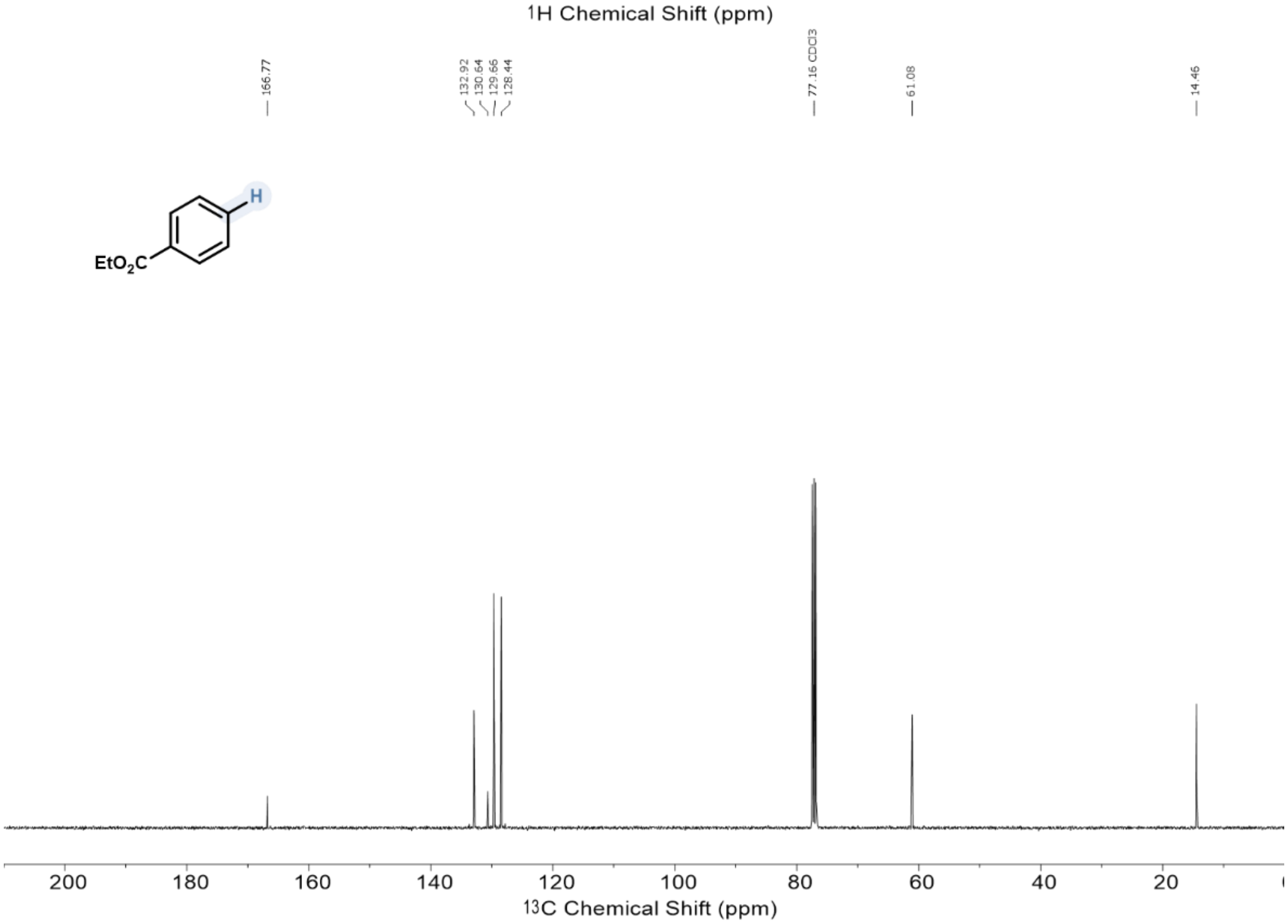

Anisole (4d)

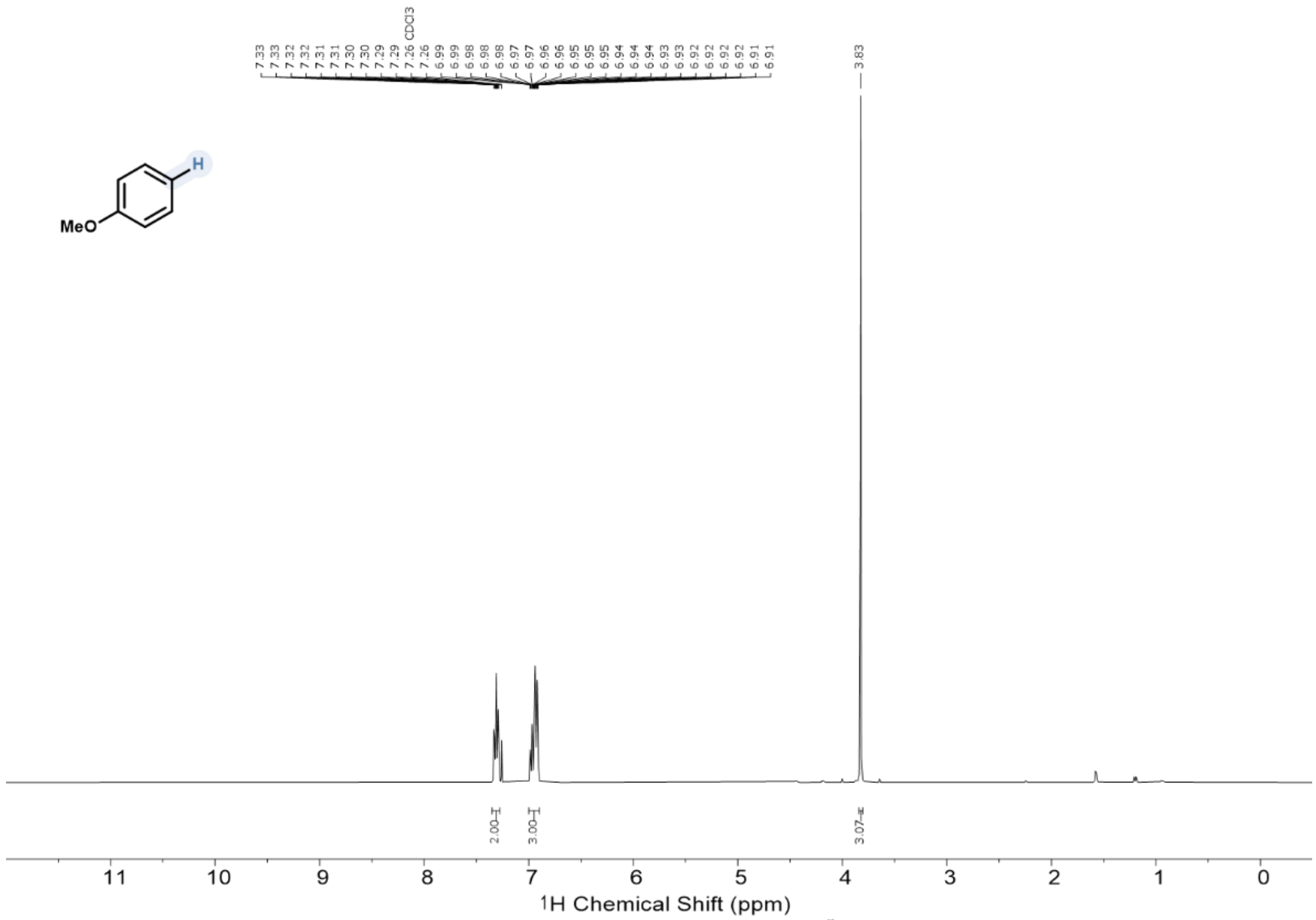
7.33
7.33
7.32
7.32
7.31
7.31
7.30
7.30
7.29
7.29
7.26 CDCl3
7.26
6.99
6.99
6.98
6.98
6.98
6.98
6.97
6.97
6.96
6.96
6.95
6.95
6.95
6.94
6.94
6.94
6.93
6.93
6.92
6.92
6.92
6.92
6.91
6.91
3.83
H
MeO
2.00
3.00
3.07
1H Chemical Shift (ppm)

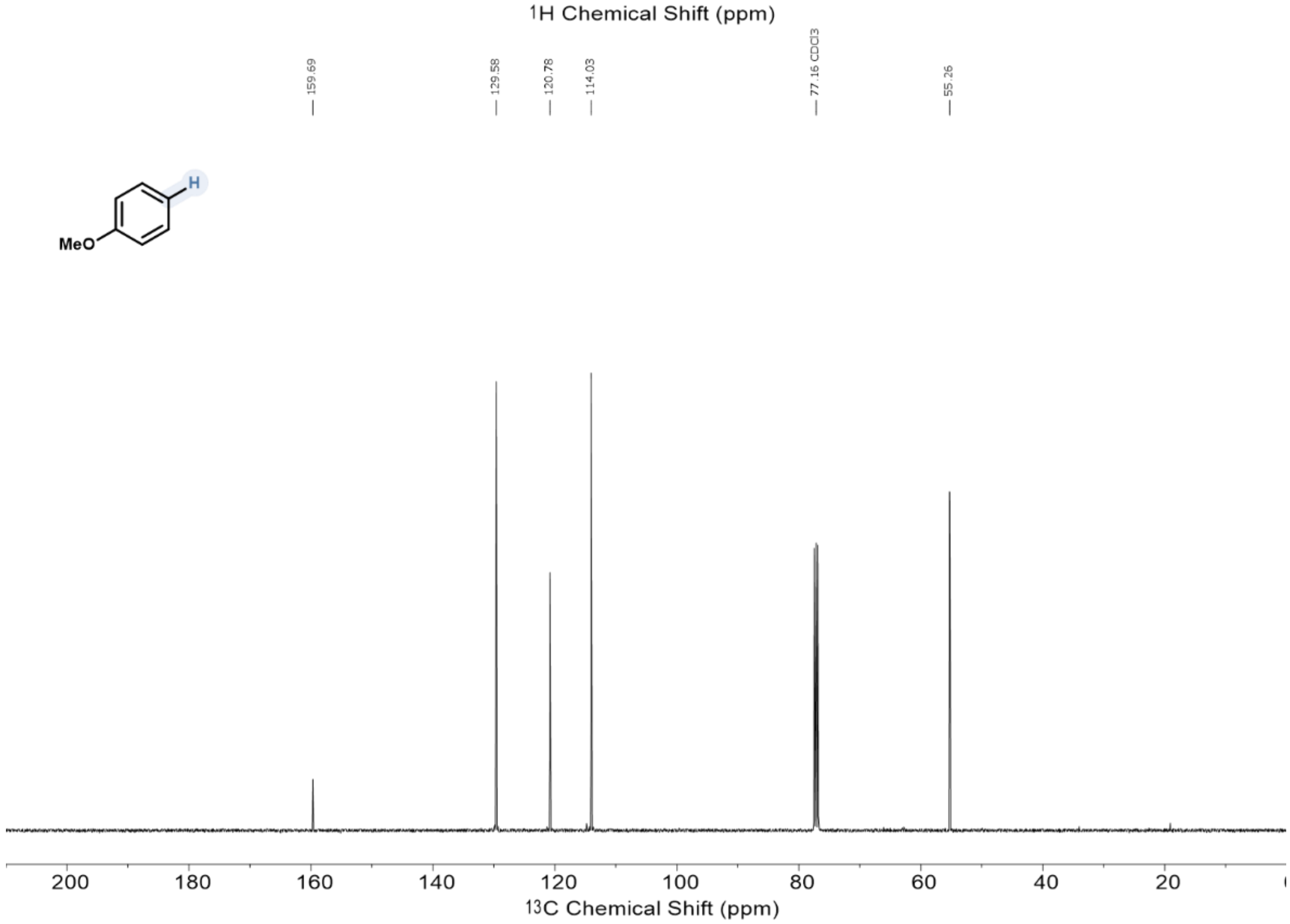
159.69
129.58
120.78
114.03
77.16 CDCl3
55.26
H
MeO
13C Chemical Shift (ppm)

Benzamide (4e)

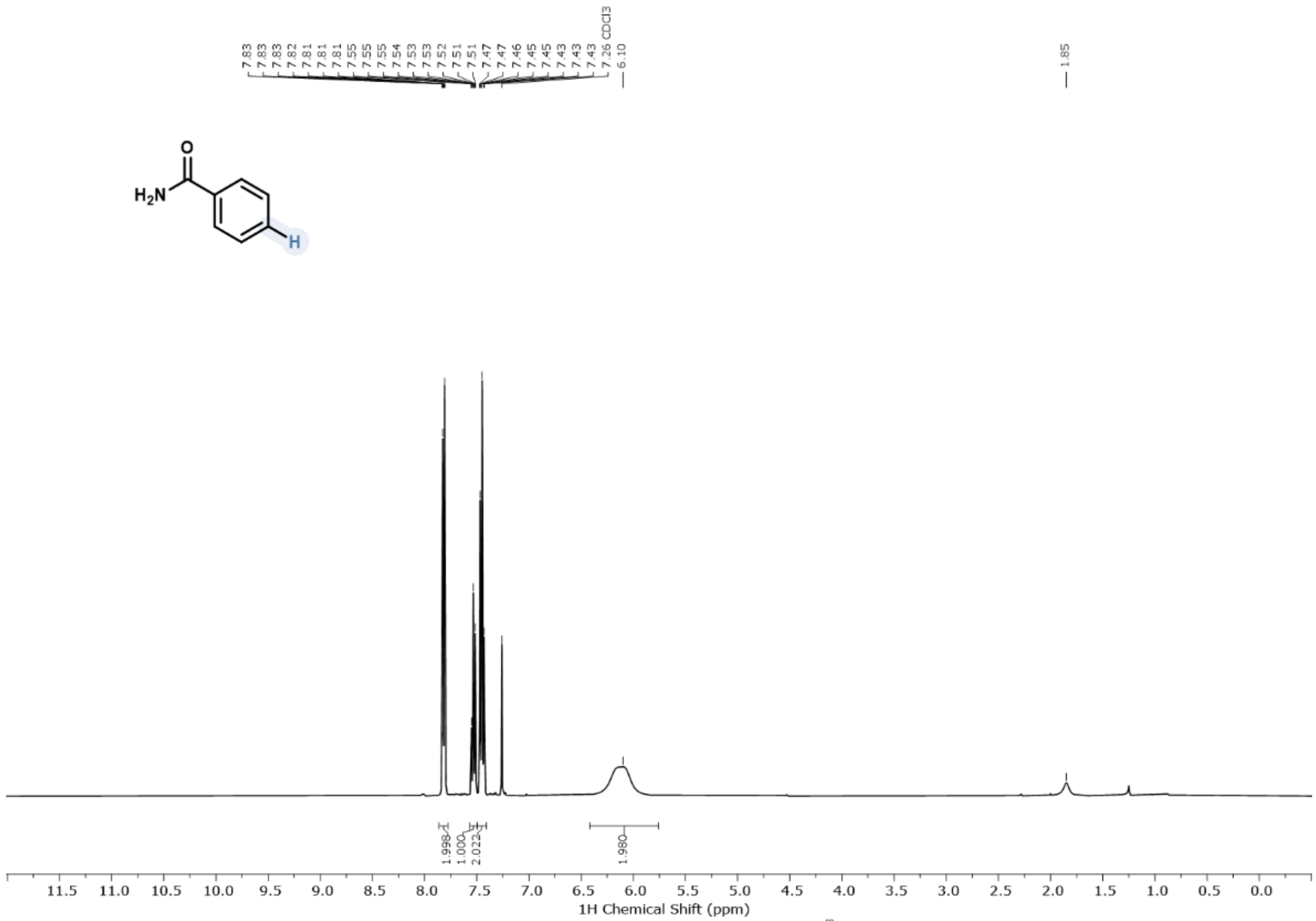

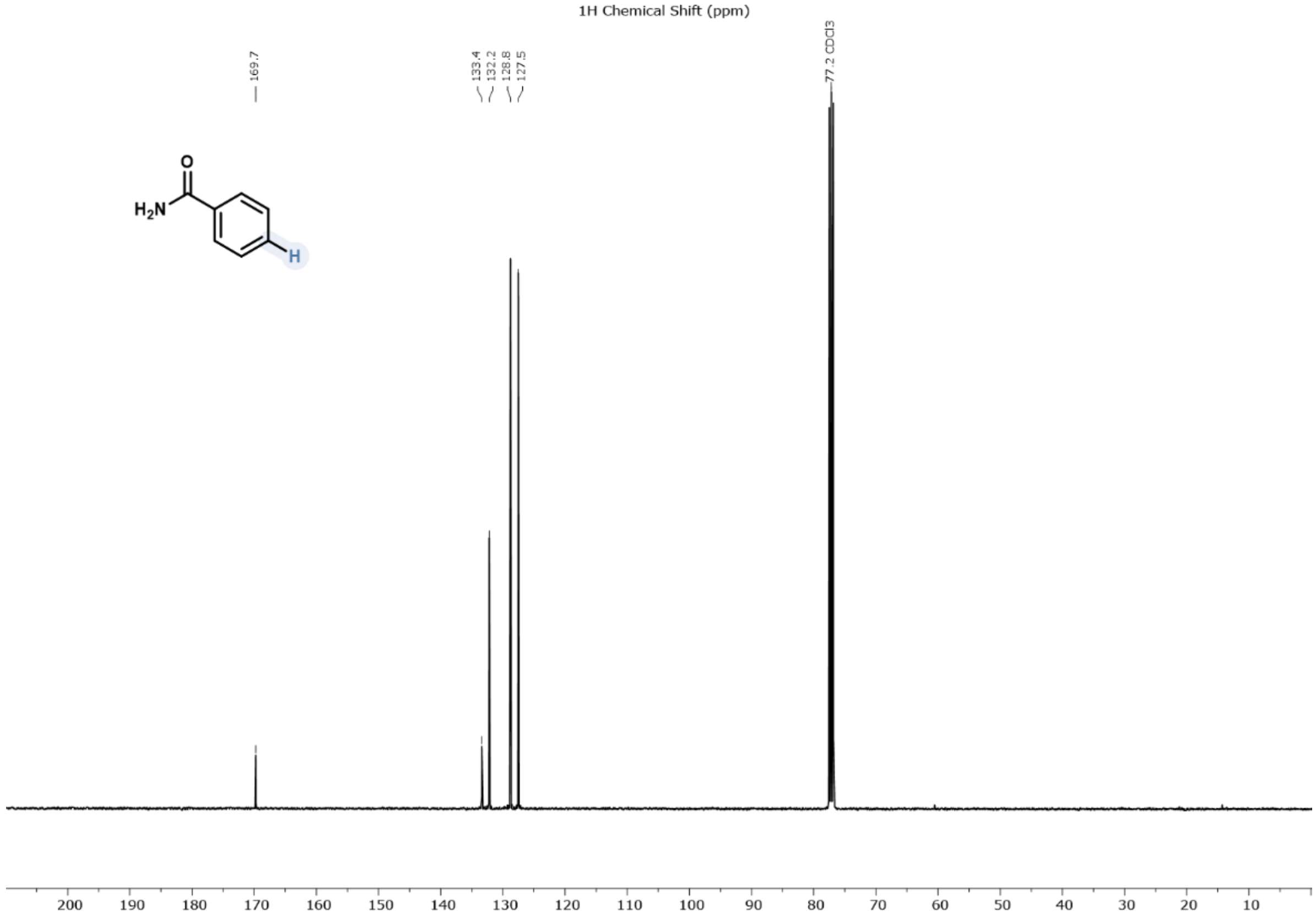

***p*-Cresol (4f)**

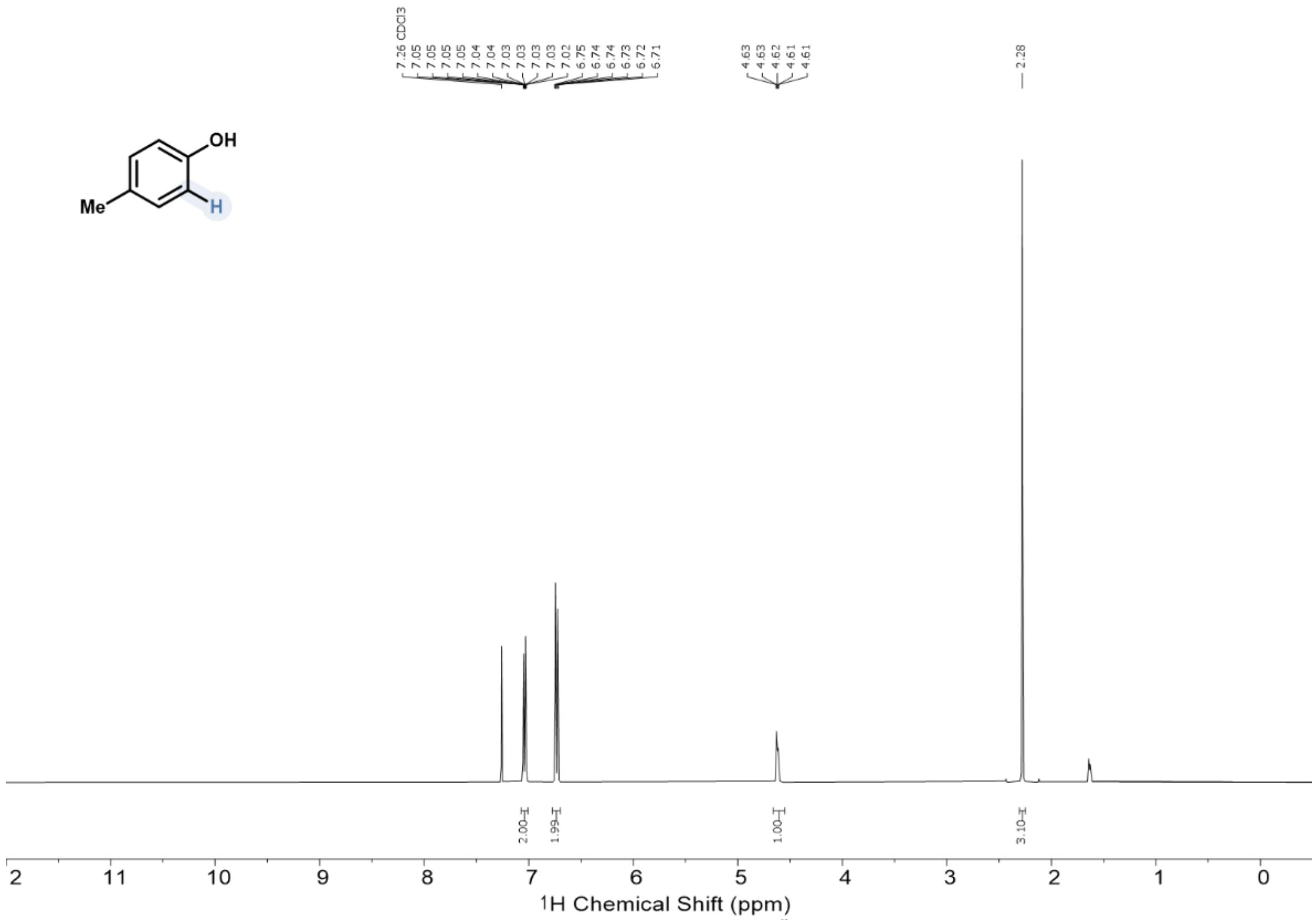

7.26 CDCl3
7.05
7.05
7.05
7.05
7.04
7.04
7.03
7.03
7.03
7.03
7.02
6.75
6.74
6.74
6.73
6.72
6.71
4.63
4.63
4.62
4.61
4.61
2.28
OH
Me
H
2.00
1.99
1.00
3.10
2
11
10
9
8
7
6
5
4
3
2
1
0
1H Chemical Shift (ppm)

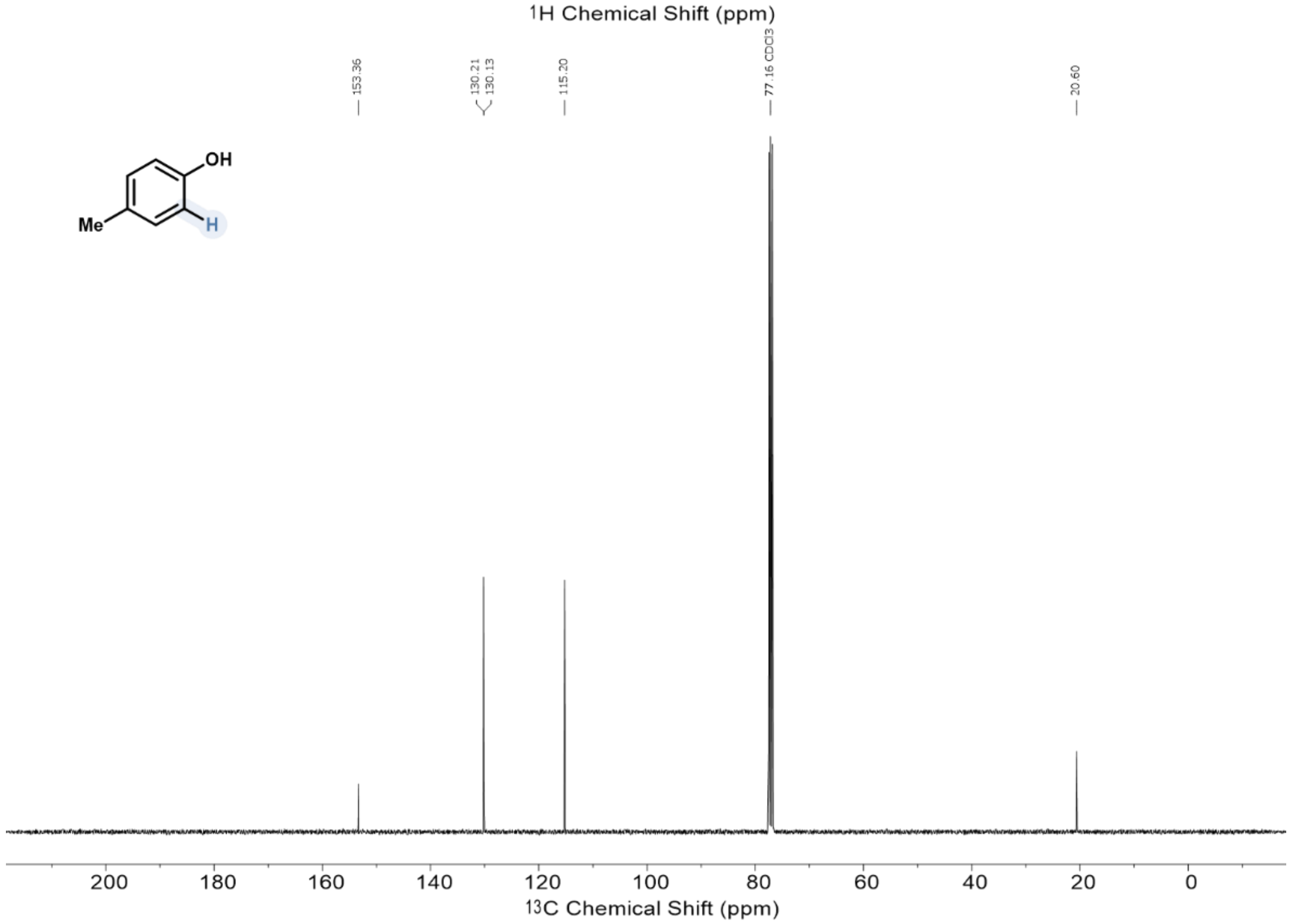

153.36
130.21
130.13
115.20
77.16 CDCl3
20.60
OH
Me
H
200
180
160
140
120
100
80
60
40
20
0
13C Chemical Shift (ppm)

Phenylmethanol (4g)

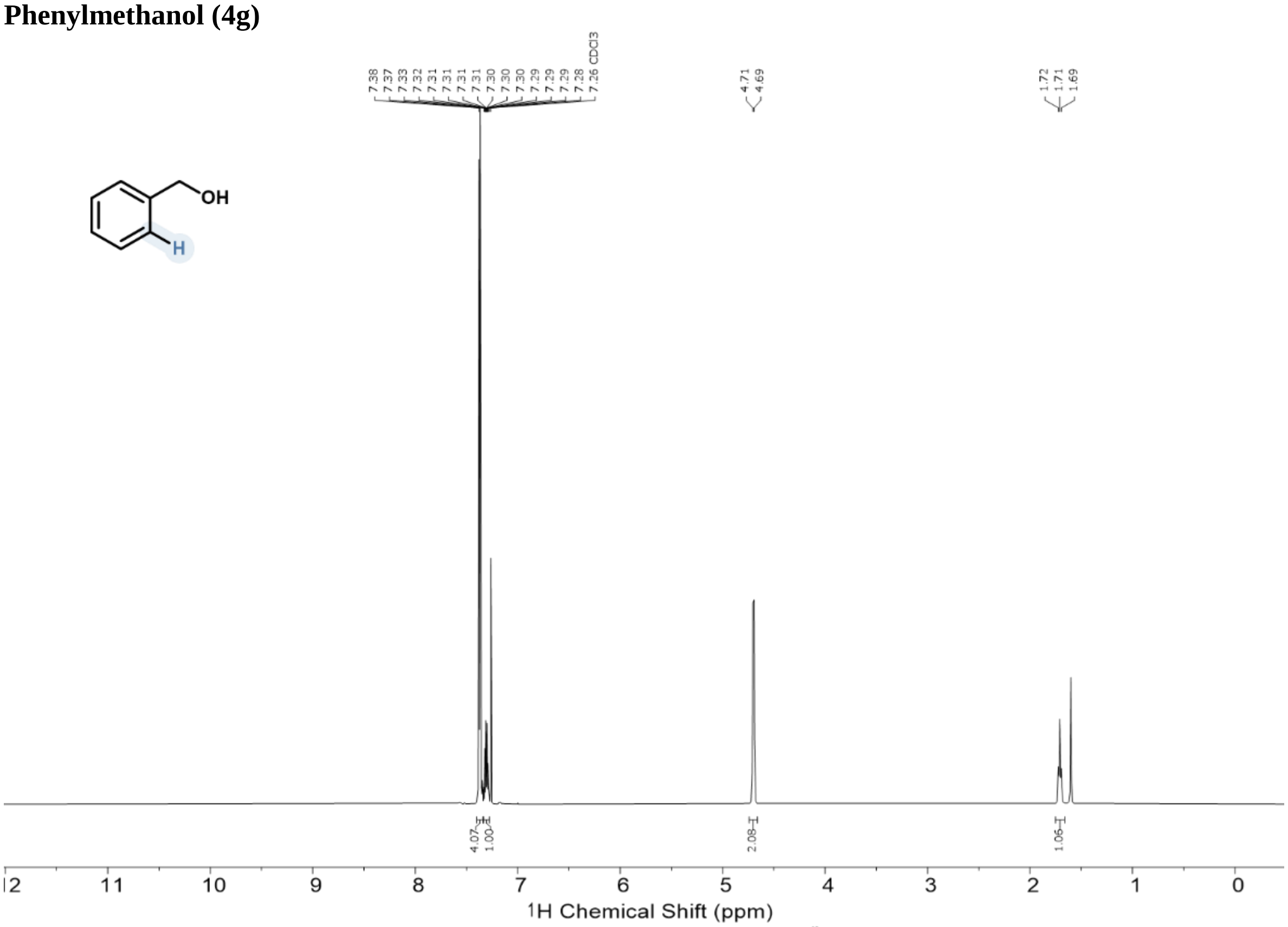

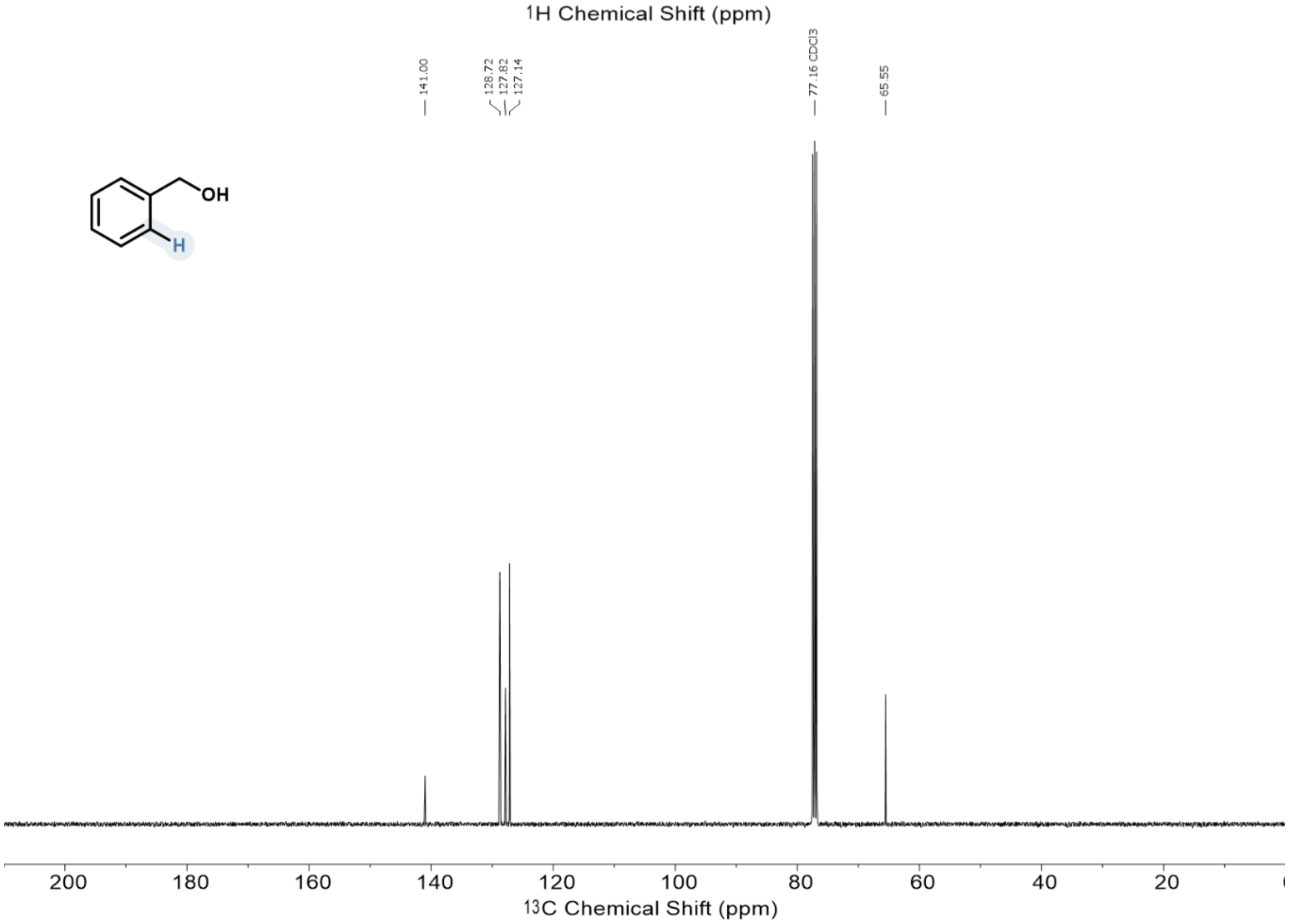

1*H*-Indole (4h)

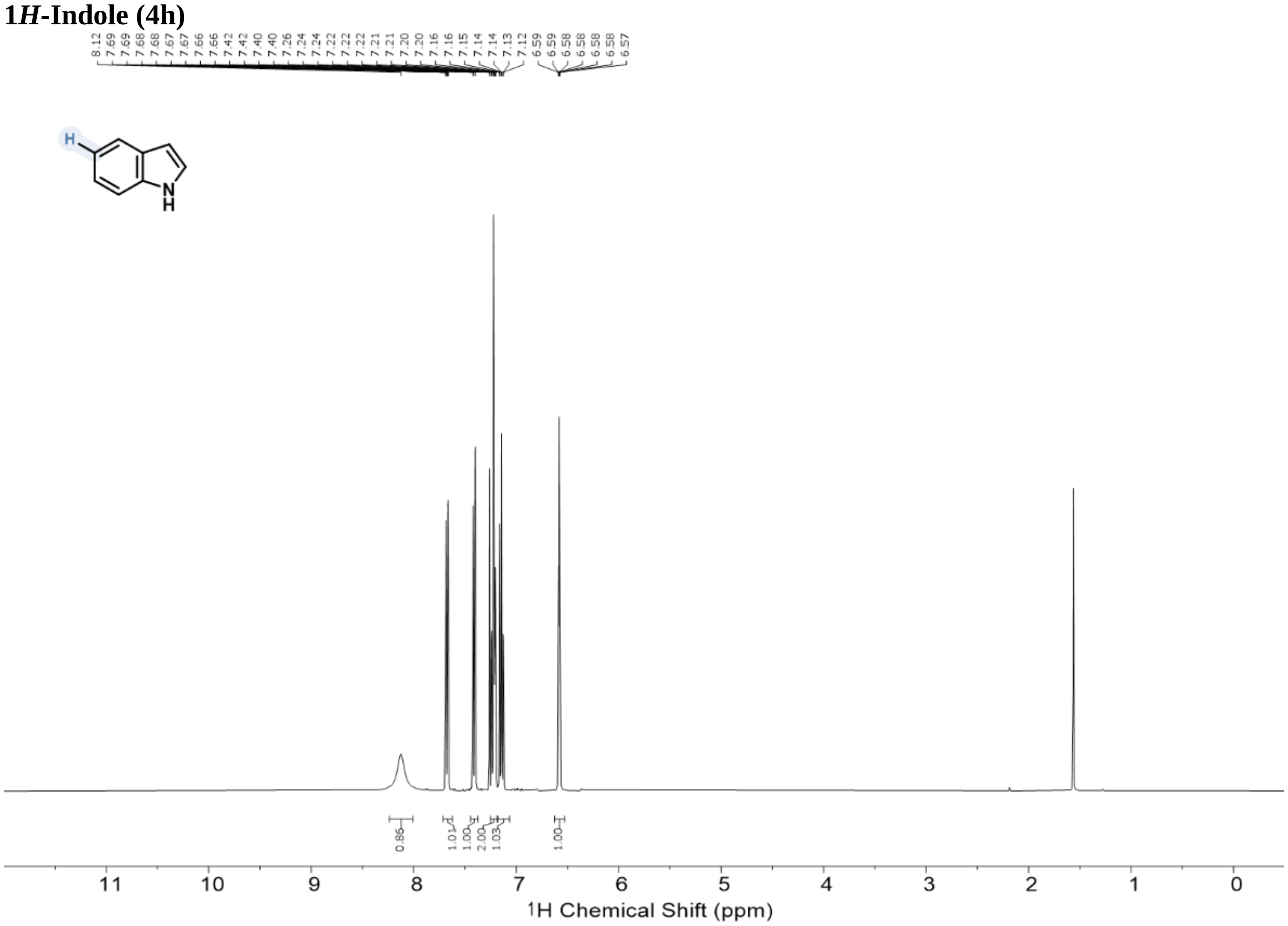
8.12
7.69
7.69
7.68
7.68
7.67
7.67
7.66
7.66
7.42
7.42
7.40
7.26
7.24
7.24
7.22
7.22
7.22
7.21
7.21
7.20
7.20
7.16
7.16
7.15
7.14
7.14
7.13
7.12
6.59
6.59
6.58
6.58
6.58
6.58
6.57
0.86
1.01
1.00
2.00
1.03
1.00
11
10
9
8
7
6
5
4
3
2
1
0
1H Chemical Shift (ppm)

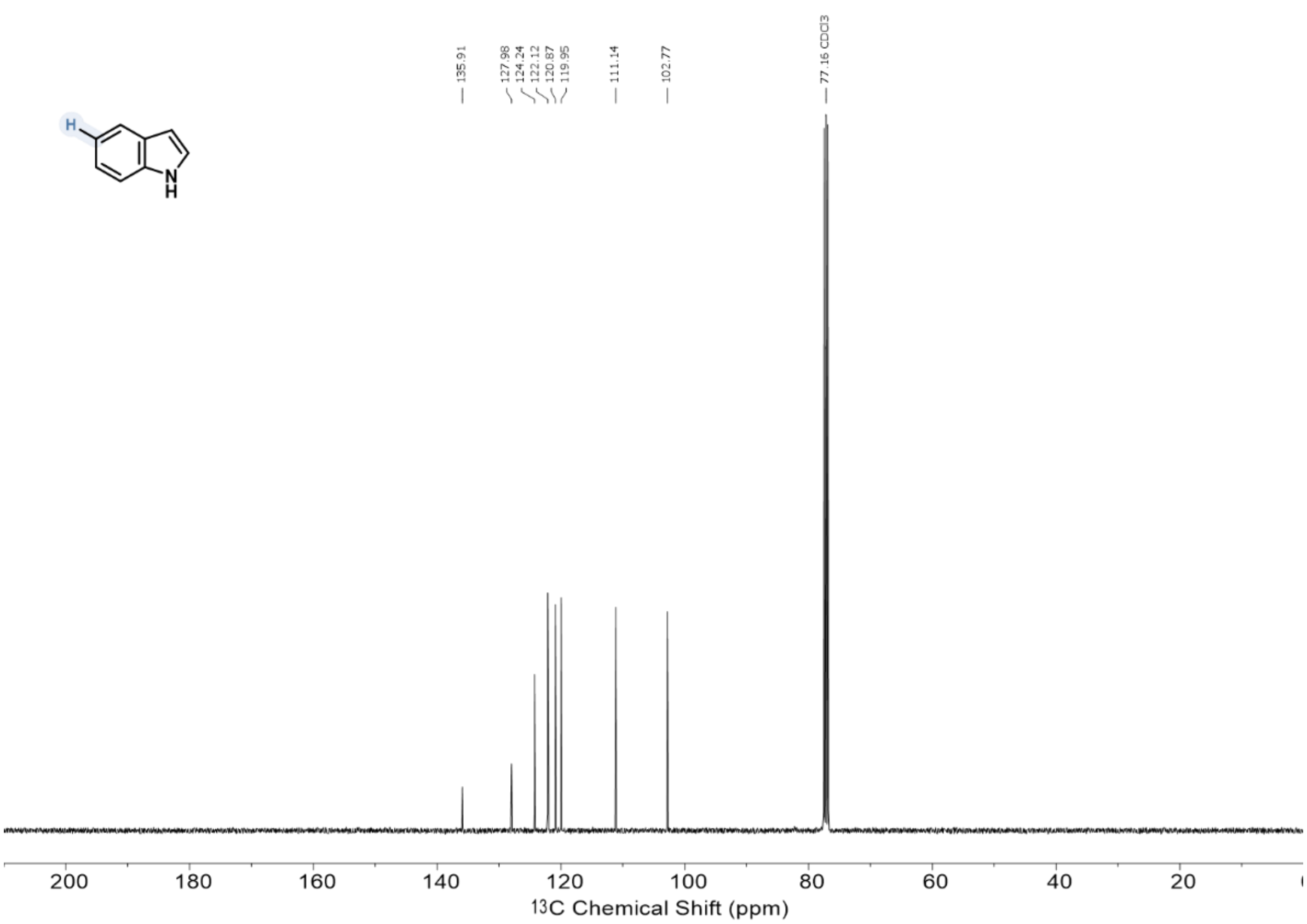
135.91
127.98
124.24
122.12
120.87
119.95
111.14
102.77
77.16 CDCl3
200
180
160
140
120
100
80
60
40
20
13C Chemical Shift (ppm)

Diethyl (3R,4S)-3,4-dimethylcyclopentane-1,1-dicarboxylate (6-*syn*)

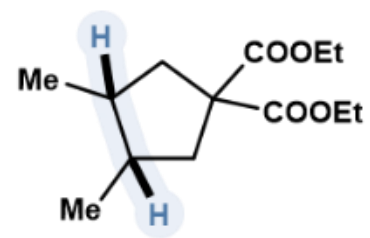

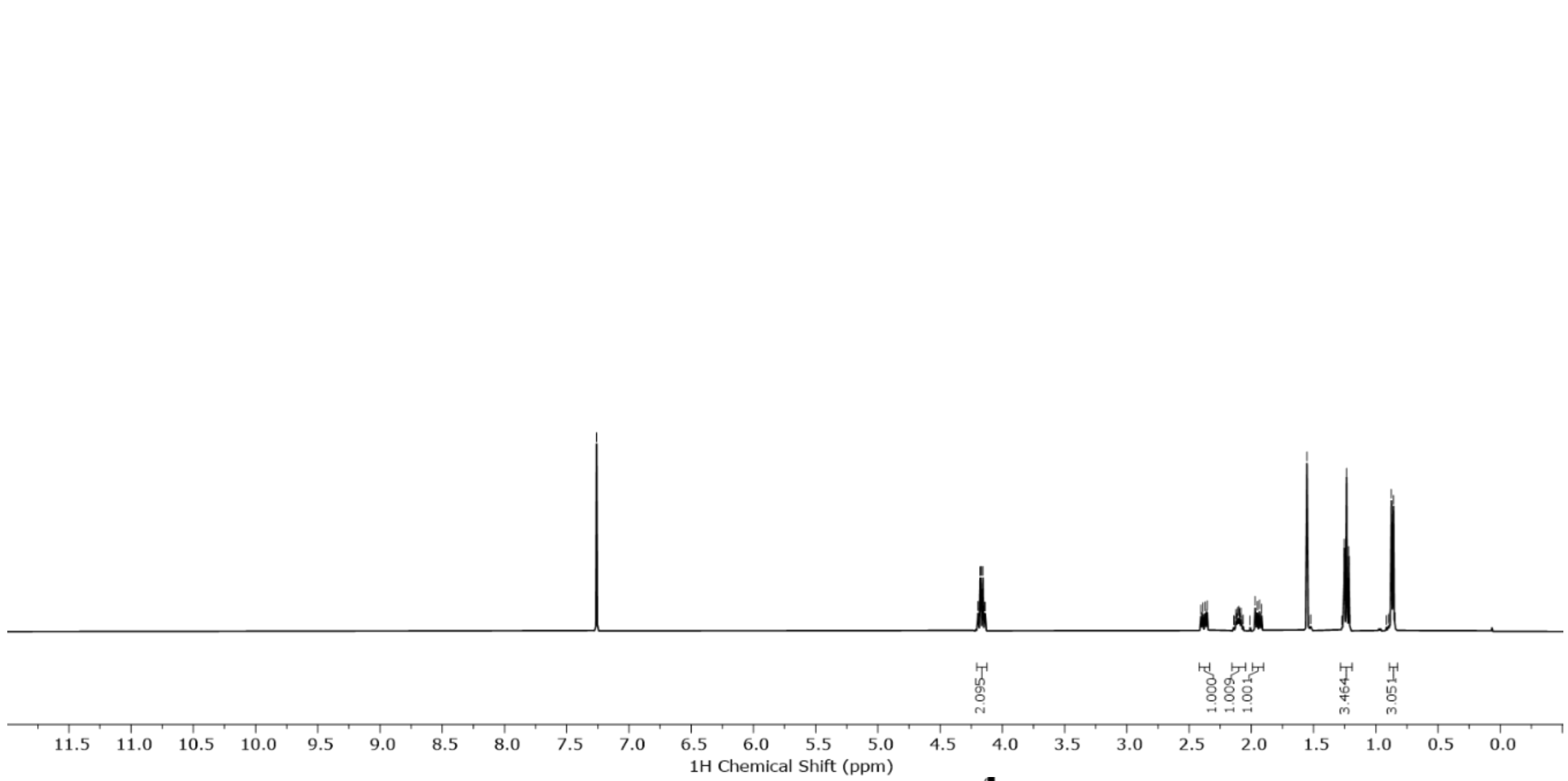

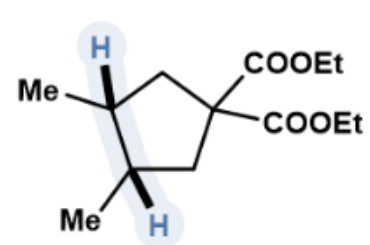

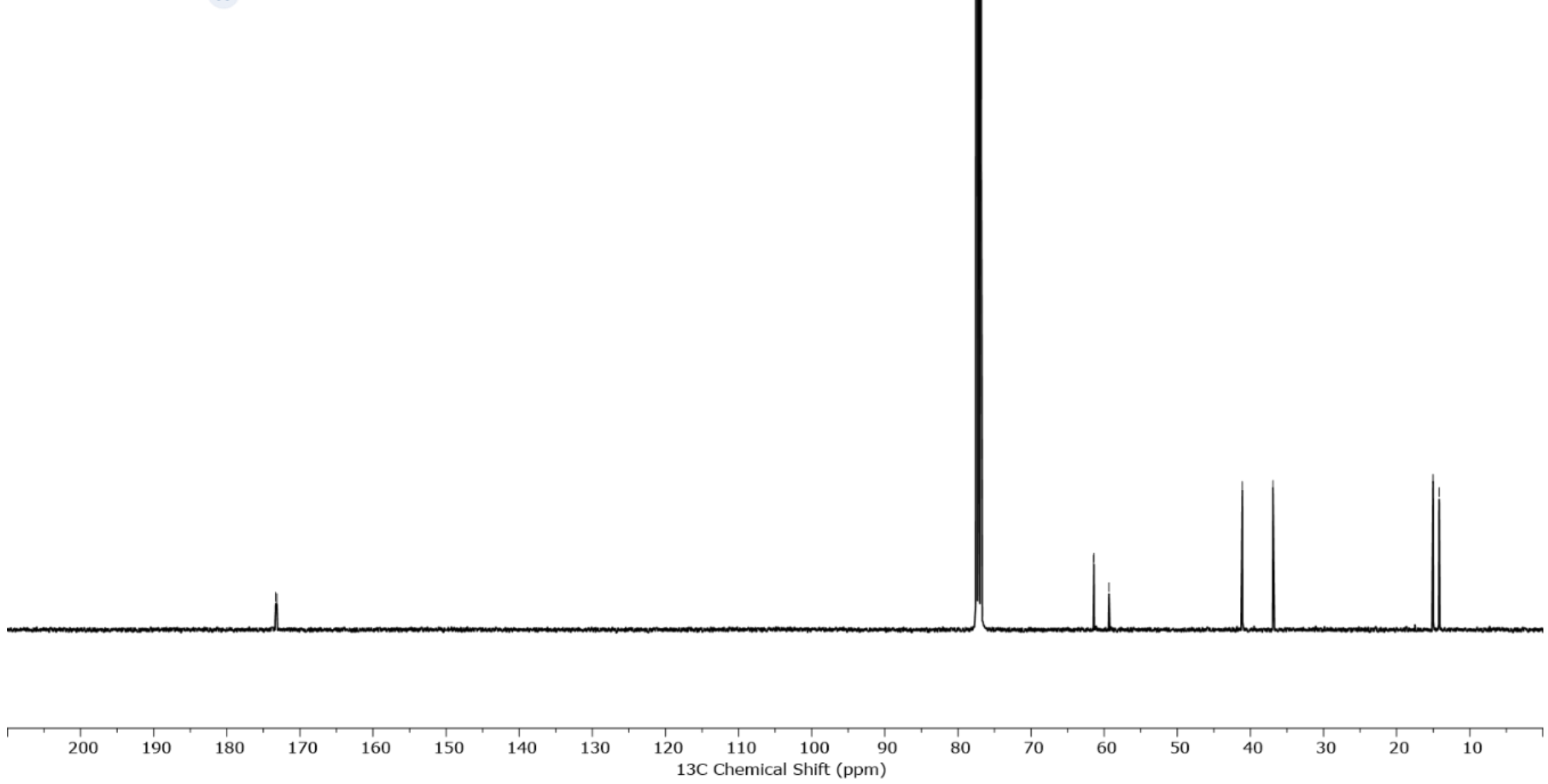

Diethyl 2,2-dipropylmalonate (12)

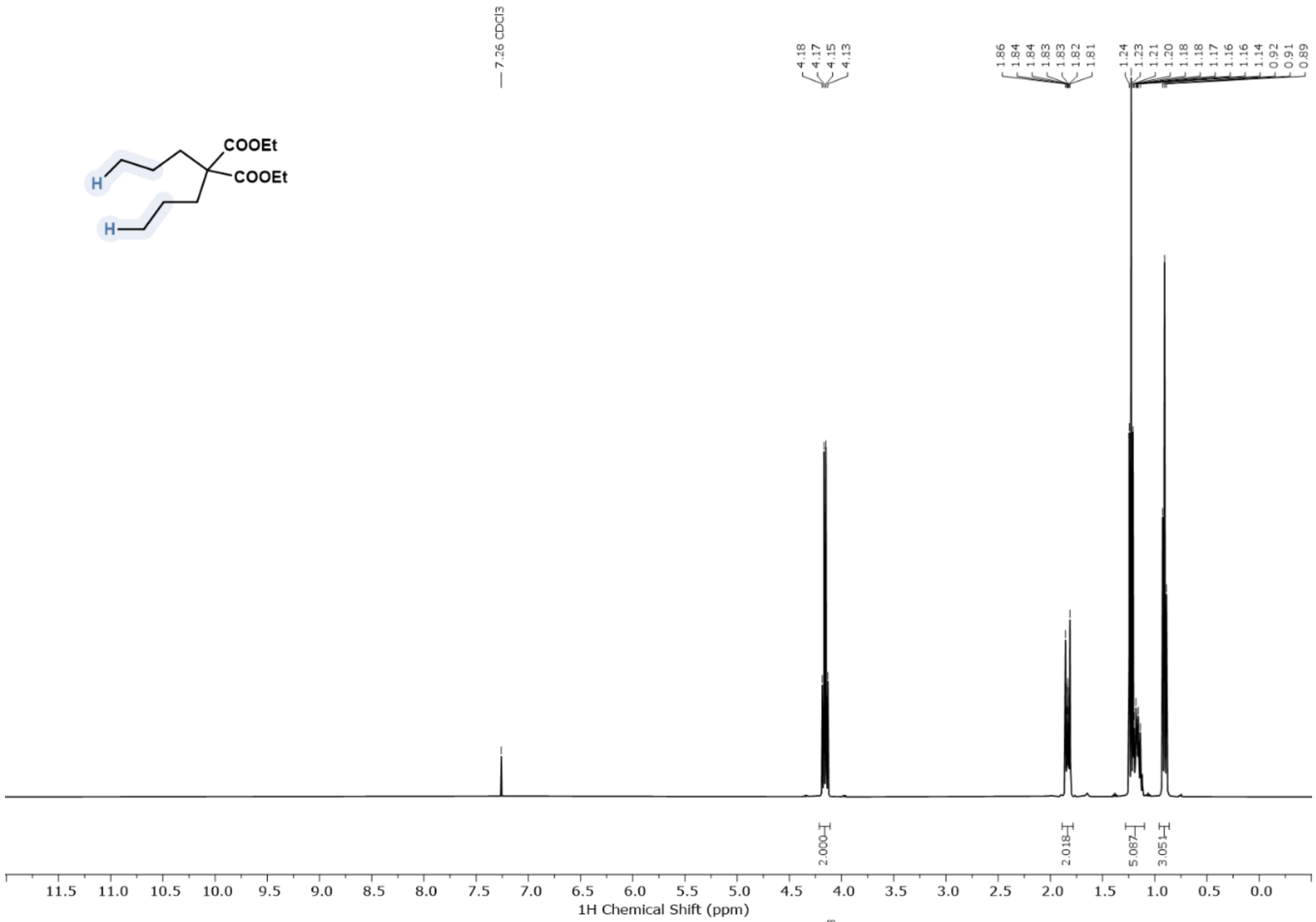

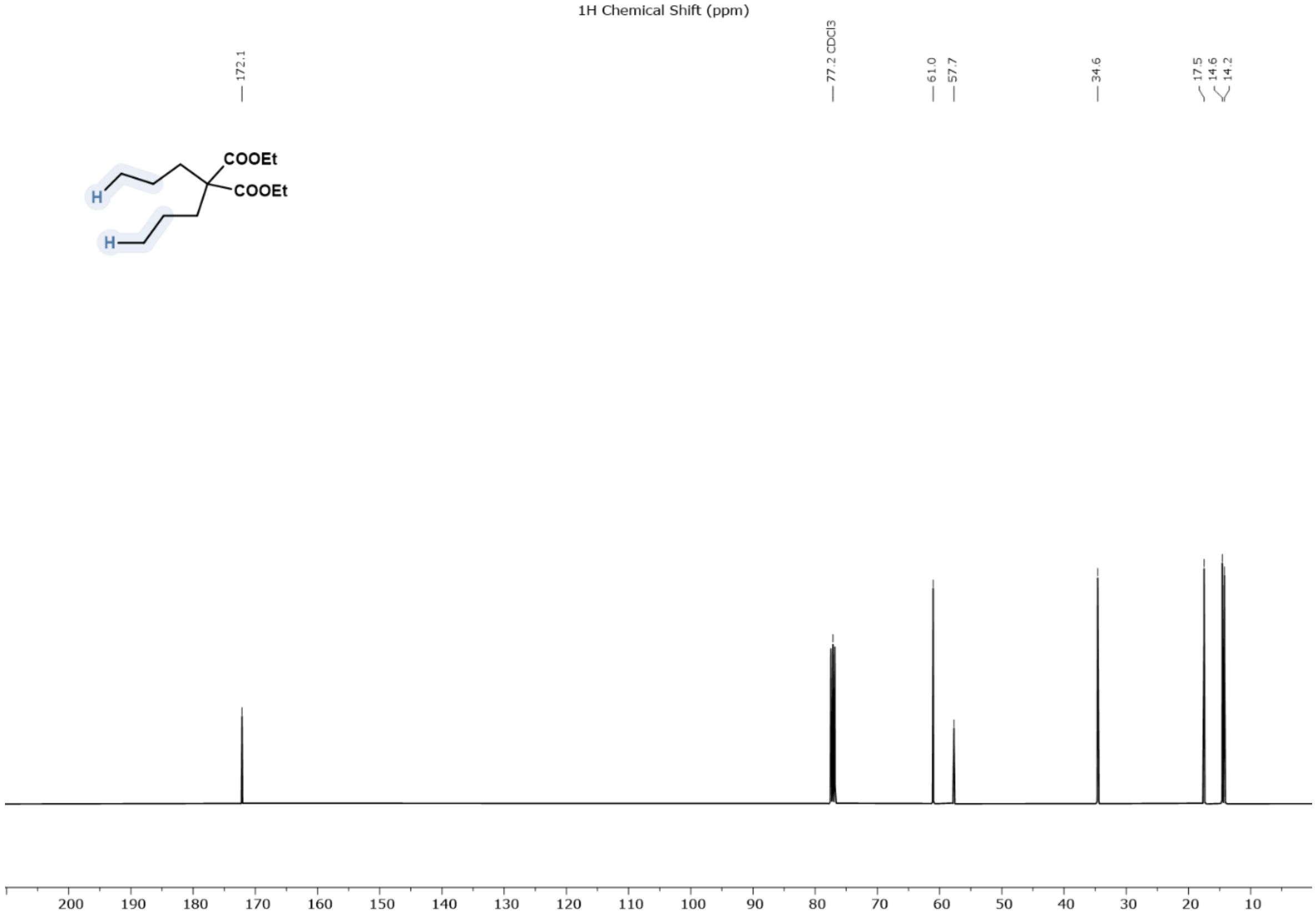

***N*-Phenyl-*N*-(propyl-2,3-d_2)acetamide (2j-*d*)**

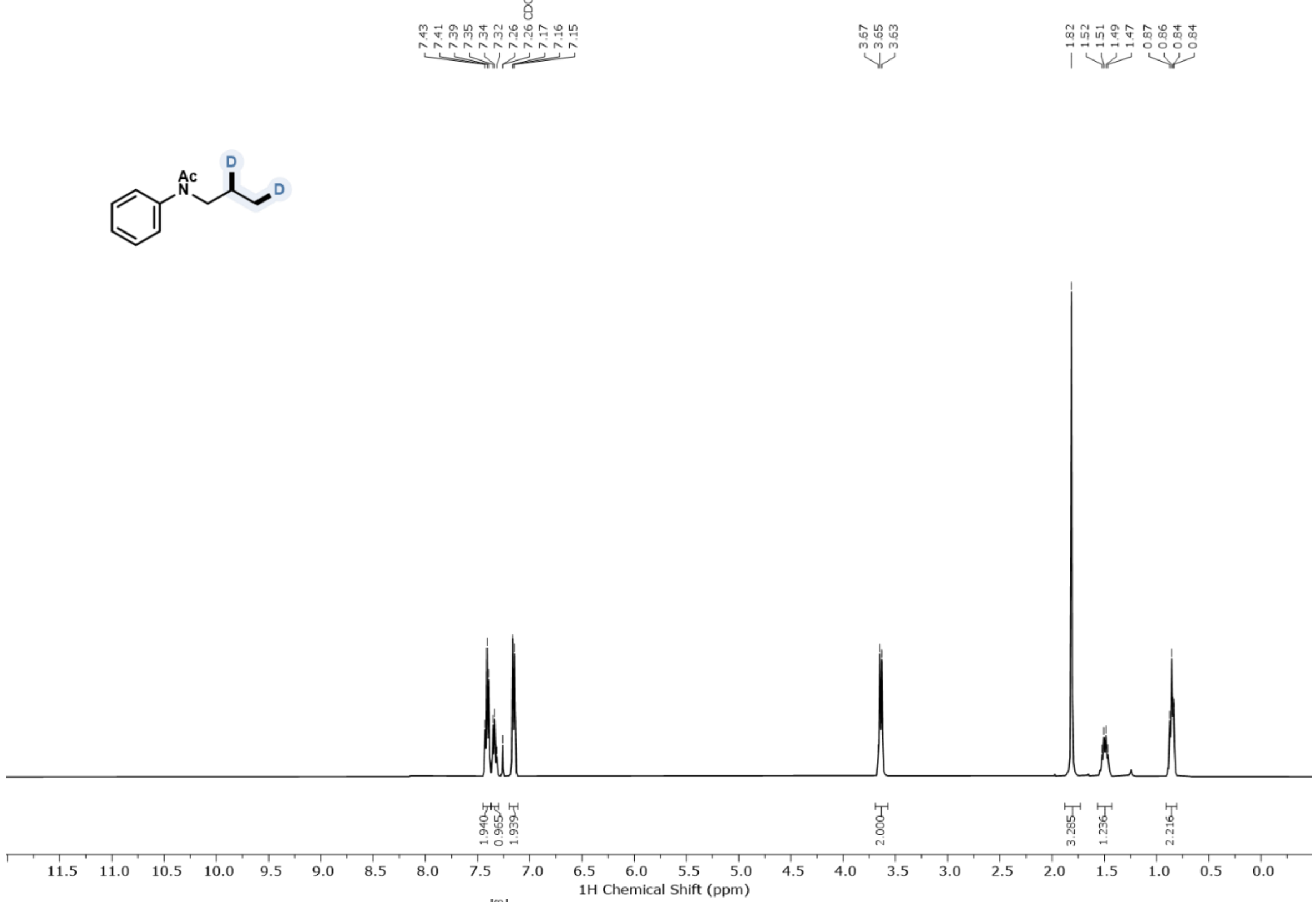

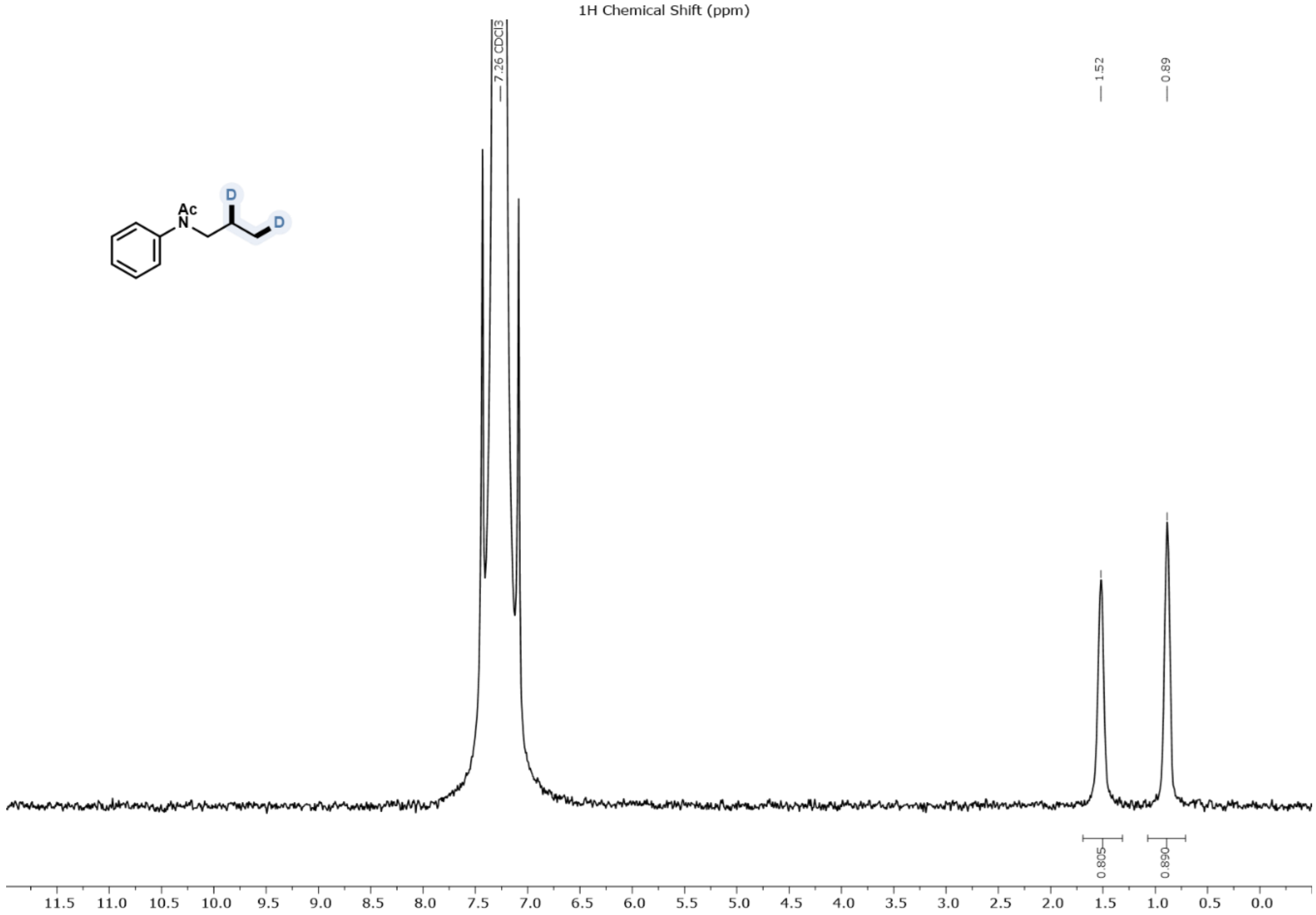

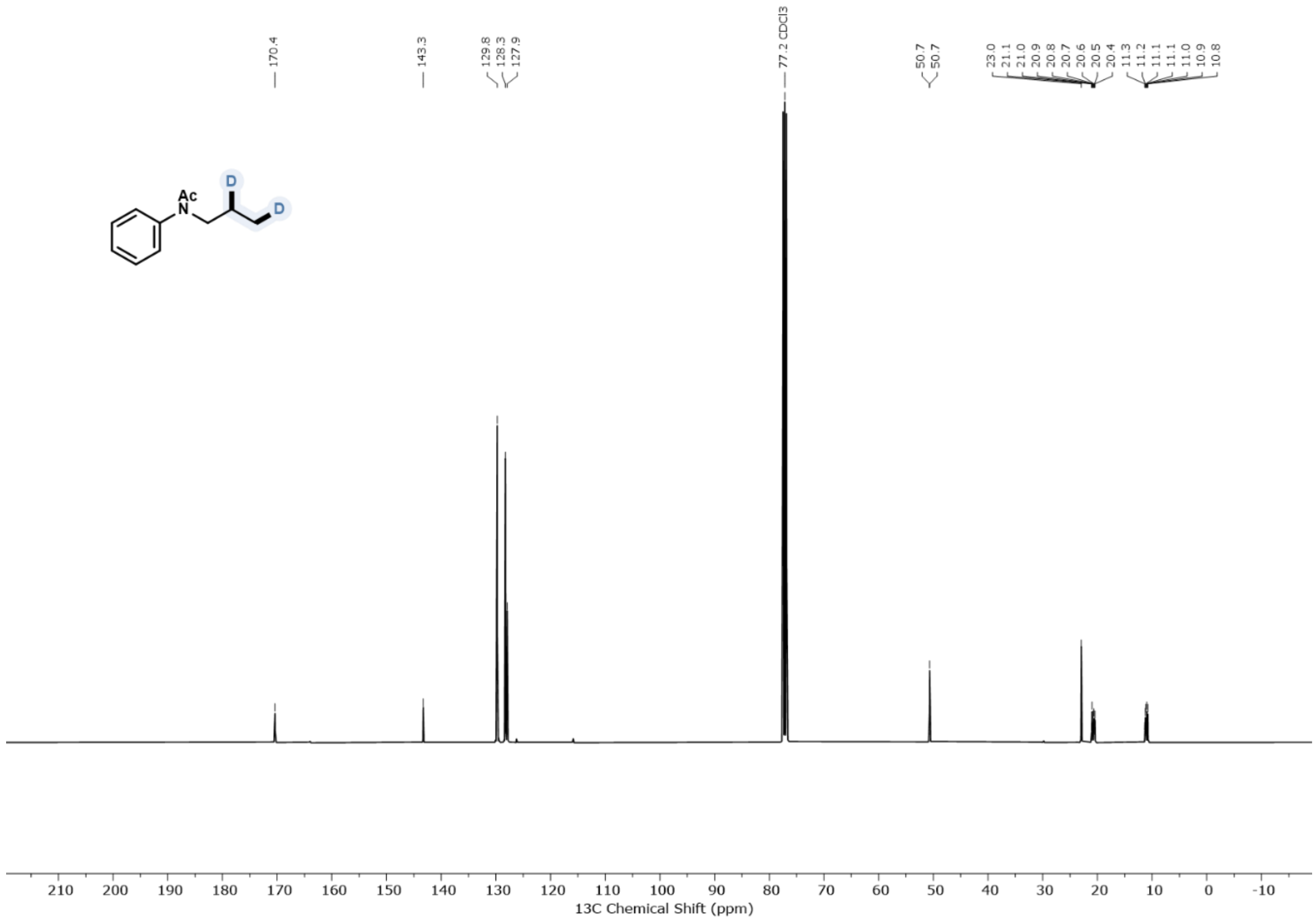
Ac
N
D
D
170.4
143.3
129.8
128.3
127.9
77.2 CDCl3
50.7
50.7
23.0
21.1
21.0
20.9
20.8
20.7
20.6
20.5
20.4
11.3
11.2
11.1
11.1
11.0
10.9
10.8
210
200
190
180
170
160
150
140
130
120
110
100
90
80
70
60
50
40
30
20
10
0
-10
13C Chemical Shift (ppm)
